# The histone code reader Spin1 controls skeletal muscle development

**DOI:** 10.1038/cddis.2017.468

**Published:** 2017-11-23

**Authors:** Holger Greschik, Delphine Duteil, Nadia Messaddeq, Dominica Willmann, Laura Arrigoni, Manuela Sum, Manfred Jung, Daniel Metzger, Thomas Manke, Thomas Günther, Roland Schüle

**Affiliations:** 1 Urologische Klinik und Zentrale Klinische Forschung, Klinikum der Universität Freiburg, Breisacher Str. 66, Freiburg, Germany; 2 IGBMC, Department of Functional Genomics and Cancer, Inserm U964, CNRS UMR7104, Université de Strasbourg, Illkirch, France; 3 Max-Planck-Institute of Immunology and Epigenetics, Stübeweg 51, Freiburg, Germany; 4 Institut für Pharmazeutische Wissenschaften, Albert-Ludwigs-Universität Freiburg, Albertstr. 25, Freiburg, Germany; 5 Deutsches Konsortium für Translationale Krebsforschung (DKTK), Standort Freiburg, Freiburg, Germany; 6 BIOSS Centre for Biological Signalling Studies, Albert-Ludwigs-Universität Freiburg, Schänzlestr. 18, Freiburg, Germany

## Abstract

While several studies correlated increased expression of the histone code reader Spin1 with tumor formation or growth, little is known about physiological functions of the protein. We generated Spin1^M5^ mice with ablation of *Spin1* in myoblast precursors using the *Myf5-Cre* deleter strain. Most Spin1^M5^ mice die shortly after birth displaying severe sarcomere disorganization and necrosis. Surviving Spin1^M5^ mice are growth-retarded and exhibit the most prominent defects in soleus, tibialis anterior, and diaphragm muscle. Transcriptome analyses of limb muscle at embryonic day (E) 15.5, E16.5, and at three weeks of age provided evidence for aberrant fetal myogenesis and identified deregulated skeletal muscle (SkM) functional networks. Determination of genome-wide chromatin occupancy in primary myoblast revealed direct Spin1 target genes and suggested that deregulated basic helix-loop-helix transcription factor networks account for developmental defects in Spin1^M5^ fetuses. Furthermore, correlating histological and transcriptome analyses, we show that aberrant expression of titin-associated proteins, abnormal glycogen metabolism, and neuromuscular junction defects contribute to SkM pathology in Spin1^M5^ mice. Together, we describe the first example of a histone code reader controlling SkM development in mice, which hints at Spin1 as a potential player in human SkM disease.

Spindlin1 (Spin1) is a histone code reader binding histone H3 trimethylated at lysine 4 (H3K4me3) with high affinity.^1, 2, 3^ H3K4me3 association is enhanced by the presence of asymmetrically dimethylated arginine 8 of histone H3^4^. Spin1 is highly expressed in several types of tumors^5, 6, 7^ and affects cell cycle, chromatin segregation, apoptosis, and transformation of cell lines, as well as tumor formation in nude mice.^6, 8, 9, 10, 11^ While these studies suggest important roles in cancer, physiological functions of Spin1 have only been subject to initial investigation. Mouse oocytes deficient for maternal *Spin1* undergo normal folliculogenesis, but fail to resume meiosis.^12^ Furthermore, mice with ubiquitous *Spin1* ablation die shortly after birth.^12^ However, tissue-restricted defects accounting for postnatal death have not been reported.

Skeletal muscle (SkM) is the most abundant tissue in vertebrates mediating support and movement and contributing to overall metabolism. SkM development is orchestrated by key transcription factors including Pax3 and Pax7, which are also required for muscle stem cell specification,^13, 14^ and the myogenic regulatory factors (MRFs) Myf5, MyoD (Myod1), Mrf4 (Myf6), and myogenin (Myog).^13, 14, 15^ MRFs are tissue-specific basic helix-loop-helix (bHLH) transcription factors acting as homodimers or as heterodimers with other bHLH transcription factors such as the ubiquitously expressed E-proteins E12/E47 (Tcf3), E2-2/ITF2 (Tcf4), and HEB/HTF4 (Tcf12).^14^

SkM fiber formation in mice comprises three successive phases, an embryonic wave from around embryonic day (E) 10.5 to E12.5, a fetal wave from around E14.5 to E17.5, and a postnatal period during which adult fibers are established.^14, 16, 17, 18^ Adult myofibers exhibit distinct contractile properties (slow- or fast-twitch), patterns of innervation, and metabolic activities (oxidative or glycolytic), which correlate with the expression of specific myosin heavy chain (MHC) isoforms.^19, 20^ Limb muscle of adult mice is composed of type I (slow, oxidative), type IIa (fast, oxidative), type IIx (fast, glycolytic), and type IIb (fast, glycolytic) fibers.^19, 20^ SkM mass and functions become compromised in disease and numerous gene mutations causing myopathies or muscular dystrophies have been documented.^21, 22, 23, 24, 25, 26, 27, 28^ Interestingly, selected fiber or muscle types preferentially degenerate in certain disease states.^29, 30^

In this study, we crossed mice harboring conditional *Spin1* alleles (*Spin1*^p/p^) with the *Myf5-Cre* deleter strain^31^ to ablate Spin1 in myogenic precursors. Most homozygous *Spin1*^p/p Myf5-Cre^ (hereafter termed Spin1^M5^) mice die shortly after birth, while surviving mice display severe growth retardation. Histological, transcriptome, and cistrome analyses provide evidence for aberrant fetal myogenesis and deregulated basic bHLH transcription factor networks around the onset of SkM defects. Furthermore, our observations suggest that altered expression of titin-associated proteins, aberrant glycogen metabolism, and defective NMJs contribute to SkM pathology in Spin1^M5^ mice. In summary, our data reveal a severe developmental defect caused by ablation of a histone code reader in SkM and hint at Spin1 as a potential player in human SkM disease.

## Results

### Loss of Spin1 in SkM results in postnatal lethality

**Figure 1:**
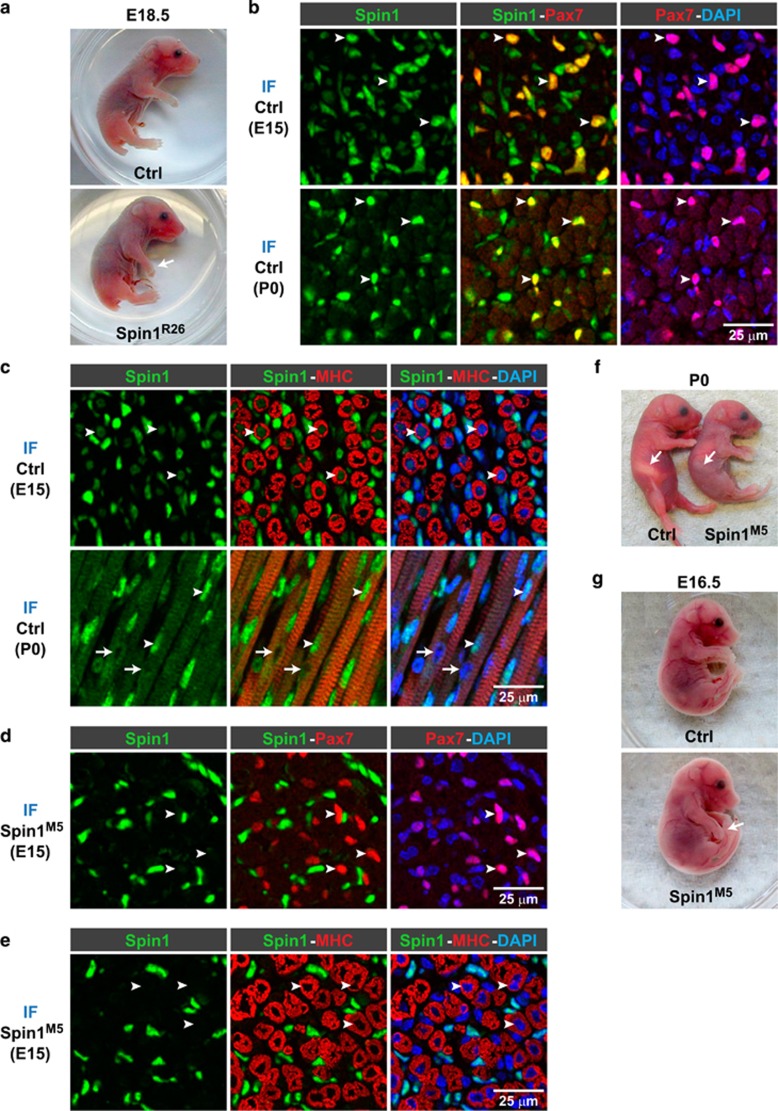
Loss of Spin1 in SkM results in postnatal lethality. (**a**) Dropping forelimbs (arrow) indicating a neuromuscular defect observed in ubiquitous *Spin1* knockout (Spin1^R26^) but not *Spin1*^+/+^ (Ctrl) fetuses. (**b**,**c**) Immunofluorescence (IF) staining of hind limb sections of control fetuses at E15 and of newborn mice (P0) with antibodies directed against Spin1 (green), Pax7 (red), or myosin heavy chain (MHC) (red). Arrowheads mark nuclei of myogenic precursors coexpressing Spin1 and Pax7 (**b**) or Spin1-positive nuclei in myofibers (**c**). Arrows depict nuclei devoid of Spin1 staining in myofibers at P0 (**c**). (**d**,**e**) IF staining of hind limb sections of Spin1^M5^ fetuses at E15 with the indicated antibodies. Loss of Spin1 expression in Pax7-positive nuclei of myogenic precursors (**d**) or myofibers (**e**) in Spin1^M5^ fetuses is highlighted with arrowheads. (**b-e**) Nuclei were visualized with DAPI (blue). (**f**) Absence of milk in the stomach (arrows) and an abnormal posture observed in newborn Spin1^M5^ mice but not control littermates. (**g**) Dropping forelimbs (arrow) observed in Spin1^M5^ but not control fetuses

To investigate physiological functions of Spin1 *in vivo*, we generated ubiquitous knockout (Spin1^R26^) mice by crossing *Spin1*^p/p^ mice with the *Rosa26-Cre* deleter strain^32^ (Supplementary Figure 1a). Spin1^R26^ mice were born, but died within one day after birth (Supplementary Figure 1b), which is in agreement with observations by others.^12^ Of note, at E18.5 Spin1^R26^ mice displayed dropping forelimbs (Figure 1a) indicating a neuromuscular defect.^33^

To address potential functions of Spin1 in SkM, we analyzed Spin1 protein expression in hind limb sections of control mice at E15 and after birth (P0) by immunofluorescence. At both time points, we observed intense Spin1 staining in Pax7-positive myoblast precursors (Figure 1b (arrowheads)) and weaker staining in nuclei of myofibers (Figure 1c (arrowheads)). Of note, in newborn mice, Spin1 expression was undetectable in some nuclei of myofibers (Figure 1c, bottom row (arrows)).

Next, we deleted *Spin1* in myoblast precursors by crossing *Spin1*^p/p^ mice with the *Myf5-Cre* deleter strain^31^ resulting in Spin1^M5^ mice. Immunostaining confirmed the absence of Spin1 protein in nuclei of Pax7-positive myoblast precursors (Figure 1d (arrowheads); Supplementary Figure 1c) and myofibers of Spin1^M5^ fetuses (Figure 1e (arrowheads); Supplementary Figure 1d). Remaining Spin1 staining is due to expression in non-myogenic cells such as Tcf4-positive fibroblasts^34^ (Supplementary Figure 1e (arrowheads); see Materials and Methods and Supplementary Figure 1f, g for further characterization).

Homozygous Spin1^M5^ mice were obtained at the expected Mendelian ratio at birth (Supplementary Figure 1h). However, about 80% of Spin1^M5^ mice died within one day after birth. Newborn Spin1^M5^ mice could typically be distinguished from control littermates by an abnormal posture and the absence of milk in the stomach (Figure 1f). Moreover, at E16.5, we observed dropping forelimbs for Spin1^M5^ fetuses (Figure 1g). Together, our data show that ablation of Spin1 in SkM causes early postnatal death of the majority of mice.

### SkM of Spin1^M5^ mice is characterized by necrosis and structural defects in non-necrotic fibers

**Figure 2:**
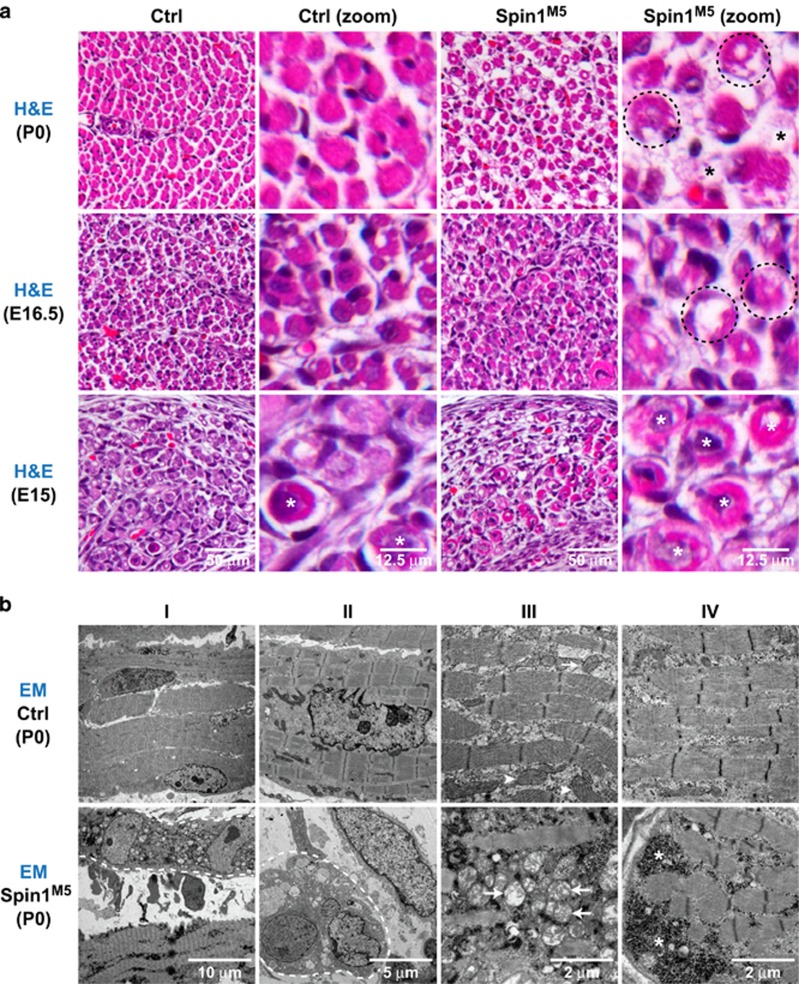
SkM of Spin1^M5^ mice is characterized by necrosis and structural defects in non-necrotic fibers. (**a**) Hematoxylin and eosin (H&E) staining of transversal tibialis anterior sections of Spin1^M5^ and control mice at P0, E16.5, and E15. Fiber loss in Spin1^M5^ mice is indicated by black asterisks (top row), fibers with irregular H&E staining are encircled, and unusually large fibers more frequently observed in Spin1^M5^ than in control fetuses at E15 are marked with white asterisks (bottom row). (**b**) Electron microscopy (EM) images of SkM samples of newborn (P0) Spin1^M5^ and control mice. Dashed lines demarcate degenerating, necrotic fibers (I–II, bottom), arrowheads mark normal mitochondria (III, top), arrows point at defective mitochondria (III, bottom), and asterisks indicate abnormal glycogen accumulation (IV, bottom)

To characterize SkM defects in Spin1^M5^ mice, we inspected hematoxylin & eosin (H&E)-stained hind limb sections at different stages of development. Compared with control littermates, we observed in newborn Spin1^M5^ mice loss of fibers (Figure 2a, top row (black asterisks)) and numerous immature or degenerating fibers lacking contractile material (dashed circles). In Spin1^M5^ fetuses at E16.5, we also noted fibers with irregular H&E staining (Figure 2a, middle row (dashed circles)) and at E15 enlarged fibers, which were less abundant in control samples (Figure 2a, bottom row (white asterisks)).

Electron microscopy analyses of hind limb sections revealed for newborn Spin1^M5^ mice degenerating, necrotic fibers (Figure 2b, columns I–II (demarcated by dashed lines)), defective mitochondria (Figure 2b, column III (arrows)), and abnormal glycogen accumulation (Figure 2b, column IV (asterisks)). Similar defects were detected for Spin1^M5^ fetuses at E16.5 (Supplementary Figure 2). Furthermore, in non-necrotic fibers of Spin1^M5^ fetuses, we observed structural defects including a low density of contractile material and the lack of a clear M-line (Supplementary Figure 2, column III (triangles)). Together, our H&E and electron microscopy analyses uncovered necrotic and structurally defective fibers in Spin1^M5^ mice. In addition, the data suggested an onset of SkM defects before E16.5.

### Transcriptome and histological analyses provide evidence for aberrant fetal myogenesis in Spin1^M5^ mice

**Figure 3:**
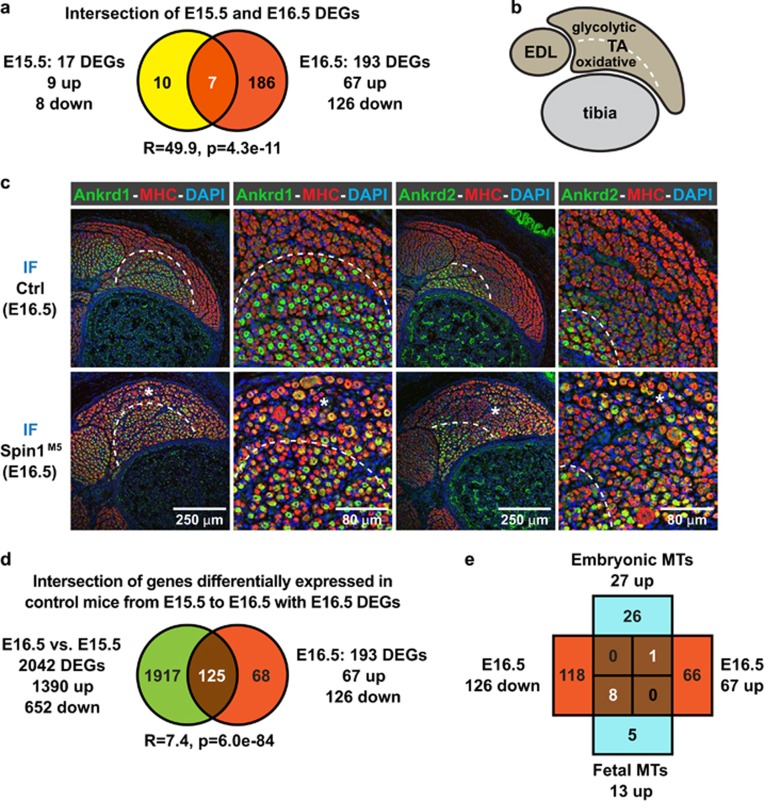
Transcriptome and histological analyses provide evidence for aberrant fetal myogenesis in Spin1^M5^ mice. (**a**) Intersection of differentially expressed genes (DEGs) observed for SkM of Spin1^M5^ and control fetuses at E15.5 (yellow) and E16.5 (red). (R: enrichment factor; p: *P*-value calculated for the intersection.) (**b**) Schematic representation of the hind limb section depicting tibialis anterior (TA), extensor digitorum longus (EDL), and tibia analyzed in **c**. Prospective oxidative or glycolytic parts of the TA (demarcated by a dashed line) are located proximal or distal to the tibia, respectively. (**c**) Detection of Ankrd1 or Ankrd2 (green) by immunofluorescence (IF) staining in transversal hind limb sections of Spin1^M5^ and control fetuses at E16.5. Muscle fibers were visualized with myosin heavy chain (MHC) antibody (red), nuclei were stained with DAPI (blue). Dashed lines demarcate areas with or without Ankrd1 or Ankrd2 expression in control fetuses. Asterisks mark regions of the TA of Spin1^M5^ fetuses, in which Ankrd1 or Ankrd2 are aberrantly expressed. (**d**) Intersection of the E16.5 DEGs (red) with genes differentially expressed in control fetuses at E15.5 and E16.5 (green). (**e**) Comparison of the E16.5 DEGs (red) with transcripts reported to be more highly expressed in either embryonic or fetal myotubes (MTs, cyan)^37^

To investigate alterations of the transcriptome in SkM of Spin1^M5^ fetuses, we performed RNA sequencing (RNA-seq) analyses using RNA isolated from limb muscle. At E15.5, we observed only 17 differentially expressed genes (DEGs) (*P*≤1e−3; fold change ≥1.5) (Figure 3a; Supplementary Table 1a). In comparison, at E16.5 RNA-seq detected 193 DEGs, of which seven (*Ankrd1, Ankrd2, Pmaip1, Scn4b* (upregulated); *Myf5, Msc, Nos1* (downregulated)) overlapped with the E15.5 DEGs (Figure 3a; Supplementary Table 1b). The strong increase in DEGs from E15.5 to E16.5 provided evidence for E15.5 as the approximate onset of SkM defects in Spin1^M5^ fetuses.

The common E15.5/E16.5 DEGs *Ankrd1* (CARP) and *Ankrd2* (ARPP) encode titin-associated regulators of sarcomere function, whose expression is often deregulated in SkM disease.^35^ We therefore investigated Ankrd1 and Ankrd2 protein levels in hind limb sections of E16.5 fetuses by immunofluorescence (Figures 3b and c). Most prominently, in control fetuses, expression of both proteins was restricted to the inner (prospective oxidative) part of the tibialis anterior (TA) neighboring the tibia (Figure 3c (demarcated by dashed lines)), whereas in Spin1^M5^ fetuses staining was also present in the outer (prospective glycolytic) part of the TA (Figure 3c (asterisks)). In comparison, at E15 Ankrd1 and Ankrd2 staining of hind limb muscle of Spin1^M5^ and control mice was similar (Supplementary Figures 3a and b). Thus, deregulation of *Ankrd1* and *Ankrd2* transcripts at E15.5 and E16.5 and aberrant protein expression at E16.5 are early markers of SkM defects in Spin1^M5^ fetuses.

To analyze which proportion of developmentally regulated genes is affected by the absence of Spin1, we compared the E16.5 DEGs with gene expression changes in control fetuses from E15.5 to E16.5. In control fetuses, we observed 2042 genes differentially expressed between E15.5 and E16.5 (Figure 3d; Supplementary Table 1c). Phenotype and pathway analyses for these 2042 DEGs using WebGestalt^36^ confirmed relevance of the identified genes for SkM development or function (Supplementary Figures 3c and d). Intersection with the 193 E16.5 DEGs revealed an overlap of 125 genes (Figure 3d). Therefore, the absence of Spin1 prevents adequate expression of a subset of genes regulated during the fetal phase of myogenesis in control mice.

To corroborate aberrant fetal myogenesis in Spin1^M5^ mice, we compared the E16.5 DEGs with transcripts previously observed to be differentially expressed in embryonic or fetal myotubes.^37^ Of the 27 transcripts more highly expressed in embryonic (compared with fetal) myotubes, only one (*Tnnc1*) overlapped with the E16.5 DEGs (Figure 3e). Importantly, eight out of thirteen transcripts more highly expressed in fetal myotubes (*Myh8, Tnni2, Tnnt3, Atp2a1, Casq1, Pvalb, Ckm,* and *Eno3*) were downregulated DEGs in Spin1^M5^ mice at E16.5 (Figure 3e). Together, our data provided evidence for an impaired progression of fetal myogenesis in Spin1^M5^ mice.

### Identification of deregulated SkM functional networks in Spin1^M5^ fetuses

**Figure 4:**
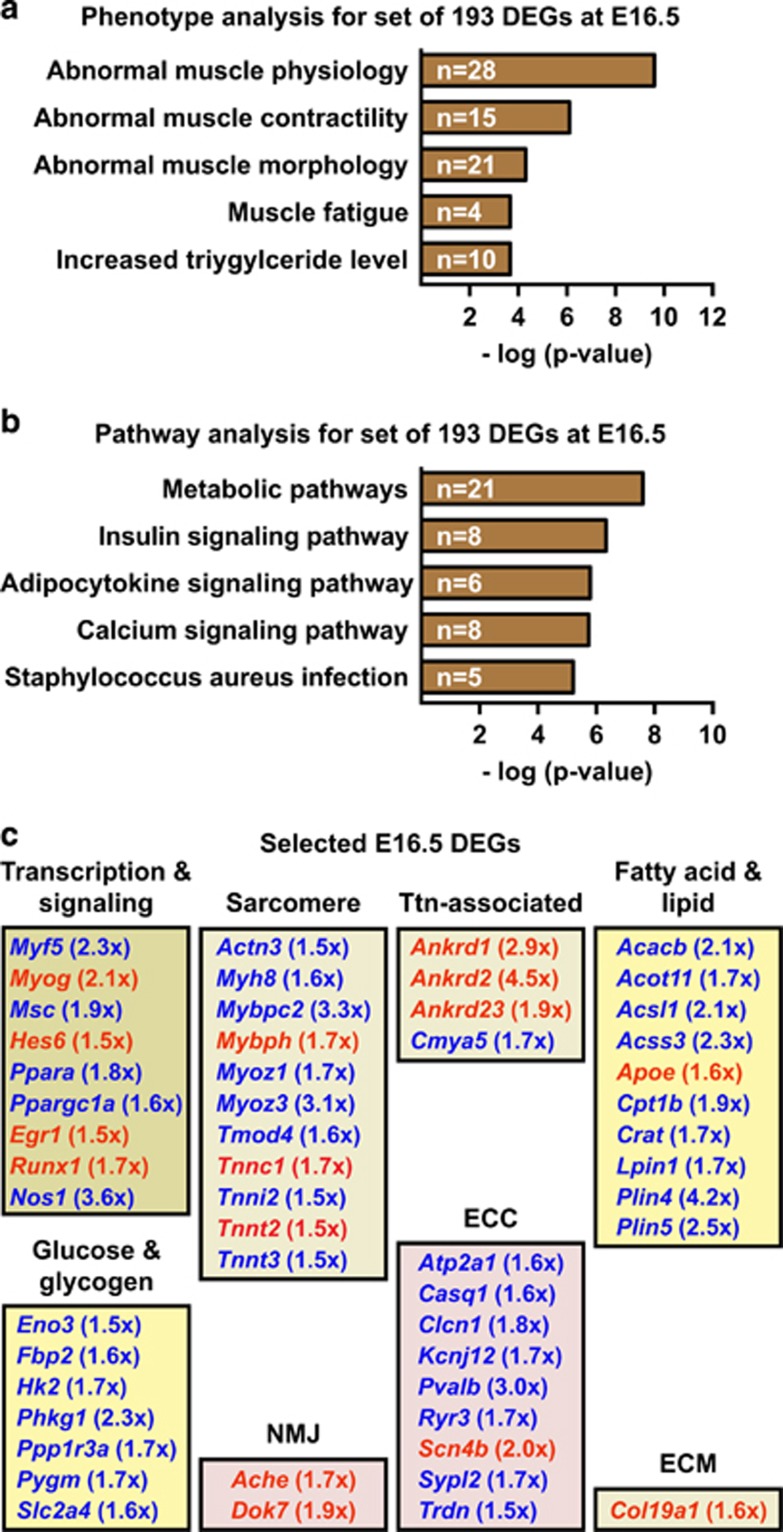
Identification of deregulated SkM functional networks in Spin1^M5^ fetuses. (**a**,**b**) Phenotype (**a**) and pathway (**b**) analysis for the set of 193 DEGs observed in SkM of Spin1^M5^ fetuses at E16.5. The number of genes in each category is indicated. For the phenotype analysis, the ‘top 5’ non-redundant terms are depicted. (**c**) Assignment of selected E16.5 DEGs identified by phenotype and pathway analyses to functional SkM networks. Upregulated DEGs are depicted in red, downregulated in blue color (fold change in brackets)

To identify genes accounting for SkM defects in Spin1^M5^ fetuses, we performed phenotype and pathway analyses for the E16.5 DEGs using WebGestalt^36^ (Figures 4a and b). These analyses revealed terms related to SkM function, metabolism, and pathology. We grouped the genes associated with the phenotype and pathway terms according to functional networks previously proposed in systems biology analyses for SkM^38, 39^ (Figure 4c). The deregulated SkM networks comprise genes encoding transcription factors and signaling molecules, proteins important for sarcomere function, titin (Ttn)-associated factors, metabolic enzymes, and proteins involved in the neuromuscular junction (NMJ) and excitation-contraction coupling (ECC) (Figure 4c). Deregulation of these functional networks likely causes SkM dysfunction in Spin1^M5^ fetuses.

### Deregulated basic helix-loop-helix transcription factor networks account for SkM defects in Spin1^M5^ fetuses

In addition to genes required for SkM structure or function, we found the myogenic bHLH transcription factor-encoding genes *Myf5*, *Myog*, *Msc* (musculin, MyoR), and *Hes6*^15, 40, 41, 42, 43^ among the E16.5 DEGs (Figure 4c (network 'transcription and signaling')). Therefore, deregulated bHLH transcription factor-controlled gene expression may be an early event accounting for aberrant myogenesis in Spin1^M5^ fetuses. In support of this idea, *Myf5* and *Msc* were also among the E15.5 DEGs (Supplementary Table 1a).

**Figure 5:**
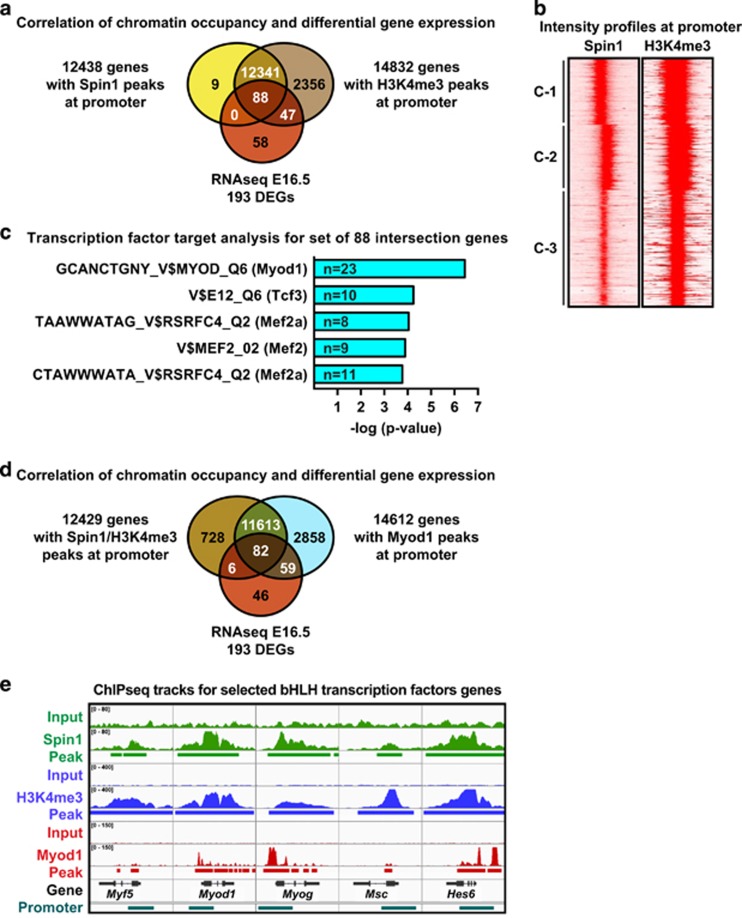
Deregulated basic helix-loop-helix transcription factor networks account for SkM defects in Spin1^M5^ fetuses. (**a**) Intersection of genes with Spin1 peaks (yellow) or H3K4me3 peaks (brown) at the promoter (transcription start site (TSS)±1 kb) with the E16.5 DEGs (red). (**b**) Intensity profiles for Spin1 and H3K4me3 occupancy of 12 429 gene promoters (TSS±1 kb) (*K*_Means_=3 clusters (C-1 to C-3)). (**c**) Transcription factor target analysis for 88 E16.5 DEGs with Spin1 and H3K4me3 peaks at the promoter observed by intersection of data sets shown in (**a**). (**d**) Intersection of genes with Spin1 and H3K4me3 peaks (brown) or Myod1 peaks (cyan) at the promoter (TSS±1 kb) with the E16.5 DEGs (red). (**e**) ChIP-seq tracks for selected genes encoding myogenic bHLH transcription factors. Spin1 (green) and H3K4me3 (blue) tracks were obtained by ChIP-seq analysis of primary myoblasts, Myod1 (red) tracks were obtained by analysis of previously deposited ChIP-seq data for C2C12 myoblasts (GEO data set GSE36024). Identified peaks and promoters (TSS±1 kb) are indicated by bars

To correlate gene expression with Spin1 chromatin occupancy, we performed chromatin immunoprecipitation followed by massive parallel sequencing (ChIP-seq) using primary myoblasts. ChIP-seq with Spin1-specific antibody revealed 17106 peaks, of which the majority (61.2%) was located at the promoters (transcription start site (TSS)±1 kb) of 12438 genes (Figure 5a; Supplementary Figure 4a). Next, ChIP-seq analysis for H3K4me3 detected 37% of the peaks at the promoters of 14832 genes (Figure 5a; Supplementary Figure 4a). Further analyses confirmed the presence of H3K4me3 at almost all gene promoters occupied by Spin1 (Figure 5a) as well as overlapping intensity profiles (Figure 5b). Thus, in primary myoblasts Spin1 and H3K4me3 occupy a large portion of cellular gene promoters.

Despite occupying thousands of gene promoters, Spin1 apparently only regulates a subset of target genes during fetal myogenesis. To further investigate Spin1-mediated gene regulation, we intersected the E16.5 DEGs with the cistrome observed in primary myoblasts. This analysis revealed the presence of Spin1 and H3K4me3 at the promoter of 88 E16.5 DEGs (Figure 5a). A transcription factor target analysis for these 88 genes using WebGestalt^36^ revealed a significant enrichment of potential Myod1 target genes followed by E12 (Tcf3) and Mef2 targets (Figure 5c).

To validate Myod1 promoter occupancy of Spin1 target genes, we analyzed a previously deposited Myod1 ChIP-seq data set for C2C12 myoblasts (GEO data set GSE36024), which uncovered Myod1 peaks at the promoters of 14612 genes (Figure 5d). Intersection with Spin1-occupied genes showed a large overlap of 11695 direct targets of Spin1 and Myod1 (Figure 5d). Comparison with the E16.5 DEGs identified 82 direct Spin1/Myod1 targets (Figure 5d). Inspection of ChIP-seq tracks confirmed the presence of Spin1, H3K4me3, and Myod1 at promoters of SkM functional, metabolic, and regulatory genes (Supplementary Figure 4b) as well as the promoters of *Myf5*, *Myod1*, *Myog*, *Msc*, and *Hes6* (Figure 5e). Together, our data suggest that deregulated bHLH transcription factors affect Spin1/Myod1-dependent target gene transcription thereby accounting, at least in part, for SkM defects in Spin1^M5^ fetuses.

### Surviving Spin1^M5^ mice exhibit major defects in soleus, tibialis anterior, and diaphragm

**Figure 6:**
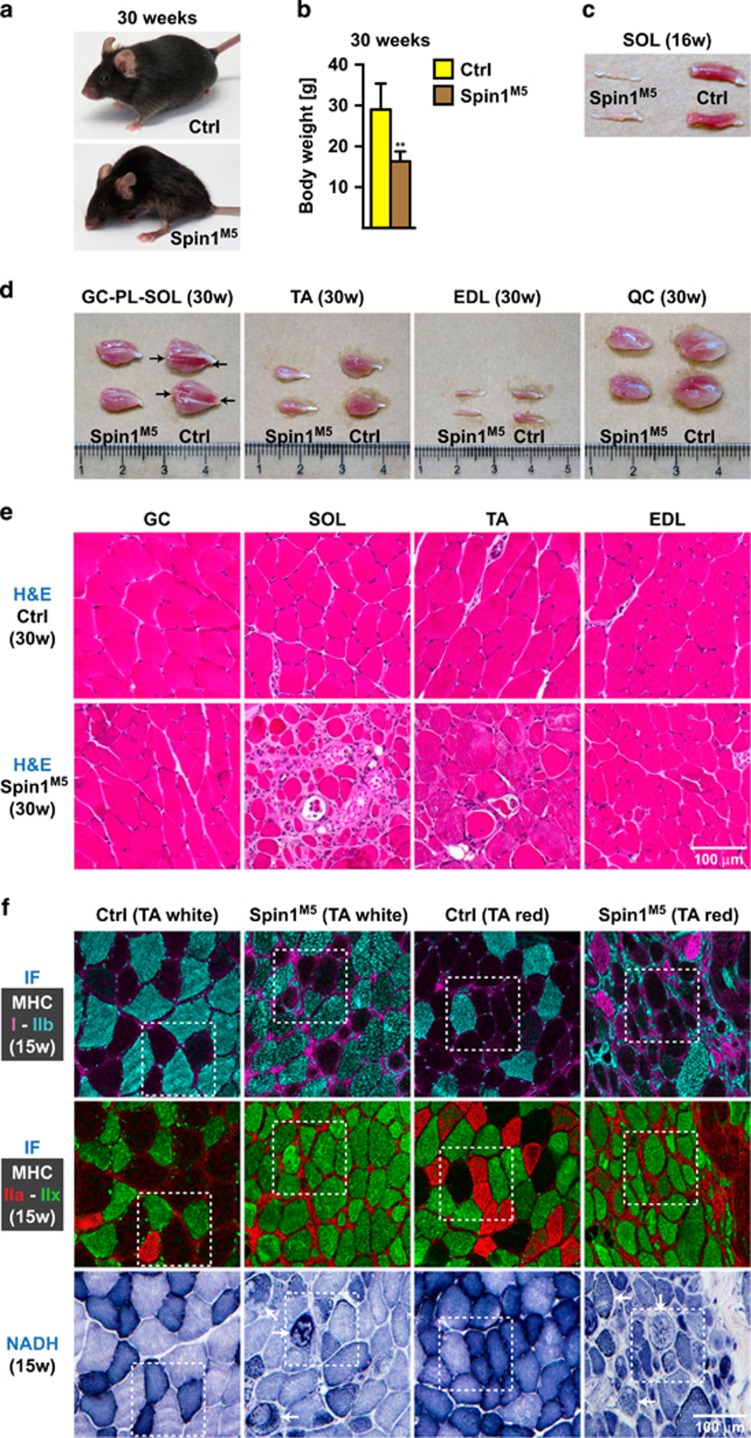
Surviving Spin1^M5^ mice exhibit major defects in soleus, tibialis anterior, and diaphragm. (**a,b**) Appearance (**a**) and average body weight (**b**) of Spin1^M5^ and control mice (*n*=5 females in each category) at 30 weeks of age. Error bars represent +S.D., **P<0.01. (**c**) Degeneration of the soleus (SOL) muscle in adult Spin1^M5^ compared with control mice exemplified at 16 weeks of age. (**d**) Hind limb muscles (gastrocnemius (GC), plantaris (PL), soleus, tibialis anterior (TA), extensor digitorum longus (EDL), and quadriceps (QC)) of Spin1^M5^ and control mice at 30 weeks of age. Arrows point at the soleus embedded in gastrocnemius and plantaris, which is visible in control but degenerated in Spin1^M5^ mice. (**e**) Hematoxylin & eosin (H&E) staining of gastrocnemius, soleus, TA, and EDL muscle of Spin1^M5^ and control mice at 30 weeks of age. (**f**) Fiber types in glycolytic (white) or oxidative (red) parts of the TA of Spin1^M5^ and control mice at 15 weeks of age (top and middle rows) observed by immunofluorescence (IF) staining. Tissue sections were stained with selective antibody directed against MHC-I (purple), MHC-IIb (cyan), MHC-IIa (red), and MHC-IIx (green). For comparison, NADH staining was included (bottom row). Fibers with abnormal NADH staining are marked with arrows. Corresponding fibers in each column of images are squared

While the majority of Spin1^M5^ mice died shortly after birth, about 20% survived and reached adulthood. We next investigated the consequences of aberrant fetal myogenesis for SkM in surviving Spin1^M5^ mice between 15 and 30 weeks of age, the latter representing the oldest cohort obtained. Adult Spin1^M5^ mice exhibit a severe scoliosis, cachexia, and weigh only around 60% of control littermates (Figures 6a and b).

Inspection of hind limb muscles revealed as the most prominent feature an abnormally thin and pale soleus (SOL) muscle in Spin1^M5^ mice (Figure 6c). Other hind limb muscles (gastrocnemius (GC), plantaris (PL), TA, extensor digitorum longus (EDL), and quadriceps (QC)) also displayed a reduced mass relative to controls (Figure 6d; Supplementary Figure 5a). The muscle mass reduction was more pronounced (about 60%) for TA than for gastrocnemius-plantaris, EDL, or quadriceps (about 30%) (Supplementary Figure 5a). Counting showed that the number of TA fibers was reduced by about 50%, but did not uncover a significant difference for the EDL of Spin1^M5^ compared with control mice (Supplementary Figure 5b).

H&E staining confirmed degeneration of the soleus as evidenced by rounded fibers, enormous differences in fiber diameters, and the presence of inflammatory cells (Figure 6e). Major defects were also observed in the TA, while gastrocnemius and EDL appeared largely normal (Figure 6e). Also, the diaphragm (DP) of Spin1^M5^ mice displayed severe muscle fiber degeneration (Supplementary Figure 5c). Gomori staining detected abnormal collagen deposition indicating fibrosis mainly in the soleus and TA muscle of Spin1^M5^ mice (Supplementary Figure 5d). Together, our observations revealed severe defects in soleus, TA, and diaphragm.

To investigate whether degenerative changes affected specific fiber types, we analyzed SkM sections by immunofluorescence. The TA of control mice exhibited typical fiber type compositions, that is, mainly type IIb fibers in white (primarily glycolytic) TA and type IIa and IIx fibers in red (primarily oxidative) TA (Figure 6f, top and middle rows). NADH staining correlated with the glycolytic or oxidative properties of the fiber types (Figure 6f, bottom row). In comparison, in the TA of some Spin1^M5^ mice, fiber types could not be clearly distinguished since most fibers expressed varying levels of MHC-IIx, in part together with MHC-IIb (Figure 6f). We rarely observed type IIa fibers in the TA of these animals (Figure 6f). However, in the TA of other Spin1^M5^ mice type IIa fibers were present (Supplementary Figure 5e, right column). Furthermore, all expected fiber types were observed in the degenerating soleus and the neighboring plantaris of Spin1^M5^ mice (Supplementary Figure 5e, left and middle columns), although defective fibers could be identified by irregular fiber size, shape, or NADH staining (Figure 6f; Supplementary Figure 5e, bottom row (arrows)). Overall, these observations argue against selective fiber type degeneration in Spin1^M5^ mice, but rather suggest that certain muscles including soleus, TA, and diaphragm degenerate.

### Identification of deregulated SkM functional networks in surviving Spin1^M5^ mice

**Figure 7:**
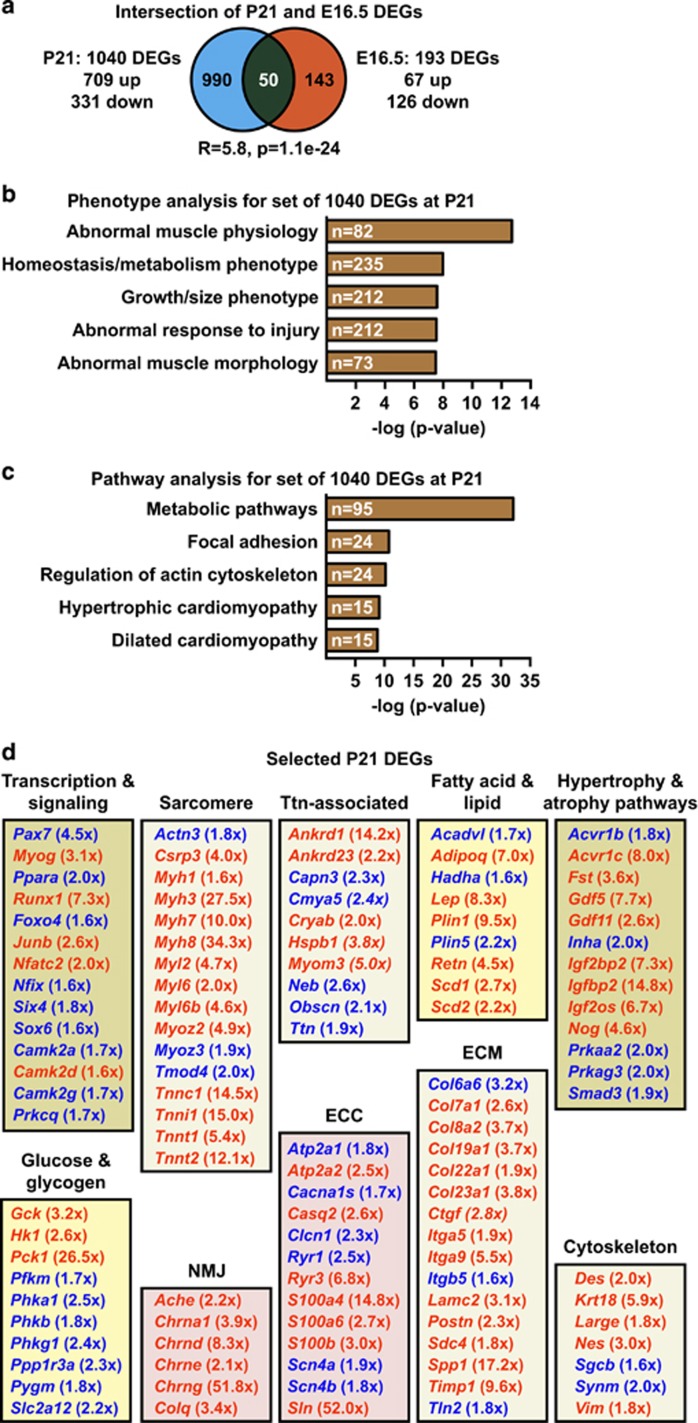
Identification of deregulated SkM functional networks in surviving Spin1^M5^ mice. (**a**) Intersection of DEGs observed for TA of Spin1^M5^ mice at P21 (blue) and SkM at E16.5 (red). (R: enrichment factor; p: *P*-value calculated for the intersection.) (**b,c**) Phenotype (**b**) and pathway (**c**) analysis for the set of 1040 DEGs observed in the TA of Spin1^M5^ mice at P21. For the phenotype analysis, the ‘top 5’ non-redundant terms are depicted. (**d**) Assignment of selected P21 DEGs identified by phenotype and pathway analyses to functional SkM networks. Upregulated DEGs are depicted in red, downregulated in blue color (fold change in brackets)

We next analyzed the transcriptomes of TA of Spin1^M5^ and control mice at three weeks of age (P21), which revealed 1040 DEGs (*P*≤1e−5; fold change ≥1.5; Figure 7a; Supplementary Table 1d). Intersection with the E16.5 DEGs detected a highly significant overlap of 50 genes (Figure 7a) suggesting that defects in surviving Spin1^M5^ mice are related to differential gene expression at early stages of disease.

Phenotype and pathway analyses for the P21 DEGs uncovered terms related to muscle function and disease, as well as metabolism (Figures 7b and c). In addition, we noted terms such as ‘focal adhesion’ and ‘regulation of actin cytoskeleton’, which were not identified at E16.5 (Figures 4a and b). Grouping of the P21 DEGs according to systems biology classifications^38, 39^ revealed the same deregulated SkM networks (Figure 7d) as for the E16.5 DEGs (Figure 4c). At P21, however, the networks contained more DEGs than at E16.5, and we identified additional networks ('hypertrophy & atrophy pathways' and 'cytoskeleton'). Of note, the strong increase in deregulated genes including collagen isoforms in the network 'extracellular matrix (ECM)' (Figure 7d) correlates with fibrosis detected in the TA of adult Spin1^M5^ mice (Supplementary Figure 5d). Together, this analysis provided a comprehensive disease signature for the TA of surviving Spin1^M5^ mice.

### Differential gene expression in surviving Spin1^M5^ mice correlates with SkM disease patterns

**Figure 8:**
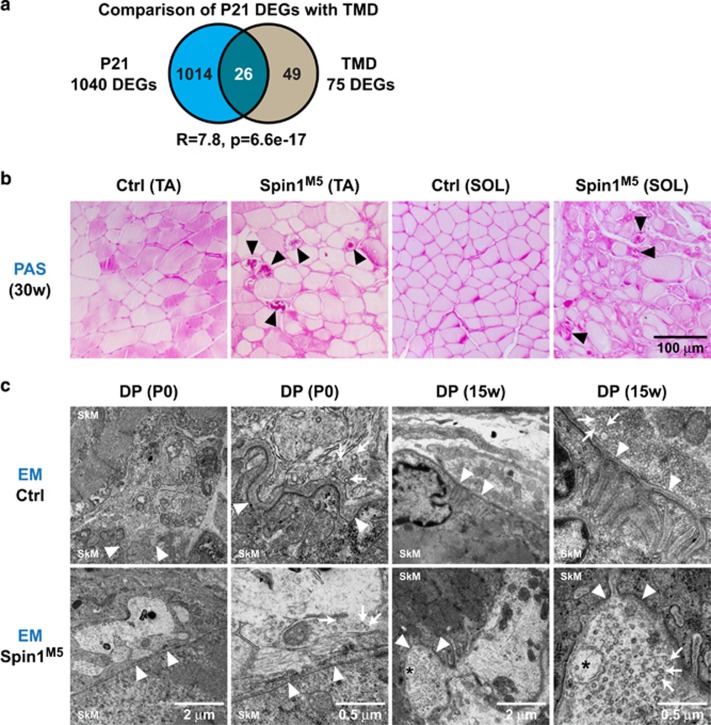
Differential gene expression in surviving Spin1^M5^ mice correlates with SkM disease patterns. (**a**) Intersection of DEGs observed for the TA of Spin1^M5^ mice at P21 (blue) and SkM of *mdm* mice^46^ (gray) representing a model for tibial muscular dystrophy (TMD) (R: enrichment factor; p: *P*-value calculated for the intersection). (**b**) Periodic acid–Schiff (PAS) staining for glycogen accumulation in TA and soleus muscle (SOL) of Spin1^M5^ and control mice at 30 weeks of age. Fibers showing abnormal glycogen accumulation are marked with black triangles. (**c**) Electron microscopy (EM) images of neuromuscular junctions in the diaphragm (DP) of newborn and 15-week-old Spin1^M5^ and control mice. Membranes at the synaptic cleft are marked with white triangles, synaptic vesicles with arrows, and vacuoles observed at neuromuscular junctions of Spin1^M5^ mice with asterisks

In the final set of experiments, we aimed to correlate differential gene expression in surviving Spin1^M5^ mice with SkM disease patterns. Since several P21 DEGs encode titin-associated proteins^21, 22^ (Figure 7d, network 'Ttn-associated'), we hypothesized similarity with diseases caused by *Ttn* mutations such as tibial muscular dystrophy (TMD).^44, 45^ Muscular dystrophy with myositis (*mdm*) mice (serving as a model of TMD) express a titin mutant lacking the N2A region, which binds Ankrd1, Ankrd2, and Ankrd23. Accordingly, defective Ankrd1 signaling has been implicated in TMD in *mdm* mice.^46^ Intersection of the P21 DEGs with 75 DEGs previously reported in *mdm* mice^46^ revealed a significant overlap of 26 genes (Figure 8a). Given that Ankrd1 and Ankrd2 are already deregulated in Spin1^M5^ fetuses at E16.5 (Figures 3c and 4c), aberrant expression of titin-associated proteins may account for SkM defects in Spin1^M5^ mice.

Next, we observed downregulation of genes involved in glycogen metabolism including *Pygm*, *Pfkm*, *Phka1*, *Phkg1*, *Prkaa2*, and *Prkag3* (Figure 7d, networks 'glucose & glycogen' metabolism and 'hypertrophy & atrophy pathways'). Mutations of these genes are known to occur in glycogen storage diseases (glycogenoses).^28^ Periodic acid–Schiff (PAS) staining revealed abnormal glycogen deposits in TA and soleus fibers of Spin1^M5^ mice (Figure 8b, black triangles). Although these deposits are limited to individual fibers, which differs from typical glycogenoses, our observation suggests that defective glycogen metabolism contributes to SkM disease in adult Spin1^M5^ mice.

Finally, we noted strong deregulation of acetylcholine receptor subunits (*Chrna1*, *Chrnd*, *Chrne*, *Chrng*) and several genes involved in excitation-contraction coupling (Figure 7d, networks 'NMJ' and 'ECC') hinting at defective neuromuscular junctions and/or excitation-contraction coupling. Therefore, we analyzed neuromuscular junctions in the diaphragm of newborn and adult Spin1^M5^ mice by electron microscopy (Figure 8c). At both time points, the analysis revealed defects of the synaptic membrane (white triangles) and an abnormal appearance as well as a reduced number of synaptic vesicles (arrows) in Spin1^M5^ mice (Figure 8c). Furthermore, we observed the presence of vacuoles at nerve terminals of adult Spin1^M5^ mice (Figure 8c, right columns (asterisks)). These data provide evidence for neuromuscular junction damage and abnormal excitation-contraction coupling in Spin1^M5^ mice.

## Discussion

In this study, we ablated the H3K4me3 reader Spin1 in myoblast precursors resulting in abnormal fetal myogenesis, early postnatal death, or severe SkM defects in few surviving Spin1^M5^ mice. Our analyses suggest that numerous E16.5 DEGs are direct target genes of Spin1 and Myod1. Furthermore, aberrant expression of Myf5, Myog, Msc, and Hes6 in Spin1^M5^ fetuses may indirectly affect Myod1-dependent gene regulation. Msc, for example, can repress transcription and antagonize the action of Myod1 in undifferentiated myoblasts by heterodimerization with E-proteins.^40^ Hes6 was reported to inhibit expression of Msc thereby enhancing myoblast differentiation.^43^ Therefore, deregulated bHLH transcription factor networks appear to determine, at least in part, aberrant gene expression in Spin1^M5^ fetuses.

The preferential degeneration of soleus, TA, and diaphragm in adult Spin1^M5^ mice raised the question whether this is caused by fiber type- or muscle type-selective mechanisms. Currently, we favor the latter hypothesis. While degeneration of type I and type II fibers might account for soleus defects, it would not convincingly explain the severe TA damage since this muscle contains only about 1 and 10% of these fibers types, respectively.^47^ Furthermore, at E15.5 or E16.5, we did not identify DEGs involved in the regulation of fiber type identity or plasticity (e.g., *Six1*, *Six4*, *Eya1*, *Sox6*).^30^

One possible determinant of muscle type-selective degeneration in Spin1^M5^ mice could be the deregulation of titin-associated proteins such as Ankrd1, which has been linked with TMD in *mdm* mice.^46^ However, despite some similarity with TMD, the SkM phenotype of Spin1^M5^ mice is apparently more complex. We exemplarily correlated the P21 DEGs with abnormal glycogen accumulation in individual TA and soleus fibers as well as neuromuscular junction defects in Spin1^M5^ mice. Due to the high number of DEGs observed in the TA of Spin1^M5^ mice at P21, these examples only provide an initial characterization of deregulated SkM networks.

Compared with transcription factors, signaling molecules, SkM structural proteins, or metabolic enzymes, the roles of epigenetic regulators in SkM physiology and disease have only more recently become a focus of research. However, while epigenetic writers, erasers, and non-coding RNAs have received considerable attention,^48, 49^ little is known about potential functions of histone code readers in SkM. Few readers (Brd4, Ing2, Sfmbt1, Dpf3) have been implicated in myogenesis using the C2C12 cell culture model^50, 51, 52, 53^ or in zebrafish.^54^ However, in these cases either knockout mouse models have not been reported, or in mice no apparent SkM phenotype was observed.^55, 56, 57^ Thus, to the best of our knowledge, Spin1 is the first histone code reader, for which ablation in SkM has fatal consequences in mice. Together, our histological and transcriptome analyses provide insight into severe SkM defects in Spin1^M5^ fetuses and surviving adult mice, hinting at Spin1 as a potential player in human SkM disease.

## Materials and methods

### Mouse studies

All mice were housed in the pathogen-free barrier facility of the University Medical Center Freiburg in accordance with institutional guidelines and approved by the regional board. Mice were maintained under temperature- and humidity-controlled conditions with a 12-h light/dark cycle, free access to water, and a standard rodent chow (3807, Provimi Kliba). Animals were killed by cervical dislocation and tissues immediately processed for further analyses.

### Generation and validation of Spin1^R26^ and Spin1^M5^ mice

The targeting strategy for generation of a conditional *Spin1* allele is outlined in Supplementary Figure 1a. Details are available upon request. The targeting construct was electroporated into C57BL/6 N Tac embryonic stem (ES) cells (Taconic), and neomycin-and puromycin-resistant clones were expanded. Selected ES cells were injected into blastocysts of C57BL/6 N mice. Resulting mice were bred to *Rosa26-Flp* mice to remove NeoR and PuroR selection markers. Offspring harbored a conditional *Spin1* allele, in which exon 4 of *Spin1* was flanked by loxP sites. C57/Bl6N mice homozygous for the conditional allele (*Spin1*^p/p^) were crossed with the *Rosa26-Cre* deleter strain^32^ to generate ubiquitous knockout (Spin1^R26^) mice or with the *Myf5-Cre* deleter strain^31^ to selectively ablate *Spin1* in myoblast and brown adipocyte precursors (Spin1^M5^ mice). Mice were maintained on the C57/Bl6N background. In phenotypic analyses of Spin1^M5^ mice, *Spin1*^+/p^ or *Spin1*^p/p^ littermates served as controls. Mice were genotyped by PCR amplification using GoTaq G2 Flexi DNA polymerase (Promega) of genomic DNA extracted from tail biopsies with the NucleoSpin 96 tissue kit (Macherey Nagel). The following genotyping primers were used: forward 5-ATAGGCTCTCTGGCATGGTG-3 / reverse 5-ACAGCGTGACACATCAAAGC-3 detecting the wild-type (177 bp) and the conditional (337 bp) allele and forward 5′-GGAGGAAGACACCTAATAGCACC-3′ /reverse 5′-AAGGCAAAACGGAGACAGC-3′ detecting the deleted allele (395 bp).

Efficient Spin1 depletion in myogenic cells and remaining Spin1 expression in non-myogenic cells of Spin1^M5^ mice was validated as follows. Spin1^M5^ and control mice were crossed with mice harboring a green fluorescent protein/nuclear lacZ (GNZ) reporter.^58^ In mice harboring the reporter, GNZ expression is Cre dependent. GNZ-positive myogenic cells in Spin1^M5^ mice did not express Spin1, whereas Spin1-positive cells were GNZ-negative and therefore non-myogenic (Supplementary Figure 1f). Part of these Spin1-expressing, non-myogenic cells were Tcf4-positive fibroblasts^34^ (Supplementary Figure 1e). Reduction of *Spin1* mRNA in hind limb muscle of newborn Spin1^M5^ mice was confirmed by quantitative RT-PCR (Supplementary Figure 1g).

### Quantitative RT-PCR and RNA sequencing

SkM tissue was homogenized in TRIzol reagent (Life Technologies, Darmstadt, Germany) using a Minilys personal homogenizer (Bertin, Montigny, France) and 0.5 or 2.0 ml CK14 lysing kits (Precellys, Montigny, France). RNA was isolated using a standard phenol/chloroform extraction protocol. cDNA was prepared by reverse transcription of total RNA using SuperScript II (Life Technologies) and oligo(dT) primer according to the supplier’s protocol. Quantitative RT-PCR was performed with a Lightcycler 480 II (Roche, Mannheim, Germany) using Absolute SYBR green ROX Mix (Thermo Scientific, Scherte, Germany) and the following primers: *Spin1* (exon 4, forward) 5′-CAGGTGCCTGTGAATCCTTC-3′, *Spin1* (exon 5, reverse) 5′-ACATGTGCTCCACTGCTTTG-3′, *Tbp* (forward) 5′-CCCCTTGTACCCTTCACCAAT-3′, *Tbp* (reverse) GAAGCTGCGGTACAATTCCAG-3′, *Hprt* (forward) 5′-GTTAAGCAGTACAGCCCCAAA-3′, *Hprt* (reverse) 5′-AGGGCATATCCAACAACAAACTT-3′, *Polr2a* (forward) 5′-CACCCCAGCTTCTCCCAAAT-3′, and *Polr2a* (reverse) 5′-AGTATGTCGGGGAGGTTGGA-3′. Data were analyzed using the 2(-Delta Delta C(T)) method.^59^

RNA-seq analyses for E15.5 and E16.5 were performed with SkM dissected from front and hind limb of three Spin1^M5^ and four control fetuses. For RNA-seq analysis of mice at three weeks of age (P21), TA isolated from four Spin1^M5^ and four control mice was used. RNA was isolated as described above, except that minced tissue in TRIzol was further homogenized with QIA shredder spin columns (Qiagen, Hilden, Germany). RNA quality was determined using the RNA 6000 Nano Kit (Agilent, Waldbronn, Germany) on an Agilent 2100 Bioanalyzer. RNA with a RIN above 8.0 was sequenced at the DKFZ core facility (Heidelberg, Germany) or the Deep Sequencing Unit (Max-Planck-Institute of Immunology and Epigenetics, Freiburg) using the standard Illumina protocol. Paired-end reads were mapped to Ensemble annotation NCBIm38/mm10 with TopHat2^60^ using default parameters. The aligned reads were counted with HOMER software^61^ and DEGs calculated with edgeR.^62^ Overrepresentation analyses for the identified DEGs (*P*≤1e−3 (E15.5 and E16.5), *P*≤1e−5 (P21); fold change ≥1.5; ≥50 reads in all Spin1^M5^ or all control samples] were performed using WebGestalt^36^ with 'genome' as reference set.

Of note, none of the RNA-seq analyses identified *Spin1* as differentially expressed gene. Examination of the normalized reads at exons 1 to 6 of the *Spin1* gene explained this observation (Supplementary Table 1e). Reads were significantly decreased at exon 4 (which is excised in a *Myf5-Cre*-dependent manner in myogenic cells), but not at other exons in Spin1^M5^ relative to control mice. Thus, in myogenic cells of Spin1^M5^ mice a truncated transcript (lacking exon 4) is expressed, which results in translation of a truncated Spin1 peptide (containing 56 N-terminal amino acids lacking any known functional domain) due to the presence of a premature STOP codon in exon 5. Normal *Spin1* transcript (containing exon 4) present in SkM of Spin1^M5^ mice is most likely produced by non-myogenic cells (Supplementary Figures 1e and f). Since mapped reads at all exons contribute to the overall count, differential expression of *Spin1* is not detected in RNA-seq analyses.

### Antibodies

The generation and validation of anti-SPIN1 antibodies 5865 and 5867 was described previously.^6^ Antibodies SPIN1(5867) and H3K4me3 (Diagenode, Oxford, UK, C15410003) were used for ChIP. For immunofluorescence staining the following primary antibodies were used: SPIN1(5865) 1 *μ*g/ml; Pax7 (PAX7, DSHB, batch 7/2/15) 2 *μ*g/ml; Tcf4 (6H5-3, Millipore, Darmstadt, Germany, 05-511, batch 2155406) 10 *μ*g/ml; Ankrd1 (Proteintech Group, Manchester, UK, 11427-1-AP, batch 1951) 1:100; Ankrd2 (Proteintech Group, 11821-1-AP, batch 7649) 1:100; dystrophin (Abcam, Cambridge, UK, ab15277, batch GR226781-6) 1:500; MHC-I (NOQ7.5.4D, Sigma, Munich, Germany, M8421, batch 035M4792V) 1:2000; MHC-IIa (SC-71, DSHB, batch 4/7/16) 1:10; MHC-IIx (6H1, DSHB, batch 3/3/16) 1:6; MHC-IIb (BF-F3, DSHB, batch 5/12/16) 1:20; MCH, skeletal, fast (MY-32, Sigma, M4276, batch 083M4790V) 1:1000; GNZ (anti-GFP, Abcam, ab13970, batch GR236651) 1:1000; normal rabbit IgG (Santa Cruz, Heidelberg, Germany, sc-2027). The following secondary antibodies were used: Alexa Fluor 488 (goat anti-mouse IgM, Molecular Probes, Karlsruhe, Germany, A21042, batch 1387726) 1:600; Alexa Fluor 488 (goat anti-mouse IgG, Molecular Probes, A11029, batch 1306597) 1:600; Alexa Fluor 546 (goat anti-mouse IgG, Molecular Probes, 11030, batch 517979) 1:600; Alexa Fluor 594 (goat anti-chicken IgG, Molecular Probes, A11042, batch 762712) 1:600; donkey anti-rabbit IgG-HRP (Santa Cruz, sc-2313) 1:500.

### Immunofluorescence staining

SkM tissues were either fixed in 4% paraformaldehyde/PBS or flash-frozen in 2-methylbutane (Sigma). Paraffin sections (5 *μ*m) were deparaffinized, heated in antigen retrieval solution (20 mM Tris (pH9.0)) for 20 min in a pressure cooker, and blocked for 1 h at room temperature in 3% skim milk powder/PBS containing 0.1% Tween (PBST). Cryosections (10 *μ*m) were blocked with 5% fetal calf serum/PBST. Sections were incubated overnight with primary antibody at 4 °C, washed with PBST, incubated with secondary antibody for 1 h at room temperature, and then washed with PBST. For Spin1 staining, signal amplification using the TSA fluorescein system (Perkin Elmer, NEL701A001KT) was applied according to the manufacturer’s instructions. Briefly, tissue sections were blocked in 0.5% TNB blocking buffer, labeled with primary and secondary antibody, and then incubated with TSA reagent for 15 minutes at room temperature followed by two washing steps with PBST. Finally, nuclei were stained with DAPI (1 *μ*g/ml) followed by two washing steps with PBST, and sections were mounted using Fluoromount-G (SouthernBiotech). Images were recorded with a confocal microscope (Leica TCS SP2 AOBS). Counting was done with ImageJ^63^ followed by visual validation of the results. Pax7-positive nuclei or nuclei of myofibers (expressing Spin1 or being Spin1-negative) were quantified using confocal images with dimensions of 187.5 *μ*m × 187.5 *μ*m or 375 *μ*m × 375 *μ*m, respectively.

### Isolation of primary myoblasts and ChIP sequencing

Primary myoblasts were isolated from 10- day-old C57BL/6 N mice using an established preplating protocol.^64^ Briefly, limb muscles were collected from front and hind leg, minced, digested for 1 h in 0.2% collagenase type I (Sigma, C0130), and filtered through 100 μm cell strainers (Falcon, 352360). Cells were collected by centrifugation, resuspended in DMEM medium (Gibco, Schwerte, Germany, 11995-065) supplemented with 10% FCS, 10% horse serum, and 1.25% chicken embryo extract (Seralab, CE-650-J), and cultivated for 1 h on 6 cm tissue culture dishes to allow for attachment of fibroblasts. Supernatants were transferred to 6 cm dishes, again incubated for one hour, and then transferred to 10 cm tissue culture dishes coated with collagen (Gibco, A10483-01). Myoblasts were cultivated for 3 to 4 days until a confluency of about 70% was reached. Cells were fixed with 1% formaldehyde for 5 minutes, quenched in glycine (1.25 M), washed with PBS buffer, collected, and snap-frozen in liquid nitrogen.

Chromatin was prepared using the NEXSON procedure.^65^ Briefly, nuclei were extracted by sonication with a Covaris E220 sonicator (75 W peak power, 2% duty factor, 200 cycles/burst, 60 s). Nuclei were pelleted, resuspended in 1 ml of shearing buffer, and sonicated for 12 min (140 W peak power, 5% duty factor, 200 cycles/burst). Chromatin was diluted 1:2 in buffer H (Diagenode auto histone ChIP-seq kit (C01010022)) before ChIP. For ChIP, one tenth of the chromatin was incubated with 1 μg of H3K4me3 antibody (Diagenode, C15410003). The remaining chromatin (from ~1.2 million cells) was incubated with 5 μg of SPIN1(5867) antibody. ChIP was performed using the automated platform SX-8G IP-Star (Diagenode) and the program 'ChIP indirect method'. Chromatin was incubated with antibody for 10 h followed by 3 h of incubation with protein A-conjugated beads. 1% of the original chromatin was used as input. After elution from beads, ChIP and input samples were reverse crosslinked, and DNA purified with MinElute columns (Qiagen, 28004). Libraries were prepared using the NEBNext Ultra DNA library preparation kit (NEB, E7370S) according to the manufacturer’s instruction and without size selection. Adapter-ligated fragments were amplified with 12 PCR cycles and sequenced on the Illumina HiSeq 2500 platform by the Deep Sequencing Unit of the Max-Planck-Institute of Immunology and Epigenetics (Freiburg).

Paired-end reads were mapped to the mouse reference genome (mm10) using bowtie 2^66^ with default parameters. Data were further analyzed using the peak finding algorithm of MACS 1.4.2^67^ using input as control. All peaks with a false discovery rate >1% were excluded from further analyses. The uniquely mapped reads were used to generate genome-wide intensity profiles, which were visualized using the integrative genomics viewer (IGV).^68^ Peaks were annotated and overlaps between different peak files were calculated with HOMER.^61^ The genomic features (promoter, exon, intron, 5′ or 3′ untranslated region, and intergenic region) were defined and calculated using Refseq and HOMER. Myod1 chromatin association in C2C12 myoblasts was analyzed using a previously deposited GEO data set (GSE36024). Venn diagrams were generated with the help of Venny.^69^ Intensity profiles for Spin1 and H3K4me3 gene promoter occupancy were analyzed with SeqMINER.^70^

### GEO data availability

The RNA and DNA sequencing data discussed in this publication have been deposited in NCBI's Gene Expression Omnibus^71^ and are accessible through GEO Series accession number GSE92539.

### Electron microscopy

Muscle samples were fixed by immersion in 2.5% glutaraldehyde and 2.5% paraformaldehyde in cacodylate buffer (0.1 M, pH 7.4) and then washed in cacodylate buffer for further 30 min. The samples were post-fixed in 1% osmium tetroxide in 0.1 M cacodylate buffer for 1 h at 4 °C, and dehydrated in an ascending ethanol gradient (50, 70, 90, and 100%) and propylene oxide for 30 min each. Samples were embedded in Epon 812 substitute (Sigma-Aldrich). Semi-thin sections cut at 2 μm and ultra-thin sections cut at 70 nm were contrasted with uranyl acetate and lead citrate and examined at 70 kV with a Morgagni 268D electron microscope. Images were digitally captured by Mega View III camera (Soft Imaging System).

### Hematoxylin & eosin staining

Deparaffinized and rehydrated or flash-frozen tissue sections (5 or 10 *μ*m, respectively) were stained according to a standard protocol with hematoxylin (Gill No. 3, Sigma, GHS332) and eosin Y solution (Sigma, HT110332) and mounted using Roti-Histokitt (Roth).

### Gomori trichrome staining

Deparaffinized and rehydrated tissue sections (5 *μ*m) were stained using Bouin’s solution (Sigma, HT10132), hematoxylin-Weigert’s iron kit (Dianova, HWI-2), and trichrome stain (blue) solution (Dianova, TGB500) according to the supplier's instructions and mounted using Roti-Histokitt (Roth).

### NADH staining

Cryosections (10 *μ*m) were incubated with staining solution (0.2 M Tris (pH 7.4), 1.5 mM NADH (Roche, 10128015001), 1.5 mM nitroblue tetrazolium (Sigma, N-6876)), dehydrated in an ascending ethanol gradient, incubated twice with xylene, and mounted using Roti-Histokitt (Roth).

### Periodic acid–Schiff staining

Deparaffinized and rehydrated tissue sections (5 *μ*m) were treated with 0.5% periodic acid solution (Sigma, 3951), stained with Schiff’s reagent (Sigma, 3952016), dehydrated, and mounted using Roti-Histokitt (Roth).

### Statistics

Transcriptome and cistrome data were analyzed as described above. Statistical significance of gene set intersections was evaluated by a hypergeometric test using the program 'R' (http://www.R-project.org) [phyper (N12-1, N1, N-N1, N2, lower.tail=FALSE) with N1 (genes in set 1), N2 (genes in set 2), N12 (genes in intersection), and N (genome size)]. The enrichment factor (R) was calculated according to *R*=(N × N12)/(N1 × N2). Other data are presented as the mean value or percentage change +S.D. Comparisons between two data sets were made using the two-tailed Student's *t*-test for parametric data and the Wilcoxon signed-rank test for nonparametric data. A *P*-value of less than 0.05 was considered statistically significant. Statistical significance is indicated as follows: *P<0.05, **P<0.01, ***P<0.001.
