# Supplementary material for: The histone code reader Spin1 controls skeletal muscle development
Source: Cell Death Dis. 2017 Nov 23;8(11):e3173–. doi: 10.1038/cddis.2017.468 (PMC5775400; doi:10.1038/cddis.2017.468)

Supplementary Figure 1 (Greschik et al.)

a

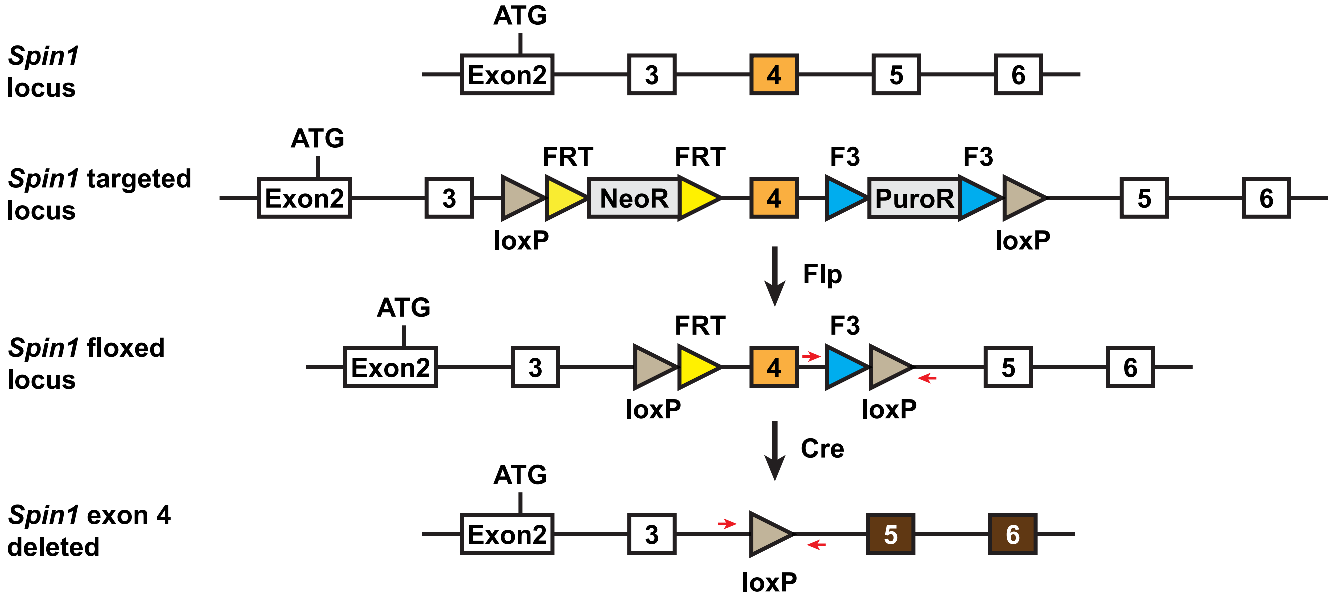

b

Spin1R26 mice

Genotypes	at birth	at weaning
Spin1+/+	25 (29.1 %)	99 (37.6 %)
Spin1+/d	43 (50.0 %)	164 (62.4 %)
Spin1d/d	18 (20.9 %)	0

c

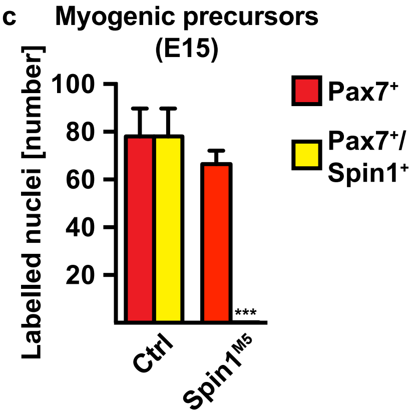

d

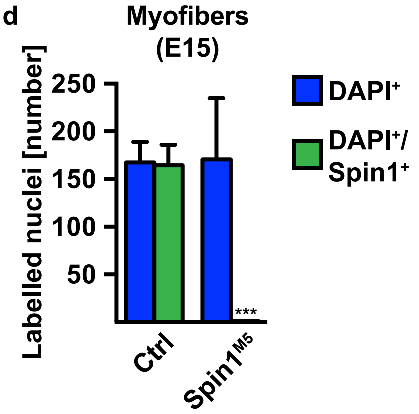

e

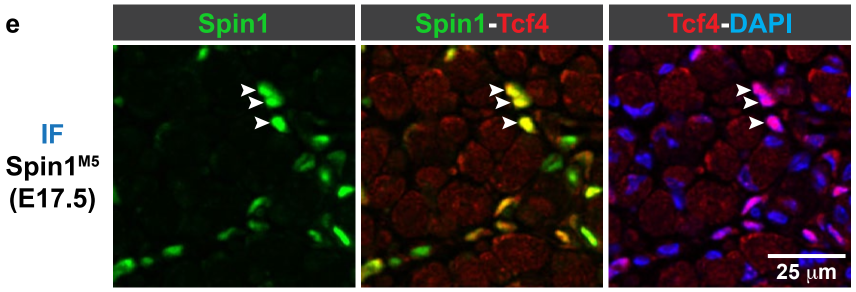

g

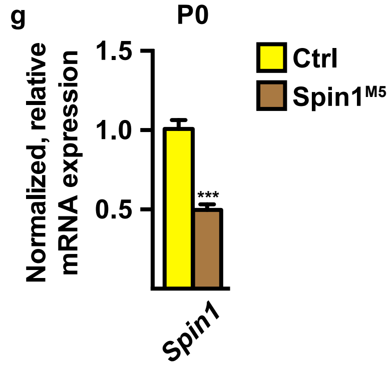

f

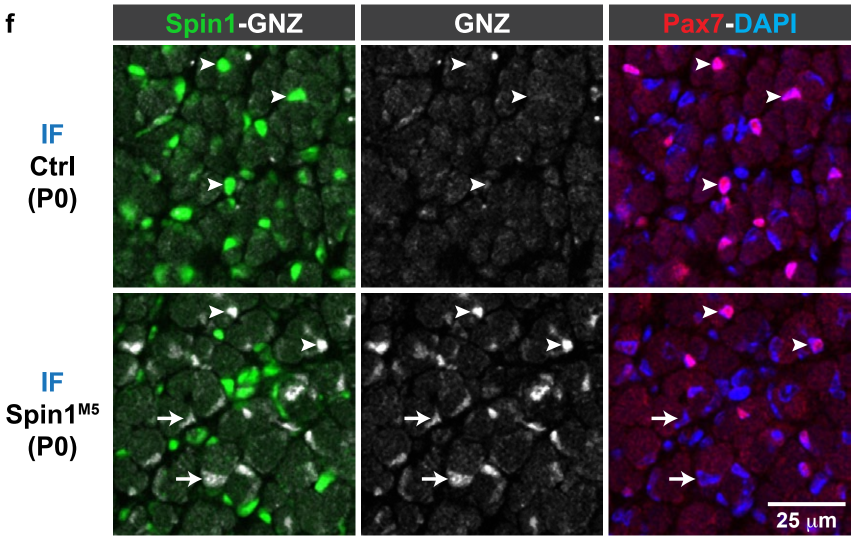

h

Spin1M5 mice

Genotypes	at birth	at weaning
Spin1+/p	54 (24.1 %)	76 (26.2 %)
Spin1+/p +/Cre	59 (26.4 %)	100 (34.5 %)
Spin1p/p	61 (27.2 %)	95 (32.7 %)
Spin1p/p +/Cre	50 (22.3 %)	19 (6.6 %)

Supplementary Figure 2 (Greschik et al.)

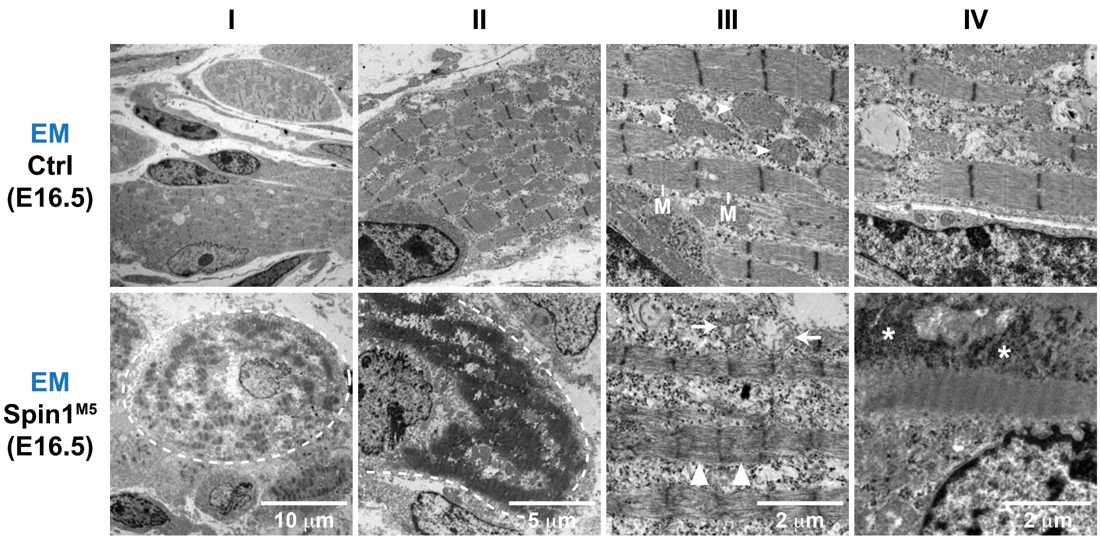

Supplementary Figure 3 (Greschik et al.)

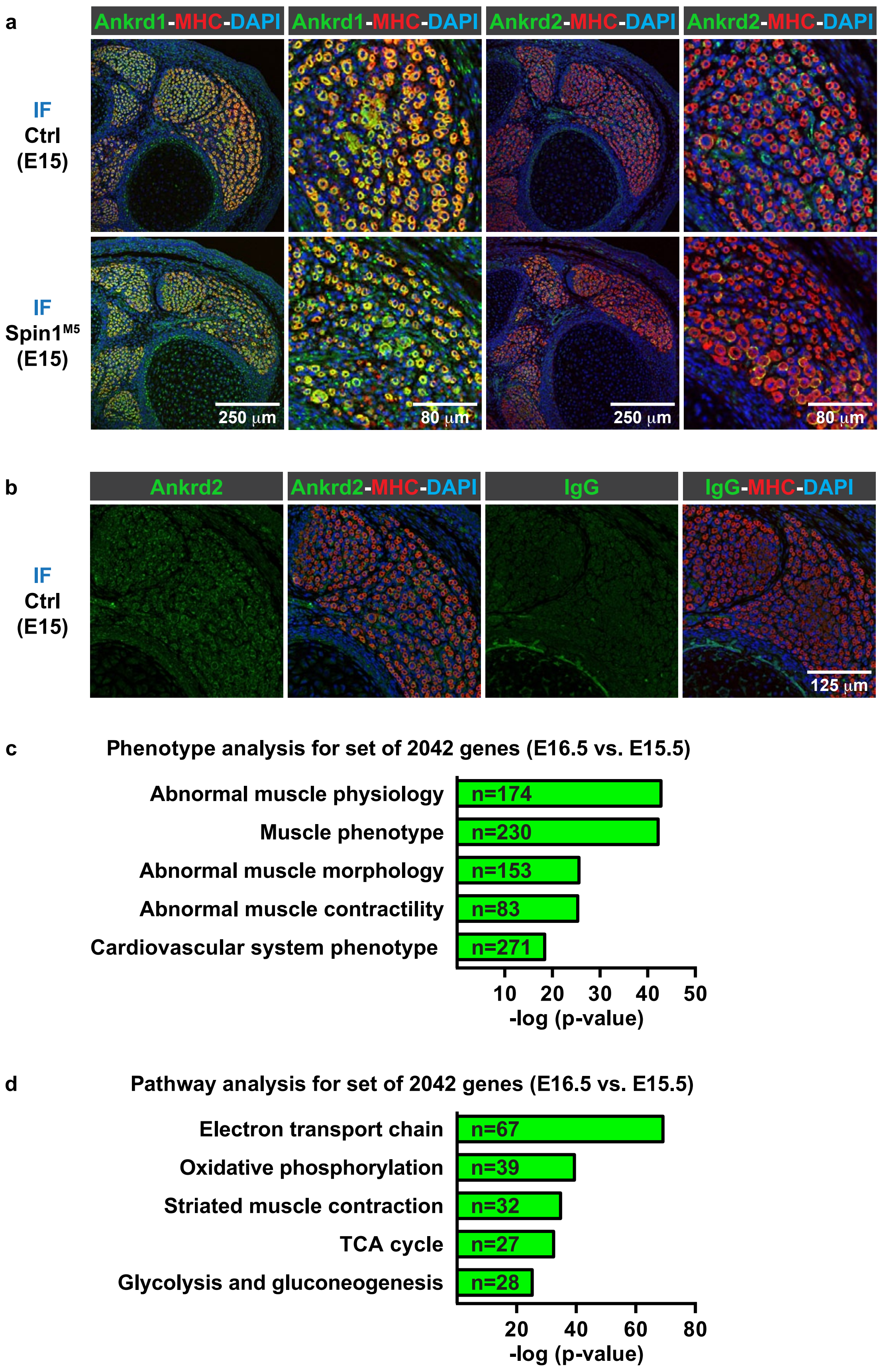

Supplementary Figure 4 (Greschik et al.)

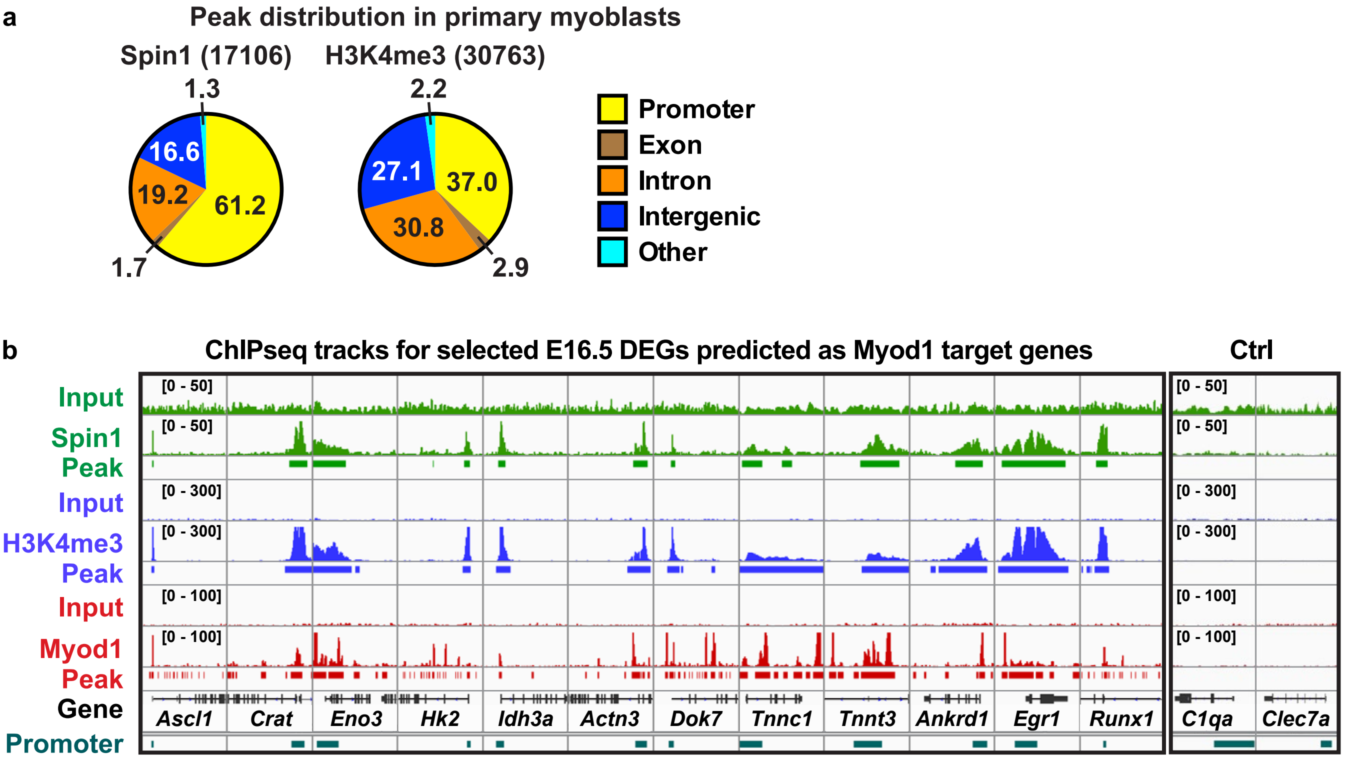

Supplementary Figure 5 (Greschik et al.)

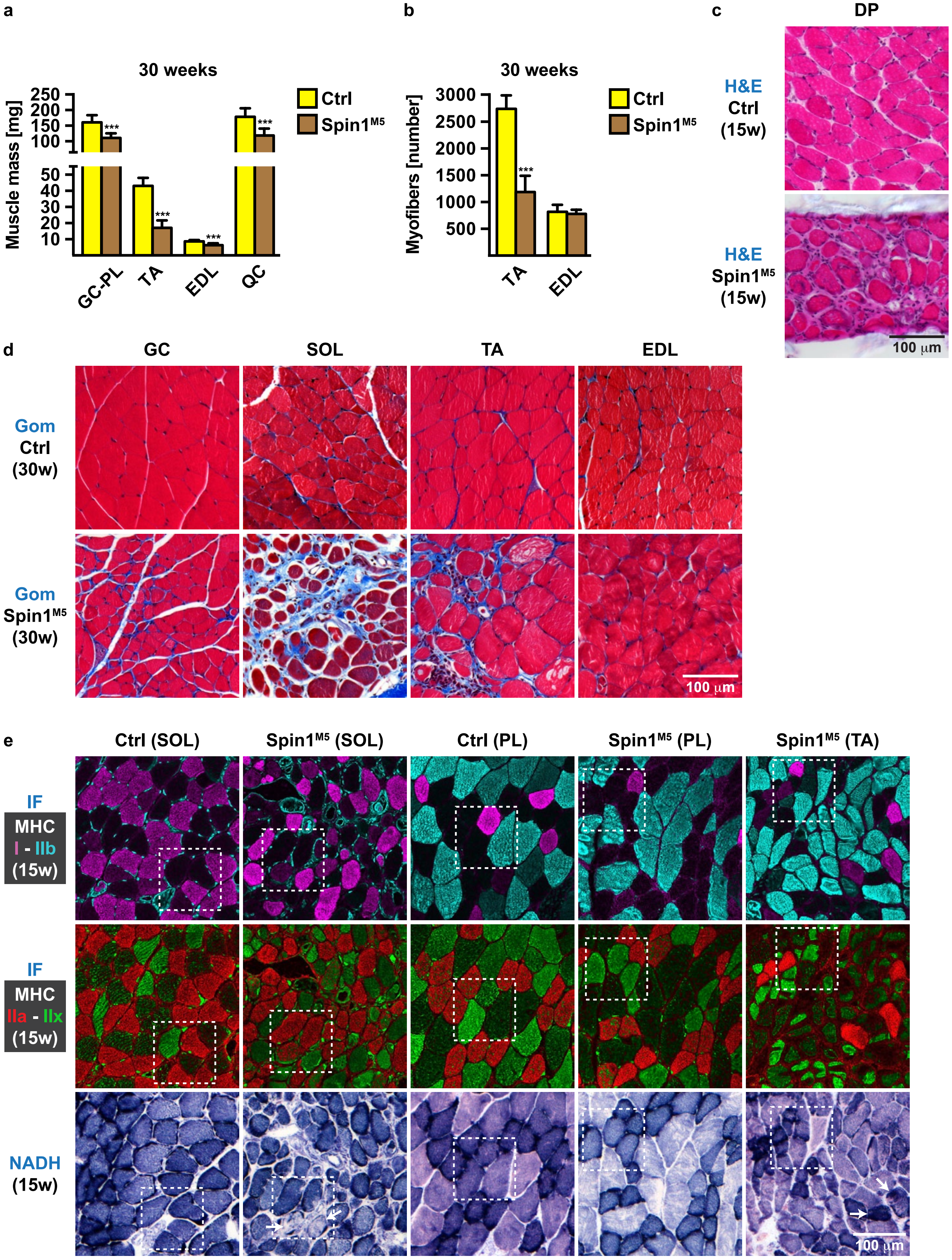

Supplement: Supplementary Figures [file cddis2017468x1.pdf]
