# Supplementary material for: The histone code reader Spin1 controls skeletal muscle development
Source: Cell Death Dis. 2017 Nov 23;8(11):e3173–. doi: 10.1038/cddis.2017.468 (PMC5775400; doi:10.1038/cddis.2017.468)
Supplement: Supplementary Tables 1 [file cddis2017468x2.pdf]

Supplementary Table 1a: Differentially expressed genes in SkM of Spin1^{M5} compared to control mice at E15.5.

Gene symbol	FC (Spin1^{M5} vs. Ctrl)	logFC (Spin1^{M5} vs. Ctrl)	FDR	p-value	Entrez gene	Protein name
Aifm3	0.49	-1.02	4.1E-01	3.5E-04	72168	apoptosis-inducing factor, mitochondrion-associated 3
Ankrd1	1.67	0.74	1.5E-01	5.8E-05	107765	ankyrin repeat domain 1 (cardiac muscle)
Ankrd2	2.17	1.12	4.1E-01	3.6E-04	56642	ankyrin repeat domain 2 (stretch responsive muscle)
Dbx1	0.51	-0.96	5.3E-02	1.5E-05	13172	developing brain homeobox 1
Gm7120	1.75	0.81	1.8E-03	8.1E-08	633640	predicted gene 7120
Hbb-b2	1.51	0.59	6.7E-01	9.3E-04	15130	hemoglobin, beta adult minor chain
Hbb-bt	1.51	0.59	6.7E-01	9.4E-04	101488143	hemoglobin, beta adult t chain
Il17b	0.67	-0.59	5.3E-02	1.6E-05	56069	interleukin 17B
Ly6a	1.79	0.84	2.3E-01	1.2E-04	110454	lymphocyte antigen 6 complex, locus A
Msc	0.62	-0.68	4.4E-02	8.7E-06	17681	musculin
Myf5	0.56	-0.82	3.9E-01	2.3E-04	17877	myogenic factor 5
Nos1	0.43	-1.22	2.2E-01	9.9E-05	18125	nitric oxide synthase 1, neuronal
Pmaip1	1.95	0.96	1.8E-03	1.2E-07	58801	phorbol-12-myristate-13-acetate-induced protein 1
Scn4b	2.04	1.03	4.5E-02	1.0E-05	399548	sodium channel, type IV, beta
Snai3	0.53	-0.92	1.7E-01	7.0E-05	30927	snail homolog 3
Snca	1.57	0.65	6.1E-01	6.2E-04	20617	synuclein, alpha
Spin1	see Supplementary Table 1e				20729	spindlin 1
Tpm3	0.49	-1.02	6.7E-01	9.4E-04	59069	tropomyosin 3, gamma

upregulated (red)
downregulated (blue)

Supplementary Table 1b: Differentially expressed genes in SkM of Spin1[M5] compared to control mice at E16.5.

Gene symbol	FC (Spin1[M5] vs. Ctrl)	logFC (Spin1[M5] vs. Ctrl)	FDR	p-value	Entrez gene	Protein name
1200014J11Rik	1.62	0.70	2.1E-04	4.9E-07	66874	RIKEN cDNA 1200014J11 gene
6430571L13Rik	0.40	-1.33	1.6E-03	5.7E-06	235599	RIKEN cDNA 6430571L13 gene
A630019I02Rik	0.51	-0.98	4.6E-04	1.3E-06	408254	uncharacterized LOC408254
A930016O22Rik	0.66	-0.60	8.0E-02	9.9E-04	100503495	RIKEN cDNA A930016O22 gene
Abca5	0.64	-0.64	2.0E-02	1.3E-04	217265	ATP-binding cassette, sub-family A (ABC1), member 5
Acacb	0.49	-1.04	6.5E-09	3.0E-12	100705	acetyl-Coenzyme A carboxylase beta
Ache	1.70	0.76	1.0E-02	5.2E-05	11423	acetylcholinesterase
Acot11	0.57	-0.81	4.9E-05	7.9E-08	329910	acyl-CoA thioesterase 11
Acsl1	0.48	-1.06	5.0E-03	2.0E-05	14081	acyl-CoA synthetase long-chain family member 1
Acss3	0.43	-1.21	5.0E-02	5.1E-04	380660	acyl-CoA synthetase short-chain family member 3
Actn3	0.65	-0.62	1.3E-02	7.3E-05	11474	actinin alpha 3
Adck3	0.65	-0.62	5.2E-02	5.4E-04	67426	aarF domain containing kinase 3
Adcyap1r1	0.59	-0.75	1.1E-02	5.6E-05	11517	adenylate cyclase activating polypeptide 1 receptor 1
Adhfe1	0.25	-2.00	4.1E-02	3.9E-04	76187	alcohol dehydrogenase, iron containing, 1
Adora1	1.74	0.80	4.3E-05	6.4E-08	11539	adenosine A1 receptor
Agbl1	0.32	-1.65	4.2E-03	1.6E-05	244071	ATP/GTP binding protein-like 1
Aldh4a1	0.55	-0.87	1.1E-06	9.4E-10	212647	aldehyde dehydrogenase 4 family, member A1
Aldh6a1	0.58	-0.79	3.3E-02	2.6E-04	104776	aldehyde dehydrogenase family 6, subfamily A1
Amigo1	0.66	-0.59	6.1E-03	2.7E-05	229715	adhesion molecule with Ig like domain 1
Ampd1	0.65	-0.63	1.7E-03	6.1E-06	229665	adenosine monophosphate deaminase 1
Ankrd1	2.95	1.56	4.1E-06	4.0E-09	107765	ankyrin repeat domain 1 (cardiac muscle)
Ankrd2	4.51	2.17	9.9E-13	6.5E-17	56642	ankyrin repeat domain 2 (stretch responsive muscle)
Ankrd23	1.94	0.95	1.1E-06	9.2E-10	78321	ankyrin repeat domain 23
Ankrd9	0.58	-0.80	4.3E-03	1.7E-05	74251	ankyrin repeat domain 9
Apoe	1.62	0.69	2.4E-02	1.7E-04	11816	apolipoprotein E
Aqp4	0.05	-4.20	6.2E-11	1.4E-14	11829	aquaporin 4
Arhgap36	0.59	-0.75	3.3E-05	4.3E-08	75404	Rho GTPase activating protein 36
Arpp21	1.62	0.69	2.8E-02	2.1E-04	74100	cyclic AMP-regulated phosphoprotein, 21
Art3	0.63	-0.68	1.5E-02	9.0E-05	109979	ADP-ribosyltransferase 3
Atcay	0.57	-0.82	6.5E-02	7.5E-04	16467	ataxia, cerebellar, Cayman type homolog
Atp1b2	0.52	-0.94	3.1E-08	2.2E-11	11932	ATPase, Na+/K+ transporting, beta 2 polypeptide
Atp2a1	0.62	-0.68	6.0E-04	1.7E-06	11937	ATPase, Ca++ transporting, cardiac muscle, fast twitch 1
Atp2b3	0.46	-1.13	3.7E-04	9.5E-07	320707	ATPase, Ca++ transporting, plasma membrane 3
Baiap2l1	1.78	0.83	2.1E-03	7.7E-06	66898	BAI1-associated protein 2-like 1
Bche	0.64	-0.64	8.3E-03	3.9E-05	12038	butyrylcholinesterase
C1qa	1.77	0.82	1.7E-03	6.0E-06	12259	complement component 1, q subcomponent, alpha polypeptide
C1qb	1.74	0.80	1.1E-04	2.0E-07	12260	complement component 1, q subcomponent, beta polypeptide
C1qc	1.69	0.75	2.6E-04	6.1E-07	12262	complement component 1, q subcomponent, C chain
C3	0.17	-2.52	1.2E-02	6.7E-05	12266	complement component 3
C3ar1	1.83	0.87	3.5E-02	2.9E-04	12267	complement component 3a receptor 1
Car3	0.44	-1.18	4.8E-03	1.9E-05	12350	carbonic anhydrase 3
Casq1	0.61	-0.71	1.3E-04	2.4E-07	12372	calsequestrin 1
Ccdc23	1.68	0.75	2.1E-04	4.8E-07	69216	coiled-coil domain containing 23
Ccdc69	0.56	-0.85	5.7E-02	6.0E-04	52570	coiled-coil domain containing 69
Cck	2.15	1.11	1.2E-02	6.3E-05	12424	cholecystokinin
Ccne1	1.58	0.66	3.3E-03	1.2E-05	12447	cyclin E1
Cd68	1.60	0.68	3.0E-02	2.4E-04	12514	CD68 antigen
Cd82	1.54	0.62	5.0E-03	2.0E-05	12521	CD82 antigen
Chrna9	0.22	-2.17	2.3E-08	1.4E-11	231252	cholinergic receptor, nicotinic, alpha polypeptide 9
Cilp	1.95	0.96	4.1E-04	1.1E-06	214425	cartilage intermediate layer protein, nucleotide pyrophosphohydrolase
Ckm	0.65	-0.62	6.8E-02	7.9E-04	12715	creatine kinase, muscle
Ckmt2	0.60	-0.73	2.1E-04	4.5E-07	76722	creatine kinase, mitochondrial 2
Clcn1	0.54	-0.88	6.1E-02	6.8E-04	12723	chloride channel 1
Clec4n	2.26	1.18	2.8E-02	2.1E-04	56620	C-type lectin domain family 4, member n
Clec7a	3.25	1.70	6.8E-04	2.1E-06	56644	C-type lectin domain family 7, member a
Clic5	0.49	-1.02	1.6E-02	1.0E-04	224796	chloride intracellular channel 5
Cmbl	0.37	-1.45	5.0E-09	2.1E-12	69574	carboxymethylenebutenolidase-like
Cmya5	0.59	-0.76	6.1E-05	1.1E-07	76469	cardiomyopathy associated 5
Col19a1	1.61	0.68	2.4E-02	1.7E-04	12823	collagen, type XIX, alpha 1
Cox8b	0.65	-0.63	7.4E-02	8.8E-04	12869	cytochrome c oxidase, subunit VIIIb
Cpt1b	0.52	-0.94	2.7E-08	1.7E-11	12895	carnitine palmitoyltransferase 1b, muscle
Crat	0.60	-0.75	1.6E-05	2.0E-08	12908	carnitine acetyltransferase
Ctss	2.03	1.02	1.3E-04	2.4E-07	13040	cathepsin S
Cx3cr1	1.68	0.74	2.2E-02	1.5E-04	13051	chemokine (C-X3-C) receptor 1
Dbndd1	2.53	1.34	2.2E-04	5.1E-07	72185	dysbindin (dystrobrevin binding protein 1) domain containing 1
Ddit4l	0.63	-0.67	4.0E-02	3.7E-04	73284	DNA-damage-inducible transcript 4-like
Dnaic1	0.44	-1.20	1.5E-02	8.8E-05	68922	dynein, axonemal, intermediate chain 1
Dok7	1.86	0.90	1.6E-03	5.4E-06	231134	docking protein 7
Eda2r	1.60	0.68	5.7E-02	5.9E-04	245527	ectodysplasin A2 receptor
Eef1a2	0.39	-1.38	2.5E-12	3.1E-16	13628	eukaryotic translation elongation factor 1 alpha 2
Egr1	1.55	0.63	4.8E-03	1.9E-05	13653	early growth response 1
Eno3	0.65	-0.63	5.0E-03	2.1E-05	13808	enolase 3, beta muscle
Ephx1	0.63	-0.66	1.7E-02	1.0E-04	13849	epoxide hydrolase 1, microsomal
Ermap	2.63	1.39	9.7E-04	3.0E-06	27028	erythroblast membrane-associated protein
Extl1	0.42	-1.26	2.3E-02	1.5E-04	56219	exostoses (multiple)-like 1
Fam131a	0.58	-0.78	3.5E-04	8.9E-07	78408	family with sequence similarity 131, member A
Fbp2	0.62	-0.69	3.3E-02	2.7E-04	14120	fructose bisphosphatase 2
Fbxl22	0.57	-0.82	4.6E-05	7.1E-08	74165	F-box and leucine-rich repeat protein 22
Fbxo17	1.52	0.60	2.6E-02	1.9E-04	50760	F-box protein 17
Fcrls	1.64	0.71	2.6E-02	1.9E-04	80891	Fc receptor-like S, scavenger receptor
Frmpd1	1.61	0.69	2.8E-02	2.1E-04	666060	FERM and PDZ domain containing 1
Frzb	0.59	-0.75	2.5E-03	9.1E-06	20378	frizzled-related protein
Fsd2	0.61	-0.72	4.7E-05	7.4E-08	244091	fibronectin type III and SPRY domain containing 2
Glb1l2	0.29	-1.78	9.7E-03	4.8E-05	244757	galactosidase, beta 1-like 2
Glis1	0.49	-1.04	4.0E-02	3.6E-04	230587	GLIS family zinc finger 1
Gm44502	0.59	-0.75	4.2E-05	6.0E-08	100134861	predicted readthrough transcript (NMD candidate) 44502
Gm5111	0.41	-1.28	1.1E-02	6.0E-05	330305	predicted gene 5111
Gm7325	2.18	1.13	5.4E-06	5.6E-09	653016	predicted gene 7325
Gpnmb	3.01	1.59	1.3E-05	1.6E-08	93695	glycoprotein (transmembrane) nmb
Grip2	0.64	-0.64	2.9E-02	2.2E-04	243547	glutamate receptor interacting protein 2
Hes6	1.55	0.63	2.3E-02	1.5E-04	55927	hairy and enhancer of split 6
Hhatl	0.67	-0.59	3.9E-02	3.5E-04	74770	hedgehog acyltransferase-like
Hk2	0.58	-0.77	3.7E-05	4.9E-08	15277	hexokinase 2
Hrasls	0.36	-1.49	6.2E-04	1.9E-06	27281	HRAS-like suppressor
Hspb6	0.54	-0.88	9.1E-07	7.5E-10	243912	heat shock protein, alpha-crystallin-related, B6
Idh3a	0.66	-0.60	9.3E-03	4.5E-05	67834	isocitrate dehydrogenase 3 (NAD+) alpha
Igf2os	1.71	0.78	8.9E-06	1.0E-08	111975	insulin-like growth factor 2, opposite strand
Isyna1	1.94	0.95	9.6E-08	7.3E-11	71780	myo-inositol 1-phosphate synthase A1
Kbtbd13	0.54	-0.89	5.0E-02	5.1E-04	74492	kelch repeat and BTB (POZ) domain containing 13
Kcna7	0.48	-1.06	3.5E-02	3.0E-04	16495	potassium voltage-gated channel, shaker-related subfamily, member 7
Kcnj12	0.59	-0.76	3.9E-02	3.5E-04	16515	potassium inwardly-rectifying channel, subfamily J, member 12

Kcnk13	1.99	0.99	1.0E-02	5.0E-05	217826	potassium channel, subfamily K, member 13
Kl	0.26	-1.96	1.2E-06	1.1E-09	16591	klotho
Krt8	0.48	-1.05	3.2E-02	2.5E-04	16691	keratin 8
Lmnb2	1.52	0.60	7.7E-03	3.6E-05	16907	lamin B2
Lpin1	0.59	-0.75	2.2E-02	1.4E-04	14245	lipin 1
Ly86	2.42	1.28	1.3E-02	7.2E-05	17084	lymphocyte antigen 86
Lyz2	1.75	0.81	3.3E-02	2.6E-04	17105	lysozyme 2
Lzts3	1.68	0.75	5.8E-02	6.1E-04	241638	leucine zipper, putative tumor suppressor family member 3
Map3k7cl	1.80	0.84	5.9E-05	1.0E-07	224419	Map3k7 C-terminal like
Mc4r	2.33	1.22	4.4E-02	4.4E-04	17202	melanocortin 4 receptor
Mettl21c	0.43	-1.21	4.4E-02	4.3E-04	433294	methyltransferase like 21C
Mettl21e	0.37	-1.42	1.3E-08	6.2E-12	403183	methyltransferase like 21E
Mlf1	0.57	-0.80	1.2E-03	3.7E-06	17349	myeloid leukemia factor 1
Mpeg1	2.40	1.26	9.8E-10	3.5E-13	17476	macrophage expressed gene 1
Msc	0.54	-0.89	2.2E-02	1.4E-04	17681	musculin
Mtus2	0.56	-0.84	6.5E-02	7.4E-04	77521	microtubule associated tumor suppressor candidate 2
Myadml2	0.66	-0.59	6.0E-02	6.6E-04	68515	myeloid-associated differentiation marker-like 2
Mybpc2	0.30	-1.74	2.5E-12	3.2E-16	233199	myosin binding protein C, fast-type
Mybph	1.71	0.78	2.2E-04	5.0E-07	53311	myosin binding protein H
Myf5	0.43	-1.23	4.3E-04	1.2E-06	17877	myogenic factor 5
Myh11	0.67	-0.59	5.9E-02	6.2E-04	17880	myosin, heavy polypeptide 11, smooth muscle
Myh13	0.22	-2.15	2.9E-02	2.2E-04	544791	myosin, heavy polypeptide 13, skeletal muscle
Myh8	0.62	-0.70	1.4E-03	4.6E-06	17885	myosin, heavy polypeptide 8, skeletal muscle, perinatal
Myo5c	0.46	-1.11	3.4E-02	2.8E-04	208943	myosin VC
Myog	2.05	1.04	8.1E-07	6.3E-10	17928	myogenin
Myoz1	0.61	-0.72	9.4E-03	4.7E-05	59011	myozenin 1
Myoz3	0.32	-1.65	1.1E-10	3.2E-14	170947	myozenin 3
Ndrg2	0.51	-0.97	6.3E-06	7.0E-09	29811	N-myc downstream regulated gene 2
Nos1	0.27	-1.87	2.7E-11	4.5E-15	18125	nitric oxide synthase 1, neuronal
Notch3	0.66	-0.59	2.3E-02	1.5E-04	18131	notch 3
Otog	2.04	1.03	3.3E-02	2.6E-04	18419	otogelin
P2ry2	0.50	-0.99	5.0E-02	5.0E-04	18442	purinergic receptor P2Y, G-protein coupled 2
Pacsin1	1.90	0.93	6.7E-02	7.8E-04	23969	protein kinase C and casein kinase substrate in neurons 1
Pde1c	0.46	-1.12	5.2E-02	5.4E-04	18575	phosphodiesterase 1C
Pdk2	0.63	-0.67	3.1E-04	7.5E-07	18604	pyruvate dehydrogenase kinase, isoenzyme 2
Perm1	0.49	-1.03	1.1E-06	9.7E-10	74183	PPARGC1 and ESRR induced regulator, muscle 1
Pgap1	1.53	0.61	2.4E-02	1.7E-04	241062	post-GPI attachment to proteins 1
Pgm2	0.62	-0.70	2.1E-04	4.5E-07	72157	phosphoglucomutase 2
Phkg1	0.43	-1.22	1.6E-03	5.4E-06	18682	phosphorylase kinase gamma 1
Pitpnc1	0.65	-0.63	2.0E-03	7.3E-06	71795	phosphatidylinositol transfer protein, cytoplasmic 1
Pla2g16	0.56	-0.83	2.6E-05	3.4E-08	225845	phospholipase A2, group XVI
Plcd4	0.19	-2.42	5.8E-11	1.1E-14	18802	phospholipase C, delta 4
Plin4	0.24	-2.08	3.1E-08	2.2E-11	57435	perilipin 4
Plin5	0.40	-1.34	4.2E-03	1.6E-05	66968	perilipin 5
Pm20d2	0.56	-0.84	1.1E-02	5.7E-05	242377	peptidase M20 domain containing 2
Pmaip1	3.76	1.91	5.6E-10	1.8E-13	58801	phorbol-12-myristate-13-acetate-induced protein 1
Ppara	0.57	-0.82	3.7E-05	5.2E-08	19013	peroxisome proliferator activated receptor alpha
Ppargc1a	0.63	-0.67	5.9E-02	6.4E-04	19017	peroxisome proliferative activated receptor, gamma, coactivator 1 alpha
Ppp1r12b	0.62	-0.69	1.3E-04	2.6E-07	329251	protein phosphatase 1, regulatory (inhibitor) subunit 12B
Ppp1r14b	1.65	0.72	2.9E-04	7.2E-07	18938	protein phosphatase 1, regulatory (inhibitor) subunit 14B
Ppp1r14c	0.60	-0.75	1.5E-04	2.9E-07	76142	protein phosphatase 1, regulatory (inhibitor) subunit 14c
Ppp1r1a	0.47	-1.08	7.7E-03	3.6E-05	58200	protein phosphatase 1, regulatory (inhibitor) subunit 1A
Ppp1r3a	0.59	-0.75	4.8E-03	1.9E-05	140491	protein phosphatase 1, regulatory (inhibitor) subunit 3A
Prune2	2.56	1.36	2.1E-15	6.8E-20	353211	prune homolog 2
Pvalb	0.34	-1.56	1.9E-02	1.2E-04	19293	parvalbumin
Pygm	0.60	-0.74	1.6E-04	3.2E-07	19309	muscle glycogen phosphorylase
Rap1gap2	1.66	0.73	1.2E-03	3.6E-06	380711	RAP1 GTPase activating protein 2
Rassf4	0.66	-0.59	7.7E-03	3.6E-05	213391	Ras association (RalGDS/AF-6) domain family member 4
Rilp	0.49	-1.04	7.4E-02	8.8E-04	280408	Rab interacting lysosomal protein
Rpl3l	0.25	-2.02	6.7E-05	1.2E-07	66211	ribosomal protein L3-like
Runx1	1.66	0.73	7.6E-02	9.3E-04	12394	runt related transcription factor 1
Ryr3	0.60	-0.74	2.7E-04	6.5E-07	20192	ryanodine receptor 3
Scn4b	2.02	1.02	1.2E-04	2.1E-07	399548	sodium channel, type IV, beta
Sema3e	2.10	1.07	1.5E-02	9.2E-05	20349	sema domain, immunoglobulin domain (Ig), short basic domain, secreted, (semaphorin) 3E
Sema6b	1.59	0.67	6.3E-03	2.9E-05	20359	sema domain, transmembrane domain (TM), and cytoplasmic domain, (semaphorin) 6B
Slc25a34	0.39	-1.37	6.2E-04	1.9E-06	384071	solute carrier family 25, member 34
Slc2a4	0.64	-0.65	6.0E-04	1.8E-06	20528	solute carrier family 2 (facilitated glucose transporter), member 4
Slc41a1	0.63	-0.67	1.6E-04	3.3E-07	98396	solute carrier family 41, member 1
Slc43a2	1.63	0.71	1.4E-02	7.8E-05	215113	solute carrier family 43, member 2
Snap91	1.92	0.94	6.0E-03	2.6E-05	20616	synaptosomal-associated protein 91
Sox11	1.54	0.62	6.8E-03	3.1E-05	20666	SRY-box containing gene 11
Spg21	2.17	1.12	1.0E-09	3.9E-13	27965	spastic paraplegia 21 homolog
Spin1	see Supplementary Table 1e				20729	spindlin 1
Ston2	0.64	-0.64	1.4E-03	4.5E-06	108800	stonin 2
Sypl2	0.60	-0.73	5.6E-05	9.1E-08	17306	synaptophysin-like 2
Tcea3	0.59	-0.76	5.0E-03	2.1E-05	21401	transcription elongation factor A (SII), 3
Tceal5	0.59	-0.77	8.3E-03	4.0E-05	331532	transcription elongation factor A (SII)-like 5
Tenm4	0.60	-0.74	1.1E-03	3.4E-06	23966	teneurin transmembrane protein 4
Tmem233	0.38	-1.39	9.4E-03	4.6E-05	545798	transmembrane protein 233
Tmem52	0.22	-2.22	2.1E-08	1.1E-11	69671	transmembrane protein 52
Tmem72	0.23	-2.12	2.1E-04	4.4E-07	319776	transmembrane protein 72
Tmod4	0.63	-0.66	2.4E-02	1.6E-04	50874	tropomodulin 4
Tnmd	1.86	0.89	7.7E-02	9.4E-04	64103	tenomodulin
Tnnc1	1.75	0.81	4.6E-03	1.8E-05	21924	troponin C, cardiac/slow skeletal
Tnni2	0.65	-0.61	2.6E-02	1.8E-04	21953	troponin I, skeletal, fast 2
Tnnt2	1.54	0.62	2.4E-02	1.7E-04	21956	troponin T2, cardiac
Tnnt3	0.66	-0.60	3.0E-02	2.3E-04	21957	troponin T3, skeletal, fast
Trdn	0.65	-0.61	4.3E-02	4.2E-04	76757	triadin
Trem2	3.13	1.64	4.3E-04	1.2E-06	83433	triggering receptor expressed on myeloid cells 2
Tst	0.31	-1.69	5.8E-04	1.7E-06	22117	thiosulfate sulfurtransferase, mitochondrial
Vgll2	1.52	0.60	3.4E-02	2.9E-04	215031	vestigial like 2 homolog
Wnk2	0.57	-0.80	1.5E-03	4.9E-06	75607	WNK lysine deficient protein kinase 2
Wnt4	0.45	-1.14	5.7E-05	9.6E-08	22417	wingless-related MMTV integration site 4

upregulated (red)
downregulated (blue)

Supplementary Table 1c: Genes differentially expressed in SkM of control mice at E16.5 compared to E15.5.

Gene symbol	FC (E16.5 vs. E15.5)	logFC (E16.5 vs. E15.5)	FDR	p-value	Entrez gene	Protein name
0610009L18Rik	2.79	1.48	2.7E-06	1.3E-07	66838	RIKEN cDNA 0610009L18 gene
0610038B21Rik	1.76	0.82	4.0E-03	4.7E-04	70345	RIKEN cDNA 0610038B21 gene
0610040F04Rik	1.94	0.96	4.4E-04	3.7E-05	75394	RIKEN cDNA 0610040F04 gene
1110001J03Rik	1.94	0.95	1.0E-04	7.1E-06	66117	RIKEN cDNA 1110001J03 gene
1110007C09Rik	1.84	0.88	7.3E-06	3.9E-07	68480	RIKEN cDNA 1110007C09 gene
1110008P14Rik	1.87	0.90	1.8E-05	1.1E-06	73737	RIKEN cDNA 1110008P14 gene
1110054M08Rik	1.95	0.96	2.1E-04	1.6E-05	68841	RIKEN cDNA 1110054M08 gene
1110058L19Rik	1.52	0.61	6.4E-03	8.4E-04	68002	RIKEN cDNA 1110058L19 gene
1500011K16Rik	1.64	0.71	5.3E-03	6.7E-04	67885	RIKEN cDNA 1500011K16 gene
1500017E21Rik	2.66	1.41	1.1E-11	2.5E-13	668215	RIKEN cDNA 1500017E21 gene
1700001G11Rik	1.85	0.89	2.7E-03	3.0E-04	69303	RIKEN cDNA 1700001G11 gene
1700021F05Rik	1.78	0.83	3.3E-05	2.0E-06	67851	RIKEN cDNA 1700021F05 gene
1700094J05Rik	1.76	0.81	2.0E-03	2.1E-04	67523	RIKEN cDNA 1700094J05 gene
1700095B10Rik	3.54	1.82	1.6E-13	3.0E-15	67360	RIKEN cDNA 1700095B10 gene
1700113A16Rik	1.74	0.80	6.0E-05	4.1E-06	76642	RIKEN cDNA 1700113A16 gene
1810011O10Rik	1.59	0.67	1.6E-04	1.2E-05	69068	RIKEN cDNA 1810011O10 gene
1810041L15Rik	1.68	0.75	4.4E-05	2.8E-06	72301	RIKEN cDNA 1810041L15 gene
2010107E04Rik	1.98	0.98	2.0E-10	5.3E-12	70257	RIKEN cDNA 2010107E04 gene
2010107G23Rik	0.23	-2.12	6.9E-08	2.6E-09	69894	RIKEN cDNA 2010107G23 gene
2010300C02Rik	0.54	-0.88	4.5E-03	5.6E-04	72097	RIKEN cDNA 2010300C02 gene
2300002M23Rik	0.00	-8.38	2.0E-47	1.8E-50	69542	RIKEN cDNA 2300002M23 gene
2310002L09Rik	3.48	1.80	9.2E-20	8.5E-22	71886	RIKEN cDNA 2310002L09 gene
2310007B03Rik	0.14	-2.87	1.7E-03	1.8E-04	71874	RIKEN cDNA 2310007B03 gene
2310015B20Rik	1.79	0.84	5.3E-04	4.6E-05	69563	RIKEN cDNA 2310015B20 gene
2310022A10Rik	1.88	0.91	3.4E-11	8.0E-13	66367	RIKEN cDNA 2310022A10 gene
2310034C09Rik	0.00	-11.35	6.6E-211	2.2E-215	117172	RIKEN cDNA 2310034C09 gene
2310040G24Rik	4.88	2.29	7.7E-13	1.5E-14	381792	RIKEN cDNA 2310040G24 gene
2310043L19Rik	2.77	1.47	5.1E-07	2.2E-08	75589	RIKEN cDNA 2310043L19 gene
2310057N15Rik	0.00	-8.15	1.5E-22	1.1E-24	69696	RIKEN cDNA 2310057N15 gene
2310061I04Rik	2.24	1.16	2.1E-10	5.6E-12	69662	RIKEN cDNA 2310061I04 gene
2410002F23Rik	0.64	-0.64	6.9E-06	3.7E-07	668661	RIKEN cDNA 2410002F23 gene
2410016O06Rik	0.61	-0.71	1.1E-03	1.0E-04	71952	RIKEN cDNA 2410016O06 gene
2610035D17Rik	0.56	-0.83	4.6E-03	5.7E-04	72386	RIKEN cDNA 2610035D17 gene
2610318N02Rik	0.44	-1.19	3.1E-04	2.5E-05	70458	RIKEN cDNA 2610318N02 gene
2700038G22Rik	0.55	-0.87	5.4E-03	6.8E-04	67194	RIKEN cDNA 2700038G22 gene
3110057O12Rik	3.61	1.85	2.6E-14	4.2E-16	269423	RIKEN cDNA 3110057O12 gene
3425401B19Rik	2.76	1.47	2.7E-14	4.4E-16	100504518	RIKEN cDNA 3425401B19 gene
4430402I18Rik	4.66	2.22	1.3E-09	3.8E-11	381218	RIKEN cDNA 4430402I18 gene
4632428N05Rik	1.53	0.62	3.5E-05	2.2E-06	74048	RIKEN cDNA 4632428N05 gene
4833403I15Rik	2.58	1.37	2.1E-10	5.7E-12	74574	RIKEN cDNA 4833403I15 gene
4833412C05Rik	2.69	1.43	3.2E-05	2.0E-06	73904	RIKEN cDNA 4833412C05 gene
4930412C18Rik	0.43	-1.23	5.2E-03	6.6E-04	320152	RIKEN cDNA 4930412C18 gene
4930427A07Rik	0.56	-0.83	8.5E-05	6.0E-06	104732	RIKEN cDNA 4930427A07 gene
4930429F24Rik	2.42	1.28	1.4E-07	5.5E-09	74633	RIKEN cDNA 4930429F24 gene
5330439B14Rik	2.00	1.00	3.1E-04	2.5E-05	321015	RIKEN cDNA 5330439B14 gene
5430416N02Rik	0.61	-0.71	7.4E-03	1.0E-03	100503199	RIKEN cDNA 5430416N02 gene
5730508B09Rik	1.60	0.68	5.2E-03	6.5E-04	70617	RIKEN cDNA 5730508B09 gene
5930412G12Rik	2.09	1.06	1.2E-07	4.6E-09	319616	RIKEN cDNA 5930412G12 gene
6030419C18Rik	0.62	-0.70	1.8E-03	1.9E-04	319477	RIKEN cDNA 6030419C18 gene
6330410L21Rik	4.95	2.31	9.8E-13	2.0E-14	319224	RIKEN cDNA 6330410L21 gene
6430411K18Rik	2.72	1.44	2.1E-19	2.0E-21	76880	RIKEN cDNA 6430411K18 gen
6430571L13Rik	8.59	3.10	2.0E-23	1.4E-25	235599	RIKEN cDNA 6430571L13 gene
8430408G22Rik	4.44	2.15	5.4E-27	2.7E-29	213393	RIKEN cDNA 8430408G22 gene
8430419L09Rik	0.65	-0.61	1.4E-04	1.0E-05	74525	RIKEN cDNA 8430419L09 gene
9330182L06Rik	0.55	-0.86	2.5E-03	2.7E-04	231014	RIKEN cDNA 9330182L06 gene
9430015G10Rik	0.61	-0.71	2.1E-04	1.6E-05	230996	RIKEN cDNA 9430015G10 gene
A230070E04Rik	0.27	-1.90	4.6E-08	1.7E-09	100043072	RIKEN cDNA A230070E04 gene
A2m	0.44	-1.18	1.2E-04	8.9E-06	232345	alpha-2-macroglobulin
A630019I02Rik	7.97	3.00	8.6E-19	8.9E-21	408254	uncharacterized LOC408254
A930003A15Rik	2.62	1.39	1.3E-05	7.1E-07	68162	RIKEN cDNA A930003A15 gene
A930016O22Rik	6.91	2.79	7.7E-35	1.6E-37	100503495	RIKEN cDNA A930016O22 gene
AA986860	0.30	-1.73	1.8E-05	1.0E-06	212439	expressed sequence AA986860
Aaas	0.66	-0.59	1.6E-03	1.7E-04	223921	achalasia, adrenocortical insufficiency, alacrimia
Aacs	0.56	-0.83	5.4E-07	2.3E-08	78894	acetoacetyl-CoA synthetase
Aamdc	1.85	0.89	6.4E-07	2.8E-08	66273	adipogenesis associated Mth938 domain containing
Aatk	1.61	0.68	4.4E-04	3.7E-05	11302	apoptosis-associated tyrosine kinase
Abca8a	1.98	0.99	9.7E-10	2.7E-11	217258	ATP-binding cassette, sub-family A (ABC1), member 8a
Abcc9	2.14	1.10	1.2E-11	2.8E-13	20928	ATP-binding cassette, sub-family C (CFTR/MRP), member 9
Abcd2	3.16	1.66	8.5E-07	3.8E-08	26874	ATP-binding cassette, sub-family D (ALD), member 2
Abcg4	0.51	-0.97	5.2E-03	6.5E-04	192663	ATP-binding cassette, sub-family G (WHITE), member 4
Abhd6	0.49	-1.02	1.3E-03	1.3E-04	66082	abhydrolase domain containing 6
Ablim1	0.63	-0.66	5.0E-03	6.2E-04	226251	actin-binding LIM protein 1
Ablim2	2.59	1.37	3.4E-11	8.1E-13	231148	actin-binding LIM protein 2
Abra	2.27	1.18	1.6E-06	7.6E-08	223513	actin-binding Rho activating protein
Acaa2	2.73	1.45	3.3E-24	2.2E-26	52538	acetyl-Coenzyme A acyltransferase 2 (mitochondrial 3-oxoacyl-Coenzyme A thiolase)
Acacb	6.04	2.59	4.8E-51	2.6E-54	100705	acetyl-Coenzyme A carboxylase beta
Acad12	1.76	0.82	4.0E-03	4.8E-04	338350	acyl-Coenzyme A dehydrogenase family, member 12
Acadl	3.27	1.71	1.5E-31	5.1E-34	11363	acyl-Coenzyme A dehydrogenase, long-chain
Acadm	3.03	1.60	3.1E-29	1.3E-31	11364	acyl-Coenzyme A dehydrogenase, medium chain
Acads	2.05	1.04	1.4E-12	2.8E-14	11409	acyl-Coenzyme A dehydrogenase, short chain
Acadvl	2.34	1.23	7.1E-17	8.9E-19	11370	acyl-Coenzyme A dehydrogenase, very long chain
Acat2	0.65	-0.61	4.6E-03	5.6E-04	110460	acetyl-Coenzyme A acetyltransferase 2
Ace	3.09	1.63	7.7E-12	1.7E-13	11421	angiotensin I converting enzyme (peptidyl-dipeptidase A) 1
Acer2	1.92	0.94	5.6E-04	4.9E-05	230379	alkaline ceramidase 2
Ache	1.54	0.62	2.1E-03	2.2E-04	11423	acetylcholinesterase
Acly	0.50	-0.99	2.1E-08	7.0E-10	104112	ATP citrate lyase
Aco2	2.75	1.46	1.6E-23	1.1E-25	11429	aconitase 2, mitochondrial
Acot1	0.45	-1.16	7.8E-04	7.2E-05	26897	acyl-CoA thioesterase 1
Acot11	2.40	1.26	2.9E-17	3.4E-19	329910	acyl-CoA thioesterase 11
Acot13	2.01	1.00	2.1E-07	8.5E-09	66834	acyl-CoA thioesterase 13
Acsl1	1.69	0.76	6.8E-03	9.1E-04	14081	acyl-CoA synthetase long-chain family member 1
Acsl5	0.65	-0.62	2.7E-04	2.1E-05	433256	acyl-CoA synthetase long-chain family member 5
Acsl6	2.31	1.21	7.4E-05	5.1E-06	216739	acyl-CoA synthetase long-chain family member 6
Acss1	1.94	0.96	1.1E-06	5.2E-08	68738	acyl-CoA synthetase short-chain family member 1
Acss2	2.87	1.52	2.0E-25	1.3E-27	60525	acyl-CoA synthetase short-chain family member 2
Acss2os	2.79	1.48	4.5E-07	1.9E-08	74945	acyl-CoA synthetase short-chain family member 2, opposite strand
Acta1	5.31	2.41	3.3E-31	1.1E-33	11459	actin, alpha 1, skeletal muscle
Actc1	2.80	1.49	4.1E-16	5.4E-18	11464	actin, alpha, cardiac muscle 1
Actg2	2.71	1.44	2.0E-09	5.8E-11	11468	actin, gamma 2, smooth muscle, enteric
Actn2	2.96	1.56	6.5E-17	8.0E-19	11472	actinin alpha 2
Actn3	6.47	2.69	8.6E-36	1.4E-38	11474	actinin alpha 3
Acyp2	2.53	1.34	1.8E-06	8.6E-08	75572	acylphosphatase 2, muscle type
Adamts17	0.61	-0.71	5.1E-06	2.7E-07	233332	a disintegrin-like and metallopeptidase (reprolysin type) with thrombospondin type 1 motif, 17
Adamts3	0.62	-0.70	6.9E-03	9.2E-04	330119	a disintegrin-like and metallopeptidase (reprolysin type) with thrombospondin type 1 motif, 3
Adamtsl4	1.94	0.96	6.4E-08	2.4E-09	229595	ADAMTS-like 4
Adck3	7.55	2.92	1.6E-48	1.2E-51	67426	aarF domain containing kinase 3
Adcy2	1.88	0.91	3.1E-11	7.4E-13	210044	adenylate cyclase 2
Adcy9	1.83	0.87	1.2E-05	6.5E-07	11515	adenylate cyclase 9
Adhfe1	7.38	2.88	1.1E-08	3.4E-10	76187	alcohol dehydrogenase, iron containing, 1
Adm	1.86	0.90	1.6E-03	1.6E-04	11535	adrenomedullin
Adora2a	1.74	0.80	1.8E-03	1.8E-04	11540	adenosine A2a receptor
Adprhl1	3.30	1.72	7.3E-14	1.3E-15	234072	ADP-ribosylhydrolase like 1
Adra2b	3.00	1.58	1.8E-06	8.6E-08	11552	adrenergic receptor, alpha 2b
Adrb2	1.95	0.96	2.0E-04	1.5E-05	11555	adrenergic receptor, beta 2
Adssl1	3.38	1.76	4.0E-23	2.9E-25	11565	adenylosuccinate synthetase like 1
AF357359	2.06	1.04	5.3E-06	2.8E-07	100303647	snoRNA AF357359
Afap1l1	1.91	0.93	2.4E-08	8.3E-10	106877	actin filament associated protein 1-like 1
Aff1	2.19	1.13	4.3E-15	6.5E-17	17355	AF4/FMR2 family, member 1
Aff2	0.51	-0.98	1.0E-03	9.5E-05	14266	AF4/FMR2 family, member 2
Agbl1	2.96	1.56	1.7E-05	9.6E-07	244071	ATP/GTP binding protein-like 1
Agbl5	0.63	-0.67	5.2E-03	6.5E-04	231093	ATP/GTP binding protein-like 5
Agl	3.29	1.72	1.2E-19	1.1E-21	77559	amylo-1,6-glucosidase, 4-alpha-glucanotransferase
Ago2	1.84	0.88	2.8E-06	1.4E-07	239528	argonaute RISC catalytic subunit 2
Agpat9	5.23	2.39	1.0E-09	2.8E-11	231510	1-acylglycerol-3-phosphate O-acyltransferase 9
Agt	9.07	3.18	8.9E-28	4.3E-30	11606	angiotensinogen (serpin peptidase inhibitor, clade A, member 8)
Agtr1a	1.55	0.63	3.1E-03	3.5E-04	11607	angiotensin II receptor, type 1a
AI464131	3.19	1.67	2.5E-26	1.4E-28	329828	expressed sequence AI464131
Aifm2	1.78	0.83	2.3E-03	2.5E-04	71361	apoptosis-inducing factor, mitochondrion-associated 2

Aifm3	1.91	0.93	4.3E-03	5.2E-04	72168	apoptosis-inducing factor, mitochondrion-associated 3
Ak1	3.53	1.82	1.4E-25	8.7E-28	11636	adenylate kinase 1
Akap1	2.69	1.43	1.9E-23	1.4E-25	11640	A kinase (PRKA) anchor protein 1
Akap6	1.76	0.82	3.4E-05	2.1E-06	238161	A kinase (PRKA) anchor protein 6
Akap7	1.74	0.80	8.6E-05	6.0E-06	432442	A kinase (PRKA) anchor protein 7
Akr1b10	1.94	0.96	5.0E-05	3.3E-06	67861	aldo-keto reductase family 1, member B10 (aldose reductase)
Akr1c14	2.29	1.19	3.5E-03	4.1E-04	105387	aldo-keto reductase family 1, member C14
Alas1	1.85	0.89	2.3E-09	7.0E-11	11655	aminolevulinic acid synthase 1
Alcam	0.50	-1.00	3.7E-09	1.1E-10	11658	activated leukocyte cell adhesion molecule
Aldh1a2	1.52	0.60	6.6E-03	8.8E-04	19378	aldehyde dehydrogenase family 1, subfamily A2
Aldh1a7	0.29	-1.79	2.5E-03	2.8E-04	26358	aldehyde dehydrogenase family 1, subfamily A7
Aldh1l2	1.64	0.72	1.4E-05	8.1E-07	216188	aldehyde dehydrogenase 1 family, member L2
Aldh4a1	2.27	1.18	1.3E-17	1.5E-19	212647	aldehyde dehydrogenase 4 family, member A1
Aldh6a1	1.82	0.86	7.9E-05	5.5E-06	104776	aldehyde dehydrogenase family 6, subfamily A1
Aldoa	3.34	1.74	3.3E-31	1.1E-33	11674	aldolase A, fructose-bisphosphate
Aldoart1	3.51	1.81	7.5E-34	1.8E-36	353204	aldolase 1 A retrogene 1
Alkbh3	1.67	0.74	2.1E-05	1.3E-06	69113	alkB, alkylation repair homolog 3
Alox12	0.39	-1.38	1.4E-03	1.4E-04	11684	arachidonate 12-lipoxygenase
Alpk2	1.66	0.73	1.4E-03	1.3E-04	225638	alpha-kinase 2
Alpk3	2.26	1.17	1.2E-10	3.1E-12	116904	alpha-kinase 3
Amigo1	1.88	0.91	1.4E-11	3.2E-13	229715	adhesion molecule with Ig like domain 1
Amotl1	1.99	0.99	7.0E-11	1.7E-12	75723	angiomotin-like 1
Ampd1	4.69	2.23	5.3E-28	2.5E-30	229665	adenosine monophosphate deaminase 1
Amtn	0.24	-2.06	7.0E-10	1.9E-11	71421	amelotin
Angptl2	1.67	0.74	1.6E-06	7.4E-08	26360	angiopoietin-like 2
Ank1	2.95	1.56	1.2E-19	1.2E-21	11733	ankyrin 1, erythroid
Ank3	2.03	1.02	2.0E-15	2.9E-17	11735	ankyrin 3, epithelial
Ankrd13d	0.40	-1.30	1.8E-06	8.3E-08	68423	ankyrin repeat domain 13 family, member D
Ankrd2	2.33	1.22	6.5E-04	5.8E-05	56642	ankyrin repeat domain 2 (stretch responsive muscle)
Ankrd23	2.25	1.17	7.1E-11	1.8E-12	78321	ankyrin repeat domain 23
Ankrd33b	2.21	1.15	1.6E-05	9.1E-07	67434	ankyrin repeat domain 33B
Ankrd37	3.21	1.68	2.1E-11	4.9E-13	654824	ankyrin repeat domain 37
Ankrd40	1.64	0.72	2.6E-07	1.0E-08	71452	ankyrin repeat domain 40
Ankrd9	3.68	1.88	2.3E-24	1.6E-26	74251	ankyrin repeat domain 9
Anxa1	0.33	-1.60	1.9E-17	2.3E-19	16952	annexin A1
Anxa11	2.18	1.12	1.2E-12	2.5E-14	11744	annexin A11
Anxa8	0.28	-1.83	9.6E-05	6.8E-06	11752	annexin A8
Aoc3	3.09	1.63	1.4E-18	1.5E-20	11754	amine oxidase, copper containing 3
Aox1	2.65	1.41	2.9E-09	8.8E-11	11761	aldehyde oxidase 1
Ap5s1	1.55	0.63	5.6E-03	7.2E-04	69596	adaptor-related protein 5 complex, sigma 1 subunit
Apcdd1	0.46	-1.11	4.0E-03	4.8E-04	494504	adenomatosis polyposis coli down-regulated 1
Apitd1	0.52	-0.94	2.1E-04	1.6E-05	69928	apoptosis-inducing, TAF9-like domain 1
Aplp1	0.46	-1.11	4.0E-07	1.7E-08	11803	amyloid beta (A4) precursor-like protein 1
Apobec2	3.33	1.74	3.2E-20	2.9E-22	11811	apolipoprotein B mRNA editing enzyme, catalytic polypeptide 2
Apoe	1.59	0.67	2.5E-05	1.5E-06	11816	apolipoprotein E
Apold1	2.29	1.20	1.6E-06	7.6E-08	381823	apolipoprotein L domain containing 1
Apool	1.94	0.95	7.8E-09	2.5E-10	68117	apolipoprotein O-like
Aqp11	3.27	1.71	1.4E-09	4.2E-11	66333	aquaporin 11
Aqp4	22.84	4.51	1.2E-19	1.2E-21	11829	aquaporin 4
Aqp7	9.68	3.28	1.9E-14	3.1E-16	11832	aquaporin 7
Arg1	0.09	-3.54	9.0E-23	6.6E-25	11846	arginase, liver
Arhgap10	1.88	0.91	1.2E-10	3.2E-12	78514	Rho GTPase activating protein 10
Arhgap26	1.64	0.71	9.1E-06	5.0E-07	71302	Rho GTPase activating protein 26
Arhgap36	4.24	2.08	1.5E-22	1.1E-24	75404	Rho GTPase activating protein 36
Arhgef19	0.51	-0.97	3.0E-06	1.5E-07	213649	Rho guanine nucleotide exchange factor (GEF) 19
Arhgef6	1.66	0.73	1.8E-05	1.1E-06	73341	Rac/Cdc42 guanine nucleotide exchange factor (GEF) 6
Arid3a	0.55	-0.86	5.8E-06	3.1E-07	13496	AT rich interactive domain 3A (BRIGHT-like)
Arl6ip5	1.89	0.92	6.6E-08	2.4E-09	65106	ADP-ribosylation factor-like 6 interacting protein 5
Art1	2.81	1.49	5.5E-18	6.1E-20	11870	ADP-ribosyltransferase 1
Art3	7.79	2.96	3.7E-48	2.9E-51	109979	ADP-ribosyltransferase 3
Art5	2.67	1.42	1.8E-08	6.2E-10	11875	ADP-ribosyltransferase 5
As3mt	1.82	0.86	4.3E-06	2.2E-07	57344	arsenic (+3 oxidation state) methyltransferase
Asb10	2.30	1.20	1.7E-05	9.6E-07	117590	ankyrin repeat and SOCS box-containing 10
Asb11	19.04	4.25	4.8E-30	1.9E-32	68854	ankyrin repeat and SOCS box-containing 11
Asb12	15.14	3.92	1.0E-28	4.4E-31	70392	ankyrin repeat and SOCS box-containing 12
Asb15	6.01	2.59	1.8E-13	3.3E-15	78910	ankyrin repeat and SOCS box-containing 15
Asb16	2.98	1.58	2.4E-10	6.5E-12	217217	ankyrin repeat and SOCS box-containing 16
Asb18	3.71	1.89	9.3E-08	3.5E-09	208372	ankyrin repeat and SOCS box-containing 18
Asb2	3.01	1.59	6.3E-17	7.8E-19	65256	ankyrin repeat and SOCS box-containing 2
Asb5	4.94	2.30	2.3E-18	2.5E-20	76294	ankyrin repeat and SOCs box-containing 5
Asb8	2.25	1.17	8.9E-16	1.2E-17	78541	ankyrin repeat and SOCS box-containing 8
Asns	1.94	0.96	2.0E-08	6.9E-10	27053	asparagine synthetase
Asph	4.32	2.11	1.3E-37	2.0E-40	65973	aspartate-beta-hydroxylase
Asphd2	0.56	-0.84	1.7E-03	1.8E-04	72898	aspartate beta-hydroxylase domain containing 2
Aspn	1.62	0.70	3.9E-05	2.5E-06	66695	asporin
Atcay	3.40	1.76	7.6E-14	1.3E-15	16467	ataxia, cerebellar, Cayman type homolog
Atcayos	4.32	2.11	1.5E-19	1.5E-21	69678	ataxia, cerebellar, Cayman type, opposite strand
Atf6b	0.64	-0.65	8.0E-07	3.5E-08	12915	activating transcription factor 6 beta
Atg4a	1.83	0.87	1.8E-05	1.0E-06	666468	autophagy related 4A, cysteine peptidase
Atp1a2	3.86	1.95	4.1E-30	1.6E-32	98660	ATPase, Na+/K+ transporting, alpha 2 polypeptide
Atp1b2	3.29	1.72	2.2E-35	3.8E-38	11932	ATPase, Na+/K+ transporting, beta 2 polypeptide
Atp1b4	5.60	2.49	7.5E-28	3.6E-30	67821	ATPase, (Na+)/K+ transporting, beta 4 polypeptide
Atp2a1	4.99	2.32	1.5E-32	4.5E-35	11937	ATPase, Ca++ transporting, cardiac muscle, fast twitch 1
Atp2a3	1.82	0.87	1.6E-04	1.2E-05	53313	ATPase, Ca++ transporting, ubiquitous
Atp2b3	3.26	1.71	2.4E-16	3.1E-18	320707	ATPase, Ca++ transporting, plasma membrane 3
Atp5a1	1.77	0.82	4.4E-09	1.4E-10	11946	ATP synthase, H+ transporting, mitochondrial F1 complex, alpha subunit 1
Atp5b	1.95	0.96	1.1E-12	2.2E-14	11947	ATP synthase, H+ transporting mitochondrial F1 complex, beta subunit
Atp5c1	1.96	0.97	1.0E-11	2.3E-13	11949	ATP synthase, H+ transporting, mitochondrial F1 complex, gamma polypeptide 1
Atp5d	1.67	0.74	1.9E-07	7.5E-09	66043	ATP synthase, H+ transporting, mitochondrial F1 complex, delta subunit
Atp5e	1.98	0.99	6.0E-07	2.6E-08	67126	ATP synthase, H+ transporting, mitochondrial F1 complex, epsilon subunit
Atp5f1	1.83	0.87	6.9E-09	2.2E-10	11950	ATP synthase, H+ transporting, mitochondrial F0 complex, subunit B1
Atp5g1	2.35	1.23	2.9E-15	4.3E-17	11951	ATP synthase, H+ transporting, mitochondrial F0 complex, subunit c1 (subunit 9)
Atp5g3	1.99	0.99	2.2E-10	5.9E-12	228033	ATP synthase, H+ transporting, mitochondrial F0 complex, subunit C3 (subunit 9)
Atp5h	1.74	0.80	4.3E-04	3.6E-05	71679	ATP synthase, H+ transporting, mitochondrial F0 complex, subunit d
Atp5j	1.71	0.77	1.5E-07	5.8E-09	11957	ATP synthase, H+ transporting, mitochondrial F0 complex, subunit F
Atp5j2	1.76	0.81	1.5E-07	5.9E-09	57423	ATP synthase, H+ transporting, mitochondrial F0 complex, subunit F2
Atp5k	1.87	0.91	6.0E-08	2.2E-09	11958	ATP synthase, H+ transporting, mitochondrial F1F0 complex, subunit e
Atp5o	2.07	1.05	7.1E-11	1.8E-12	28080	ATP synthase, H+ transporting, mitochondrial F1 complex, O subunit
Atp6v0e2	1.53	0.62	6.2E-03	8.0E-04	76252	ATPase, H+ transporting, lysosomal V0 subunit E2
Atp8a1	1.56	0.64	1.2E-03	1.2E-04	11980	ATPase, aminophospholipid transporter (APLT), class I, type 8A, member 1
Atraid	1.52	0.61	1.1E-05	6.1E-07	381629	all-trans retinoic acid induced differentiation factor
Auh	2.01	1.01	3.2E-07	1.3E-08	11992	AU RNA binding protein/enoyl-coenzyme A hydratase
AW551984	1.93	0.95	2.4E-08	8.2E-10	244810	expressed sequence AW551984
B230118H07Rik	1.60	0.68	1.7E-03	1.8E-04	68170	RIKEN cDNA B230118H07 gene
B230119M05Rik	2.62	1.39	1.1E-06	4.9E-08	77983	RIKEN cDNA B230119M05 gene
B3galt2	1.82	0.87	3.0E-04	2.4E-05	26878	UDP-Gal:betaGlcNAc beta 1,3-galactosyltransferase, polypeptide 2
B3gnt3	2.15	1.10	2.7E-06	1.3E-07	72297	UDP-GlcNAc:betaGal beta-1,3-N-acetylglucosaminyltransferase 3
B3gnt7	0.48	-1.06	1.1E-04	7.7E-06	227327	UDP-GlcNAc:betaGal beta-1,3-N-acetylglucosaminyltransferase 7
B4galnt4	0.23	-2.13	1.1E-18	1.2E-20	330671	beta-1,4-N-acetyl-galactosaminyl transferase 4
Bag2	1.71	0.78	1.2E-05	6.6E-07	213539	BCL2-associated athanogene 2
Bai2	0.36	-1.46	9.9E-13	2.0E-14	230775	brain-specific angiogenesis inhibitor 2
Baiap2	0.58	-0.78	4.7E-04	4.0E-05	108100	brain-specific angiogenesis inhibitor 1-associated protein 2
Bambi	0.58	-0.79	4.8E-04	4.1E-05	68010	BMP and activin membrane-bound inhibitor, homolog
BC002163	1.63	0.70	1.9E-05	1.1E-06	170658	NADH dehydrogenase Fe-S protein 5 pseudogene
BC022687	0.54	-0.88	3.3E-03	3.8E-04	217887	cDNA sequence BC022687
Bcas3	1.67	0.74	4.6E-06	2.4E-07	192197	breast carcinoma amplified sequence 3
Bche	2.85	1.51	1.4E-17	1.6E-19	12038	butyrylcholinesterase
Bcl11a	0.26	-1.94	2.2E-06	1.0E-07	14025	B cell CLL/lymphoma 11A (zinc finger protein)
Bcl11b	0.30	-1.75	7.1E-03	9.6E-04	58208	B cell leukemia/lymphoma 11B
Bcl6	0.54	-0.89	4.1E-04	3.4E-05	12053	B cell leukemia/lymphoma 6
Bcr	0.58	-0.80	5.2E-07	2.2E-08	110279	breakpoint cluster region
Bdh1	0.29	-1.77	1.8E-06	8.3E-08	71911	3-hydroxybutyrate dehydrogenase, type 1
Bean1	2.15	1.10	2.1E-06	1.0E-07	65115	brain expressed, associated with Nedd4, 1
Begain	0.63	-0.68	5.4E-03	6.9E-04	380785	brain-enriched guanylate kinase-associated
Bend5	0.61	-0.71	2.4E-03	2.6E-04	67621	BEN domain containing 5
Bend7	0.47	-1.08	1.3E-03	1.3E-04	209645	BEN domain containing 7
Best3	1.76	0.82	4.6E-04	3.9E-05	382427	bestrophin 3
Bex1	1.51	0.59	3.7E-03	4.4E-04	19716	brain expressed gene 1
Bex2	0.46	-1.11	9.7E-05	6.9E-06	12069	brain expressed X-linked 2
Bex4	0.53	-0.92	7.4E-04	6.7E-05	406217	brain expressed gene 4
Bhlhe40	2.02	1.01	2.1E-13	3.9E-15	20893	basic helix-loop-helix family, member e40

Bin1	2.42	1.27	5.2E-14	9.0E-16	30948	bridging integrator 1
Blnk	0.26	-1.96	4.7E-04	3.9E-05	17060	B cell linker
Bloc1s1	1.52	0.60	6.9E-04	6.2E-05	14533	biogenesis of lysosome-related organelles complex-1, subunit 1
Bmp7	0.49	-1.02	1.8E-06	8.3E-08	12162	bone morphogenetic protein 7
Bmper	1.58	0.66	7.9E-04	7.2E-05	73230	BMP-binding endothelial regulator
Bnip3	1.90	0.92	2.2E-08	7.5E-10	12176	BCL2/adenovirus E1B interacting protein 3
Bola3	1.94	0.95	6.0E-07	2.6E-08	78653	bolA-like 3
Bora	0.65	-0.63	2.4E-03	2.6E-04	77744	bora, aurora kinase A activator
Brinp1	0.32	-1.63	1.7E-09	5.1E-11	56710	bone morphogenic protein/retinoic acid inducible neural specific 1
Bsn	0.55	-0.86	5.2E-03	6.6E-04	12217	bassoon
Bspry	0.37	-1.45	1.0E-03	9.7E-05	192120	B-box and SPRY domain containing
Bst2	1.61	0.69	4.4E-03	5.4E-04	69550	bone marrow stromal cell antigen 2
Btbd1	1.94	0.95	1.1E-09	3.3E-11	83962	BTB (POZ) domain containing 1
Btbd11	0.49	-1.03	4.8E-05	3.1E-06	74007	BTB (POZ) domain containing 11
Btnl9	2.46	1.30	3.2E-07	1.3E-08	237754	butyrophilin-like 9
Bves	3.69	1.88	9.8E-22	8.0E-24	23828	blood vessel epicardial substance
C030006K11Rik	2.25	1.17	9.7E-06	5.4E-07	223665	RIKEN cDNA C030006K11 gene
C030037D09Rik	2.59	1.38	1.3E-06	5.9E-08	193280	RIKEN cDNA C030037D09 gene
C130021I20Rik	0.35	-1.50	6.8E-08	2.5E-09	100504399	Riken cDNA C130021I20 gene
C130080G10Rik	2.97	1.57	5.8E-09	1.8E-10	100303644	RIKEN cDNA C130080G10 gene
C1qtnf9	2.51	1.33	7.7E-11	1.9E-12	239126	C1q and tumor necrosis factor related protein 9
C1ra	3.07	1.62	2.6E-10	6.9E-12	50909	complement component 1, r subcomponent A
C1s1	3.19	1.68	1.5E-28	6.5E-31	50908	complement component 1, s subcomponent 1
C3	16.71	4.06	3.6E-10	9.9E-12	12266	complement component 3
C77080	0.64	-0.64	8.1E-04	7.5E-05	97130	expressed sequence C77080
Cachd1	0.62	-0.69	3.2E-05	2.0E-06	320508	cache domain containing 1
Cacna1c	0.65	-0.62	1.4E-03	1.5E-04	12288	calcium channel, voltage-dependent, L type, alpha 1C subunit
Cacna1d	0.50	-1.01	1.5E-04	1.1E-05	12289	calcium channel, voltage-dependent, L type, alpha 1D subunit
Cacna1e	0.51	-0.98	4.5E-03	5.5E-04	12290	calcium channel, voltage-dependent, R type, alpha 1E subunit
Cacna1g	0.55	-0.85	5.9E-07	2.5E-08	12291	calcium channel, voltage-dependent, T type, alpha 1G subunit
Cacna1i	2.41	1.27	8.7E-05	6.1E-06	239556	calcium channel, voltage-dependent, alpha 1I subunit
Cacna1s	2.96	1.57	8.0E-20	7.3E-22	12292	calcium channel, voltage-dependent, L type, alpha 1S subunit
Cacna2d1	1.81	0.85	1.3E-05	7.2E-07	12293	calcium channel, voltage-dependent, alpha2/delta subunit 1
Cacna2d3	0.47	-1.07	3.6E-04	2.9E-05	12294	calcium channel, voltage-dependent, alpha2/delta subunit 3
Cacnb1	2.69	1.43	2.7E-16	3.5E-18	12295	calcium channel, voltage-dependent, beta 1 subunit
Cacng1	4.57	2.19	1.3E-31	4.3E-34	12299	calcium channel, voltage-dependent, gamma subunit 1
Cacng4	0.46	-1.11	3.2E-04	2.6E-05	54377	calcium channel, voltage-dependent, gamma subunit 4
Cacng6	2.23	1.16	1.4E-07	5.3E-09	54378	calcium channel, voltage-dependent, gamma subunit 6
Cad	0.63	-0.66	3.1E-06	1.5E-07	69719	carbamoyl-phosphate synthetase 2, aspartate transcarbamylase, and dihydroorotase
Calcr	2.62	1.39	7.6E-07	3.3E-08	12311	calcitonin receptor
Calcrl	1.82	0.86	1.1E-06	4.7E-08	54598	calcitonin receptor-like
Calml4	2.15	1.10	3.5E-06	1.8E-07	75600	calmodulin-like 4
Calr	0.65	-0.63	1.2E-05	6.5E-07	12317	calreticulin
Camk1d	0.66	-0.59	3.4E-03	3.9E-04	227541	calcium/calmodulin-dependent protein kinase ID
Camk2a	1.96	0.97	5.2E-08	1.9E-09	12322	calcium/calmodulin-dependent protein kinase II alpha
Camk2b	5.66	2.50	2.8E-34	6.5E-37	12323	calcium/calmodulin-dependent protein kinase II, beta
Camk2d	1.54	0.62	2.1E-05	1.3E-06	108058	calcium/calmodulin-dependent protein kinase II, delta
Camkv	0.47	-1.08	9.7E-04	9.2E-05	235604	CaM kinase-like vesicle-associated
Camta1	1.96	0.97	2.9E-04	2.3E-05	100072	calmodulin binding transcription activator 1
Cap2	2.34	1.23	2.0E-11	4.6E-13	67252	CAP, adenylate cyclase-associated protein, 2
Capn3	3.28	1.71	1.5E-22	1.1E-24	12335	calpain 3
Car12	0.30	-1.74	2.0E-06	9.3E-08	76459	carbonic anyhydrase 12
Car14	3.73	1.90	2.9E-16	3.7E-18	23831	carbonic anhydrase 14
Car3	9.16	3.20	3.9E-27	1.9E-29	12350	carbonic anhydrase 3
Card14	0.38	-1.40	2.5E-08	8.5E-10	170720	caspase recruitment domain family, member 14
Carmn	1.87	0.90	2.5E-03	2.7E-04	328968	cardiac mesoderm enhancer-associated non-coding RNA
Carns1	3.39	1.76	5.5E-16	7.5E-18	107239	carnosine synthase 1
Casd1	1.59	0.67	2.2E-05	1.3E-06	213819	CAS1 domain containing 1
Casq1	7.45	2.90	1.4E-39	2.1E-42	12372	calsequestrin 1
Casq2	3.98	1.99	4.6E-25	2.9E-27	12373	calsequestrin 2
Cav1	1.91	0.93	1.0E-10	2.6E-12	12389	caveolin 1, caveolae protein
Cav2	1.82	0.87	1.2E-06	5.7E-08	12390	caveolin 2
Cav3	2.85	1.51	2.6E-17	3.1E-19	12391	caveolin 3
Cbr4	1.69	0.76	8.6E-04	8.0E-05	234309	carbonyl reductase 4
Cbs	0.49	-1.02	2.1E-03	2.2E-04	12411	cystathionine beta-synthase
Ccdc106	0.42	-1.25	5.6E-06	2.9E-07	232821	coiled-coil domain containing 106
Ccdc141	1.93	0.95	4.2E-07	1.8E-08	545428	coiled-coil domain containing 141
Ccdc43	1.75	0.81	2.4E-07	9.5E-09	52715	coiled-coil domain containing 43
Ccdc69	3.26	1.70	5.8E-14	1.0E-15	52570	coiled-coil domain containing 69
Ccdc85a	1.85	0.89	4.6E-04	3.9E-05	216613	coiled-coil domain containing 85A
Ccdc88c	1.93	0.95	2.5E-10	6.6E-12	68339	coiled-coil domain containing 88C
Ccdc91	1.53	0.62	1.1E-04	7.9E-06	67015	coiled-coil domain containing 91
Ccdc92	0.32	-1.63	1.7E-04	1.2E-05	215707	coiled-coil domain containing 92
Ccl21a	0.55	-0.86	7.2E-03	9.7E-04	18829	chemokine (C-C motif) ligand 21A (serine)
Ccnd2	0.60	-0.75	9.3E-07	4.1E-08	12444	cyclin D2
Ccne1	0.61	-0.71	1.8E-05	1.1E-06	12447	cyclin E1
Ccng1	3.04	1.60	9.1E-22	7.4E-24	12450	cyclin G1
Cd163	4.40	2.14	1.6E-17	1.8E-19	93671	CD163 antigen
Cd300lg	4.14	2.05	1.6E-16	2.0E-18	52685	CD300 antigen like family member G
Cd33	1.86	0.90	2.5E-03	2.7E-04	12489	CD33 antigen
Cd36	3.35	1.75	6.2E-22	4.9E-24	12491	CD36 antigen
Cd59a	2.14	1.09	1.6E-06	7.2E-08	12509	CD59a antigen
Cd84	1.82	0.86	9.6E-04	9.1E-05	12523	CD84 antigen
Cd93	1.52	0.60	2.5E-05	1.5E-06	17064	CD93 antigen
Cd97	1.50	0.59	6.2E-04	5.5E-05	26364	CD97 antigen
Cdc34	1.95	0.96	5.0E-09	1.6E-10	216150	cell division cycle 34
Cdc42bpg	0.62	-0.68	2.0E-03	2.1E-04	240505	CDC42 binding protein kinase gamma (DMPK-like)
Cdc42ep2	1.96	0.97	1.1E-11	2.4E-13	104252	CDC42 effector protein (Rho GTPase binding) 2
Cdca7	0.58	-0.79	1.4E-09	4.2E-11	66953	cell division cycle associated 7
Cdca7l	0.65	-0.63	3.0E-03	3.4E-04	217946	cell division cycle associated 7 like
Cdh11	0.63	-0.67	1.9E-05	1.1E-06	12552	cadherin 11
Cdh13	3.02	1.59	7.1E-29	3.0E-31	12554	cadherin 13
Cdh24	0.52	-0.93	1.3E-04	9.5E-06	239096	cadherin-like 24
Cdh3	0.31	-1.71	1.8E-10	4.7E-12	12560	cadherin 3
Cdh6	0.51	-0.97	1.1E-03	1.1E-04	12563	cadherin 6
Cdhr3	3.29	1.72	1.3E-07	4.9E-09	68764	cadherin-related family member 3
Ceacam1	1.92	0.94	6.7E-04	6.0E-05	26365	carcinoembryonic antigen-related cell adhesion molecule 1
Cebpd	2.02	1.02	1.4E-05	8.3E-07	12609	CCAAT/enhancer binding protein (C/EBP), delta
Celsr1	0.38	-1.40	2.6E-03	2.9E-04	12614	cadherin, EGF LAG seven-pass G-type receptor 1
Celsr2	0.45	-1.17	2.9E-03	3.3E-04	53883	cadherin, EGF LAG seven-pass G-type receptor 2
Cep170b	0.48	-1.07	1.4E-03	1.4E-04	217882	centrosomal protein 170B
Cep85	2.16	1.11	1.2E-15	1.8E-17	70012	centrosomal protein 85
Cep85l	1.94	0.96	2.3E-05	1.4E-06	100038725	centrosomal protein 85-like
Cers1	1.57	0.65	9.7E-04	9.1E-05	93898	ceramide synthase 1
Cfl2	2.54	1.34	2.5E-15	3.8E-17	12632	cofilin 2, muscle
Chaf1a	0.64	-0.64	1.1E-06	5.2E-08	27221	chromatin assembly factor 1, subunit A (p150)
Chaf1b	0.64	-0.65	1.8E-04	1.3E-05	110749	chromatin assembly factor 1, subunit B (p60)
Chchd10	5.89	2.56	3.0E-42	3.6E-45	103172	coiled-coil-helix-coiled-coil-helix domain containing 10
Chchd3	2.18	1.12	1.0E-14	1.6E-16	66075	coiled-coil-helix-coiled-coil-helix domain containing 3
Chd3	0.56	-0.83	4.7E-08	1.7E-09	216848	chromodomain helicase DNA binding protein 3
Chd5	0.29	-1.77	2.5E-03	2.8E-04	269610	chromodomain helicase DNA binding protein 5
Chd7	1.53	0.61	1.4E-04	1.0E-05	320790	chromodomain helicase DNA binding protein 7
Chrac1	1.54	0.63	6.0E-04	5.3E-05	93696	chromatin accessibility complex 1
Chrdl1	2.21	1.14	1.2E-05	6.6E-07	83453	chordin-like 1
Chrm3	0.51	-0.97	5.9E-03	7.6E-04	12671	cholinergic receptor, muscarinic 3, cardiac
Chrna9	13.55	3.76	1.8E-30	6.4E-33	231252	cholinergic receptor, nicotinic, alpha polypeptide 9
Chrnb1	1.70	0.76	1.1E-05	6.1E-07	11443	cholinergic receptor, nicotinic, beta polypeptide 1 (muscle)
Chrnd	2.00	1.00	4.2E-08	1.5E-09	11447	cholinergic receptor, nicotinic, delta polypeptide
Chrng	1.70	0.77	6.1E-05	4.2E-06	11449	cholinergic receptor, nicotinic, gamma polypeptide
Chst10	0.55	-0.87	4.6E-04	3.9E-05	98388	carbohydrate sulfotransferase 10
Chst2	0.61	-0.70	3.9E-06	2.0E-07	54371	carbohydrate sulfotransferase 2
Cidea	5.27	2.40	2.0E-15	2.9E-17	12683	cell death-inducing DNA fragmentation factor, alpha subunit-like effector A
Cilp	1.53	0.62	5.7E-03	7.4E-04	214425	cartilage intermediate layer protein, nucleotide pyrophosphohydrolase
Cisd3	3.40	1.77	1.7E-12	3.6E-14	217149	CDGSH iron sulfur domain 3
Cish	2.64	1.40	1.1E-09	3.2E-11	12700	cytokine inducible SH2-containing protein
Cited1	1.72	0.78	6.7E-04	6.0E-05	12705	Cbp/p300-interacting transactivator with Glu/Asp-rich carboxy-terminal domain 1
Ciz1	1.54	0.62	2.4E-05	1.4E-06	68379	CDKN1A interacting zinc finger protein 1
Ckm	7.11	2.83	3.3E-35	6.0E-38	12715	creatine kinase, muscle
Ckmt2	9.88	3.30	7.9E-41	1.1E-43	76722	creatine kinase, mitochondrial 2
Clcf1	0.52	-0.94	4.3E-03	5.2E-04	56708	cardiotrophin-like cytokine factor 1
Clcn1	3.89	1.96	6.2E-15	9.5E-17	12723	chloride channel 1

Cldn1	0.38	-1.38	1.8E-06	8.5E-08	12737	claudin 1
Cldn15	5.01	2.32	1.6E-11	3.8E-13	60363	claudin 15
Cldn4	0.19	-2.38	1.3E-03	1.3E-04	12740	claudin 4
Cldn5	1.78	0.83	2.8E-08	9.6E-10	12741	claudin 5
Clec16a	1.53	0.62	6.7E-05	4.6E-06	74374	C-type lectin domain family 16, member A
Clec2d	2.40	1.26	1.2E-05	6.8E-07	93694	C-type lectin domain family 2, member d
Clec2l	0.50	-1.00	4.6E-03	5.6E-04	665180	C-type lectin domain family, member L
Clec3b	2.62	1.39	1.3E-21	1.1E-23	21922	C-type lectin domain family 3, member b
Clic4	1.50	0.59	4.1E-05	2.6E-06	29876	chloride intracellular channel 4 (mitochondrial)
Clic5	3.35	1.75	4.3E-15	6.6E-17	224796	chloride intracellular channel 5
Clpx	1.52	0.60	2.9E-05	1.8E-06	270166	caseinolytic peptidase X
Clu	0.49	-1.02	8.6E-05	6.0E-06	12759	clusterin
Cluh	1.74	0.80	3.1E-07	1.3E-08	74148	clustered mitochondria (cluA/CLU1) homolog
Clybl	1.97	0.98	1.5E-06	6.7E-08	69634	citrate lyase beta like
Cmbl	6.09	2.61	9.0E-36	1.5E-38	69574	carboxymethylenebutenolidase-like
Cmya5	7.37	2.88	2.3E-29	9.3E-32	76469	cardiomyopathy associated 5
Cnih2	0.41	-1.29	2.7E-07	1.1E-08	12794	cornichon homolog 2
Cnn1	3.06	1.61	5.1E-08	1.9E-09	12797	calponin 1
Cnp	1.94	0.96	1.3E-09	3.9E-11	12799	2',3'-cyclic nucleotide 3' phosphodiesterase
Cntn4	0.49	-1.02	4.6E-03	5.7E-04	269784	contactin 4
Cntnap4	0.42	-1.24	2.6E-05	1.6E-06	170571	contactin associated protein-like 4
Cobl	1.74	0.80	3.2E-08	1.1E-09	12808	cordon-bleu
Cog4	1.55	0.64	6.0E-06	3.2E-07	102339	component of oligomeric golgi complex 4
Col15a1	1.55	0.63	3.4E-05	2.2E-06	12819	collagen, type XV, alpha 1
Col23a1	1.55	0.63	2.6E-05	1.6E-06	237759	collagen, type XXIII, alpha 1
Col26a1	0.62	-0.69	2.7E-04	2.1E-05	140709	collagen, type XXVI, alpha 1
Col28a1	3.34	1.74	6.2E-07	2.7E-08	213945	collagen, type XXVIII, alpha 1
Col4a1	1.63	0.71	9.0E-07	4.0E-08	12826	collagen, type IV, alpha 1
Col4a2	1.54	0.62	3.0E-05	1.9E-06	12827	collagen, type IV, alpha 2
Col4a6	0.37	-1.42	6.6E-04	5.9E-05	94216	collagen, type IV, alpha 6
Col5a3	2.50	1.32	2.7E-21	2.3E-23	53867	collagen, type V, alpha 3
Col6a6	1.72	0.78	3.3E-06	1.6E-07	245026	collagen, type VI, alpha 6
Col8a2	0.43	-1.22	1.6E-06	7.2E-08	329941	collagen, type VIII, alpha 2
Col9a2	0.41	-1.27	4.3E-03	5.3E-04	12840	collagen, type IX, alpha 2
Coq10a	2.88	1.52	1.3E-16	1.7E-18	210582	coenzyme Q10 homolog A
Coq3	1.78	0.83	5.1E-05	3.4E-06	230027	coenzyme Q3 homolog, methyltransferase
Coq5	2.25	1.17	9.2E-16	1.3E-17	52064	coenzyme Q5 homolog, methyltransferase
Coq7	1.65	0.72	2.3E-04	1.8E-05	12850	demethyl-Q 7
Coq9	2.74	1.45	4.7E-25	3.1E-27	67914	coenzyme Q9 homolog
Coro2a	0.59	-0.77	5.4E-03	6.9E-04	107684	coronin, actin binding protein 2A
Coro6	3.44	1.78	6.8E-19	6.9E-21	216961	coronin 6
Cox14	1.72	0.78	8.1E-07	3.6E-08	66379	cytochrome c oxidase assembly protein 14
Cox15	1.56	0.65	1.7E-06	8.1E-08	226139	COX15 homolog, cytochrome c oxidase assembly protein
Cox17	1.85	0.89	4.5E-05	3.0E-06	12856	cytochrome c oxidase, subunit XVII assembly protein homolog
Cox4i1	1.88	0.91	8.0E-09	2.6E-10	12857	cytochrome c oxidase subunit IV isoform 1
Cox5a	2.18	1.12	2.3E-15	3.4E-17	12858	cytochrome c oxidase, subunit Va
Cox5b	1.80	0.85	1.9E-03	1.9E-04	12859	cytochrome c oxidase, subunit Vb
Cox6a2	3.65	1.87	6.2E-28	2.9E-30	12862	cytochrome c oxidase, subunit VI a, polypeptide 2
Cox6b1	1.82	0.87	5.4E-09	1.7E-10	110323	cytochrome c oxidase, subunit VIb polypeptide 1
Cox6c	1.57	0.65	3.6E-05	2.3E-06	12864	cytochrome c oxidase, subunit VIc
Cox7a1	12.91	3.69	6.7E-26	4.0E-28	12865	cytochrome c oxidase, subunit VIIa 1
Cox7a2	2.02	1.01	5.5E-07	2.3E-08	12866	cytochrome c oxidase, subunit VIIa 2
Cox7b	1.74	0.80	3.2E-08	1.1E-09	66142	cytochrome c oxidase subunit VIIb
Cox8b	4.37	2.13	2.2E-23	1.6E-25	12869	cytochrome c oxidase, subunit VIIIb
Cpa4	3.79	1.92	1.5E-03	1.5E-04	71791	carboxypeptidase A4
Cpeb3	2.64	1.40	1.0E-07	4.0E-09	208922	cytoplasmic polyadenylation element binding protein 3
Cpeb4	1.77	0.82	1.4E-04	9.9E-06	67579	cytoplasmic polyadenylation element binding protein 4
Cpm	0.57	-0.82	3.5E-08	1.2E-09	70574	carboxypeptidase M
Cpne5	0.36	-1.47	8.3E-08	3.1E-09	240058	copine V
Cpped1	1.71	0.78	3.6E-08	1.3E-09	223978	calcineurin-like phosphoesterase domain containing 1
Cpt1b	8.24	3.04	2.3E-53	1.1E-56	12895	carnitine palmitoyltransferase 1b, muscle
Cpt2	1.73	0.79	2.1E-07	8.2E-09	12896	carnitine palmitoyltransferase 2
Cpxm2	0.42	-1.24	6.9E-03	9.1E-04	55987	carboxypeptidase X 2 (M14 family)
Crabp1	0.40	-1.33	4.3E-13	8.3E-15	12903	cellular retinoic acid binding protein I
Crabp2	0.54	-0.88	1.5E-03	1.5E-04	12904	cellular retinoic acid binding protein II
Crat	3.80	1.92	5.2E-31	1.8E-33	12908	carnitine acetyltransferase
Creld1	1.61	0.69	1.5E-05	8.6E-07	171508	cysteine-rich with EGF-like domains 1
Creld2	0.52	-0.94	3.9E-09	1.2E-10	76737	cysteine-rich with EGF-like domains 2
Crmp1	0.55	-0.85	1.5E-03	1.6E-04	12933	collapsin response mediator protein 1
Cryab	2.03	1.02	5.1E-08	1.9E-09	12955	crystallin, alpha B
Cryl1	0.55	-0.87	2.2E-04	1.7E-05	68631	crystallin, lambda 1
Cs	1.92	0.94	6.5E-11	1.6E-12	12974	citrate synthase
Csf2rb2	2.35	1.23	8.8E-08	3.3E-09	12984	colony stimulating factor 2 receptor, beta 2, low-affinity (granulocyte-macrophage)
Csl	1.76	0.82	7.0E-04	6.3E-05	71832	citrate synthase like
Cspg5	0.42	-1.23	6.2E-06	3.3E-07	29873	chondroitin sulfate proteoglycan 5
Csrp3	2.96	1.56	1.7E-13	3.1E-15	13009	cysteine and glycine-rich protein 3
Ctla2a	1.74	0.80	2.2E-03	2.4E-04	13024	cytotoxic T lymphocyte-associated protein 2 alpha
Ctnna3	3.68	1.88	1.1E-12	2.3E-14	216033	catenin (cadherin associated protein), alpha 3
Ctnnbip1	1.75	0.81	1.1E-08	3.6E-10	67087	catenin beta interacting protein 1
Cttnbp2	0.47	-1.08	9.3E-06	5.1E-07	30785	cortactin binding protein 2
Cux2	0.51	-0.97	2.8E-04	2.2E-05	13048	cut-like homeobox 2
Cwh43	0.25	-2.01	1.7E-03	1.7E-04	231293	cell wall biogenesis 43 C-terminal homolog
Cxcl14	0.28	-1.82	1.6E-08	5.2E-10	57266	chemokine (C-X-C motif) ligand 14
Cyb5d2	1.65	0.72	4.6E-03	5.7E-04	192986	cytochrome b5 domain containing 2
Cyb5r1	1.87	0.90	3.4E-11	8.2E-13	72017	cytochrome b5 reductase 1
Cyc1	2.18	1.13	1.0E-15	1.5E-17	66445	cytochrome c-1
Cycs	2.16	1.11	5.8E-05	3.9E-06	13063	cytochrome c, somatic
Cyp1b1	2.42	1.27	1.6E-03	1.6E-04	13078	cytochrome P450, family 1, subfamily b, polypeptide 1
Cyp27a1	3.01	1.59	7.6E-11	1.9E-12	104086	cytochrome P450, family 27, subfamily a, polypeptide 1
Cyp2d22	1.82	0.87	7.3E-05	5.0E-06	56448	cytochrome P450, family 2, subfamily d, polypeptide 22
Cyp2w1	0.17	-2.60	6.5E-03	8.6E-04	545817	cytochrome P450, family 2, subfamily w, polypeptide 1
Cyp51	0.60	-0.74	2.6E-08	9.2E-10	13121	cytochrome P450, family 51
Cystm1	6.47	2.69	2.0E-17	2.4E-19	66060	cysteine-rich transmembrane module containing 1
D030025P21Rik	0.47	-1.09	4.8E-04	4.1E-05	100303738	RIKEN cDNA D030025P21 gene
D030028A08Rik	0.48	-1.06	6.7E-05	4.6E-06	319371	RIKEN cDNA D030028A08 gene
D10Jhu81e	1.69	0.75	5.5E-08	2.0E-09	28295	DNA segment, Chr 10, Johns Hopkins University 81 expressed
D330045A20Rik	1.98	0.98	2.1E-03	2.2E-04	102871	RIKEN cDNA D330045A20 gene
D630045J12Rik	0.61	-0.72	1.5E-04	1.1E-05	330286	RIKEN cDNA D630045J12 gene
D830015G02Rik	1.89	0.92	5.3E-07	2.3E-08	791403	RIKEN cDNA D830015G02 gene
Dag1	1.66	0.74	2.2E-06	1.0E-07	13138	dystroglycan 1
Dand5	1.69	0.75	6.0E-03	7.7E-04	23863	DAN domain family, member 5
Dbndd1	0.58	-0.79	3.2E-03	3.7E-04	72185	dysbindin (dystrobrevin binding protein 1) domain containing 1
Dbndd2	1.63	0.70	6.8E-03	9.0E-04	52840	dysbindin (dystrobrevin binding protein 1) domain containing 2
Dbnl	0.63	-0.67	7.7E-05	5.3E-06	13169	drebrin-like
Dbp	1.70	0.77	7.1E-04	6.4E-05	13170	D site albumin promoter binding protein
Dcaf6	1.74	0.80	9.8E-10	2.8E-11	74106	DDB1 and CUL4 associated factor 6
Dcc	0.38	-1.38	2.4E-05	1.5E-06	13176	deleted in colorectal carcinoma
Dct	0.49	-1.02	3.0E-03	3.4E-04	13190	dopachrome tautomerase
Dctd	0.64	-0.65	7.1E-04	6.5E-05	320685	dCMP deaminase
Ddc	2.16	1.11	2.9E-09	8.7E-11	13195	dopa decarboxylase
Ddit4l	2.12	1.08	1.1E-08	3.4E-10	73284	DNA-damage-inducible transcript 4-like
Ddo	10.85	3.44	2.8E-33	7.3E-36	70503	D-aspartate oxidase
Ddx11	0.61	-0.72	3.6E-04	2.9E-05	320209	DEAD/H (Asp-Glu-Ala-Asp/His) box helicase 11
Ddx25	0.41	-1.30	1.2E-05	6.7E-07	30959	DEAD (Asp-Glu-Ala-Asp) box polypeptide 25
Deb1	1.68	0.75	4.5E-04	3.8E-05	26901	differentially expressed in B16F10 1
Decr1	1.95	0.96	4.3E-11	1.0E-12	67460	2,4-dienoyl CoA reductase 1, mitochondrial
Dennd4b	1.65	0.73	4.6E-07	2.0E-08	229541	DENN/MADD domain containing 4B
Deptor	2.06	1.04	1.8E-07	7.2E-09	97998	DEP domain containing MTOR-interacting protein
Des	3.46	1.79	9.1E-21	7.9E-23	13346	desmin
Dhcr24	0.55	-0.88	3.4E-07	1.4E-08	74754	24-dehydrocholesterol reductase
Dhdh	2.36	1.24	7.7E-07	3.4E-08	71755	dihydrodiol dehydrogenase (dimeric)
Dhrs7c	4.09	2.03	2.6E-26	1.5E-28	68460	dehydrogenase/reductase (SDR family) member 7C
Dio2	1.96	0.97	6.2E-04	5.4E-05	13371	deiodinase, iodothyronine, type II
Dkk2	0.60	-0.75	6.0E-04	5.3E-05	56811	dickkopf homolog 2
Dlat	2.35	1.23	5.0E-19	5.1E-21	235339	dihydrolipoamide S-acetyltransferase (E2 component of pyruvate dehydrogenase complex)
Dld	2.07	1.05	2.7E-12	5.8E-14	13382	dihydrolipoamide dehydrogenase
Dlst	1.79	0.84	3.8E-09	1.2E-10	78920	dihydrolipoamide S-succinyltransferase (E2 component of 2-oxo-glutarate complex)
Dlx5	0.43	-1.21	1.8E-05	1.1E-06	13395	distal-less homeobox 5
Dmd	2.65	1.41	1.3E-19	1.2E-21	13405	dystrophin, muscular dystrophy
Dmpk	2.20	1.14	2.2E-12	4.7E-14	13400	dystrophia myotonica-protein kinase
Dnaaf2	0.66	-0.60	5.2E-03	6.5E-04	109065	dynein, axonemal assembly factor 2

Dnah11	0.59	-0.77	6.6E-03	8.7E-04	13411	dynein, axonemal, heavy chain 11
Dnaic1	7.18	2.84	3.1E-24	2.1E-26	68922	dynein, axonemal, intermediate chain 1
Dnaja3	1.56	0.65	5.3E-06	2.8E-07	83945	DnaJ (Hsp40) homolog, subfamily A, member 3
Dnaja4	1.84	0.88	1.7E-07	6.8E-09	58233	DnaJ (Hsp40) homolog, subfamily A, member 4
Dnajb11	0.66	-0.60	1.1E-05	6.0E-07	67838	DnaJ (Hsp40) homolog, subfamily B, member 11
Dnajb4	1.99	0.99	5.3E-11	1.3E-12	67035	DnaJ (Hsp40) homolog, subfamily B, member 4
Dnajb5	1.96	0.97	9.6E-11	2.4E-12	56323	DnaJ (Hsp40) homolog, subfamily B, member 5
Dnajc12	2.35	1.23	6.2E-07	2.7E-08	30045	DnaJ (Hsp40) homolog, subfamily C, member 12
Dnajc15	1.54	0.62	2.9E-03	3.2E-04	66148	DnaJ (Hsp40) homolog, subfamily C, member 15
Dnajc19	1.63	0.71	6.1E-03	8.0E-04	67713	DnaJ (Hsp40) homolog, subfamily C, member 19
Dnase1l1	2.44	1.29	2.6E-12	5.5E-14	69537	deoxyribonuclease 1-like 1
Dner	0.45	-1.17	1.3E-04	9.5E-06	227325	delta/notch-like EGF-related receptor
Dnm3os	0.61	-0.72	1.5E-06	7.0E-08	474332	dynamin 3, opposite strand
Dnmt1	0.64	-0.63	7.9E-06	4.3E-07	13433	DNA methyltransferase (cytosine-5) 1
Dnph1	0.53	-0.92	2.1E-04	1.6E-05	381101	2'-deoxynucleoside 5'-phosphate N-hydrolase 1
Doc2b	0.47	-1.10	3.6E-04	3.0E-05	13447	double C2, beta
Dos	0.59	-0.77	5.2E-03	6.5E-04	100503659	downstream of Stk11
Dpep1	1.70	0.76	4.3E-04	3.6E-05	13479	dipeptidase 1 (renal)
Dpf3	2.87	1.52	1.3E-07	5.1E-09	70127	D4, zinc and double PHD fingers, family 3
Dpysl4	0.60	-0.74	1.7E-03	1.7E-04	26757	dihydropyrimidinase-like 4
Dram2	1.62	0.70	2.0E-05	1.2E-06	67171	DNA-damage regulated autophagy modulator 2
Dsc2	0.23	-2.12	5.3E-19	5.3E-21	13506	desmocollin 2
Dsg2	0.22	-2.20	1.2E-04	8.7E-06	13511	desmoglein 2
Dtna	4.07	2.03	1.9E-26	1.1E-28	13527	dystrobrevin alpha
Dtnb	0.62	-0.69	2.3E-03	2.5E-04	13528	dystrobrevin, beta
Dupd1	8.81	3.14	8.3E-18	9.3E-20	435391	dual specificity phosphatase and pro isomerase domain containing 1
Dusp1	1.57	0.65	3.1E-04	2.5E-05	19252	dual specificity phosphatase 1
Dusp10	1.61	0.69	2.5E-05	1.5E-06	63953	dual specificity phosphatase 10
Dusp13	5.77	2.53	2.7E-20	2.4E-22	27389	dual specificity phosphatase 13
Dusp2	1.74	0.80	1.6E-03	1.7E-04	13537	dual specificity phosphatase 2
Dusp22	1.71	0.78	2.1E-06	9.9E-08	105352	dual specificity phosphatase 22
Dusp23	2.54	1.35	3.4E-11	8.1E-13	68440	dual specificity phosphatase 23
Dusp26	5.59	2.48	5.9E-27	3.0E-29	66959	dual specificity phosphatase 26 (putative)
Dusp27	2.23	1.16	1.4E-09	4.0E-11	240892	dual specificity phosphatase 27 (putative)
Dusp28	1.86	0.90	8.6E-05	6.1E-06	67446	dual specificity phosphatase 28
Dusp3	1.83	0.87	2.4E-08	8.4E-10	72349	dual specificity phosphatase 3 (vaccinia virus phosphatase VH1-related)
Dusp7	0.62	-0.69	3.6E-04	2.9E-05	235584	dual specificity phosphatase 7
Dusp8	1.51	0.59	4.8E-03	6.0E-04	18218	dual specificity phosphatase 8
Dusp9	0.34	-1.54	2.0E-04	1.5E-05	75590	dual specificity phosphatase 9
Dynap	0.01	-6.21	4.9E-25	3.2E-27	75577	dynactin associated protein
Dyrk1b	2.28	1.19	5.4E-15	8.3E-17	13549	dual-specificity tyrosine-(Y)-phosphorylation regulated kinase 1b
Dyrk2	1.54	0.62	9.2E-05	6.5E-06	69181	dual-specificity tyrosine-(Y)-phosphorylation regulated kinase 2
Dzip1	0.62	-0.69	1.4E-04	1.0E-05	66573	DAZ interacting protein 1
E130012A19Rik	0.48	-1.07	4.6E-04	3.8E-05	103551	RIKEN cDNA E130012A19 gene
E2f6	1.59	0.67	1.1E-06	5.1E-08	50496	E2F transcription factor 6
Eci1	2.31	1.21	2.0E-13	3.7E-15	13177	enoyl-Coenzyme A delta isomerase 1
Eci2	1.52	0.61	2.1E-04	1.7E-05	23986	enoyl-Coenzyme A delta isomerase 2
Ecm1	1.98	0.99	2.5E-03	2.7E-04	13601	extracellular matrix protein 1
Eda2r	1.71	0.78	3.8E-03	4.5E-04	245527	ectodysplasin A2 receptor
Ednrb	1.57	0.65	1.5E-04	1.1E-05	13618	endothelin receptor type B
Eef1a2	19.88	4.31	9.1E-66	2.7E-69	13628	eukaryotic translation elongation factor 1 alpha 2
Eepd1	2.02	1.02	7.2E-08	2.7E-09	67484	endonuclease/exonuclease/phosphatase family domain containing 1
Efcab2	1.79	0.84	1.0E-03	9.6E-05	68226	EF-hand calcium binding domain 2
Efcab6	5.77	2.53	2.0E-14	3.3E-16	77627	EF-hand calcium binding domain 6
Efemp1	1.85	0.89	4.2E-03	5.0E-04	216616	epidermal growth factor-containing fibulin-like extracellular matrix protein 1
Efna2	0.59	-0.77	1.1E-03	1.0E-04	13637	ephrin A2
Efna3	0.22	-2.19	4.3E-05	2.8E-06	13638	ephrin A3
Efna4	0.44	-1.18	2.7E-06	1.3E-07	13639	ephrin A4
Efna5	0.53	-0.92	4.5E-06	2.3E-07	13640	ephrin A5
Efnb3	0.38	-1.41	3.5E-09	1.1E-10	13643	ephrin B3
Egf	3.65	1.87	1.8E-13	3.3E-15	13645	epidermal growth factor
Egfbp2	5.14	2.36	2.2E-14	3.5E-16	13647	epidermal growth factor binding protein type B
Egfl6	0.59	-0.76	4.0E-06	2.0E-07	54156	EGF-like-domain, multiple 6
Egln3	2.05	1.03	1.2E-09	3.3E-11	112407	EGL nine homolog 3
Ehbp1l1	2.08	1.06	1.7E-09	5.1E-11	114601	EH domain binding protein 1-like 1
Ehd4	1.76	0.81	1.5E-06	6.7E-08	98878	EH-domain containing 4
Ehhadh	1.84	0.88	1.5E-04	1.1E-05	74147	enoyl-Coenzyme A, hydratase/3-hydroxyacyl Coenzyme A dehydrogenase
Eif4ebp1	1.75	0.81	3.9E-08	1.4E-09	13685	eukaryotic translation initiation factor 4E binding protein 1
Elf5	0.04	-4.78	2.1E-14	3.3E-16	13711	E74-like factor 5
Elfn1	0.39	-1.35	2.2E-06	1.0E-07	243312	leucine rich repeat and fibronectin type III, extracellular 1
Elovl6	0.60	-0.73	1.1E-06	5.1E-08	170439	ELOVL family member 6, elongation of long chain fatty acids
Eltd1	1.95	0.96	2.0E-06	9.4E-08	170757	EGF, latrophilin seven transmembrane domain containing 1
Emb	0.60	-0.74	3.1E-03	3.5E-04	13723	embigin
Emc2	1.63	0.71	2.2E-07	8.8E-09	66736	ER membrane protein complex subunit 2
Emc9	2.69	1.43	1.6E-08	5.5E-10	85308	ER membrane protein complex subunit 9
Eme1	0.66	-0.60	1.7E-03	1.7E-04	268465	essential meiotic endonuclease 1 homolog 1
Emid1	0.62	-0.70	5.3E-03	6.7E-04	140703	EMI domain containing 1
Emilin3	0.57	-0.82	2.9E-05	1.8E-06	280635	elastin microfibril interfacer 3
Enho	2.84	1.51	1.3E-12	2.6E-14	69638	energy homeostasis associated
Enkd1	0.59	-0.77	2.4E-03	2.6E-04	102124	enkurin domain containing 1
Eno3	5.91	2.56	3.3E-35	5.9E-38	13808	enolase 3, beta muscle
Enpp2	0.63	-0.66	7.1E-03	9.5E-04	18606	ectonucleotide pyrophosphatase/phosphodiesterase 2
Enpp4	1.64	0.72	2.6E-03	2.9E-04	224794	ectonucleotide pyrophosphatase/phosphodiesterase 4
Entpd1	1.71	0.77	9.7E-08	3.7E-09	12495	ectonucleoside triphosphate diphosphohydrolase 1
Entpd2	3.39	1.76	7.5E-22	6.1E-24	12496	ectonucleoside triphosphate diphosphohydrolase 2
Epb4.1l5	0.53	-0.91	1.1E-03	1.1E-04	226352	erythrocyte protein band 4.1-like 5
Epdr1	1.81	0.85	2.0E-06	9.3E-08	105298	ependymin related protein 1 (zebrafish)
Epha1	0.25	-1.99	2.2E-04	1.7E-05	13835	Eph receptor A1
Epha7	0.52	-0.94	3.8E-04	3.1E-05	13841	Eph receptor A7
Epha8	0.13	-2.89	2.3E-14	3.8E-16	13842	Eph receptor A8
Ephb1	0.60	-0.75	5.1E-03	6.4E-04	270190	Eph receptor B1
Ephb2	0.64	-0.64	4.1E-07	1.7E-08	13844	Eph receptor B2
Ephx1	2.39	1.26	1.1E-13	1.9E-15	13849	epoxide hydrolase 1, microsomal
Ephx2	2.60	1.38	7.6E-05	5.3E-06	13850	epoxide hydrolase 2, cytoplasmic
Epm2a	2.90	1.54	1.1E-08	3.4E-10	13853	epilepsy, progressive myoclonic epilepsy, type 2 gene alpha
Eps8l2	0.33	-1.61	3.4E-03	4.0E-04	98845	EPS8-like 2
Erc2	0.47	-1.08	8.1E-04	7.5E-05	238988	ELKS/RAB6-interacting/CAST family member 2
Erdr1	0.18	-2.44	9.4E-04	8.9E-05	170942	erythroid differentiation regulator 1
Esd	1.57	0.65	4.6E-04	3.9E-05	13885	esterase D/formylglutathione hydrolase
Esr1	2.35	1.23	3.3E-04	2.7E-05	13982	estrogen receptor 1 (alpha)
Esrra	2.02	1.02	1.2E-12	2.5E-14	26379	estrogen related receptor, alpha
Esrrg	3.07	1.62	2.6E-08	9.2E-10	26381	estrogen-related receptor gamma
Etfa	2.02	1.01	1.2E-10	3.1E-12	110842	electron transferring flavoprotein, alpha polypeptide
Etfb	2.10	1.07	1.0E-13	1.8E-15	110826	electron transferring flavoprotein, beta polypeptide
Etfdh	2.05	1.03	2.1E-12	4.4E-14	66841	electron transferring flavoprotein, dehydrogenase
Evpl	0.18	-2.43	5.8E-04	5.1E-05	14027	envoplakin
Exo1	0.62	-0.68	7.4E-04	6.7E-05	26909	exonuclease 1
Exoc6	1.94	0.95	1.3E-06	5.9E-08	107371	exocyst complex component 6
Exog	0.59	-0.76	2.8E-04	2.2E-05	208194	endo/exonuclease (5'-3'), endonuclease G-like
Exosc6	0.64	-0.65	6.8E-03	9.0E-04	72544	exosome component 6
Extl1	0.48	-1.05	3.9E-04	3.2E-05	56219	exostoses (multiple)-like 1
Eya4	1.75	0.81	1.6E-05	9.0E-07	14051	eyes absent 4 homolog
Ezr	0.46	-1.13	3.5E-04	2.8E-05	22350	ezrin
F2rl1	0.56	-0.84	4.8E-04	4.0E-05	14063	coagulation factor II (thrombin) receptor-like 1
Fabp3	1.97	0.98	4.1E-04	3.4E-05	14077	fatty acid binding protein 3, muscle and heart
Fabp4	7.94	2.99	1.8E-26	9.8E-29	11770	fatty acid binding protein 4, adipocyte
Fahd1	1.63	0.71	1.5E-03	1.5E-04	68636	fumarylacetoacetate hydrolase domain containing 1
Fam101a	0.43	-1.21	4.9E-04	4.2E-05	73121	family with sequence similarity 101, member A
Fam120c	1.67	0.74	3.5E-06	1.8E-07	207375	family with sequence similarity 120C
Fam129b	0.40	-1.32	1.1E-08	3.7E-10	227737	family with sequence similarity 129, member B
Fam131a	4.88	2.29	7.5E-45	8.1E-48	78408	family with sequence similarity 131, member A
Fam134b	5.09	2.35	3.6E-30	1.4E-32	66270	family with sequence similarity 134, member B
Fam136a	1.74	0.80	4.4E-09	1.4E-10	66488	family with sequence similarity 136, member A
Fam13a	2.28	1.19	2.9E-09	8.8E-11	58909	family with sequence similarity 13, member A
Fam151a	2.28	1.19	9.6E-08	3.7E-09	230579	family with sequence similarity 151, member A
Fam160a1	2.23	1.16	1.0E-15	1.4E-17	229488	family with sequence similarity 160, member A1
Fam167a	0.66	-0.60	1.9E-03	2.0E-04	219148	family with sequence similarity 167, member A
Fam174b	0.67	-0.59	2.9E-03	3.3E-04	100038347	family with sequence similarity 174, member B
Fam189a1	0.34	-1.54	3.8E-07	1.6E-08	70638	family with sequence similarity 189, member A1
Fam189a2	3.15	1.66	2.3E-21	1.9E-23	381217	family with sequence similarity 189, member A2
Fam195a	3.01	1.59	1.2E-10	3.0E-12	68241	family with sequence similarity 195, member A

Fam210a	1.72	0.78	5.4E-07	2.3E-08	108654	family with sequence similarity 210, member A
Fam212b	1.56	0.64	4.3E-04	3.6E-05	109050	family with sequence similarity 212, member B
Fam213b	1.62	0.70	2.3E-04	1.8E-05	66469	family with sequence similarity 213, member B
Fam214a	1.81	0.86	1.6E-03	1.6E-04	235493	family with sequence similarity 214, member A
Fam49b	1.90	0.92	7.1E-10	2.0E-11	223601	family with sequence similarity 49, member B
Fam57a	0.44	-1.17	4.5E-04	3.8E-05	116972	family with sequence similarity 57, member A
Fam69c	1.82	0.87	1.4E-03	1.5E-04	240479	family with sequence similarity 69, member C
Fam83h	0.26	-1.94	3.8E-03	4.5E-04	105732	family with sequence similarity 83, member H
Fam84a	0.28	-1.86	9.8E-07	4.4E-08	105005	family with sequence similarity 84, member A
Fam96b	1.60	0.68	3.0E-05	1.8E-06	68523	family with sequence similarity 96, member B
Fanca	0.63	-0.67	4.8E-04	4.1E-05	14087	Fanconi anemia, complementation group A
Fancb	0.58	-0.78	4.0E-03	4.8E-04	237211	Fanconi anemia, complementation group B
Fap	1.59	0.67	7.6E-06	4.1E-07	14089	fibroblast activation protein
Fasn	0.59	-0.76	2.9E-06	1.4E-07	14104	fatty acid synthase
Fastkd1	1.72	0.78	2.0E-04	1.5E-05	320720	FAST kinase domains 1
Fbp2	5.30	2.41	1.3E-20	1.2E-22	14120	fructose bisphosphatase 2
Fbxl19	0.53	-0.91	2.1E-08	7.2E-10	233902	F-box and leucine-rich repeat protein 19
Fbxl22	2.71	1.44	6.3E-15	9.7E-17	74165	F-box and leucine-rich repeat protein 22
Fbxo31	2.06	1.04	2.8E-16	3.7E-18	76454	F-box protein 31
Fbxo32	1.99	0.99	7.1E-07	3.1E-08	67731	F-box protein 32
Fbxo40	3.48	1.80	2.8E-12	6.1E-14	207215	F-box protein 40
Fcgr2b	2.73	1.45	1.2E-09	3.5E-11	14130	Fc receptor, IgG, low affinity IIb
Fcna	2.65	1.40	4.0E-07	1.7E-08	14133	ficolin A
Fem1a	2.39	1.26	1.3E-17	1.6E-19	14154	feminization 1 homolog a
Fem1c	1.54	0.62	4.7E-06	2.4E-07	240263	fem-1 homolog c
Fermt2	1.97	0.98	4.5E-11	1.1E-12	218952	fermitin family homolog 2
Fetub	0.09	-3.45	1.9E-12	3.9E-14	59083	fetuin beta
Fgf1	1.75	0.81	5.4E-04	4.7E-05	14164	fibroblast growth factor 1
Fgf13	1.56	0.64	3.0E-04	2.4E-05	14168	fibroblast growth factor 13
Fgf16	2.04	1.03	7.3E-04	6.6E-05	80903	fibroblast growth factor 16
Fgfr2	0.50	-0.99	6.1E-06	3.2E-07	14183	fibroblast growth factor receptor 2
Fgfr3	0.42	-1.24	3.2E-06	1.6E-07	14184	fibroblast growth factor receptor 3
Fgfrl1	2.48	1.31	6.1E-19	6.2E-21	116701	fibroblast growth factor receptor-like 1
Fggy	1.73	0.79	1.3E-03	1.2E-04	75578	FGGY carbohydrate kinase domain containing
Fgl2	1.80	0.85	3.2E-03	3.6E-04	14190	fibrinogen-like protein 2
Fh1	1.82	0.86	1.3E-10	3.5E-12	14194	fumarate hydratase 1
Fhl1	4.01	2.00	7.9E-26	4.8E-28	14199	four and a half LIM domains 1
Fhl3	1.91	0.93	2.8E-11	6.5E-13	14201	four and a half LIM domains 3
Filip1	2.01	1.01	1.1E-09	3.2E-11	70598	filamin A interacting protein 1
Fitm1	3.18	1.67	3.8E-28	1.7E-30	68680	fat storage-inducing transmembrane protein 1
Fjx1	0.61	-0.72	8.3E-04	7.6E-05	14221	four jointed box 1
Fkbp3	1.53	0.62	7.7E-05	5.3E-06	30795	FK506 binding protein 3
Flnc	1.82	0.87	8.2E-07	3.6E-08	68794	filamin C, gamma
Flot1	1.69	0.75	3.4E-07	1.4E-08	14251	flotillin 1
Flot2	1.62	0.70	2.2E-07	8.6E-09	14252	flotillin 2
Flrt3	0.57	-0.82	3.6E-05	2.3E-06	71436	fibronectin leucine rich transmembrane protein 3
Flt1	1.69	0.75	2.7E-08	9.3E-10	14254	FMS-like tyrosine kinase 1
Fmo1	1.95	0.96	2.7E-11	6.5E-13	14261	flavin containing monooxygenase 1
Fn3k	4.59	2.20	7.6E-18	8.5E-20	63828	fructosamine 3 kinase
Fndc3c1	1.72	0.78	4.4E-05	2.9E-06	333564	fibronectin type III domain containing 3C1
Fndc4	0.61	-0.72	7.7E-06	4.2E-07	64339	fibronectin type III domain containing 4
Folr2	2.07	1.05	2.4E-04	1.8E-05	14276	folate receptor 2 (fetal)
Foxp4	0.58	-0.78	7.5E-07	3.3E-08	74123	forkhead box P4
Foxq1	0.02	-5.45	1.5E-20	1.3E-22	15220	forkhead box Q1
Foxred2	0.56	-0.85	1.1E-03	1.1E-04	239554	FAD-dependent oxidoreductase domain containing 2
Frem1	0.66	-0.60	3.8E-03	4.5E-04	329872	Fras1 related extracellular matrix protein 1
Fry	2.14	1.10	1.1E-14	1.7E-16	320365	furry homolog
Frzb	1.60	0.67	4.9E-05	3.2E-06	20378	frizzled-related protein
Fsd1l	1.66	0.73	2.7E-04	2.1E-05	319636	fibronectin type III and SPRY domain containing 1-like
Fsd2	4.39	2.13	1.7E-26	9.4E-29	244091	fibronectin type III and SPRY domain containing 2
Fth1	1.68	0.75	3.4E-08	1.2E-09	14319	ferritin heavy chain 1
Fundc2	1.72	0.78	6.0E-08	2.2E-09	67391	FUN14 domain containing 2
Fxr1	1.76	0.82	9.1E-07	4.0E-08	14359	fragile X mental retardation gene 1, autosomal homolog
Fxyd1	3.23	1.69	5.5E-27	2.8E-29	56188	FXYD domain-containing ion transport regulator 1
Fyco1	2.14	1.10	4.9E-16	6.5E-18	17281	FYVE and coiled-coil domain containing 1
Fzd3	0.60	-0.73	8.3E-05	5.8E-06	14365	frizzled homolog 3
G0s2	2.75	1.46	8.0E-15	1.2E-16	14373	G0/G1 switch gene 2
G630090E17Rik	2.49	1.31	1.5E-05	8.5E-07	100041085	RIKEN cDNA G630090E17 gene
G6pdx	0.55	-0.85	7.3E-05	5.1E-06	14381	glucose-6-phosphate dehydrogenase X-linked
Gadd45a	1.64	0.71	2.6E-03	2.9E-04	13197	growth arrest and DNA-damage-inducible 45 alpha
Gadd45gip1	1.57	0.65	2.3E-05	1.4E-06	102060	growth arrest and DNA-damage-inducible, gamma interacting protein 1
Gadl1	2.77	1.47	2.2E-07	8.8E-09	73748	glutamate decarboxylase-like 1
Gale	0.58	-0.78	4.2E-04	3.5E-05	74246	galactose-4-epimerase, UDP
Galm	1.79	0.84	7.4E-06	4.0E-07	319625	galactose mutarotase
Galnt3	0.53	-0.93	7.0E-03	9.3E-04	14425	UDP-N-acetyl-alpha-D-galactosamine:polypeptide N-acetylgalactosaminyltransferase 3
Galt	1.79	0.84	1.4E-05	7.7E-07	14430	galactose-1-phosphate uridyl transferase
Gamt	2.62	1.39	1.3E-15	1.9E-17	14431	guanidinoacetate methyltransferase
Ganc	1.71	0.77	3.0E-04	2.4E-05	76051	glucosidase, alpha; neutral C
Gas2	0.46	-1.13	2.5E-05	1.5E-06	14453	growth arrest specific 2
Gas6	1.96	0.97	1.5E-11	3.5E-13	14456	growth arrest specific 6
Gata6	2.01	1.01	5.7E-03	7.3E-04	14465	GATA binding protein 6
Gatm	2.62	1.39	5.6E-13	1.1E-14	67092	glycine amidinotransferase (L-arginine:glycine amidinotransferase)
Gatsl2	2.38	1.25	1.0E-13	1.9E-15	80909	GATS protein-like 2
Gbas	2.68	1.42	3.4E-22	2.6E-24	14467	glioblastoma amplified sequence
Gbe1	3.31	1.73	1.8E-19	1.8E-21	74185	glucan (1,4-alpha-), branching enzyme 1
Gbp2	2.38	1.25	1.5E-03	1.6E-04	14469	guanylate binding protein 2
Gbp3	2.50	1.32	7.6E-05	5.3E-06	55932	guanylate binding protein 3
Gbp7	1.87	0.90	2.6E-04	2.0E-05	229900	guanylate binding protein 7
Gbp9	1.67	0.74	2.7E-03	3.1E-04	236573	guanylate-binding protein 9
Gch1	2.37	1.24	7.0E-06	3.8E-07	14528	GTP cyclohydrolase 1
Gdf1	1.58	0.66	9.3E-04	8.8E-05	14559	growth differentiation factor 1
Gdf10	0.64	-0.65	8.2E-04	7.5E-05	14560	growth differentiation factor 10
Gdf5	0.22	-2.16	9.5E-06	5.2E-07	14563	growth differentiation factor 5
Gdf6	0.35	-1.53	2.9E-04	2.3E-05	242316	growth differentiation factor 6
Gemin4	0.64	-0.63	1.3E-04	9.8E-06	276919	gem (nuclear organelle) associated protein 4
Gfra1	0.65	-0.63	1.1E-03	1.1E-04	14585	glial cell line derived neurotrophic factor family receptor alpha 1
Ggt5	2.30	1.20	3.0E-08	1.1E-09	23887	gamma-glutamyltransferase 5
Ghitm	1.66	0.73	3.9E-07	1.6E-08	66092	growth hormone inducible transmembrane protein
Gimap8	1.75	0.81	1.3E-03	1.3E-04	243374	GTPase, IMAP family member 8
Gins1	0.61	-0.70	4.1E-03	4.9E-04	69270	GINS complex subunit 1 (Psf1 homolog)
Gins3	0.60	-0.73	1.5E-03	1.6E-04	78833	GINS complex subunit 3 (Psf3 homolog)
Gipr	4.75	2.25	1.7E-14	2.7E-16	381853	gastric inhibitory polypeptide receptor
Gja1	0.54	-0.88	2.8E-05	1.7E-06	14609	gap junction protein, alpha 1
Gja3	2.13	1.09	7.4E-07	3.2E-08	14611	gap junction protein, alpha 3
Gjc3	2.65	1.40	1.5E-06	6.9E-08	118446	gap junction protein, gamma 3
Gjd4	1.61	0.69	6.0E-03	7.8E-04	225152	gap junction protein, delta 4
Gldc	0.13	-2.97	4.4E-06	2.3E-07	104174	glycine decarboxylase
Glt28d2	1.96	0.97	6.7E-05	4.6E-06	320302	glycosyltransferase 28 domain containing 2
Glul	1.83	0.87	9.2E-10	2.6E-11	14645	glutamate-ammonia ligase (glutamine synthetase)
Gm11149	1.83	0.87	9.8E-05	7.0E-06	100036537	predicted gene 11149
Gm11201	1.65	0.72	5.4E-03	6.9E-04	790910	predicted gene 11201
Gm13031	2.52	1.34	1.6E-05	9.2E-07	100126227	predicted gene 13031
Gm13889	2.31	1.21	1.1E-08	3.4E-10	620695	predicted gene 13889
Gm1987	0.55	-0.86	6.8E-03	8.9E-04	100504362	predicted gene 1987
Gm20741	0.00	-10.40	4.6E-128	6.1E-132	433047	predicted gene 20741
Gm21949	2.50	1.32	4.1E-14	7.0E-16	100505386	predicted gene 21949
Gm44502	4.89	2.29	5.7E-54	2.6E-57	100134861	predicted readthrough transcript (NMD candidate) 44502
Gm4814	7.96	2.99	1.9E-26	1.1E-28	100502942	predicted gene 4814
Gm4980	4.19	2.07	2.9E-20	2.6E-22	100503386	predicted gene 4980
Gm5105	2.66	1.41	2.1E-07	8.5E-09	329763	predicted gene 5105
Gm5111	5.21	2.38	5.1E-14	8.7E-16	330305	predicted gene 5111
Gm5483	8.46	3.08	1.0E-13	1.8E-15	433016	predicted gene 5483
Gm572	2.41	1.27	7.5E-05	5.2E-06	230909	predicted gene 572
Gm6307	2.06	1.04	2.3E-04	1.8E-05	622283	predicted gene 6307
Gm7120	0.19	-2.41	2.2E-13	4.1E-15	633640	predicted gene 7120
Gmppb	0.65	-0.63	1.2E-03	1.1E-04	331026	GDP-mannose pyrophosphorylase B
Gmpr	2.50	1.32	2.0E-15	3.0E-17	66355	guanosine monophosphate reductase
Gng4	0.24	-2.03	1.0E-03	9.7E-05	14706	guanine nucleotide binding protein (G protein), gamma 4
Got1	2.57	1.36	1.2E-18	1.3E-20	14718	glutamate oxaloacetate transaminase 1, soluble
Got2	1.88	0.91	3.4E-11	8.2E-13	14719	glutamate oxaloacetate transaminase 2, mitochondrial
Gp1bb	0.49	-1.02	1.6E-05	9.0E-07	14724	glycoprotein Ib, beta polypeptide

Gpc2	0.50	-1.00	5.1E-05	3.4E-06	71951	glypican 2 (cerebroglycan)
Gpd1	45.28	5.50	2.1E-33	5.4E-36	14555	glycerol-3-phosphate dehydrogenase 1 (soluble)
Gpd1l	1.73	0.79	5.4E-09	1.7E-10	333433	glycerol-3-phosphate dehydrogenase 1-like
Gpi1	1.74	0.80	3.4E-09	1.0E-10	14751	glucose phosphate isomerase 1
Gpihbp1	2.23	1.16	1.1E-09	3.1E-11	68453	GPI-anchored HDL-binding protein 1
Gpr116	1.92	0.94	7.0E-12	1.6E-13	224792	G protein-coupled receptor 116
Gpr146	2.58	1.37	5.2E-10	1.4E-11	80290	G protein-coupled receptor 146
Gpr157	2.42	1.27	6.0E-17	7.4E-19	269604	G protein-coupled receptor 157
Gpr160	0.42	-1.26	4.3E-03	5.2E-04	71862	G protein-coupled receptor 160
Gpr27	0.51	-0.98	2.0E-03	2.1E-04	14761	G protein-coupled receptor 27
Gpr88	0.11	-3.22	5.2E-16	7.1E-18	64378	G-protein coupled receptor 88
Gprc5a	0.38	-1.40	1.3E-08	4.3E-10	232431	G protein-coupled receptor, family C, group 5, member A
Gprc5c	1.52	0.61	1.5E-03	1.5E-04	70355	G protein-coupled receptor, family C, group 5, member C
Gprin2	0.29	-1.79	5.6E-03	7.2E-04	432839	G protein regulated inducer of neurite outgrowth 2
Gpt	2.22	1.15	3.6E-04	2.9E-05	76282	glutamic pyruvic transaminase, soluble
Gpx3	0.09	-3.47	7.9E-47	7.5E-50	14778	glutathione peroxidase 3
Gramd1b	1.55	0.64	4.3E-03	5.1E-04	235283	GRAM domain containing 1B
Grb14	2.25	1.17	2.1E-06	9.8E-08	50915	growth factor receptor bound protein 14
Grem1	0.46	-1.13	1.9E-03	2.0E-04	23892	gremlin 1
Grhl1	0.24	-2.05	5.6E-03	7.2E-04	195733	grainyhead-like 1
Gria1	0.45	-1.15	3.7E-03	4.4E-04	14799	glutamate receptor, ionotropic, AMPA1 (alpha 1)
Grik3	0.22	-2.19	1.8E-08	5.9E-10	14807	glutamate receptor, ionotropic, kainate 3
Grina	1.52	0.60	2.0E-05	1.2E-06	66168	glutamate receptor, ionotropic, N-methyl D-aspartate-associated protein 1 (glutamate binding)
Grip1	0.59	-0.75	2.1E-03	2.2E-04	74053	glutamate receptor interacting protein 1
Grip2	1.97	0.98	8.4E-08	3.2E-09	243547	glutamate receptor interacting protein 2
Gsap	1.67	0.74	6.7E-03	8.8E-04	212167	gamma-secretase activating protein
Gsn	1.58	0.66	4.2E-06	2.1E-07	227753	gelsolin
Gsta4	0.28	-1.85	4.2E-09	1.3E-10	14860	glutathione S-transferase, alpha 4
Gstm1	1.90	0.92	1.9E-06	9.1E-08	14862	glutathione S-transferase, mu 1
Gstt3	0.52	-0.93	2.4E-04	1.9E-05	103140	glutathione S-transferase, theta 3
Gstz1	1.53	0.62	9.7E-05	6.9E-06	14874	glutathione transferase zeta 1 (maleylacetoacetate isomerase)
Gtse1	0.66	-0.61	1.9E-03	2.0E-04	29870	G two S phase expressed protein 1
Gyg	2.18	1.13	6.6E-17	8.1E-19	27357	glycogenin
Gyltl1b	0.26	-1.94	8.0E-04	7.3E-05	228366	glycosyltransferase-like 1B
Gys1	2.42	1.27	6.6E-20	6.0E-22	14936	glycogen synthase 1, muscle
H19	1.95	0.96	4.3E-12	9.5E-14	14955	H19 fetal liver mRNA
H2-Eb1	1.96	0.97	6.7E-03	8.9E-04	14969	histocompatibility 2, class II antigen E beta
H2afy2	0.56	-0.84	3.5E-09	1.1E-10	404634	H2A histone family, member Y2
H2afy3	0.57	-0.81	1.5E-03	1.5E-04	67552	H2A histone family, member Y3
Hadh	1.89	0.92	2.7E-09	8.2E-11	15107	hydroxyacyl-Coenzyme A dehydrogenase
Hadha	1.77	0.82	4.0E-09	1.2E-10	97212	hydroxyacyl-Coenzyme A dehydrogenase/3-ketoacyl-Coenzyme A thiolase/enoyl-Coenzyme A hydratase (trifunctional protein), alpha subun
Hadhb	1.72	0.78	3.0E-04	2.4E-05	231086	hydroxyacyl-Coenzyme A dehydrogenase/3-ketoacyl-Coenzyme A thiolase/enoyl-Coenzyme A hydratase (trifunctional protein), beta subunit
Hagh	1.66	0.73	1.5E-04	1.1E-05	14651	hydroxyacyl glutathione hydrolase
Hand2	0.32	-1.64	1.3E-05	7.3E-07	15111	heart and neural crest derivatives expressed transcript 2
Hba-x	0.17	-2.56	7.7E-16	1.1E-17	15126	hemoglobin X, alpha-like embryonic chain in Hba complex
Hbb-y	0.18	-2.49	9.8E-13	2.0E-14	15135	hemoglobin Y, beta-like embryonic chain
Hcfc1r1	2.08	1.06	2.3E-13	4.3E-15	353502	host cell factor C1 regulator 1 (XPO1-dependent)
Hdac9	1.72	0.78	6.7E-05	4.6E-06	79221	histone deacetylase 9
Hdgfrp3	2.08	1.06	7.5E-12	1.7E-13	29877	hepatoma-derived growth factor, related protein 3
Heatr1	0.64	-0.64	4.7E-05	3.1E-06	217995	HEAT repeat containing 1
Hectd2	1.68	0.75	3.4E-04	2.8E-05	226098	HECT domain containing 2
Herc3	3.10	1.63	2.4E-21	2.0E-23	73998	hect domain and RLD 3
Hfe	1.69	0.76	2.3E-03	2.5E-04	15216	hemochromatosis
Hfe2	2.22	1.15	3.7E-11	8.8E-13	69585	hemochromatosis type 2 (juvenile)
Hhatl	4.19	2.07	5.5E-26	3.3E-28	74770	hedgehog acyltransferase-like
Hibadh	1.87	0.90	3.7E-10	1.0E-11	58875	3-hydroxyisobutyrate dehydrogenase
Hibch	1.65	0.72	8.9E-04	8.3E-05	227095	3-hydroxyisobutyryl-Coenzyme A hydrolase
Hif1an	1.77	0.82	3.9E-09	1.2E-10	319594	hypoxia-inducible factor 1, alpha subunit inhibitor
Hif3a	1.63	0.71	1.7E-06	7.9E-08	53417	hypoxia inducible factor 3, alpha subunit
Higd2a	1.52	0.61	1.5E-04	1.1E-05	67044	HIG1 domain family, member 2A
Hils1	2.63	1.40	1.8E-05	1.1E-06	54388	histone H1-like protein in spermatids 1
Hint2	2.33	1.22	6.3E-07	2.7E-08	68917	histidine triad nucleotide binding protein 2
Hint3	1.69	0.76	3.4E-03	3.9E-04	66847	histidine triad nucleotide binding protein 3
Hip1	1.73	0.79	6.4E-07	2.8E-08	215114	huntingtin interacting protein 1
Hist1h1c	2.25	1.17	3.4E-10	9.1E-12	50708	histone cluster 1, H1c
Hist1h2bc	2.25	1.17	5.2E-09	1.6E-10	68024	histone cluster 1, H2bc
Hk2	1.88	0.91	6.4E-11	1.6E-12	15277	hexokinase 2
Hmcn1	0.64	-0.64	4.2E-03	5.0E-04	545370	hemicentin 1
Hmga2	0.58	-0.79	3.6E-07	1.5E-08	15364	high mobility group AT-hook 2
Hn1l	0.59	-0.75	2.2E-08	7.5E-10	52009	hematological and neurological expressed 1-like
Homer1	1.61	0.68	8.9E-04	8.3E-05	26556	homer homolog 1
Hoxa3	1.64	0.72	7.1E-03	9.6E-04	15400	homeobox A3
Hoxa4	2.95	1.56	7.1E-08	2.7E-09	15401	homeobox A4
Hoxa5	2.22	1.15	1.4E-04	9.9E-06	15402	homeobox A5
Hoxa7	1.71	0.78	2.6E-03	2.9E-04	15404	homeobox A7
Hoxb4	2.37	1.24	5.2E-04	4.5E-05	15412	homeobox B4
Hoxd10	0.42	-1.25	3.2E-03	3.7E-04	15430	homeobox D10
Hoxd11	0.34	-1.56	8.8E-04	8.2E-05	15431	homeobox D11
Hp	2.51	1.33	9.7E-04	9.2E-05	15439	haptoglobin
Hpse2	0.45	-1.16	1.6E-03	1.7E-04	545291	heparanase 2
Hrasls	4.99	2.32	1.9E-12	4.0E-14	27281	HRAS-like suppressor
Hrc	3.35	1.74	4.7E-22	3.7E-24	15464	histidine rich calcium binding protein
Hsd11b2	2.56	1.36	3.4E-07	1.4E-08	15484	hydroxysteroid 11-beta dehydrogenase 2
Hsd17b7	0.55	-0.86	2.1E-04	1.6E-05	15490	hydroxysteroid (17-beta) dehydrogenase 7
Hsdl2	1.55	0.63	3.4E-03	3.9E-04	72479	hydroxysteroid dehydrogenase like 2
Hsf2	1.55	0.63	3.0E-04	2.4E-05	15500	heat shock factor 2
Hsf2bp	3.13	1.64	1.6E-09	4.8E-11	74377	heat shock transcription factor 2 binding protein
Hspa12a	1.53	0.62	6.9E-03	9.1E-04	73442	heat shock protein 12A
Hspa1a	0.32	-1.66	3.8E-04	3.1E-05	193740	heat shock protein 1A
Hspa1b	0.23	-2.12	7.9E-05	5.6E-06	15511	heat shock protein 1B
Hspa1l	1.71	0.78	4.1E-03	4.9E-04	15482	heat shock protein 1-like
Hspa5	0.59	-0.77	4.7E-06	2.4E-07	14828	heat shock protein 5
Hspb2	1.67	0.74	3.1E-05	1.9E-06	69253	heat shock protein 2
Hspb3	2.39	1.26	1.3E-09	3.9E-11	56534	heat shock protein 3
Hspb6	5.85	2.55	7.6E-48	6.7E-51	243912	heat shock protein, alpha-crystallin-related, B6
Hspb7	4.62	2.21	4.6E-21	4.0E-23	29818	heat shock protein family, member 7 (cardiovascular)
Hspb8	2.84	1.51	4.1E-18	4.5E-20	80888	heat shock protein 8
Hunk	0.55	-0.86	1.8E-08	6.2E-10	26559	hormonally upregulated Neu-associated kinase
Hydin	4.43	2.15	1.8E-10	4.8E-12	244653	HYDIN, axonemal central pair apparatus protein
Hyou1	0.52	-0.95	4.0E-13	7.7E-15	12282	hypoxia up-regulated 1
Idh2	1.59	0.67	2.7E-06	1.3E-07	269951	isocitrate dehydrogenase 2 (NADP+), mitochondrial
Idh3a	3.50	1.81	1.9E-29	7.7E-32	67834	isocitrate dehydrogenase 3 (NAD+) alpha
Idh3b	1.90	0.93	5.3E-12	1.2E-13	170718	isocitrate dehydrogenase 3 (NAD+) beta
Idh3g	1.77	0.82	1.4E-09	4.1E-11	15929	isocitrate dehydrogenase 3 (NAD+), gamma
Ier3	2.32	1.22	5.3E-06	2.8E-07	15937	immediate early response 3
Ifi27	1.64	0.71	2.8E-04	2.2E-05	52668	interferon, alpha-inducible protein 27
Ifnlr1	0.26	-1.96	3.6E-04	2.9E-05	242700	interferon lambda receptor 1
Ift122	1.63	0.70	1.3E-05	7.2E-07	81896	intraflagellar transport 122
Igf2bp1	0.65	-0.61	3.5E-05	2.2E-06	140486	insulin-like growth factor 2 mRNA binding protein 1
Igfbp6	2.36	1.24	6.3E-11	1.6E-12	16012	insulin-like growth factor binding protein 6
Igfbp7	1.72	0.79	3.1E-07	1.3E-08	29817	insulin-like growth factor binding protein 7
Iglon5	0.39	-1.36	8.6E-06	4.7E-07	210094	IgLON family member 5
Igsf1	7.38	2.88	5.3E-45	5.4E-48	209268	immunoglobulin superfamily, member 1
Igsf9	0.36	-1.46	5.9E-03	7.6E-04	93842	immunoglobulin superfamily, member 9
Igtp	2.64	1.40	1.1E-11	2.4E-13	16145	interferon gamma induced GTPase
Iigp1	2.55	1.35	1.1E-04	7.6E-06	60440	interferon inducible GTPase 1
Il10ra	1.76	0.82	4.5E-03	5.5E-04	16154	interleukin 10 receptor, alpha
Il33	1.79	0.84	6.5E-03	8.5E-04	77125	interleukin 33
Immt	1.64	0.72	8.8E-07	3.9E-08	76614	inner membrane protein, mitochondrial
Impa2	1.69	0.75	2.1E-07	8.5E-09	114663	inositol (myo)-1(or 4)-monophosphatase 2
Inafm1	0.65	-0.61	3.3E-03	3.9E-04	66300	InaF motif containing 1
Inhbb	0.49	-1.03	6.9E-03	9.1E-04	16324	inhibin beta-B
Ino80dos	1.92	0.94	9.6E-07	4.3E-08	100380944	INO80 complex subunit D, opposite strand
Inpp4b	1.89	0.92	4.3E-04	3.6E-05	234515	inositol polyphosphate-4-phosphatase, type II
Inpp5a	1.84	0.88	8.6E-10	2.4E-11	212111	inositol polyphosphate-5-phosphatase A
Inpp5k	1.86	0.89	2.3E-09	7.0E-11	19062	inositol polyphosphate 5-phosphatase K
Insr	1.80	0.85	3.4E-09	1.0E-10	16337	insulin receptor
Ip6k3	8.35	3.06	4.9E-26	2.9E-28	271424	inositol hexaphosphate kinase 3
Ipo13	1.58	0.66	1.9E-06	9.1E-08	230673	importin 13
Irf6	0.55	-0.87	4.1E-04	3.4E-05	54139	interferon regulatory factor 6
Irgm2	2.85	1.51	3.2E-10	8.8E-12	54396	immunity-related GTPase family M member 2

Irx1	0.23	-2.12	1.3E-03	1.3E-04	16371	Iroquois related homeobox 1
Isca1	1.82	0.86	1.1E-09	3.3E-11	69046	iron-sulfur cluster assembly 1 homolog
Isg20l2	0.59	-0.76	8.5E-08	3.2E-09	229504	interferon stimulated exonuclease gene 20-like 2
Ism1	0.45	-1.14	3.6E-05	2.3E-06	319909	isthmin 1 homolog
Isyna1	0.65	-0.62	2.1E-05	1.3E-06	71780	myo-inositol 1-phosphate synthase A1
Itga7	2.12	1.09	3.1E-09	9.4E-11	16404	integrin alpha 7
Itgal	1.77	0.82	4.2E-04	3.5E-05	16408	integrin alpha L
Itgb1bp2	3.72	1.89	3.9E-23	2.8E-25	26549	integrin beta 1 binding protein 2
Itgb6	1.78	0.83	6.8E-04	6.1E-05	16420	integrin beta 6
Itih5	1.70	0.76	7.1E-06	3.8E-07	209378	inter-alpha (globulin) inhibitor H5
Itm2a	1.69	0.76	2.6E-06	1.3E-07	16431	integral membrane protein 2A
Itpkc	0.65	-0.62	5.6E-03	7.1E-04	233011	inositol 1,4,5-trisphosphate 3-kinase C
Itprip	0.52	-0.95	1.1E-06	5.2E-08	414801	inositol 1,4,5-triphosphate receptor interacting protein
Jam2	1.84	0.88	1.0E-07	3.9E-09	67374	junction adhesion molecule 2
Jph1	3.16	1.66	6.0E-20	5.4E-22	57339	junctophilin 1
Jph2	2.26	1.18	5.7E-11	1.4E-12	59091	junctophilin 2
Jph4	0.52	-0.93	8.5E-04	7.9E-05	319984	junctophilin 4
Jrk	0.59	-0.75	3.8E-03	4.6E-04	16469	jerky
Jsrp1	3.88	1.96	1.3E-31	4.2E-34	71912	junctional sarcoplasmic reticulum protein 1
Jup	0.48	-1.05	4.9E-03	6.1E-04	16480	junction plakoglobin
Kat2b	1.54	0.62	1.4E-04	1.0E-05	18519	K(lysine) acetyltransferase 2B
Kazn	0.54	-0.88	2.7E-05	1.6E-06	71529	kazrin, periplakin interacting protein
Kbtbd12	3.51	1.81	1.1E-11	2.4E-13	74589	kelch repeat and BTB (POZ) domain containing 12
Kbtbd13	3.00	1.58	7.1E-13	1.4E-14	74492	kelch repeat and BTB (POZ) domain containing 13
Kcna1	0.49	-1.04	3.6E-05	2.3E-06	16485	potassium voltage-gated channel, shaker-related subfamily, member 1
Kcna2	1.72	0.78	4.6E-03	5.7E-04	16490	potassium voltage-gated channel, shaker-related subfamily, member 2
Kcna7	6.93	2.79	3.3E-21	2.8E-23	16495	potassium voltage-gated channel, shaker-related subfamily, member 7
Kcnb1	2.50	1.32	3.5E-13	6.6E-15	16500	potassium voltage gated channel, Shab-related subfamily, member 1
Kcnc1	3.77	1.92	1.6E-11	3.7E-13	16502	potassium voltage gated channel, Shaw-related subfamily, member 1
Kcnc4	2.17	1.12	6.8E-05	4.7E-06	99738	potassium voltage gated channel, Shaw-related subfamily, member 4
Kcne1l	1.97	0.98	1.1E-09	3.2E-11	66240	potassium voltage-gated channel, Isk-related family, member 1-like, pseudogene
Kcnf1	9.34	3.22	7.2E-28	3.4E-30	382571	potassium voltage-gated channel, subfamily F, member 1
Kcnj10	2.04	1.03	5.4E-03	6.8E-04	16513	potassium inwardly-rectifying channel, subfamily J, member 10
Kcnj11	3.16	1.66	4.4E-18	4.8E-20	16514	potassium inwardly rectifying channel, subfamily J, member 11
Kcnj12	3.55	1.83	2.9E-18	3.1E-20	16515	potassium inwardly-rectifying channel, subfamily J, member 12
Kcnj15	0.55	-0.85	1.9E-03	2.0E-04	16516	potassium inwardly-rectifying channel, subfamily J, member 15
Kcnj8	1.58	0.66	5.8E-04	5.1E-05	16523	potassium inwardly-rectifying channel, subfamily J, member 8
Kcnk2	0.57	-0.81	1.2E-03	1.2E-04	16526	potassium channel, subfamily K, member 2
Kcnk3	5.58	2.48	3.6E-07	1.5E-08	16527	potassium channel, subfamily K, member 3
Kcnma1	1.71	0.77	2.8E-03	3.1E-04	16531	potassium large conductance calcium-activated channel, subfamily M, alpha member 1
Kcnmb4	0.52	-0.95	6.7E-04	6.0E-05	58802	potassium large conductance calcium-activated channel, subfamily M, beta member 4
Kcnq1	2.56	1.36	3.0E-08	1.1E-09	16535	potassium voltage-gated channel, subfamily Q, member 1
Kcnq4	2.51	1.33	6.7E-10	1.9E-11	60613	potassium voltage-gated channel, subfamily Q, member 4
Kctd1	0.42	-1.25	6.8E-05	4.7E-06	106931	potassium channel tetramerisation domain containing 1
Kctd12	0.65	-0.61	3.2E-04	2.6E-05	239217	potassium channel tetramerisation domain containing 12
Kctd15	0.59	-0.77	1.1E-05	6.1E-07	233107	potassium channel tetramerisation domain containing 15
Kdf1	0.23	-2.09	6.1E-03	7.9E-04	69073	keratinocyte differentiation factor
Kif1b	1.83	0.87	1.8E-08	6.0E-10	16561	kinesin family member 1B
Kif21a	0.33	-1.60	4.7E-05	3.1E-06	16564	kinesin family member 21A
Kif21b	0.66	-0.60	7.1E-03	9.5E-04	16565	kinesin family member 21B
Kif26b	0.43	-1.23	7.2E-07	3.2E-08	269152	kinesin family member 26B
Kif7	0.61	-0.72	3.1E-04	2.5E-05	16576	kinesin family member 7
Kirrel3	0.38	-1.38	7.4E-05	5.1E-06	67703	kin of IRRE like 3
Kl	4.34	2.12	4.7E-17	5.7E-19	16591	klotho
Klc3	0.45	-1.15	5.3E-04	4.6E-05	232943	kinesin light chain 3
Klf15	2.74	1.45	6.2E-11	1.5E-12	66277	Kruppel-like factor 15
Klf5	0.47	-1.09	5.5E-04	4.8E-05	12224	Kruppel-like factor 5
Klf9	2.76	1.46	2.8E-11	6.5E-13	16601	Kruppel-like factor 9
Klhdc1	1.78	0.83	2.0E-03	2.1E-04	271005	kelch domain containing 1
Klhdc3	1.62	0.70	1.0E-06	4.6E-08	71765	kelch domain containing 3
Klhl23	1.57	0.66	1.8E-05	1.1E-06	277396	kelch-like 23
Klhl24	2.03	1.02	3.5E-11	8.4E-13	75785	kelch-like 24
Klhl3	2.16	1.11	2.7E-06	1.3E-07	100503085	kelch-like 3
Klhl30	2.86	1.52	1.6E-16	2.1E-18	70788	kelch-like 30
Klhl31	2.97	1.57	1.2E-15	1.7E-17	244923	kelch-like 31
Klhl38	3.56	1.83	1.6E-17	1.8E-19	268807	kelch-like 38
Klhl40	2.14	1.10	2.8E-11	6.6E-13	72330	kelch-like 40
Klhl41	2.60	1.38	2.7E-14	4.5E-16	228003	kelch-like 41
Klk10	0.14	-2.89	5.6E-07	2.4E-08	69540	kallikrein related-peptidase 10
Klk1b26	5.06	2.34	5.2E-14	9.0E-16	16618	kallikrein 1-related petidase b26
Klrg2	0.32	-1.64	2.3E-03	2.5E-04	74253	killer cell lectin-like receptor subfamily G, member 2
Kremen2	0.24	-2.05	2.0E-04	1.5E-05	73016	kringle containing transmembrane protein 2
Krt13	0.05	-4.20	1.2E-20	1.1E-22	16663	keratin 13
Krt18	0.42	-1.25	4.5E-05	2.9E-06	16668	keratin 18
Krt19	0.01	-6.97	6.8E-203	4.4E-207	16669	keratin 19
Krt4	0.01	-6.89	4.5E-95	8.9E-99	16682	keratin 4
Krt6a	0.01	-6.20	1.0E-51	5.2E-55	16687	keratin 6A
Krt6b	0.02	-5.40	1.2E-10	3.0E-12	16688	keratin 6B
Krt7	0.04	-4.83	2.5E-54	1.1E-57	110310	keratin 7
Krt77	0.07	-3.74	9.3E-04	8.8E-05	406220	keratin 77
Krt8	0.60	-0.75	2.0E-03	2.2E-04	16691	keratin 8
Krtap13	0.00	-13.01	2.9E-183	2.8E-187	16699	keratin associated protein 13
Ksr1	1.78	0.83	6.4E-07	2.8E-08	16706	kinase suppressor of ras 1
Ktn1	1.56	0.64	7.7E-06	4.1E-07	16709	kinectin 1
Ky	1.82	0.86	2.6E-05	1.6E-06	16716	kyphoscoliosis peptidase
L2hgdh	1.71	0.77	5.1E-07	2.2E-08	217666	L-2-hydroxyglutarate dehydrogenase
Lace1	1.65	0.72	2.3E-03	2.4E-04	215951	lactation elevated 1
Lama2	1.94	0.96	1.2E-10	3.2E-12	16773	laminin, alpha 2
Larp1b	2.93	1.55	1.4E-12	2.9E-14	214048	La ribonucleoprotein domain family, member 1B
Lbp	1.77	0.82	1.7E-03	1.7E-04	16803	lipopolysaccharide binding protein
Lce3f	0.03	-5.22	1.6E-15	2.4E-17	69520	late cornified envelope 3F
Lcn2	6.16	2.62	7.7E-11	1.9E-12	16819	lipocalin 2
Ldb3	4.59	2.20	1.0E-26	5.5E-29	24131	LIM domain binding 3
Ldha	1.96	0.97	8.0E-12	1.8E-13	16828	lactate dehydrogenase A
Ldhb	1.87	0.90	4.4E-12	9.7E-14	16832	lactate dehydrogenase B
Lef1	0.38	-1.41	7.0E-04	6.3E-05	16842	lymphoid enhancer binding factor 1
Lgals3bp	1.83	0.87	3.4E-06	1.7E-07	19039	lectin, galactoside-binding, soluble, 3 binding protein
Lgmn	1.63	0.71	9.3E-07	4.2E-08	19141	legumain
Lhfpl1	0.53	-0.91	2.8E-04	2.2E-05	237091	lipoma HMGIC fusion partner-like 1
Limk2	0.56	-0.83	5.9E-04	5.2E-05	16886	LIM motif-containing protein kinase 2
Lims2	2.31	1.21	5.9E-07	2.5E-08	225341	LIM and senescent cell antigen like domains 2
Lingo3	4.78	2.26	7.3E-17	9.1E-19	237403	leucine rich repeat and Ig domain containing 3
Lipe	6.03	2.59	2.8E-06	1.4E-07	16890	lipase, hormone sensitive
Lipg	0.41	-1.30	4.7E-04	4.0E-05	16891	lipase, endothelial
Lix1	0.43	-1.21	7.8E-06	4.2E-07	66643	limb expression 1 homolog
Lmcd1	2.46	1.30	5.7E-11	1.4E-12	30937	LIM and cysteine-rich domains 1
Lmo1	0.38	-1.40	8.3E-04	7.6E-05	109594	LIM domain only 1
Lmo4	0.67	-0.59	1.4E-04	1.0E-05	16911	LIM domain only 4
Lmo7	2.03	1.02	6.0E-13	1.2E-14	380928	LIM domain only 7
Lmod1	2.02	1.02	1.5E-04	1.1E-05	93689	leiomodin 1 (smooth muscle)
Lmod2	3.69	1.88	1.9E-14	3.0E-16	93677	leiomodin 2 (cardiac)
Lmod3	2.50	1.32	4.3E-10	1.2E-11	320502	leiomodin 3 (fetal)
Lmtk3	0.39	-1.37	5.1E-04	4.4E-05	381983	lemur tyrosine kinase 3
Lmx1b	0.32	-1.64	4.8E-06	2.5E-07	16917	LIM homeobox transcription factor 1 beta
Lpar2	0.46	-1.14	6.3E-05	4.3E-06	53978	lysophosphatidic acid receptor 2
Lpcat4	0.56	-0.83	5.7E-04	5.0E-05	99010	lysophosphatidylcholine acyltransferase 4
Lphn1	0.66	-0.61	1.5E-05	8.8E-07	330814	latrophilin 1
Lpin1	2.70	1.43	3.9E-13	7.5E-15	14245	lipin 1
Lpl	4.91	2.30	2.9E-26	1.7E-28	16956	lipoprotein lipase
Lrfn1	0.49	-1.02	4.5E-06	2.3E-07	80749	leucine rich repeat and fibronectin type III domain containing 1
Lrig1	1.51	0.60	1.5E-05	8.9E-07	16206	leucine-rich repeats and immunoglobulin-like domains 1
Lrp8	0.43	-1.21	1.6E-06	7.3E-08	16975	low density lipoprotein receptor-related protein 8, apolipoprotein e receptor
Lrrc1	0.55	-0.86	6.4E-05	4.4E-06	214345	leucine rich repeat containing 1
Lrrc14b	2.35	1.23	4.1E-13	7.8E-15	432779	leucine rich repeat containing 14B
Lrrc16a	0.61	-0.72	3.0E-04	2.4E-05	68732	leucine rich repeat containing 16A
Lrrc16b	0.52	-0.96	3.0E-03	3.4E-04	268747	leucine rich repeat containing 16B
Lrrc2	14.88	3.90	3.9E-23	2.8E-25	74249	leucine rich repeat containing 2
Lrrc20	1.78	0.83	5.7E-09	1.8E-10	216011	leucine rich repeat containing 20
Lrrc30	2.00	1.00	8.7E-04	8.1E-05	240131	leucine rich repeat containing 30
Lrrc38	1.82	0.86	2.5E-03	2.7E-04	242735	leucine rich repeat containing 38
Lrrc39	4.01	2.01	2.8E-17	3.4E-19	109245	leucine rich repeat containing 39

Lrrc45	0.66	-0.59	1.4E-03	1.4E-04	217366	leucine rich repeat containing 45
Lrrc75a	1.74	0.80	3.7E-07	1.5E-08	192976	leucine rich repeat containing 75A
Lrrfip1	2.29	1.20	4.5E-14	7.7E-16	16978	leucine rich repeat (in FLII) interacting protein 1
Lrtm1	3.43	1.78	1.2E-14	1.9E-16	319476	leucine-rich repeats and transmembrane domains 1
Lsmem1	2.33	1.22	1.6E-05	9.2E-07	380755	leucine-rich single-pass membrane protein 1
Lsr	0.34	-1.54	2.7E-03	3.0E-04	54135	lipolysis stimulated lipoprotein receptor
Lss	0.45	-1.15	1.1E-05	6.4E-07	16987	lanosterol synthase
Ltbp1	0.57	-0.81	2.1E-06	1.0E-07	268977	latent transforming growth factor beta binding protein 1
Ltbp2	0.61	-0.72	1.5E-05	8.5E-07	16997	latent transforming growth factor beta binding protein 2
Ltf	4.48	2.16	1.8E-08	6.1E-10	17002	lactotransferrin
Ly6a	2.89	1.53	7.0E-09	2.2E-10	110454	lymphocyte antigen 6 complex, locus A
Ly6c1	2.65	1.41	3.4E-10	9.2E-12	17067	lymphocyte antigen 6 complex, locus C1
Lynx1	1.79	0.84	8.8E-06	4.8E-07	23936	Ly6/neurotoxin 1
Lypd6b	0.28	-1.85	6.1E-03	7.9E-04	71897	LY6/PLAUR domain containing 6B
Lyrm5	2.14	1.10	2.4E-08	8.2E-10	67636	LYR motif containing 5
Lyrm7	1.80	0.85	4.2E-04	3.4E-05	75530	LYR motif containing 7
Lyz2	1.83	0.87	4.8E-05	3.1E-06	17105	lysozyme 2
Mab21l2	0.38	-1.38	2.8E-06	1.4E-07	23937	mab-21-like 2
Macrod1	2.95	1.56	1.2E-26	6.4E-29	107227	MACRO domain containing 1
Mafa	5.41	2.44	7.5E-20	6.9E-22	378435	v-maf musculoaponeurotic fibrosarcoma oncogene family, protein A
Maff	1.74	0.80	7.5E-05	5.2E-06	17133	v-maf musculoaponeurotic fibrosarcoma oncogene family, protein F
Mal	0.18	-2.49	9.0E-50	5.3E-53	17153	myelin and lymphocyte protein, T cell differentiation protein
Mall	0.48	-1.07	1.9E-07	7.7E-09	228576	mal, T cell differentiation protein-like
Mamstr	2.27	1.18	7.7E-12	1.7E-13	74490	MEF2 activating motif and SAP domain containing transcriptional regulator
Manba	1.59	0.67	5.3E-03	6.6E-04	110173	mannosidase, beta A, lysosomal
Manf	0.62	-0.69	9.2E-05	6.5E-06	74840	mesencephalic astrocyte-derived neurotrophic factor
Map1lc3a	1.98	0.98	2.2E-10	5.9E-12	66734	microtubule-associated protein 1 light chain 3 alpha
Map3k15	2.17	1.11	6.7E-16	9.2E-18	270672	mitogen-activated protein kinase kinase kinase 15
Map3k7cl	1.78	0.83	6.0E-04	5.3E-05	224419	Map3k7 C-terminal like
Map9	0.58	-0.79	3.2E-03	3.6E-04	213582	microtubule-associated protein 9
Mapk12	2.19	1.13	4.8E-10	1.3E-11	29857	mitogen-activated protein kinase 12
March9	0.48	-1.07	1.9E-04	1.4E-05	216438	membrane-associated ring finger (C3HC4) 9
Marcksl1	0.55	-0.85	2.4E-09	7.2E-11	17357	MARCKS-like 1
Matn4	0.33	-1.58	6.1E-04	5.3E-05	17183	matrilin 4
Mb	2.06	1.04	2.6E-04	2.1E-05	17189	myoglobin
Mboat2	0.49	-1.02	2.8E-03	3.2E-04	67216	membrane bound O-acyltransferase domain containing 2
Mbp	2.77	1.47	2.0E-08	6.7E-10	17196	myelin basic protein
Mcee	1.76	0.81	2.3E-04	1.8E-05	73724	methylmalonyl CoA epimerase
Mcf2l	2.01	1.01	8.1E-11	2.0E-12	17207	mcf.2 transforming sequence-like
Mchr1	4.02	2.01	1.3E-13	2.3E-15	207911	melanin-concentrating hormone receptor 1
Mcm10	0.55	-0.86	1.3E-05	7.5E-07	70024	minichromosome maintenance deficient 10
Mcm3	0.65	-0.62	3.7E-06	1.9E-07	17215	minichromosome maintenance deficient 3
Mcm5	0.62	-0.70	3.8E-07	1.6E-08	17218	minichromosome maintenance deficient 5, cell division cycle 46
Mcpt4	3.21	1.68	3.3E-10	8.9E-12	17227	mast cell protease 4
Mdfi	0.56	-0.83	2.5E-07	1.0E-08	17240	MyoD family inhibitor
Mdh1	2.16	1.11	4.7E-16	6.3E-18	17449	malate dehydrogenase 1, NAD (soluble)
Mdh2	1.90	0.93	1.8E-11	4.1E-13	17448	malate dehydrogenase 2, NAD (mitochondrial)
Mdk	0.54	-0.89	9.3E-08	3.5E-09	17242	midkine
Medag	1.79	0.84	9.9E-06	5.4E-07	70717	mesenteric estrogen dependent adipogenesis
Mef2c	1.89	0.92	2.4E-07	9.7E-09	17260	myocyte enhancer factor 2C
Mef2d	1.62	0.70	2.4E-05	1.4E-06	17261	myocyte enhancer factor 2D
Megf6	0.49	-1.04	3.4E-08	1.2E-09	230971	multiple EGF-like-domains 6
Mettl11b	3.17	1.66	1.9E-06	8.9E-08	240879	methyltransferase like 11B
Mettl20	1.86	0.90	8.6E-04	8.0E-05	320204	methyltransferase like 20
Mettl21c	18.04	4.17	2.5E-26	1.4E-28	433294	methyltransferase like 21C
Mettl21e	14.92	3.90	7.3E-27	3.8E-29	403183	methyltransferase like 21E
Mettl22	2.00	1.00	2.1E-06	1.0E-07	239706	methyltransferase like 22
Mfap5	2.21	1.14	7.8E-18	8.7E-20	50530	microfibrillar associated protein 5
Mfn2	2.21	1.14	9.7E-17	1.2E-18	170731	mitofusin 2
Mfsd2a	0.22	-2.16	2.3E-04	1.8E-05	76574	major facilitator superfamily domain containing 2A
Mfsd5	0.63	-0.66	1.2E-04	8.9E-06	106073	major facilitator superfamily domain containing 5
Mgat3	0.62	-0.68	4.8E-04	4.1E-05	17309	mannoside acetylglucosaminyltransferase 3
Mgll	1.83	0.87	7.7E-08	2.9E-09	23945	monoglyceride lipase
Mgst3	1.70	0.77	3.2E-05	2.0E-06	66447	microsomal glutathione S-transferase 3
Miat	0.42	-1.24	4.1E-09	1.3E-10	330166	myocardial infarction associated transcript (non-protein coding)
Mical3	1.76	0.82	1.4E-04	9.9E-06	194401	microtubule associated monoxygenase, calponin and LIM domain containing 3
Micu1	1.53	0.62	1.5E-05	8.6E-07	216001	mitochondrial calcium uptake 1
Mid1	0.53	-0.91	4.3E-03	5.2E-04	17318	midline 1
Mief2	2.56	1.36	2.6E-17	3.1E-19	237781	mitochondrial elongation factor 2
Mir127	3.07	1.62	4.6E-08	1.7E-09	387146	microRNA 127
Mir22hg	1.65	0.72	3.0E-04	2.4E-05	100042498	Mir22 host gene (non-protein coding)
Mir410	1.89	0.92	6.2E-04	5.5E-05	723863	microRNA 410
Mir431	2.90	1.54	8.9E-10	2.5E-11	723866	microRNA 431
Mir433	2.64	1.40	2.9E-09	8.9E-11	723937	microRNA 433
Mir434	2.86	1.52	8.9E-08	3.4E-09	723867	microRNA 434
Mir5114	0.66	-0.60	4.1E-03	4.9E-04	100628619	microRNA 5114
Mir675	1.86	0.89	3.2E-10	8.6E-12	735280	microRNA 675
Mirg	1.79	0.84	1.6E-09	4.6E-11	100040724	miRNA containing gene
Mkl1	1.62	0.69	4.6E-07	1.9E-08	223701	MKL (megakaryoblastic leukemia)/myocardin-like 1
Mknk2	1.52	0.61	8.4E-05	5.9E-06	17347	MAP kinase-interacting serine/threonine kinase 2
Mkrn2	1.61	0.69	3.2E-06	1.6E-07	67027	makorin, ring finger protein, 2
Mlf1	7.17	2.84	2.6E-34	5.8E-37	17349	myeloid leukemia factor 1
Mlip	6.97	2.80	2.6E-26	1.5E-28	69642	muscular LMNA-interacting protein
Mllt1	0.66	-0.61	1.2E-05	7.0E-07	64144	myeloid/lymphoid or mixed-lineage leukemia; translocated to, 1
Mllt11	1.91	0.94	6.0E-06	3.2E-07	56772	myeloid/lymphoid or mixed-lineage leukemia; translocated to, 11
Mllt3	2.64	1.40	3.0E-21	2.6E-23	70122	myeloid/lymphoid or mixed-lineage leukemia; translocated to, 3
Mlycd	1.75	0.80	4.3E-06	2.2E-07	56690	malonyl-CoA decarboxylase
Mme	0.55	-0.87	1.5E-05	8.7E-07	17380	membrane metallo endopeptidase
Mmp11	0.44	-1.20	2.4E-06	1.1E-07	17385	matrix metallopeptidase 11
Mmp8	9.56	3.26	1.7E-14	2.7E-16	17394	matrix metallopeptidase 8
Mndal	2.00	1.00	3.2E-03	3.7E-04	100040462	myeloid nuclear differentiation antigen like
Mocos	0.29	-1.80	7.2E-05	4.9E-06	68591	molybdenum cofactor sulfurase
Mospd1	1.82	0.86	2.5E-07	1.0E-08	70380	motile sperm domain containing 1
Mpc1	1.90	0.93	2.4E-03	2.5E-04	55951	mitochondrial pyruvate carrier 1
Mpc2	1.82	0.86	1.3E-08	4.2E-10	70456	mitochondrial pyruvate carrier 2
Mpi	2.54	1.34	3.0E-18	3.3E-20	110119	mannose phosphate isomerase
Mpp3	2.61	1.39	2.7E-12	5.9E-14	13384	membrane protein, palmitoylated 3 (MAGUK p55 subfamily member 3)
Mpp6	1.67	0.74	6.2E-06	3.3E-07	56524	membrane protein, palmitoylated 6 (MAGUK p55 subfamily member 6)
Mpp7	0.46	-1.12	5.0E-03	6.3E-04	75739	membrane protein, palmitoylated 7 (MAGUK p55 subfamily member 7)
Mpped1	0.23	-2.12	2.3E-07	9.2E-09	223726	metallophosphoesterase domain containing 1
Mpped2	0.52	-0.95	1.9E-04	1.4E-05	77015	metallophosphoesterase domain containing 2
Mpv17	1.57	0.65	1.9E-05	1.1E-06	17527	MpV17 mitochondrial inner membrane protein
Mpz	2.08	1.06	1.6E-05	9.4E-07	17528	myelin protein zero
Mpzl2	0.29	-1.79	7.2E-04	6.5E-05	14012	myelin protein zero-like 2
Mras	1.60	0.68	1.6E-05	9.1E-07	17532	muscle and microspikes RAS
Mrc1	1.57	0.65	1.9E-05	1.1E-06	17533	mannose receptor, C type 1
Mrgprb1	12.65	3.66	1.2E-15	1.7E-17	233231	MAS-related GPR, member B1
Mrgprb2	5.50	2.46	1.6E-14	2.5E-16	243979	MAS-related GPR, member B2
Mrpl12	1.82	0.86	1.1E-06	4.9E-08	56282	mitochondrial ribosomal protein L12
Mrpl30	1.60	0.68	8.5E-07	3.7E-08	107734	mitochondrial ribosomal protein L30
Mrpl33	1.52	0.60	1.7E-03	1.7E-04	66845	mitochondrial ribosomal protein L33
Mrpl42	2.01	1.01	5.3E-10	1.5E-11	67270	mitochondrial ribosomal protein L42
Mrpl47	1.54	0.62	5.2E-03	6.5E-04	74600	mitochondrial ribosomal protein L47
Mrpl51	1.74	0.79	1.8E-09	5.2E-11	66493	mitochondrial ribosomal protein L51
Mrpl53	1.60	0.68	3.0E-04	2.4E-05	68499	mitochondrial ribosomal protein L53
Mrps28	1.53	0.61	3.9E-03	4.6E-04	66230	mitochondrial ribosomal protein S28
Mrps35	1.78	0.83	1.4E-07	5.5E-09	232536	mitochondrial ribosomal protein S35
Msh6	0.65	-0.62	2.1E-06	1.0E-07	17688	mutS homolog 6
Msi1	0.55	-0.86	4.1E-04	3.4E-05	17690	Musashi homolog 1
Msi2	1.62	0.70	3.9E-03	4.7E-04	76626	Musashi homolog 2
Msln	0.04	-4.70	1.3E-41	1.7E-44	56047	mesothelin
Msmo1	0.54	-0.89	1.4E-08	4.5E-10	66234	methylsterol monoxygenase 1
Msr1	1.63	0.70	5.8E-03	7.5E-04	20288	macrophage scavenger receptor 1
Msrb1	1.60	0.68	1.0E-03	9.5E-05	27361	methionine sulfoxide reductase B1
Msrb3	1.57	0.65	2.8E-06	1.4E-07	320183	methionine sulfoxide reductase B3
Mstn	1.66	0.73	4.9E-04	4.2E-05	17700	myostatin
Mt1	2.15	1.11	5.7E-07	2.4E-08	17748	metallothionein 1
Mt2	2.37	1.24	1.7E-06	8.0E-08	17750	metallothionein 2
Mtfp1	2.36	1.24	1.4E-07	5.7E-09	67900	mitochondrial fission process 1
Mtm1	1.60	0.67	6.9E-03	9.1E-04	17772	X-linked myotubular myopathy gene 1
Mtus1	1.66	0.73	2.6E-06	1.3E-07	102103	mitochondrial tumor suppressor 1

Mtus2	4.08	2.03	1.1E-17	1.2E-19	77521	microtubule associated tumor suppressor candidate 2
Muc15	0.17	-2.55	2.3E-03	2.5E-04	269328	mucin 15
Murc	3.52	1.82	3.5E-18	3.9E-20	68016	muscle-related coiled-coil protein
Mustn1	2.47	1.30	8.4E-14	1.5E-15	66175	musculoskeletal, embryonic nuclear protein 1
Myadml2	5.39	2.43	1.0E-20	8.8E-23	68515	myeloid-associated differentiation marker-like 2
Mybpc1	5.21	2.38	2.6E-28	1.2E-30	109272	myosin binding protein C, slow-type
Mybpc2	61.33	5.94	1.3E-74	3.0E-78	233199	myosin binding protein C, fast-type
Myc	0.61	-0.70	2.5E-06	1.2E-07	17869	myelocytomatosis oncogene
Myeov2	1.65	0.72	4.3E-03	5.2E-04	66915	myeloma overexpressed 2
Myf6	3.34	1.74	3.1E-17	3.8E-19	17878	myogenic factor 6
Myh1	3.42	1.78	1.9E-12	3.9E-14	17879	myosin, heavy polypeptide 1, skeletal muscle, adult
Myh11	3.05	1.61	3.2E-14	5.3E-16	17880	myosin, heavy polypeptide 11, smooth muscle
Myh2	4.39	2.13	5.6E-06	3.0E-07	17882	myosin, heavy polypeptide 2, skeletal muscle, adult
Myh3	2.05	1.03	4.2E-07	1.8E-08	17883	myosin, heavy polypeptide 3, skeletal muscle, embryonic
Myh7	2.08	1.06	1.2E-07	4.5E-09	140781	myosin, heavy polypeptide 7, cardiac muscle, beta
Myh8	6.62	2.73	7.1E-33	2.0E-35	17885	myosin, heavy polypeptide 8, skeletal muscle, perinatal
Myl1	5.15	2.36	1.0E-31	3.2E-34	17901	myosin, light polypeptide 1
Myl2	4.25	2.09	8.4E-18	9.5E-20	17906	myosin, light polypeptide 2, regulatory, cardiac, slow
Myl3	2.84	1.50	5.4E-14	9.2E-16	17897	myosin, light polypeptide 3
Myl4	1.71	0.77	7.2E-05	4.9E-06	17896	myosin, light polypeptide 4
Myl9	2.30	1.20	4.5E-14	7.6E-16	98932	myosin, light polypeptide 9, regulatory
Mylk2	4.99	2.32	6.8E-33	1.9E-35	228785	myosin, light polypeptide kinase 2, skeletal muscle
Mylk3	4.96	2.31	2.3E-15	3.4E-17	213435	myosin light chain kinase 3
Mylk4	2.33	1.22	1.1E-10	2.7E-12	238564	myosin light chain kinase family, member 4
Mylpf	4.42	2.14	1.2E-22	8.8E-25	17907	myosin light chain, phosphorylatable, fast skeletal muscle
Myo18b	3.38	1.76	1.0E-17	1.2E-19	74376	myosin XVIIIb
Myo19	0.65	-0.62	5.2E-03	6.6E-04	66196	myosin XIX
Myo5b	0.65	-0.61	2.6E-03	2.9E-04	17919	myosin VB
Myo5c	5.36	2.42	7.0E-18	7.7E-20	208943	myosin VC
Myom1	3.26	1.70	1.2E-18	1.3E-20	17929	myomesin 1
Myom2	3.52	1.82	3.4E-21	2.9E-23	17930	myomesin 2
Myom3	2.24	1.17	7.3E-10	2.1E-11	242702	myomesin family, member 3
Myot	4.80	2.26	1.4E-26	7.4E-29	58916	myotilin
Myoz1	7.69	2.94	3.5E-39	5.1E-42	59011	myozenin 1
Myoz2	3.76	1.91	7.6E-18	8.5E-20	59006	myozenin 2
Myoz3	7.83	2.97	4.8E-35	9.2E-38	170947	myozenin 3
Mypn	3.09	1.63	1.4E-17	1.6E-19	68802	myopalladin
Naalad2	1.59	0.67	4.1E-03	5.0E-04	72560	N-acetylated alpha-linked acidic dipeptidase 2
Naca	1.98	0.98	2.1E-08	7.0E-10	17938	nascent polypeptide-associated complex alpha polypeptide
Nalcn	0.32	-1.63	1.2E-05	6.8E-07	338370	sodium leak channel, non-selective
Nampt	1.90	0.93	2.3E-09	7.0E-11	59027	nicotinamide phosphoribosyltransferase
Napepld	1.73	0.79	2.5E-03	2.8E-04	242864	N-acyl phosphatidylethanolamine phospholipase D
Nasp	0.62	-0.70	3.3E-07	1.4E-08	50927	nuclear autoantigenic sperm protein (histone-binding)
Nbeal2	0.39	-1.36	5.7E-05	3.8E-06	235627	neurobeachin-like 2
Ncam1	2.11	1.08	1.1E-10	2.9E-12	17967	neural cell adhesion molecule 1
Nceh1	1.84	0.88	1.7E-04	1.3E-05	320024	arylacetamide deacetylase-like 1
Ndrg2	4.05	2.02	3.1E-32	9.6E-35	29811	N-myc downstream regulated gene 2
Ndufa1	1.68	0.75	2.1E-05	1.3E-06	54405	NADH dehydrogenase (ubiquinone) 1 alpha subcomplex, 1
Ndufa10	1.71	0.78	4.8E-09	1.5E-10	67273	NADH dehydrogenase (ubiquinone) 1 alpha subcomplex 10
Ndufa11	1.58	0.66	8.1E-06	4.4E-07	69875	NADH dehydrogenase (ubiquinone) 1 alpha subcomplex 11
Ndufa12	1.74	0.80	3.7E-05	2.4E-06	66414	NADH dehydrogenase (ubiquinone) 1 alpha subcomplex, 12
Ndufa13	1.60	0.67	9.8E-06	5.4E-07	67184	NADH dehydrogenase (ubiquinone) 1 alpha subcomplex, 13
Ndufa2	1.54	0.63	4.4E-05	2.8E-06	17991	NADH dehydrogenase (ubiquinone) 1 alpha subcomplex, 2
Ndufa4	1.68	0.75	3.5E-07	1.4E-08	17992	NADH dehydrogenase (ubiquinone) 1 alpha subcomplex, 4
Ndufa5	2.04	1.03	2.5E-11	5.9E-13	68202	NADH dehydrogenase (ubiquinone) 1 alpha subcomplex, 5
Ndufa6	2.04	1.03	5.3E-14	9.2E-16	67130	NADH dehydrogenase (ubiquinone) 1 alpha subcomplex, 6 (B14)
Ndufa8	1.87	0.91	2.1E-11	5.0E-13	68375	NADH dehydrogenase (ubiquinone) 1 alpha subcomplex, 8
Ndufa9	1.78	0.83	1.6E-09	4.8E-11	66108	NADH dehydrogenase (ubiquinone) 1 alpha subcomplex, 9
Ndufab1	1.67	0.74	1.4E-03	1.4E-04	70316	NADH dehydrogenase (ubiquinone) 1, alpha/beta subcomplex, 1
Ndufaf1	1.63	0.70	4.5E-04	3.8E-05	69702	NADH dehydrogenase (ubiquinone) 1 alpha subcomplex, assembly factor 1
Ndufaf5	1.71	0.78	4.9E-04	4.2E-05	69487	NADH dehydrogenase (ubiquinone) complex I, assembly factor 5
Ndufb10	2.42	1.28	1.4E-17	1.6E-19	68342	NADH dehydrogenase (ubiquinone) 1 beta subcomplex, 10
Ndufb11	1.73	0.79	3.9E-08	1.4E-09	104130	NADH dehydrogenase (ubiquinone) 1 beta subcomplex, 11
Ndufb2	1.82	0.86	2.9E-05	1.8E-06	68198	NADH dehydrogenase (ubiquinone) 1 beta subcomplex, 2
Ndufb3	1.60	0.68	4.3E-06	2.2E-07	66495	NADH dehydrogenase (ubiquinone) 1 beta subcomplex 3
Ndufb5	1.92	0.94	7.1E-12	1.6E-13	66046	NADH dehydrogenase (ubiquinone) 1 beta subcomplex, 5
Ndufb6	1.65	0.72	1.6E-06	7.5E-08	230075	NADH dehydrogenase (ubiquinone) 1 beta subcomplex, 6
Ndufb7	1.80	0.85	1.6E-09	4.8E-11	66916	NADH dehydrogenase (ubiquinone) 1 beta subcomplex, 7
Ndufb8	2.00	1.00	3.7E-11	8.9E-13	67264	NADH dehydrogenase (ubiquinone) 1 beta subcomplex 8
Ndufb9	2.35	1.23	3.5E-16	4.5E-18	66218	NADH dehydrogenase (ubiquinone) 1 beta subcomplex, 9
Ndufc1	1.86	0.89	3.1E-06	1.5E-07	66377	NADH dehydrogenase (ubiquinone) 1, subcomplex unknown, 1
Ndufc2	1.76	0.82	1.7E-08	5.5E-10	68197	NADH dehydrogenase (ubiquinone) 1, subcomplex unknown, 2
Ndufs1	2.05	1.04	2.3E-13	4.3E-15	227197	NADH dehydrogenase (ubiquinone) Fe-S protein 1
Ndufs2	1.77	0.82	6.2E-09	2.0E-10	226646	NADH dehydrogenase (ubiquinone) Fe-S protein 2
Ndufs3	1.78	0.83	7.0E-07	3.0E-08	68349	NADH dehydrogenase (ubiquinone) Fe-S protein 3
Ndufs4	1.89	0.92	1.2E-09	3.5E-11	17993	NADH dehydrogenase (ubiquinone) Fe-S protein 4
Ndufs6	1.70	0.76	2.7E-05	1.7E-06	407785	NADH dehydrogenase (ubiquinone) Fe-S protein 6
Ndufs7	1.91	0.93	1.9E-11	4.5E-13	75406	NADH dehydrogenase (ubiquinone) Fe-S protein 7
Ndufs8	1.55	0.64	1.1E-05	6.2E-07	225887	NADH dehydrogenase (ubiquinone) Fe-S protein 8
Ndufv1	1.69	0.76	3.4E-08	1.2E-09	17995	NADH dehydrogenase (ubiquinone) flavoprotein 1
Ndufv2	2.08	1.06	3.6E-13	6.9E-15	72900	NADH dehydrogenase (ubiquinone) flavoprotein 2
Ndufv3	1.96	0.97	6.3E-12	1.4E-13	78330	NADH dehydrogenase (ubiquinone) flavoprotein 3
Neb	3.46	1.79	1.1E-15	1.6E-17	17996	nebulin
Nebl	0.18	-2.46	7.1E-04	6.4E-05	74103	nebulette
Necab2	0.43	-1.22	9.2E-05	6.5E-06	117148	N-terminal EF-hand calcium binding protein 2
Nefl	0.25	-1.98	3.7E-13	7.2E-15	18039	neurofilament, light polypeptide
Nefm	0.29	-1.78	4.7E-10	1.3E-11	18040	neurofilament, medium polypeptide
Nell1	0.60	-0.73	4.7E-03	5.7E-04	338352	NEL-like 1
Nell2	0.49	-1.02	1.3E-03	1.2E-04	54003	NEL-like 2
Neurl1a	1.65	0.73	1.5E-04	1.1E-05	18011	neuralized homolog 1A
Neurl2	2.60	1.38	1.0E-11	2.4E-13	415115	neuralized-like 2
Nexn	2.61	1.38	4.0E-13	7.8E-15	68810	nexilin
Nfatc4	0.64	-0.64	2.9E-05	1.8E-06	73181	nuclear factor of activated T cells, cytoplasmic, calcineurin dependent 4
Nfu1	1.50	0.59	6.9E-03	9.2E-04	56748	NFU1 iron-sulfur cluster scaffold homolog
Ngp	3.97	1.99	1.3E-06	5.8E-08	18054	neutrophilic granule protein
Nid1	1.65	0.72	7.5E-07	3.3E-08	18073	nidogen 1
Ninj1	1.63	0.71	5.6E-05	3.8E-06	18081	ninjurin 1
Nipal2	0.41	-1.30	4.4E-03	5.4E-04	223473	NIPA-like domain containing 2
Nipsnap1	0.63	-0.68	2.0E-03	2.2E-04	18082	4-nitrophenylphosphatase domain and non-neuronal SNAP25-like protein homolog 1
Nkain1	0.58	-0.78	6.8E-06	3.6E-07	67149	Na+/K+ transporting ATPase interacting 1
Nkd1	0.58	-0.79	1.1E-06	4.9E-08	93960	naked cuticle 1 homolog
Nkd2	0.50	-0.99	4.3E-04	3.6E-05	72293	naked cuticle 2 homolog
Nkiras1	1.56	0.65	6.1E-06	3.2E-07	69721	NFKB inhibitor interacting Ras-like protein 1
Nkx6-2	1.71	0.78	2.1E-03	2.3E-04	14912	NK6 homeobox 2
Nlgn3	0.53	-0.91	1.5E-04	1.1E-05	245537	neuroligin 3
Nmnat2	0.48	-1.06	2.0E-04	1.5E-05	226518	nicotinamide nucleotide adenylyltransferase 2
Nmnat3	2.20	1.14	2.5E-03	2.8E-04	74080	nicotinamide nucleotide adenylyltransferase 3
Nmrk2	2.23	1.16	1.0E-03	1.0E-04	69564	nicotinamide riboside kinase 2
Nnat	0.54	-0.89	6.3E-08	2.4E-09	18111	neuronatin
Nnmt	2.27	1.18	3.1E-06	1.5E-07	18113	nicotinamide N-methyltransferase
Nnt	1.97	0.98	2.2E-05	1.3E-06	18115	nicotinamide nucleotide transhydrogenase
Nol3	1.97	0.98	2.0E-09	5.8E-11	78688	nucleolar protein 3 (apoptosis repressor with CARD domain)
Nol9	0.63	-0.67	8.0E-04	7.4E-05	74035	nucleolar protein 9
Nop56	0.61	-0.71	1.8E-07	7.3E-09	67134	NOP56 ribonucleoprotein homolog
Nos1	4.18	2.06	2.8E-14	4.7E-16	18125	nitric oxide synthase 1, neuronal
Npepl1	1.78	0.83	6.9E-10	1.9E-11	228961	aminopeptidase-like 1
Npr1	1.63	0.71	7.7E-05	5.4E-06	18160	natriuretic peptide receptor 1
Nptx2	0.23	-2.10	2.8E-12	6.0E-14	53324	neuronal pentraxin 2
Nqo2	1.52	0.60	4.2E-03	5.0E-04	18105	NAD(P)H dehydrogenase, quinone 2
Nr1d1	1.84	0.88	6.5E-09	2.1E-10	217166	nuclear receptor subfamily 1, group D, member 1
Nr1d2	2.65	1.41	8.9E-16	1.2E-17	353187	nuclear receptor subfamily 1, group D, member 2
Nr1h3	2.56	1.35	1.6E-04	1.2E-05	22259	nuclear receptor subfamily 1, group H, member 3
Nr3c2	1.73	0.79	2.8E-03	3.1E-04	110784	nuclear receptor subfamily 3, group C, member 2
Nr4a1	1.64	0.71	2.4E-06	1.1E-07	15370	nuclear receptor subfamily 4, group A, member 1
Nrap	4.18	2.06	5.8E-22	4.5E-24	18175	nebulin-related anchoring protein
Nrgn	0.55	-0.86	2.6E-03	2.9E-04	64011	neurogranin
Nrk	1.57	0.65	1.6E-03	1.7E-04	27206	Nik related kinase
Nrxn1	0.42	-1.24	1.9E-04	1.5E-05	18189	neurexin I
Nt5c2	1.50	0.59	1.5E-04	1.1E-05	76952	5'-nucleotidase, cytosolic II
Nt5c3	2.18	1.12	2.7E-08	9.5E-10	107569	5'-nucleotidase, cytosolic III
Nt5dc3	1.82	0.86	9.9E-06	5.4E-07	103466	5'-nucleotidase domain containing 3

Nt5e	0.57	-0.82	2.2E-03	2.3E-04	23959	5' nucleotidase, ecto
Ntn3	0.58	-0.78	4.9E-04	4.2E-05	18209	netrin 3
Ntng1	0.51	-0.96	1.8E-03	1.9E-04	80883	netrin G1
Nudt3	1.57	0.65	4.6E-06	2.4E-07	56409	nudix (nucleotide diphosphate linked moiety X)-type motif 3
Nudt4	1.85	0.88	1.8E-09	5.2E-11	71207	nudix (nucleoside diphosphate linked moiety X)-type motif 4
Nyap1	0.55	-0.86	1.8E-03	1.8E-04	243300	neuronal tyrosine-phosphorylated phosphoinositide 3-kinase adaptor 1
Nynrin	0.66	-0.60	3.3E-05	2.1E-06	277154	NYN domain and retroviral integrase containing
Obscn	2.83	1.50	9.7E-15	1.5E-16	380698	obscurin, cytoskeletal calmodulin and titin-interacting RhoGEF
Ocln	0.22	-2.18	7.1E-04	6.4E-05	18260	occludin
Odf3l1	2.32	1.22	2.0E-04	1.5E-05	382075	outer dense fiber of sperm tails 3-like 1
Ogdh	1.76	0.81	3.3E-08	1.2E-09	18293	oxoglutarate dehydrogenase (lipoamide)
Olfm4	7.33	2.87	7.5E-12	1.7E-13	380924	olfactomedin 4
Olfml2a	1.97	0.98	4.8E-04	4.1E-05	241327	olfactomedin-like 2A
Olfr558	2.05	1.04	3.5E-05	2.2E-06	259097	olfactory receptor 558
Oma1	1.64	0.72	1.7E-03	1.7E-04	67013	OMA1 homolog, zinc metallopeptidase
Orai1	1.72	0.78	1.7E-05	9.8E-07	109305	ORAI calcium release-activated calcium modulator 1
Orc1	0.54	-0.88	3.9E-04	3.2E-05	18392	origin recognition complex, subunit 1
Oxld1	2.90	1.54	5.9E-09	1.9E-10	66431	oxidoreductase like domain containing 1
Oxnad1	1.82	0.86	1.4E-06	6.4E-08	218885	oxidoreductase NAD-binding domain containing 1
Oxsm	1.59	0.67	1.8E-03	1.9E-04	71147	3-oxoacyl-ACP synthase, mitochondrial
P2rx5	2.41	1.27	1.2E-06	5.6E-08	94045	purinergic receptor P2X, ligand-gated ion channel, 5
P2rx6	2.73	1.45	2.2E-06	1.1E-07	18440	purinergic receptor P2X, ligand-gated ion channel, 6
P2rx7	1.90	0.93	7.3E-04	6.7E-05	18439	purinergic receptor P2X, ligand-gated ion channel, 7
P2ry1	1.69	0.75	2.3E-03	2.5E-04	18441	purinergic receptor P2Y, G-protein coupled 1
P2ry2	3.00	1.59	2.9E-10	7.8E-12	18442	purinergic receptor P2Y, G-protein coupled 2
Pacrg	2.85	1.51	8.1E-06	4.4E-07	69310	PARK2 co-regulated
Pacsin3	2.45	1.30	2.4E-15	3.5E-17	80708	protein kinase C and casein kinase substrate in neurons 3
Padi2	2.99	1.58	1.6E-23	1.1E-25	18600	peptidyl arginine deiminase, type II
Pak1	2.13	1.09	1.5E-10	3.9E-12	18479	p21 protein (Cdc42/Rac)-activated kinase 1
Pank1	1.51	0.59	2.0E-03	2.2E-04	75735	pantothenate kinase 1
Papss1	0.66	-0.60	1.6E-05	9.3E-07	23971	3'-phosphoadenosine 5'-phosphosulfate synthase 1
Papss2	0.65	-0.62	2.5E-03	2.8E-04	23972	3'-phosphoadenosine 5'-phosphosulfate synthase 2
Paqr5	0.23	-2.11	4.1E-03	4.8E-04	74090	progestin and adipoQ receptor family member V
Pard6g	0.65	-0.63	4.3E-05	2.8E-06	93737	par-6 partitioning defective 6 homolog gamma
Park2	1.71	0.77	6.0E-03	7.8E-04	50873	Parkinson disease (autosomal recessive, juvenile) 2, parkin
Parp10	1.78	0.83	6.4E-03	8.3E-04	671535	poly (ADP-ribose) polymerase family, member 10
Parp14	1.80	0.85	5.4E-04	4.7E-05	547253	poly (ADP-ribose) polymerase family, member 14
Parp4	1.62	0.70	1.2E-04	8.6E-06	328417	poly (ADP-ribose) polymerase family, member 4
Parvb	2.64	1.40	5.1E-18	5.6E-20	170736	parvin, beta
Pbxip1	2.89	1.53	2.5E-22	1.9E-24	229534	pre B cell leukemia transcription factor interacting protein 1
Pcbd1	1.85	0.89	2.0E-03	2.1E-04	13180	pterin 4 alpha carbinolamine dehydratase/dimerization cofactor of hepatocyte nuclear factor 1 alpha (TCF1) 1
Pcdh10	0.25	-1.99	3.0E-11	7.2E-13	18526	protocadherin 10
Pcdh9	0.56	-0.83	2.0E-03	2.2E-04	211712	protocadherin 9
Pcdha1	0.54	-0.90	9.9E-04	9.4E-05	116731	protocadherin alpha 1
Pcdha10	0.55	-0.87	1.5E-03	1.5E-04	12943	protocadherin alpha 10
Pcdha11	0.53	-0.91	7.7E-04	7.0E-05	12942	protocadherin alpha 11
Pcdha12	0.53	-0.93	5.4E-04	4.7E-05	192164	protocadherin alpha 12
Pcdha2	0.54	-0.89	1.1E-03	1.1E-04	353234	protocadherin alpha 2
Pcdha3	0.54	-0.88	1.3E-03	1.2E-04	192163	protocadherin alpha 3
Pcdha4	0.54	-0.89	1.2E-03	1.1E-04	12936	protocadherin alpha 4
Pcdha5	0.54	-0.88	1.1E-03	1.0E-04	12941	protocadherin alpha 5
Pcdha6	0.54	-0.89	1.2E-03	1.1E-04	12937	protocadherin alpha 6
Pcdha7	0.54	-0.89	1.2E-03	1.1E-04	12939	protocadherin alpha 7
Pcdha8	0.54	-0.90	9.2E-04	8.6E-05	353235	protocadherin alpha 8
Pcdha9	0.54	-0.88	1.3E-03	1.2E-04	192161	protocadherin alpha 9
Pcdhac1	0.54	-0.88	1.2E-03	1.2E-04	353236	protocadherin alpha subfamily C, 1
Pcdhac2	0.51	-0.97	6.8E-05	4.6E-06	353237	protocadherin alpha subfamily C, 2
Pcna	0.64	-0.64	5.6E-05	3.7E-06	18538	proliferating cell nuclear antigen
Pcnt	1.65	0.72	1.0E-06	4.6E-08	18541	pericentrin (kendrin)
Pcsk9	0.49	-1.04	4.3E-03	5.1E-04	100102	proprotein convertase subtilisin/kexin type 9
Pcx	1.88	0.91	5.0E-03	6.3E-04	18563	pyruvate carboxylase
Pde4b	2.08	1.06	9.3E-09	3.0E-10	18578	phosphodiesterase 4B, cAMP specific
Pde4d	2.02	1.02	6.0E-15	9.2E-17	238871	phosphodiesterase 4D, cAMP specific
Pde4dip	3.69	1.88	1.8E-24	1.2E-26	83679	phosphodiesterase 4D interacting protein (myomegalin)
Pde6d	1.61	0.69	3.8E-05	2.4E-06	18582	phosphodiesterase 6D, cGMP-specific, rod, delta
Pdgfb	1.54	0.62	1.9E-05	1.1E-06	18591	platelet derived growth factor, B polypeptide
Pdgfc	0.67	-0.59	1.4E-04	1.0E-05	54635	platelet-derived growth factor, C polypeptide
Pdha1	2.17	1.12	4.9E-15	7.5E-17	18597	pyruvate dehydrogenase E1 alpha 1
Pdhb	2.17	1.12	7.5E-16	1.0E-17	68263	pyruvate dehydrogenase (lipoamide) beta
Pdhx	1.84	0.88	6.0E-08	2.2E-09	27402	pyruvate dehydrogenase complex, component X
Pdia4	0.60	-0.75	7.1E-08	2.6E-09	12304	protein disulfide isomerase associated 4
Pdk1	1.84	0.88	8.4E-11	2.1E-12	228026	pyruvate dehydrogenase kinase, isoenzyme 1
Pdk2	3.45	1.78	4.8E-35	9.4E-38	18604	pyruvate dehydrogenase kinase, isoenzyme 2
Pdk4	5.38	2.43	1.7E-48	1.3E-51	27273	pyruvate dehydrogenase kinase, isoenzyme 4
Pdlim1	1.74	0.80	6.0E-08	2.2E-09	54132	PDZ and LIM domain 1 (elfin)
Pdlim3	2.82	1.50	8.4E-18	9.6E-20	53318	PDZ and LIM domain 3
Pdlim5	4.74	2.24	2.5E-26	1.4E-28	56376	PDZ and LIM domain 5
Pdlim7	2.01	1.01	6.6E-10	1.8E-11	67399	PDZ and LIM domain 7
Pdp1	1.63	0.71	1.5E-04	1.1E-05	381511	pyruvate dehyrogenase phosphatase catalytic subunit 1
Pdpk1	1.54	0.62	1.6E-03	1.6E-04	18607	3-phosphoinositide dependent protein kinase 1
Pdpr	1.53	0.61	6.1E-03	8.0E-04	319518	pyruvate dehydrogenase phosphatase regulatory subunit
Pdss2	1.95	0.96	3.2E-05	2.0E-06	71365	prenyl (solanesyl) diphosphate synthase, subunit 2
Pdzd4	0.63	-0.68	2.1E-04	1.6E-05	245469	PDZ domain containing 4
Pdzd9	2.53	1.34	6.5E-07	2.8E-08	67983	PDZ domain containing 9
Pdzk1ip1	0.19	-2.38	4.5E-03	5.5E-04	67182	PDZK1 interacting protein 1
Pecam1	1.60	0.68	4.0E-07	1.7E-08	18613	platelet/endothelial cell adhesion molecule 1
Pemt	3.10	1.63	6.4E-07	2.8E-08	18618	phosphatidylethanolamine N-methyltransferase
Per1	1.54	0.62	9.2E-06	5.1E-07	18626	period homolog 1
Perm1	1.98	0.98	2.0E-10	5.4E-12	74183	PPARGC1 and ESRR induced regulator, muscle 1
Pf4	1.88	0.91	2.3E-07	9.2E-09	56744	platelet factor 4
Pfkfb4	1.64	0.71	3.6E-04	3.0E-05	270198	6-phosphofructo-2-kinase/fructose-2,6-biphosphatase 4
Pfkm	3.42	1.77	6.6E-26	4.0E-28	18642	phosphofructokinase, muscle
Pfkp	1.77	0.82	8.7E-11	2.2E-12	56421	phosphofructokinase, platelet
Pgam2	4.57	2.19	4.2E-29	1.8E-31	56012	phosphoglycerate mutase 2
Pgk1	1.59	0.67	3.5E-06	1.7E-07	18655	phosphoglycerate kinase 1
Pgm2	4.03	2.01	2.8E-37	4.5E-40	72157	phosphoglucomutase 2
Pgpep1	1.59	0.67	3.8E-07	1.6E-08	66522	pyroglutamyl-peptidase I
Phgdh	0.57	-0.81	1.2E-03	1.1E-04	236539	3-phosphoglycerate dehydrogenase
Phka1	1.74	0.80	6.4E-06	3.4E-07	18679	phosphorylase kinase alpha 1
Phkb	1.69	0.76	4.9E-08	1.8E-09	102093	phosphorylase kinase beta
Phkg1	6.97	2.80	7.2E-29	3.1E-31	18682	phosphorylase kinase gamma 1
Phldb3	0.30	-1.73	2.4E-04	1.9E-05	232970	pleckstrin homology-like domain, family B, member 3
Phtf2	2.17	1.12	2.0E-13	3.7E-15	68770	putative homeodomain transcription factor 2
Phyh	1.57	0.65	5.4E-03	6.9E-04	16922	phytanoyl-CoA hydroxylase
Phyhd1	2.45	1.29	1.3E-08	4.4E-10	227696	phytanoyl-CoA dioxygenase domain containing 1
Phyhip	0.33	-1.62	5.1E-03	6.4E-04	105653	phytanoyl-CoA hydroxylase interacting protein
Pi15	1.91	0.94	2.4E-04	1.9E-05	94227	peptidase inhibitor 15
Pi16	2.32	1.21	2.1E-08	7.2E-10	74116	peptidase inhibitor 16
Pianp	0.36	-1.47	1.8E-05	1.1E-06	319352	PILR alpha associated neural protein
Piezo2	0.55	-0.87	6.7E-08	2.5E-09	667742	piezo-type mechanosensitive ion channel component 2
Pigh	0.52	-0.94	1.4E-05	7.7E-07	110417	phosphatidylinositol glycan anchor biosynthesis, class H
Pigp	2.04	1.03	9.9E-09	3.2E-10	56176	phosphatidylinositol glycan anchor biosynthesis, class P
Pik3c2b	1.61	0.69	1.0E-06	4.7E-08	240752	phosphoinositide-3-kinase, class 2, beta polypeptide
Pik3ip1	1.66	0.73	2.6E-03	2.8E-04	216505	phosphoinositide-3-kinase interacting protein 1
Pilra	1.97	0.98	8.3E-04	7.7E-05	231805	paired immunoglobin-like type 2 receptor alpha
Pink1	1.71	0.77	1.1E-07	4.2E-09	68943	PTEN induced putative kinase 1
Pisd-ps1	0.62	-0.69	3.9E-03	4.7E-04	236604	phosphatidylserine decarboxylase, pseudogene 1
Pitpnc1	2.35	1.23	8.5E-19	8.7E-21	71795	phosphatidylinositol transfer protein, cytoplasmic 1
Pitx1	0.47	-1.09	7.3E-05	5.0E-06	18740	paired-like homeodomain transcription factor 1
Pitx2	1.83	0.87	3.6E-05	2.3E-06	18741	paired-like homeodomain transcription factor 2
Pja2	1.89	0.91	8.3E-09	2.7E-10	224938	praja 2, RING-H2 motif containing
Pkhd1	2.04	1.03	1.7E-03	1.7E-04	241035	polycystic kidney and hepatic disease 1
Pkia	2.11	1.08	2.8E-09	8.5E-11	18767	protein kinase inhibitor, alpha
Pkig	1.76	0.82	2.3E-07	9.2E-09	18769	protein kinase inhibitor, gamma
Pkm	1.80	0.85	1.1E-09	3.2E-11	18746	pyruvate kinase, muscle
Pla2g12a	1.82	0.87	1.9E-05	1.1E-06	66350	phospholipase A2, group XIIA
Pla2g16	2.42	1.28	7.7E-16	1.1E-17	225845	phospholipase A2, group XVI
Pla2g3	0.25	-1.99	4.1E-03	5.0E-04	237625	phospholipase A2, group III
Pla2g7	2.50	1.32	4.0E-09	1.2E-10	27226	phospholipase A2, group VII (platelet-activating factor acetylhydrolase, plasma)
Pla2r1	1.99	0.99	5.0E-04	4.3E-05	18779	phospholipase A2 receptor 1
Plcd4	11.60	3.54	2.5E-34	5.5E-37	18802	phospholipase C, delta 4

Plec	1.57	0.65	5.8E-05	3.9E-06	18810	plectin
Plekha8	0.66	-0.60	2.3E-03	2.5E-04	231999	pleckstrin homology domain containing, family A (phosphoinositide binding specific) member 8
Plekhf1	1.62	0.69	5.1E-03	6.3E-04	72287	pleckstrin homology domain containing, family F (with FYVE domain) member 1
Plekhh1	0.41	-1.30	2.7E-12	5.8E-14	211945	pleckstrin homology domain containing, family H (with MyTH4 domain) member 1
Plekho1	1.67	0.74	2.7E-06	1.3E-07	67220	pleckstrin homology domain containing, family O member 1
Plet1	0.26	-1.96	1.7E-04	1.3E-05	76509	placenta expressed transcript 1
Plin4	77.81	6.28	5.0E-55	2.0E-58	57435	perilipin 4
Plin5	31.69	4.99	3.9E-48	3.2E-51	66968	perilipin 5
Pllp	0.17	-2.55	1.4E-19	1.3E-21	67801	plasma membrane proteolipid
Plp1	1.59	0.67	7.0E-03	9.4E-04	18823	proteolipid protein (myelin) 1
Plppr3	0.39	-1.37	3.2E-08	1.1E-09	216152	phospholipid phosphatase related 3
Plscr4	1.80	0.84	6.2E-04	5.4E-05	235527	phospholipid scramblase 4
Plxnb1	0.54	-0.90	2.6E-08	8.9E-10	235611	plexin B1
Plxnb3	1.72	0.78	4.0E-03	4.7E-04	140571	plexin B3
Pm20d2	2.89	1.53	3.1E-14	5.2E-16	242377	peptidase M20 domain containing 2
Pnck	1.89	0.92	8.0E-05	5.6E-06	93843	pregnancy upregulated non-ubiquitously expressed CaM kinase
Pnkd	1.73	0.79	5.0E-04	4.3E-05	56695	paroxysmal nonkinesiogenic dyskinesia
Pnpla2	4.82	2.27	7.5E-09	2.4E-10	66853	patatin-like phospholipase domain containing 2
Podxl	1.56	0.64	5.8E-06	3.0E-07	27205	podocalyxin-like
Pole2	0.66	-0.61	6.6E-03	8.7E-04	18974	polymerase (DNA directed), epsilon 2 (p59 subunit)
Polr3g	0.31	-1.67	1.0E-05	5.5E-07	67486	polymerase (RNA) III (DNA directed) polypeptide G
Pop1	0.58	-0.78	4.8E-05	3.1E-06	67724	processing of precursor 1, ribonuclease P/MRP family
Popdc2	2.60	1.38	6.4E-14	1.1E-15	64082	popeye domain containing 2
Popdc3	3.44	1.78	2.1E-14	3.4E-16	78977	popeye domain containing 3
Ppa2	1.77	0.83	1.8E-06	8.4E-08	74776	pyrophosphatase (inorganic) 2
Ppap2a	1.57	0.65	1.1E-05	6.4E-07	19012	phosphatidic acid phosphatase type 2A
Ppap2c	0.52	-0.93	1.2E-03	1.1E-04	50784	phosphatidic acid phosphatase type 2C
Ppapdc3	2.36	1.24	1.9E-11	4.6E-13	227721	phosphatidic acid phosphatase type 2 domain containing 3
Ppara	6.96	2.80	2.5E-49	1.6E-52	19013	peroxisome proliferator activated receptor alpha
Pparg	4.10	2.04	7.7E-05	5.4E-06	19016	peroxisome proliferator activated receptor gamma
Ppargc1a	3.45	1.78	1.1E-19	1.0E-21	19017	peroxisome proliferative activated receptor, gamma, coactivator 1 alpha
Ppargc1b	2.25	1.17	2.6E-07	1.1E-08	170826	peroxisome proliferative activated receptor, gamma, coactivator 1 beta
Ppfia3	0.35	-1.53	4.4E-03	5.3E-04	76787	protein tyrosine phosphatase, receptor type, f polypeptide (PTPRF), interacting protein (liprin), alpha 3
Ppif	1.52	0.60	1.2E-03	1.2E-04	105675	peptidylprolyl isomerase F (cyclophilin F)
Ppm1b	1.58	0.66	1.2E-04	8.6E-06	19043	protein phosphatase 1B, magnesium dependent, beta isoform
Ppm1j	2.37	1.24	4.2E-06	2.2E-07	71887	protein phosphatase 1J
Ppm1k	2.21	1.15	5.1E-06	2.7E-07	243382	protein phosphatase 1K (PP2C domain containing)
Ppp1r12b	3.60	1.85	5.6E-22	4.4E-24	329251	protein phosphatase 1, regulatory (inhibitor) subunit 12B
Ppp1r13l	0.43	-1.22	1.3E-04	9.8E-06	333654	protein phosphatase 1, regulatory (inhibitor) subunit 13 like
Ppp1r14c	3.56	1.83	2.5E-29	1.0E-31	76142	protein phosphatase 1, regulatory (inhibitor) subunit 14c
Ppp1r1a	3.20	1.68	8.7E-13	1.8E-14	58200	protein phosphatase 1, regulatory (inhibitor) subunit 1A
Ppp1r27	3.88	1.95	2.4E-13	4.6E-15	68701	protein phosphatase 1, regulatory subunit 27
Ppp1r3a	4.37	2.13	2.1E-21	1.7E-23	140491	protein phosphatase 1, regulatory (inhibitor) subunit 3A
Ppp1r3c	2.13	1.09	3.3E-12	7.1E-14	53412	protein phosphatase 1, regulatory (inhibitor) subunit 3C
Ppp1r3d	1.98	0.99	2.2E-06	1.1E-07	228966	protein phosphatase 1, regulatory subunit 3D
Ppp2r3a	2.53	1.34	2.2E-14	3.5E-16	235542	protein phosphatase 2, regulatory subunit B'', alpha
Pprc1	0.65	-0.61	8.9E-06	4.9E-07	226169	peroxisome proliferative activated receptor, gamma, coactivator-related 1
Pptc7	1.55	0.64	6.7E-05	4.6E-06	320717	PTC7 protein phosphatase homolog
Prdm11	0.53	-0.91	2.5E-03	2.7E-04	100042784	PR domain containing 11
Prdx3	1.62	0.69	1.1E-06	4.9E-08	11757	peroxiredoxin 3
Prelid2	1.80	0.85	7.9E-04	7.2E-05	77619	PRELI domain containing 2
Prepl	2.86	1.51	1.2E-15	1.7E-17	213760	prolyl endopeptidase-like
Prkaa2	3.20	1.68	3.0E-17	3.6E-19	108079	protein kinase, AMP-activated, alpha 2 catalytic subunit
Prkab2	2.00	1.00	3.1E-12	6.7E-14	108097	protein kinase, AMP-activated, beta 2 non-catalytic subunit
Prkaca	2.58	1.37	9.7E-17	1.2E-18	18747	protein kinase, cAMP dependent, catalytic, alpha
Prkag3	1.88	0.91	1.9E-06	8.9E-08	241113	protein kinase, AMP-activated, gamma 3 non-catatlytic subunit
Prkcq	2.44	1.29	8.9E-11	2.2E-12	18761	protein kinase C, theta
Prob1	1.89	0.92	3.8E-08	1.4E-09	381148	proline rich basic protein 1
Procr	1.74	0.80	5.8E-04	5.1E-05	19124	protein C receptor, endothelial
Prr16	0.55	-0.86	2.3E-03	2.4E-04	71373	proline rich 16
Prr32	2.54	1.34	3.0E-12	6.5E-14	68800	proline rich 32
Prr33	3.36	1.75	1.3E-21	1.1E-23	677289	proline rich 33
Prrg4	0.36	-1.47	1.9E-03	2.0E-04	228413	proline rich Gla (G-carboxyglutamic acid) 4 (transmembrane)
Prrx2	0.50	-1.01	5.2E-05	3.5E-06	20204	paired related homeobox 2
Prune	1.96	0.97	1.2E-10	3.0E-12	229589	prune homolog
Psca	0.05	-4.43	2.8E-17	3.4E-19	72373	prostate stem cell antigen
Pstpip2	2.62	1.39	5.9E-16	8.0E-18	19201	proline-serine-threonine phosphatase-interacting protein 2
Ptges2	2.30	1.20	1.9E-08	6.4E-10	96979	prostaglandin E synthase 2
Ptges3l	2.48	1.31	2.3E-16	2.9E-18	73635	prostaglandin E synthase 3 (cytosolic)-like
Ptgr2	2.36	1.24	2.4E-15	3.6E-17	77219	prostaglandin reductase 2
Ptma	0.64	-0.64	4.3E-04	3.6E-05	19231	prothymosin alpha
Ptp4a3	2.11	1.08	1.3E-14	2.0E-16	19245	protein tyrosine phosphatase 4a3
Ptpla	3.11	1.64	1.2E-22	8.7E-25	30963	protein tyrosine phosphatase-like (proline instead of catalytic arginine), member a
Ptplb	0.59	-0.75	3.3E-03	3.8E-04	70757	protein tyrosine phosphatase-like (proline instead of catalytic arginine), member b
Ptpn13	0.52	-0.93	4.2E-06	2.2E-07	19249	protein tyrosine phosphatase, non-receptor type 13
Ptpn7	0.57	-0.81	5.4E-03	6.9E-04	320139	protein tyrosine phosphatase, non-receptor type 7
Ptprb	2.17	1.12	1.1E-13	1.9E-15	19263	protein tyrosine phosphatase, receptor type, B
Ptprf	0.42	-1.27	2.8E-07	1.2E-08	19268	protein tyrosine phosphatase, receptor type, F
Ptprt	0.31	-1.67	3.0E-05	1.9E-06	19281	protein tyrosine phosphatase, receptor type, T
Ptpru	0.44	-1.19	1.8E-05	1.0E-06	19273	protein tyrosine phosphatase, receptor type, U
Ptx3	0.65	-0.63	4.3E-03	5.2E-04	19288	pentraxin related gene
Pvalb	41.10	5.36	3.4E-37	5.5E-40	19293	parvalbumin
Pvr	1.85	0.89	6.8E-07	3.0E-08	52118	poliovirus receptor
Pvrl1	0.55	-0.86	3.0E-06	1.5E-07	58235	poliovirus receptor-related 1
Pygm	6.01	2.59	7.0E-39	1.1E-41	19309	muscle glycogen phosphorylase
Qdpr	1.63	0.70	1.4E-06	6.6E-08	110391	quinoid dihydropteridine reductase
Qpct	2.35	1.23	5.8E-11	1.4E-12	70536	glutaminyl-peptide cyclotransferase (glutaminyl cyclase)
Rab11fip4	0.50	-1.01	1.1E-03	1.1E-04	268451	RAB11 family interacting protein 4 (class II)
Rab38	0.39	-1.37	2.6E-03	2.9E-04	72433	RAB38, member of RAS oncogene family
Rab3a	2.40	1.26	9.1E-11	2.3E-12	19339	RAB3A, member RAS oncogene family
Rab3d	0.55	-0.87	6.7E-04	6.0E-05	19340	RAB3D, member RAS oncogene family
Rab3ip	1.59	0.67	1.8E-04	1.4E-05	216363	RAB3A interacting protein
Rab40b	2.66	1.41	1.9E-16	2.4E-18	217371	Rab40b, member RAS oncogene family
Rabep2	1.53	0.61	5.5E-04	4.8E-05	70314	rabaptin, RAB GTPase binding effector protein 2
Rac3	0.46	-1.13	3.3E-05	2.0E-06	170758	RAS-related C3 botulinum substrate 3
Rad23a	1.52	0.60	2.7E-05	1.7E-06	19358	RAD23a homolog
Rai2	2.25	1.17	1.1E-09	3.1E-11	24004	retinoic acid induced 2
Ramp1	3.67	1.88	2.7E-16	3.5E-18	51801	receptor (calcitonin) activity modifying protein 1
Rapgefl1	0.44	-1.19	5.2E-04	4.5E-05	268480	Rap guanine nucleotide exchange factor (GEF)-like 1
Rarg	0.59	-0.77	7.2E-06	3.9E-07	19411	retinoic acid receptor, gamma
Rarres2	2.09	1.07	7.3E-08	2.8E-09	71660	retinoic acid receptor responder (tazarotene induced) 2
Rasgrp2	1.75	0.81	8.3E-05	5.8E-06	19395	RAS, guanyl releasing protein 2
Rassf3	1.59	0.67	1.1E-04	8.0E-06	192678	Ras association (RalGDS/AF-6) domain family member 3
Rassf4	1.98	0.99	1.2E-10	3.0E-12	213391	Ras association (RalGDS/AF-6) domain family member 4
Rbfox1	3.14	1.65	3.5E-19	3.5E-21	268859	RNA binding protein, fox-1 homolog 1
Rbm14	0.66	-0.61	2.1E-05	1.3E-06	56275	RNA binding motif protein 14
Rbm15b	0.65	-0.61	3.4E-05	2.1E-06	109095	RNA binding motif protein 15B
Rbm24	1.94	0.95	1.3E-07	5.1E-09	666794	RNA binding motif protein 24
Rbm38	2.54	1.34	1.1E-15	1.5E-17	56190	RNA binding motif protein 38
Rbmxl1	0.64	-0.64	2.1E-04	1.6E-05	19656	RNA binding motif protein, X linked-like-1
Rcan2	1.81	0.86	2.9E-04	2.3E-05	53901	regulator of calcineurin 2
Rcc2	0.59	-0.77	8.8E-06	4.8E-07	108911	regulator of chromosome condensation 2
Rcsd1	2.95	1.56	1.9E-24	1.3E-26	226594	RCSD domain containing 1
Rdh14	1.68	0.75	5.6E-05	3.8E-06	105014	retinol dehydrogenase 14 (all-trans and 9-cis)
Recql4	0.59	-0.77	1.7E-03	1.7E-04	79456	RecQ protein-like 4
Reep1	1.95	0.96	4.7E-08	1.7E-09	52250	receptor accessory protein 1
Reep4	0.59	-0.76	9.9E-04	9.4E-05	72549	receptor accessory protein 4
Retnlg	25.28	4.66	1.1E-18	1.1E-20	245195	resistin like gamma
Retsat	2.07	1.05	1.3E-08	4.1E-10	67442	retinol saturase (all trans retinol 13,14 reductase)
Rfng	0.60	-0.74	4.7E-05	3.1E-06	19719	RFNG O-fucosylpeptide 3-beta-N-acetylglucosaminyltransferase
Rgcc	1.62	0.70	3.1E-03	3.6E-04	66214	regulator of cell cycle
Rgs4	1.69	0.76	6.3E-03	8.2E-04	19736	regulator of G-protein signaling 4
Rgs5	1.59	0.67	1.6E-05	9.5E-07	19737	regulator of G-protein signaling 5
Rhbdl1	1.90	0.93	8.0E-05	5.6E-06	214951	rhomboid, veinlet-like 1
Rhod	0.62	-0.69	3.4E-03	3.9E-04	11854	ras homolog gene family, member D
Rhof	0.54	-0.88	2.4E-03	2.6E-04	23912	ras homolog gene family, member f
Rhpn1	0.40	-1.33	3.9E-03	4.6E-04	14787	rhophilin, Rho GTPase binding protein 1
Rian	1.87	0.90	2.5E-10	6.6E-12	75745	RNA imprinted and accumulated in nucleus
Rilp	7.93	2.99	1.4E-19	1.3E-21	280408	Rab interacting lysosomal protein
Rilpl1	1.85	0.89	1.7E-10	4.4E-12	75695	Rab interacting lysosomal protein-like 1
Rimklb	0.21	-2.24	7.9E-10	2.2E-11	108653	ribosomal modification protein rimK-like family member B

Ripply3	2.18	1.12	1.9E-05	1.1E-06	170765	ripply3 homolog (zebrafish)
Rmdn1	1.83	0.87	1.0E-04	7.3E-06	66302	regulator of microtubule dynamics 1
Rnf123	1.64	0.72	1.9E-08	6.5E-10	84585	ring finger protein 123
Rnf128	2.75	1.46	4.8E-11	1.2E-12	66889	ring finger protein 128
Rnf217	1.61	0.69	4.6E-05	3.0E-06	268291	ring finger protein 217
Rnf26	0.64	-0.64	4.3E-05	2.8E-06	213211	ring finger protein 26
Rnf43	0.25	-1.98	2.8E-04	2.2E-05	207742	ring finger protein 43
Rock2	1.75	0.81	3.1E-07	1.3E-08	19878	Rho-associated coiled-coil containing protein kinase 2
Rorc	2.22	1.15	3.6E-04	2.9E-05	19885	RAR-related orphan receptor gamma
Rph3al	2.65	1.40	9.8E-07	4.4E-08	380714	rabphilin 3A-like (without C2 domains)
Rpl10a	0.57	-0.82	2.9E-03	3.2E-04	19896	ribosomal protein L10A
Rpl3l	71.46	6.16	9.8E-41	1.4E-43	66211	ribosomal protein L3-like
Rpn1	0.65	-0.63	7.9E-06	4.3E-07	103963	ribophorin I
Rps6ka3	1.60	0.68	4.4E-05	2.9E-06	110651	ribosomal protein S6 kinase polypeptide 3
Rrad	1.63	0.70	2.1E-03	2.2E-04	56437	Ras-related associated with diabetes
Rragd	2.05	1.03	4.3E-08	1.5E-09	52187	Ras-related GTP binding D
Rras	2.51	1.33	9.8E-23	7.2E-25	20130	Harvey rat sarcoma oncogene, subgroup R
Rsph3b	1.72	0.78	6.7E-03	8.8E-04	100037282	radial spoke 3B homolog
Rsu1	1.56	0.64	5.1E-06	2.7E-07	20163	Ras suppressor protein 1
Rtkn2	1.78	0.83	5.1E-04	4.4E-05	170799	rhotekin 2
Rtl1	2.83	1.50	3.7E-19	3.7E-21	353326	retrotransposon-like 1
Rtn2	2.99	1.58	1.1E-20	1.0E-22	20167	reticulon 2 (Z-band associated protein)
Rtn4	1.78	0.83	3.3E-07	1.4E-08	68585	reticulon 4
Rtn4ip1	1.70	0.77	5.7E-04	5.0E-05	170728	reticulon 4 interacting protein 1
Rtn4r	0.45	-1.16	8.7E-05	6.1E-06	65079	reticulon 4 receptor
Rtp4	2.39	1.26	4.0E-04	3.3E-05	67775	receptor transporter protein 4
Runx2	0.64	-0.64	3.1E-03	3.5E-04	12393	runt related transcription factor 2
Runx3	0.46	-1.11	1.2E-05	6.8E-07	12399	runt related transcription factor 3
Rusc2	1.98	0.99	1.1E-09	3.0E-11	100213	RUN and SH3 domain containing 2
Rxrg	1.74	0.80	2.0E-05	1.2E-06	20183	retinoid X receptor gamma
Rybp	0.64	-0.64	1.3E-03	1.3E-04	56353	RING1 and YY1 binding protein
Ryr1	2.39	1.26	2.6E-13	5.0E-15	20190	ryanodine receptor 1, skeletal muscle
Ryr3	2.69	1.43	8.7E-14	1.5E-15	20192	ryanodine receptor 3
S100a8	6.91	2.79	5.1E-12	1.1E-13	20201	S100 calcium binding protein A8 (calgranulin A)
S100a9	6.40	2.68	3.2E-14	5.4E-16	20202	S100 calcium binding protein A9 (calgranulin B)
Sacs	2.48	1.31	1.2E-05	6.7E-07	50720	sacsin
Samd10	0.52	-0.93	6.4E-03	8.4E-04	229011	sterile alpha motif domain containing 10
Samd5	0.50	-0.99	5.6E-04	4.8E-05	320825	sterile alpha motif domain containing 5
Samd9l	1.98	0.99	1.2E-05	6.8E-07	209086	sterile alpha motif domain containing 9-like
Samm50	1.63	0.71	1.1E-07	4.1E-09	68653	sorting and assembly machinery component 50 homolog
Scarb2	1.50	0.59	8.0E-06	4.3E-07	12492	scavenger receptor class B, member 2
Sccpdh	2.52	1.33	8.4E-18	9.6E-20	109232	saccharopine dehydrogenase (putative)
Scfd2	2.25	1.17	5.6E-12	1.2E-13	212986	Sec1 family domain containing 2
Schip1	2.72	1.44	2.5E-14	4.1E-16	30953	schwannomin interacting protein 1
Scn1b	1.98	0.99	3.0E-05	1.8E-06	20266	sodium channel, voltage-gated, type I, beta
Scn3a	2.57	1.36	1.3E-06	5.7E-08	20269	sodium channel, voltage-gated, type III, alpha
Scn3b	1.56	0.64	2.4E-03	2.6E-04	235281	sodium channel, voltage-gated, type III, beta
Scn4a	3.21	1.68	8.4E-25	5.5E-27	110880	sodium channel, voltage-gated, type IV, alpha
Scn4b	7.29	2.87	3.1E-33	8.4E-36	399548	sodium channel, type IV, beta
Scn5a	2.36	1.24	3.9E-08	1.4E-09	20271	sodium channel, voltage-gated, type V, alpha
Scn7a	3.79	1.92	2.1E-06	9.9E-08	20272	sodium channel, voltage-gated, type VII, alpha
Scnn1a	0.25	-2.02	4.9E-05	3.2E-06	20276	sodium channel, nonvoltage-gated 1 alpha
Scube1	0.43	-1.21	2.2E-09	6.6E-11	64706	signal peptide, CUB domain, EGF-like 1
Scube3	0.45	-1.14	7.9E-06	4.3E-07	268935	signal peptide, CUB domain, EGF-like 3
Sdc1	0.50	-0.99	2.3E-10	6.2E-12	20969	syndecan 1
Sdf2l1	0.43	-1.21	1.1E-07	4.4E-09	64136	stromal cell-derived factor 2-like 1
Sdha	2.08	1.05	2.5E-13	4.6E-15	66945	succinate dehydrogenase complex, subunit A, flavoprotein (Fp)
Sdhb	2.03	1.02	1.2E-10	3.2E-12	67680	succinate dehydrogenase complex, subunit B, iron sulfur (Ip)
Sdhc	1.73	0.79	4.4E-09	1.4E-10	66052	succinate dehydrogenase complex, subunit C, integral membrane protein
Sdhd	2.08	1.06	1.1E-13	2.0E-15	66925	succinate dehydrogenase complex, subunit D, integral membrane protein
Sec14l5	3.15	1.66	1.3E-08	4.4E-10	665119	SEC14-like 5
Sec31b	2.40	1.26	3.6E-05	2.3E-06	240667	Sec31 homolog B
Sec61a1	0.66	-0.60	1.7E-05	1.0E-06	53421	Sec61 alpha 1 subunit
Sel1l3	2.98	1.58	1.5E-20	1.3E-22	231238	sel-1 suppressor of lin-12-like 3
Sema3f	0.47	-1.10	2.8E-10	7.6E-12	20350	sema domain, immunoglobulin domain (Ig), short basic domain, secreted, (semaphorin) 3F
Sema4a	0.40	-1.32	2.7E-03	3.0E-04	20351	sema domain, immunoglobulin domain (Ig), transmembrane domain (TM) and short cytoplasmic domain, (semaphorin) 4A
Sema4g	0.54	-0.88	8.0E-04	7.3E-05	26456	sema domain, immunoglobulin domain (Ig), transmembrane domain (TM) and short cytoplasmic domain, (semaphorin) 4G
Sept3	0.47	-1.09	3.4E-05	2.1E-06	24050	septin 3
Sept4	1.62	0.69	3.3E-05	2.0E-06	18952	septin 4
Sept5	0.49	-1.04	9.7E-09	3.1E-10	18951	septin 5
Serpina3j	0.00	-10.63	7.7E-35	1.6E-37	238395	serine (or cysteine) peptidase inhibitor, clade A (alpha-1 antiproteinase, antitrypsin), member 3J
Serpinb11	0.04	-4.81	4.1E-15	6.2E-17	66957	serine (or cysteine) peptidase inhibitor, clade B (ovalbumin), member 11
Serpinb1a	1.71	0.78	3.6E-05	2.3E-06	66222	serine (or cysteine) peptidase inhibitor, clade B, member 1a
Serping1	1.61	0.69	1.4E-05	8.3E-07	12258	serine (or cysteine) peptidase inhibitor, clade G, member 1
Sesn2	0.56	-0.83	6.0E-03	7.7E-04	230784	sestrin 2
Sez6l	0.47	-1.10	3.7E-03	4.3E-04	56747	seizure related 6 homolog like
Sf3b4	0.66	-0.60	4.2E-03	5.0E-04	107701	splicing factor 3b, subunit 4
Sfrp2	0.49	-1.04	1.6E-09	4.6E-11	20319	secreted frizzled-related protein 2
Sgca	2.87	1.52	7.1E-21	6.2E-23	20391	sarcoglycan, alpha (dystrophin-associated glycoprotein)
Sgcb	1.70	0.77	3.2E-06	1.6E-07	24051	sarcoglycan, beta (dystrophin-associated glycoprotein)
Sgcd	1.68	0.75	2.3E-03	2.4E-04	24052	sarcoglycan, delta (dystrophin-associated glycoprotein)
Sgcg	2.65	1.41	2.6E-14	4.2E-16	24053	sarcoglycan, gamma (dystrophin-associated glycoprotein)
Sgms1	1.51	0.60	1.1E-05	6.2E-07	208449	sphingomyelin synthase 1
Sgsm1	0.57	-0.80	3.7E-03	4.4E-04	52850	small G protein signaling modulator 1
Sh3bgr	4.37	2.13	4.7E-30	1.8E-32	50795	SH3-binding domain glutamic acid-rich protein
Sh3bgrl2	0.44	-1.18	2.9E-04	2.3E-05	212531	SH3 domain binding glutamic acid-rich protein like 2
Sh3bp1	0.61	-0.71	1.1E-04	7.8E-06	20401	SH3-domain binding protein 1
Sh3bp5	1.62	0.69	2.5E-08	8.6E-10	24056	SH3-domain binding protein 5 (BTK-associated)
Sh3gl2	0.33	-1.60	2.4E-07	9.7E-09	20404	SH3-domain GRB2-like 2
Sh3yl1	0.56	-0.84	2.7E-03	3.0E-04	24057	Sh3 domain YSC-like 1
Shank1	0.62	-0.68	2.7E-03	3.0E-04	243961	SH3/ankyrin domain gene 1
Shisa2	1.52	0.61	2.0E-04	1.5E-05	219134	shisa homolog 2
Shisa4	2.57	1.36	3.5E-14	5.9E-16	77552	shisa homolog 4
Shisa6	0.56	-0.83	5.2E-03	6.5E-04	380702	shisa homolog 6
Shisa7	0.12	-3.04	1.3E-08	4.1E-10	232813	shisa homolog 7
Shkbp1	0.60	-0.75	1.3E-03	1.3E-04	192192	Sh3kbp1 binding protein 1
Shox2	0.60	-0.73	3.0E-03	3.4E-04	20429	short stature homeobox 2
Shroom1	0.51	-0.96	5.2E-03	6.6E-04	71774	shroom family member 1
Siglec1	3.15	1.65	1.1E-13	2.0E-15	20612	sialic acid binding Ig-like lectin 1, sialoadhesin
Sim1	2.21	1.15	7.0E-04	6.3E-05	20464	single-minded homolog 1
Sirt3	1.93	0.95	2.8E-06	1.4E-07	64384	sirtuin 3 (silent mating type information regulation 2, homolog) 3
Skp2	0.65	-0.63	5.1E-05	3.4E-06	27401	S-phase kinase-associated protein 2 (p45)
Slain1	0.41	-1.28	4.2E-04	3.4E-05	105439	SLAIN motif family, member 1
Slain2	1.58	0.66	3.7E-06	1.8E-07	75991	SLAIN motif family, member 2
Slc14a1	0.53	-0.92	5.0E-04	4.3E-05	108052	solute carrier family 14 (urea transporter), member 1
Slc16a10	1.57	0.65	7.8E-04	7.2E-05	72472	solute carrier family 16 (monocarboxylic acid transporters), member 10
Slc16a12	1.80	0.85	1.6E-03	1.6E-04	240638	solute carrier family 16 (monocarboxylic acid transporters), member 12
Slc16a13	0.52	-0.94	1.7E-03	1.7E-04	69309	solute carrier family 16 (monocarboxylic acid transporters), member 13
Slc16a3	3.04	1.60	1.4E-30	4.9E-33	80879	solute carrier family 16 (monocarboxylic acid transporters), member 3
Slc18a1	3.16	1.66	1.4E-07	5.4E-09	110877	solute carrier family 18 (vesicular monoamine), member 1
Slc1a2	0.47	-1.10	2.3E-05	1.4E-06	20511	solute carrier family 1 (glial high affinity glutamate transporter), member 2
Slc20a2	1.61	0.69	1.1E-06	5.0E-08	20516	solute carrier family 20, member 2
Slc22a23	0.59	-0.75	6.0E-03	7.7E-04	73102	solute carrier family 22, member 23
Slc22a3	0.39	-1.37	1.2E-06	5.4E-08	20519	solute carrier family 22 (organic cation transporter), member 3
Slc25a1	0.66	-0.59	4.1E-03	5.0E-04	13358	solute carrier family 25 (mitochondrial carrier, citrate transporter), member 1
Slc25a11	1.92	0.94	2.4E-12	5.2E-14	67863	solute carrier family 25 (mitochondrial carrier oxoglutarate carrier), member 11
Slc25a12	3.37	1.75	5.4E-32	1.7E-34	78830	solute carrier family 25 (mitochondrial carrier, Aralar), member 12
Slc25a20	2.31	1.21	1.1E-13	2.0E-15	57279	solute carrier family 25 (mitochondrial carnitine/acylcarnitine translocase), member 20
Slc25a3	1.57	0.65	6.2E-06	3.3E-07	18674	solute carrier family 25 (mitochondrial carrier, phosphate carrier), member 3
Slc25a34	10.90	3.45	4.6E-36	7.5E-39	384071	solute carrier family 25, member 34
Slc25a37	0.52	-0.96	7.8E-09	2.5E-10	67712	solute carrier family 25, member 37
Slc25a4	2.99	1.58	4.7E-28	2.2E-30	11739	solute carrier family 25 (mitochondrial carrier, adenine nucleotide translocator), member 4
Slc26a2	0.48	-1.07	1.1E-05	5.9E-07	13521	solute carrier family 26 (sulfate transporter), member 2
Slc26a7	0.39	-1.35	1.9E-05	1.1E-06	208890	solute carrier family 26, member 7
Slc27a3	0.59	-0.75	1.9E-04	1.4E-05	26568	solute carrier family 27 (fatty acid transporter), member 3
Slc29a2	1.56	0.64	4.8E-03	5.9E-04	13340	solute carrier family 29 (nucleoside transporters), member 2
Slc2a1	0.60	-0.74	1.8E-04	1.3E-05	20525	solute carrier family 2 (facilitated glucose transporter), member 1
Slc2a12	1.88	0.91	7.5E-05	5.2E-06	353169	solute carrier family 2 (facilitated glucose transporter), member 12
Slc2a3	0.15	-2.73	7.7E-69	2.0E-72	20527	solute carrier family 2 (facilitated glucose transporter), member 3
Slc2a4	3.66	1.87	1.0E-40	1.4E-43	20528	solute carrier family 2 (facilitated glucose transporter), member 4
Slc30a10	3.17	1.67	4.7E-08	1.7E-09	226781	solute carrier family 30, member 10

Slc30a2	0.29	-1.80	1.4E-11	3.1E-13	230810	solute carrier family 30 (zinc transporter), member 2
Slc35e4	2.14	1.10	6.3E-06	3.3E-07	103710	solute carrier family 35, member E4
Slc36a2	2.81	1.49	2.0E-03	2.1E-04	246049	solute carrier family 36 (proton/amino acid symporter), member 2
Slc37a4	1.91	0.94	2.1E-07	8.4E-09	14385	solute carrier family 37 (glucose-6-phosphate transporter), member 4
Slc39a11	0.62	-0.68	1.2E-03	1.2E-04	69806	solute carrier family 39 (metal ion transporter), member 11
Slc3a1	2.73	1.45	6.8E-16	9.4E-18	20532	solute carrier family 3, member 1
Slc41a1	2.04	1.03	1.6E-14	2.5E-16	98396	solute carrier family 41, member 1
Slc46a1	0.49	-1.03	4.8E-04	4.1E-05	52466	solute carrier family 46, member 1
Slc47a1	3.01	1.59	2.9E-10	7.7E-12	67473	solute carrier family 47, member 1
Slc52a3	0.41	-1.30	3.6E-06	1.8E-07	69698	solute carrier protein family 52, member 3
Slc5a1	0.16	-2.62	1.6E-03	1.6E-04	20537	solute carrier family 5 (sodium/glucose cotransporter), member 1
Slc6a13	2.61	1.39	1.5E-08	4.9E-10	14412	solute carrier family 6 (neurotransmitter transporter, GABA), member 13
Slc6a17	0.51	-0.97	3.0E-05	1.9E-06	229706	solute carrier family 6 (neurotransmitter transporter), member 17
Slc7a11	0.18	-2.47	3.3E-04	2.7E-05	26570	solute carrier family 7 (cationic amino acid transporter, y+ system), member 11
Slc7a2	1.67	0.74	1.6E-07	6.5E-09	11988	solute carrier family 7 (cationic amino acid transporter, y+ system), member 2
Slc7a6	0.66	-0.60	1.2E-04	8.4E-06	330836	solute carrier family 7 (cationic amino acid transporter, y+ system), member 6
Slc8a2	0.40	-1.32	2.7E-08	9.4E-10	110891	solute carrier family 8 (sodium/calcium exchanger), member 2
Slc9a2	5.56	2.48	1.2E-28	5.3E-31	226999	solute carrier family 9 (sodium/hydrogen exchanger), member 2
Slco2b1	1.90	0.93	2.9E-07	1.2E-08	101488	solute carrier organic anion transporter family, member 2b1
Slfn2	1.88	0.91	6.5E-04	5.8E-05	20556	schlafen 2
Slfn5	2.73	1.45	6.7E-12	1.5E-13	327978	schlafen 5
Slfn5os	1.83	0.87	3.5E-04	2.9E-05	76392	schlafen 5, opposite strand
Slit1	2.17	1.12	9.4E-04	8.9E-05	20562	slit homolog 1
Slit2	0.66	-0.61	3.9E-03	4.6E-04	20563	slit homolog 2
Slmap	1.52	0.60	6.2E-05	4.2E-06	83997	sarcolemma associated protein
Sln	5.00	2.32	4.0E-28	1.9E-30	66402	sarcolipin
Smagp	0.50	-1.00	2.8E-03	3.1E-04	207818	small cell adhesion glycoprotein
Smarcd3	1.65	0.72	3.9E-06	2.0E-07	66993	SWI/SNF related, matrix associated, actin dependent regulator of chromatin, subfamily d, member 3
Smim19	1.70	0.76	3.1E-05	1.9E-06	102032	small integral membrane protein 19
Smim20	1.63	0.70	5.4E-05	3.6E-06	66278	small integral membrane protein 20
Smim5	1.66	0.73	1.9E-03	2.0E-04	66528	small integral membrane protein 5
Smoc1	1.59	0.67	3.2E-05	2.0E-06	64075	SPARC related modular calcium binding 1
Smpdl3b	0.61	-0.71	6.1E-03	8.0E-04	100340	sphingomyelin phosphodiesterase, acid-like 3B
Smpx	3.26	1.71	1.9E-18	2.1E-20	66106	small muscle protein, X-linked
Sms	1.79	0.84	2.2E-06	1.1E-07	20603	spermine synthase
Smtn	1.52	0.60	1.2E-04	8.9E-06	29856	smoothelin
Smtnl1	14.03	3.81	4.3E-24	3.0E-26	68678	smoothelin-like 1
Smtnl2	2.10	1.07	6.0E-12	1.3E-13	276829	smoothelin-like 2
Smyd1	1.93	0.95	3.0E-07	1.2E-08	12180	SET and MYND domain containing 1
Sned1	0.55	-0.87	1.5E-04	1.1E-05	208777	sushi, nidogen and EGF-like domains 1
Snta1	3.81	1.93	2.5E-26	1.4E-28	20648	syntrophin, acidic 1
Sntb1	2.34	1.22	3.3E-12	7.3E-14	20649	syntrophin, basic 1
Snx11	0.58	-0.79	1.7E-03	1.8E-04	74479	sorting nexin 11
Snx21	1.83	0.87	3.5E-08	1.2E-09	101113	sorting nexin family member 21
Sobp	1.50	0.59	2.5E-03	2.7E-04	109205	sine oculis-binding protein homolog
Socs2	1.92	0.94	3.5E-05	2.2E-06	216233	suppressor of cytokine signaling 2
Socs7	1.69	0.76	5.9E-06	3.1E-07	192157	suppressor of cytokine signaling 7
Sod2	2.27	1.19	9.8E-16	1.4E-17	20656	superoxide dismutase 2, mitochondrial
Sod3	3.72	1.89	1.1E-10	2.9E-12	20657	superoxide dismutase 3, extracellular
Sorbs1	2.01	1.00	3.6E-13	6.8E-15	20411	sorbin and SH3 domain containing 1
Sos2	1.67	0.74	9.4E-09	3.0E-10	20663	son of sevenless homolog 2
Sox17	1.77	0.82	1.9E-04	1.4E-05	20671	SRY-box containing gene 17
Sox6	1.66	0.73	3.9E-08	1.4E-09	20679	SRY-box containing gene 6
Sox9	0.51	-0.96	6.0E-04	5.3E-05	20682	SRY-box containing gene 9
Sp6	0.15	-2.70	1.5E-03	1.5E-04	83395	trans-acting transcription factor 6
Spag7	1.55	0.63	1.2E-06	5.4E-08	216873	sperm associated antigen 7
Sparcl1	1.97	0.98	3.7E-12	8.1E-14	13602	SPARC-like 1
Speg	2.45	1.29	1.2E-13	2.1E-15	11790	SPEG complex locus
Spint2	0.34	-1.57	1.3E-05	7.6E-07	20733	serine protease inhibitor, Kunitz type 2
Spop	1.68	0.75	5.7E-08	2.1E-09	20747	speckle-type POZ protein
Spp1	1.97	0.98	3.5E-03	4.1E-04	20750	secreted phosphoprotein 1
Sprr1a	0.05	-4.38	1.2E-16	1.6E-18	20753	small proline-rich protein 1A
Sprr3	0.00	-7.65	9.1E-66	3.0E-69	20766	small proline-rich protein 3
Sptb	3.52	1.82	1.5E-24	9.7E-27	20741	spectrin beta, erythrocytic
Sptbn2	0.29	-1.78	1.9E-03	2.0E-04	20743	spectrin beta, non-erythrocytic 2
Sptlc1	0.63	-0.66	1.6E-03	1.6E-04	268656	serine palmitoyltransferase, long chain base subunit 1
Sqle	0.51	-0.96	8.4E-09	2.7E-10	20775	squalene epoxidase
Sqrdl	1.62	0.70	7.3E-03	9.8E-04	59010	sulfide quinone reductase-like
Src	0.66	-0.61	1.2E-05	6.8E-07	20779	Rous sarcoma oncogene
Srd5a1	0.51	-0.98	1.5E-03	1.6E-04	78925	steroid 5 alpha-reductase 1
Srf	1.57	0.65	8.7E-05	6.2E-06	20807	serum response factor
Srl	2.92	1.55	8.7E-15	1.3E-16	106393	sarcalumenin
Srm	0.63	-0.67	1.3E-06	5.8E-08	20810	spermidine synthase
Srpk3	2.09	1.07	1.6E-08	5.4E-10	56504	serine/arginine-rich protein specific kinase 3
Srpx2	1.88	0.91	7.4E-08	2.8E-09	68792	sushi-repeat-containing protein, X-linked 2
Srsf12	0.40	-1.33	1.2E-05	6.8E-07	272009	serine/arginine-rich splicing factor 12
Ssh3	0.36	-1.47	7.9E-06	4.3E-07	245857	slingshot homolog 3
Sspn	2.65	1.41	6.3E-21	5.4E-23	16651	sarcospan
St3gal6	1.66	0.73	3.1E-03	3.5E-04	54613	ST3 beta-galactoside alpha-2,3-sialyltransferase 6
St6galnac4	1.83	0.87	2.6E-08	9.0E-10	20448	ST6 (alpha-N-acetyl-neuraminyl-2,3-beta-galactosyl-1,3)-N-acetylgalactosaminide alpha-2,6-sialyltransferase 4
St6galnac6	1.65	0.72	7.1E-08	2.7E-09	50935	ST6 (alpha-N-acetyl-neuraminyl-2,3-beta-galactosyl-1,3)-N-acetylgalactosaminide alpha-2,6-sialyltransferase 6
St8sia2	0.57	-0.81	1.1E-04	8.0E-06	20450	ST8 alpha-N-acetyl-neuraminide alpha-2,8-sialyltransferase 2
St8sia4	1.51	0.60	4.3E-03	5.3E-04	20452	ST8 alpha-N-acetyl-neuraminide alpha-2,8-sialyltransferase 4
St8sia5	3.43	1.78	6.0E-10	1.7E-11	225742	ST8 alpha-N-acetyl-neuraminide alpha-2,8-sialyltransferase 5
Stac3	1.75	0.81	1.7E-06	8.1E-08	237611	SH3 and cysteine rich domain 3
Stard10	0.53	-0.91	5.9E-03	7.6E-04	56018	START domain containing 10
Stard5	0.55	-0.87	4.3E-03	5.2E-04	170460	StAR-related lipid transfer (START) domain containing 5
Stard7	1.67	0.74	6.3E-08	2.3E-09	99138	START domain containing 7
Stat5a	1.54	0.62	9.1E-05	6.4E-06	20850	signal transducer and activator of transcription 5A
Stat5b	1.57	0.65	2.0E-06	9.5E-08	20851	signal transducer and activator of transcription 5B
Stau2	1.57	0.65	9.2E-04	8.6E-05	29819	staufen (RNA binding protein) homolog 2
Stbd1	4.46	2.16	1.7E-26	9.2E-29	52331	starch binding domain 1
Steap3	1.72	0.79	1.6E-05	9.4E-07	68428	STEAP family member 3
Steap4	1.86	0.90	1.4E-07	5.5E-09	117167	STEAP family member 4
Stim1	2.59	1.37	7.8E-26	4.7E-28	20866	stromal interaction molecule 1
Stim2	1.78	0.83	2.5E-07	1.0E-08	116873	stromal interaction molecule 2
Stk17b	1.88	0.91	8.1E-07	3.6E-08	98267	serine/threonine kinase 17b (apoptosis-inducing)
Stk32b	0.39	-1.36	7.2E-03	9.7E-04	64293	serine/threonine kinase 32B
Stmnd1	2.31	1.20	2.3E-04	1.8E-05	380842	stathmin domain containing 1
Stom	2.23	1.16	2.7E-19	2.6E-21	13830	stomatin
Stra6	0.41	-1.29	1.6E-05	9.0E-07	20897	stimulated by retinoic acid gene 6
Stradb	2.01	1.00	1.8E-09	5.2E-11	227154	STE20-related kinase adaptor beta
Strip2	3.41	1.77	5.7E-11	1.4E-12	320609	striatin interacting protein 2
Sucla2	2.33	1.22	2.8E-16	3.7E-18	20916	succinate-Coenzyme A ligase, ADP-forming, beta subunit
Suclg1	1.98	0.99	2.6E-14	4.2E-16	56451	succinate-CoA ligase, GDP-forming, alpha subunit
Sulf2	0.48	-1.05	4.9E-10	1.3E-11	72043	sulfatase 2
Sult1a1	2.28	1.19	2.9E-07	1.2E-08	20887	sulfotransferase family 1A, phenol-preferring, member 1
Susd4	0.49	-1.04	1.6E-03	1.7E-04	96935	sushi domain containing 4
Suv39h2	0.54	-0.88	7.5E-05	5.2E-06	64707	suppressor of variegation 3-9 homolog 2
Sv2b	1.86	0.90	2.0E-03	2.1E-04	64176	synaptic vesicle glycoprotein 2 b
Svil	1.51	0.59	7.2E-05	5.0E-06	225115	supervillin
Syn2	3.34	1.74	9.5E-08	3.6E-09	20965	synapsin II
Sync	1.66	0.73	5.0E-05	3.3E-06	68828	syncoilin
Synj2	1.76	0.82	1.0E-06	4.5E-08	20975	synaptojanin 2
Synm	3.15	1.65	4.2E-15	6.4E-17	233335	synemin, intermediate filament protein
Synpo2	2.91	1.54	2.9E-16	3.7E-18	118449	synaptopodin 2
Synpo2l	1.95	0.96	1.9E-07	7.4E-09	68760	synaptopodin 2-like
Sypl2	5.76	2.53	5.6E-33	1.6E-35	17306	synaptophysin-like 2
Tacc2	1.66	0.73	5.3E-08	1.9E-09	57752	transforming, acidic coiled-coil containing protein 2
Tagln	1.78	0.83	1.3E-07	5.2E-09	21345	transgelin
Tap2	1.70	0.77	3.4E-03	3.9E-04	21355	transporter 2, ATP-binding cassette, sub-family B (MDR/TAP)
Tarsl2	2.11	1.07	2.1E-11	4.9E-13	272396	threonyl-tRNA synthetase-like 2
Tas1r1	1.83	0.87	4.0E-03	4.7E-04	110326	taste receptor, type 1, member 1
Tbc1d10a	0.64	-0.64	3.2E-03	3.6E-04	103724	TBC1 domain family, member 10a
Tbc1d4	1.59	0.67	2.2E-05	1.4E-06	210789	TBC1 domain family, member 4
Tbx18	0.63	-0.66	3.3E-05	2.1E-06	76365	T-box18
Tbx5	0.39	-1.37	1.5E-05	8.5E-07	21388	T-box 5
Tcaim	1.73	0.79	6.6E-04	5.9E-05	382117	T cell activation inhibitor, mitochondrial
Tcap	4.12	2.04	1.2E-26	6.3E-29	21393	titin-cap
Tcea3	2.70	1.43	3.9E-19	3.9E-21	21401	transcription elongation factor A (SII), 3
Tceal1	1.82	0.86	4.5E-06	2.3E-07	237052	transcription elongation factor A (SII)-like 1

Tceal3	1.79	0.84	2.7E-06	1.3E-07	594844	transcription elongation factor A (SII)-like 3
Tceal5	2.46	1.30	7.6E-13	1.5E-14	331532	transcription elongation factor A (SII)-like 5
Tceal7	3.71	1.89	7.7E-15	1.2E-16	100040972	transcription elongation factor A (SII)-like 7
Tchh	0.44	-1.20	2.5E-05	1.5E-06	99681	trichohyalin
Tcp11l2	2.07	1.05	9.9E-12	2.2E-13	216198	t-complex 11 like 2
Tdrkh	1.66	0.73	4.1E-03	4.9E-04	72634	tudor and KH domain containing protein
Tdrp	3.12	1.64	2.1E-11	5.0E-13	72148	testis development related protein
Tead3	0.65	-0.62	1.3E-03	1.3E-04	21678	TEA domain family member 3
Tecrl	2.44	1.29	8.7E-07	3.9E-08	243078	trans-2,3-enoyl-CoA reductase-like
Tef	1.94	0.95	1.4E-12	2.9E-14	21685	thyrotroph embryonic factor
Tefm	1.56	0.64	6.4E-03	8.4E-04	68550	transcription elongation factor, mitochondrial
Tekt5	2.40	1.26	4.4E-06	2.2E-07	70426	tektin 5
Tenm2	0.38	-1.41	1.6E-03	1.7E-04	23964	teneurin transmembrane protein 2
Tert	0.49	-1.04	1.0E-04	7.2E-06	21752	telomerase reverse transcriptase
Tes	0.50	-1.01	5.8E-05	3.9E-06	21753	testis derived transcript
Tesc	0.28	-1.83	1.4E-03	1.4E-04	57816	tescalcin
Tet2	1.86	0.89	1.0E-10	2.6E-12	214133	tet methylcytosine dioxygenase 2
Tfap2b	0.24	-2.07	6.4E-03	8.4E-04	21419	transcription factor AP-2 beta
Tfdp2	1.56	0.64	3.0E-06	1.5E-07	211586	transcription factor Dp 2
Tfeb	2.37	1.24	2.7E-12	5.9E-14	21425	transcription factor EB
Tfpi2	1.73	0.79	6.4E-03	8.4E-04	21789	tissue factor pathway inhibitor 2
Tfrc	2.02	1.02	2.5E-13	4.7E-15	22042	transferrin receptor
Tgfa	0.36	-1.48	2.1E-04	1.6E-05	21802	transforming growth factor alpha
Tgm2	5.26	2.39	6.1E-45	6.4E-48	21817	transglutaminase 2, C polypeptide
Thrsp	13.51	3.76	1.1E-20	9.9E-23	21835	thyroid hormone responsive
Tiam1	0.46	-1.11	5.6E-04	4.9E-05	21844	T cell lymphoma invasion and metastasis 1
Timeless	0.66	-0.60	3.3E-05	2.0E-06	21853	timeless homolog
Tll2	0.45	-1.15	7.3E-05	5.0E-06	24087	tolloid-like 2
Tln2	1.67	0.74	3.5E-06	1.8E-07	70549	talin 2
Tm6sf1	2.87	1.52	1.1E-17	1.2E-19	107769	transmembrane 6 superfamily member 1
Tm7sf2	1.88	0.91	6.4E-07	2.8E-08	73166	transmembrane 7 superfamily member 2
Tmem108	0.43	-1.21	1.3E-05	7.6E-07	81907	transmembrane protein 108
Tmem116	2.92	1.55	2.8E-09	8.4E-11	77462	transmembrane protein 116
Tmem117	2.83	1.50	3.3E-12	7.2E-14	320709	transmembrane protein 117
Tmem132c	0.46	-1.12	7.5E-05	5.2E-06	208213	transmembrane protein 132C
Tmem143	2.32	1.21	2.9E-10	7.7E-12	70209	transmembrane protein 143
Tmem151b	0.42	-1.25	5.4E-04	4.7E-05	210573	transmembrane protein 151B
Tmem170b	1.63	0.71	1.5E-06	6.8E-08	621976	transmembrane protein 170B
Tmem182	3.43	1.78	3.0E-18	3.2E-20	381339	transmembrane protein 182
Tmem184a	0.37	-1.45	4.8E-04	4.1E-05	231832	transmembrane protein 184a
Tmem200b	0.62	-0.69	4.6E-03	5.6E-04	623230	transmembrane protein 200B
Tmem233	7.44	2.89	1.6E-21	1.3E-23	545798	transmembrane protein 233
Tmem25	2.33	1.22	5.1E-05	3.4E-06	71687	transmembrane protein 25
Tmem252	1.59	0.67	4.7E-03	5.8E-04	226040	transmembrane protein 252
Tmem255a	1.74	0.80	4.9E-09	1.5E-10	245386	ransmembrane protein 255A
Tmem26	0.53	-0.91	1.4E-07	5.3E-09	327766	transmembrane protein 26
Tmem30b	0.50	-1.01	1.8E-06	8.7E-08	238257	transmembrane protein 30B
Tmem37	2.33	1.22	1.2E-06	5.6E-08	170706	transmembrane protein 37
Tmem38a	3.45	1.79	6.9E-27	3.5E-29	74166	transmembrane protein 38A
Tmem38b	2.35	1.23	1.8E-13	3.4E-15	52076	transmembrane protein 38B
Tmem50b	0.57	-0.80	4.6E-05	3.0E-06	77975	transmembrane protein 50B
Tmem51	0.54	-0.88	7.3E-04	6.6E-05	214359	transmembrane protein 51
Tmem52	17.48	4.13	1.0E-32	3.0E-35	69671	transmembrane protein 52
Tmem53	2.04	1.03	9.1E-04	8.5E-05	68777	transmembrane protein 53
Tmem70	1.54	0.62	1.9E-05	1.1E-06	70397	transmembrane protein 70
Tmem72	9.16	3.20	2.6E-19	2.5E-21	319776	transmembrane protein 72
Tmem79	0.28	-1.83	2.8E-03	3.2E-04	71913	transmembrane protein 79
Tmem8c	1.70	0.77	7.7E-05	5.4E-06	66139	transmembrane protein 8C
Tmem97	0.60	-0.74	3.3E-06	1.6E-07	69071	transmembrane protein 97
Tmod1	2.92	1.55	4.0E-17	4.8E-19	21916	tropomodulin 1
Tmod4	6.11	2.61	4.8E-42	6.0E-45	50874	tropomodulin 4
Tmtc1	1.83	0.87	3.8E-06	1.9E-07	387314	transmembrane and tetratricopeptide repeat containing 1
Tnfaip3	1.68	0.75	1.0E-03	9.7E-05	21929	tumor necrosis factor, alpha-induced protein 3
Tnfsf10	2.63	1.40	1.2E-05	6.7E-07	22035	tumor necrosis factor (ligand) superfamily, member 10
Tnik	1.80	0.85	2.7E-06	1.3E-07	665113	TRAF2 and NCK interacting kinase
Tnnc2	5.08	2.34	3.6E-33	9.9E-36	21925	troponin C2, fast
Tnni1	1.55	0.64	5.8E-03	7.4E-04	21952	troponin I, skeletal, slow 1
Tnni2	6.37	2.67	1.5E-35	2.6E-38	21953	troponin I, skeletal, fast 2
Tnnt1	1.69	0.75	3.6E-04	2.9E-05	21955	troponin T1, skeletal, slow
Tnnt3	5.72	2.52	5.9E-31	2.0E-33	21957	troponin T3, skeletal, fast
Tnxb	2.17	1.12	1.2E-11	2.6E-13	81877	tenascin XB
Tob1	1.85	0.88	2.1E-09	6.4E-11	22057	transducer of ErbB-2.1
Tob2	1.71	0.78	1.3E-08	4.2E-10	57259	transducer of ERBB2, 2
Tpbg	0.40	-1.32	9.2E-09	3.0E-10	21983	trophoblast glycoprotein
Tpd52l1	1.73	0.79	1.0E-03	9.7E-05	21987	tumor protein D52-like 1
Tpi1	2.17	1.12	1.8E-15	2.6E-17	21991	triosephosphate isomerase 1
Tpm1	5.03	2.33	1.5E-34	3.3E-37	22003	tropomyosin 1, alpha
Tpm2	3.46	1.79	2.8E-23	2.0E-25	22004	tropomyosin 2, beta
Tppp	2.75	1.46	4.7E-14	8.1E-16	72948	tubulin polymerization promoting protein
Tppp3	2.20	1.14	3.6E-07	1.5E-08	67971	tubulin polymerization-promoting protein family member 3
Tpsg1	0.55	-0.88	2.7E-03	3.0E-04	26945	tryptase gamma 1
Traf3ip3	2.22	1.15	8.1E-14	1.4E-15	215243	TRAF3 interacting protein 3
Traip	0.59	-0.76	5.0E-03	6.3E-04	22036	TRAF-interacting protein
Trak1	1.68	0.75	5.6E-08	2.1E-09	67095	trafficking protein, kinesin binding 1
Trdn	6.42	2.68	8.0E-32	2.5E-34	76757	triadin
Trf	2.24	1.17	4.0E-05	2.6E-06	22041	transferrin
Tril	0.65	-0.61	8.3E-05	5.8E-06	66873	TLR4 interactor with leucine-rich repeats
Trim2	0.53	-0.92	6.5E-05	4.5E-06	80890	tripartite motif-containing 2
Trim35	2.07	1.05	4.4E-11	1.1E-12	66854	tripartite motif-containing 35
Trim47	1.84	0.88	5.8E-09	1.8E-10	217333	tripartite motif-containing 47
Trim54	2.88	1.53	1.2E-18	1.3E-20	58522	tripartite motif-containing 54
Trim62	0.59	-0.75	3.8E-03	4.5E-04	67525	tripartite motif-containing 62
Trim63	2.36	1.24	4.0E-08	1.4E-09	433766	tripartite motif-containing 63
Trim72	2.67	1.42	1.1E-14	1.8E-16	434246	tripartite motif-containing 72
Trmt61a	0.56	-0.83	1.3E-04	9.2E-06	328162	tRNA methyltransferase 61 homolog A
Tro	0.65	-0.62	4.1E-04	3.4E-05	56191	trophinin
Trp53cor1	1.77	0.82	5.1E-03	6.4E-04	100504267	tumor protein p53 pathway corepressor 1
Trp53inp2	3.03	1.60	7.4E-27	3.8E-29	68728	transformation related protein 53 inducible nuclear protein 2
Trp63	0.37	-1.44	5.0E-03	6.2E-04	22061	transformation related protein 63
Trpt1	1.69	0.76	3.6E-03	4.3E-04	107328	tRNA phosphotransferase 1
Trpv4	0.52	-0.93	1.6E-04	1.2E-05	63873	transient receptor potential cation channel, subfamily V, member 4
Tspan12	2.18	1.12	1.0E-11	2.4E-13	269831	tetraspanin 12
Tspan13	1.95	0.96	1.5E-10	3.9E-12	66109	tetraspanin 13
Tspan15	1.63	0.70	7.0E-04	6.4E-05	70423	tetraspanin 15
Tspan17	1.72	0.78	2.1E-03	2.2E-04	74257	tetraspanin 17
Tspan2	1.71	0.77	2.2E-05	1.3E-06	70747	tetraspanin 2
Tspan7	1.58	0.66	2.3E-06	1.1E-07	21912	tetraspanin 7
Tspo	2.06	1.04	1.7E-05	1.0E-06	12257	translocator protein
Tspyl4	0.40	-1.33	6.5E-11	1.6E-12	72480	TSPY-like 4
Tst	2.58	1.37	1.1E-05	6.0E-07	22117	thiosulfate sulfurtransferase, mitochondrial
Tstd2	2.13	1.09	6.0E-12	1.3E-13	272027	thiosulfate sulfurtransferase (rhodanese)-like domain containing 2
Ttc39a	0.47	-1.10	3.1E-04	2.5E-05	230603	tetratricopeptide repeat domain 39A
Ttc39c	0.51	-0.97	5.4E-04	4.7E-05	72747	tetratricopeptide repeat domain 39C
Ttc9	5.02	2.33	3.4E-23	2.5E-25	69480	tetratricopeptide repeat domain 9
Ttn	3.25	1.70	5.9E-13	1.2E-14	22138	titin
Ttpal	0.57	-0.81	2.5E-06	1.2E-07	76080	tocopherol (alpha) transfer protein-like
Ttyh2	1.59	0.67	1.0E-05	5.8E-07	117160	tweety homolog 2
Tuba4a	1.73	0.79	3.2E-08	1.1E-09	22145	tubulin, alpha 4A
Tusc5	2.85	1.51	1.1E-05	6.0E-07	237858	tumor suppressor candidate 5
Twf2	1.63	0.71	3.7E-07	1.6E-08	23999	twinfilin, actin-binding protein, homolog 2
Twist1	0.49	-1.03	9.1E-08	3.4E-09	22160	twist homolog 1
Twist2	0.35	-1.50	5.0E-04	4.3E-05	13345	twist homolog 2
Txlnb	3.00	1.58	8.3E-19	8.5E-21	378431	taxilin beta
Tyro3	0.55	-0.87	2.5E-07	9.9E-09	22174	TYRO3 protein tyrosine kinase 3
Uaca	1.57	0.65	3.4E-05	2.1E-06	72565	uveal autoantigen with coiled-coil domains and ankyrin repeats
Ubac1	2.26	1.18	1.3E-16	1.6E-18	98766	ubiquitin associated domain containing 1
Ube2b	1.54	0.62	6.1E-05	4.1E-06	22210	ubiquitin-conjugating enzyme E2B
Ube2h	1.51	0.59	3.4E-05	2.1E-06	22214	ubiquitin-conjugating enzyme E2H
Ube2ql1	0.37	-1.44	2.8E-06	1.4E-07	76980	ubiquitin-conjugating enzyme E2Q family-like 1
Ubl3	1.58	0.66	1.5E-05	8.5E-07	24109	ubiquitin-like 3
Ubr3	1.58	0.66	6.0E-06	3.2E-07	68795	ubiquitin protein ligase E3 component n-recognin 3

Uck2	0.59	-0.77	1.6E-05	9.2E-07	80914	uridine-cytidine kinase 2
Uckl1	1.80	0.85	3.4E-05	2.1E-06	68556	uridine-cytidine kinase 1-like 1
Uckl1os	3.80	1.92	8.5E-14	1.5E-15	100271841	uridine-cytidine kinase 1-like 1, opposite strand
Ucp3	5.56	2.48	5.1E-14	8.7E-16	22229	uncoupling protein 3 (mitochondrial, proton carrier)
Ufsp1	1.74	0.80	1.1E-03	1.1E-04	70240	UFM1-specific peptidase 1
Ugp2	2.17	1.12	4.7E-13	9.3E-15	216558	UDP-glucose pyrophosphorylase 2
Uhrf1	0.61	-0.72	1.0E-07	4.0E-09	18140	ubiquitin-like, containing PHD and RING finger domains, 1
Unc13d	0.50	-1.01	1.7E-03	1.7E-04	70450	unc-13 homolog D
Unc45b	1.97	0.98	8.3E-08	3.1E-09	217012	unc-45 homolog B
Unc5d	0.22	-2.20	1.7E-10	4.5E-12	210801	unc-5 homolog D
Upk1b	0.03	-5.32	1.0E-95	1.7E-99	22268	uroplakin 1B
Upk3b	0.07	-3.89	6.9E-16	9.5E-18	100647	uroplakin 3B
Upk3bl	0.06	-4.03	4.9E-27	2.4E-29	69665	uroplakin 3B-like
Uqcc1	1.53	0.62	1.5E-05	8.7E-07	56046	ubiquinol-cytochrome c reductase complex assembly factor 1
Uqcr10	1.84	0.88	5.3E-09	1.7E-10	66152	ubiquinol-cytochrome c reductase, complex III subunit X
Uqcr11	2.21	1.14	9.9E-11	2.5E-12	66594	ubiquinol-cytochrome c reductase, complex III subunit XI
Uqcrb	2.01	1.01	3.0E-09	9.2E-11	67530	ubiquinol-cytochrome c reductase binding protein
Uqcrc1	1.83	0.87	1.3E-09	3.8E-11	22273	ubiquinol-cytochrome c reductase core protein 1
Uqcrc2	2.06	1.04	1.7E-14	2.8E-16	67003	ubiquinol cytochrome c reductase core protein 2
Uqcrfs1	2.15	1.10	3.1E-14	5.2E-16	66694	ubiquinol-cytochrome c reductase, Rieske iron-sulfur polypeptide 1
Uqcrh	1.80	0.85	7.1E-10	2.0E-11	66576	ubiquinol-cytochrome c reductase hinge protein
Uqcrq	1.86	0.89	1.4E-09	3.9E-11	22272	ubiquinol-cytochrome c reductase, complex III subunit VII
Ushbp1	1.62	0.70	3.1E-03	3.5E-04	234395	Usher syndrome 1C binding protein 1
Usmg5	1.85	0.89	7.3E-04	6.7E-05	66477	upregulated during skeletal muscle growth 5
Usp13	2.76	1.46	1.6E-14	2.5E-16	72607	ubiquitin specific peptidase 13 (isopeptidase T-3)
Usp2	3.25	1.70	6.0E-24	4.1E-26	53376	ubiquitin specific peptidase 2
Usp28	2.05	1.03	2.0E-10	5.3E-12	235323	ubiquitin specific peptidase 28
Uvrag	1.56	0.64	2.4E-05	1.4E-06	78610	UV radiation resistance associated gene
Vangl2	0.58	-0.79	8.3E-04	7.7E-05	93840	vang-like 2 (van gogh)
Vav3	0.54	-0.89	4.5E-03	5.5E-04	57257	vav 3 oncogene
Vcam1	0.62	-0.69	5.9E-06	3.1E-07	22329	vascular cell adhesion molecule 1
Vdac1	1.77	0.83	1.8E-09	5.5E-11	22333	voltage-dependent anion channel 1
Vdac2	1.79	0.84	1.9E-08	6.4E-10	22334	voltage-dependent anion channel 2
Vdac3	1.51	0.59	3.2E-05	2.0E-06	22335	voltage-dependent anion channel 3
Vegfa	1.52	0.60	4.8E-06	2.5E-07	22339	vascular endothelial growth factor A
Vegfb	1.59	0.67	3.6E-05	2.3E-06	22340	vascular endothelial growth factor B
Vill	0.56	-0.84	2.3E-03	2.4E-04	22351	villin-like
Vldlr	1.86	0.90	2.2E-08	7.4E-10	22359	very low density lipoprotein receptor
Vps13c	1.63	0.70	1.4E-03	1.4E-04	320528	vacuolar protein sorting 13C
Vsig8	0.03	-5.28	7.8E-44	8.9E-47	240916	V-set and immunoglobulin domain containing 8
Vtn	3.00	1.58	3.8E-07	1.6E-08	22370	vitronectin
Vwc2	2.79	1.48	1.6E-11	3.8E-13	319922	von Willebrand factor C domain containing 2
Vwce	0.53	-0.92	1.8E-03	1.8E-04	71768	von Willebrand factor C and EGF domains
Vwf	1.71	0.77	1.3E-07	5.1E-09	22371	Von Willebrand factor homolog
Wbscr17	2.27	1.19	3.1E-19	3.0E-21	212996	Williams-Beuren syndrome chromosome region 17 homolog
Wdr44	1.79	0.84	8.9E-06	4.9E-07	72404	WD repeat domain 44
Wfdc1	6.31	2.66	4.6E-19	4.6E-21	67866	WAP four-disulfide core domain 1
Wipf3	1.67	0.74	4.1E-03	4.9E-04	330319	WAS/WASL interacting protein family, member 3
Wnk2	2.07	1.05	1.2E-14	1.8E-16	75607	WNK lysine deficient protein kinase 2
Wnk4	0.49	-1.04	1.5E-04	1.1E-05	69847	WNK lysine deficient protein kinase 4
Wnt10a	0.37	-1.44	2.8E-03	3.1E-04	22409	wingless related MMTV integration site 10a
Wnt5a	0.59	-0.77	2.3E-08	7.8E-10	22418	wingless-related MMTV integration site 5A
Wnt6	0.36	-1.46	3.2E-04	2.6E-05	22420	wingless-related MMTV integration site 6
Wnt7b	0.18	-2.49	4.4E-04	3.6E-05	22422	wingless-related MMTV integration site 7B
Wscd2	0.39	-1.36	1.8E-06	8.5E-08	320916	WSC domain containing 2
Wwc1	0.37	-1.45	3.0E-04	2.4E-05	211652	WW, C2 and coiled-coil domain containing 1
Wwp2	0.63	-0.66	2.8E-03	3.2E-04	66894	WW domain containing E3 ubiquitin protein ligase 2
Xirp1	3.12	1.64	5.4E-15	8.3E-17	22437	xin actin-binding repeat containing 1
Xirp2	12.13	3.60	2.3E-22	1.7E-24	241431	xin actin-binding repeat containing 2
Xk	2.82	1.50	1.0E-10	2.7E-12	22439	Kell blood group precursor (McLeod phenotype) homolog
Xylb	0.57	-0.82	5.3E-04	4.6E-05	102448	xylulokinase homolog
Ydjc	0.46	-1.13	4.2E-04	3.5E-05	69101	YdjC homolog
Yipf7	5.84	2.55	2.1E-28	9.3E-31	75581	Yip1 domain family, member 7
Zak	2.23	1.16	1.6E-11	3.6E-13	65964	sterile alpha motif and leucine zipper containing kinase AZK
Zbtb16	3.01	1.59	1.1E-13	2.0E-15	235320	zinc finger and BTB domain containing 16
Zbtb18	1.57	0.65	2.9E-04	2.3E-05	30928	zinc finger and BTB domain containing 18
Zc3h6	1.65	0.72	3.5E-03	4.1E-04	78751	zinc finger CCCH type containing 6
Zdbf2	2.05	1.03	8.8E-06	4.8E-07	73884	zinc finger, DBF-type containing 2
Zfp184	0.49	-1.04	1.7E-03	1.8E-04	193452	zinc finger protein 184 (Kruppel-like)
Zfp185	0.45	-1.16	8.6E-13	1.7E-14	22673	zinc finger protein 185
Zfp239	0.49	-1.04	5.0E-04	4.3E-05	22685	zinc finger protein 239
Zfp503	0.63	-0.66	7.7E-06	4.2E-07	218820	zinc finger protein 503
Zfp518b	0.67	-0.59	3.5E-04	2.8E-05	100515	zinc finger protein 518B
Zfp568	1.66	0.73	1.7E-04	1.2E-05	243905	zinc finger protein 568
Zfp57	0.57	-0.80	1.2E-04	8.8E-06	22715	zinc finger protein 57
Zfp599	0.49	-1.04	5.5E-04	4.8E-05	235048	zinc finger protein 599
Zfp641	2.79	1.48	1.0E-08	3.2E-10	239652	zinc finger protein 641
Zfp711	0.59	-0.75	1.8E-03	1.9E-04	245595	zinc finger protein 711
Zim1	1.66	0.73	2.0E-04	1.5E-05	22776	zinc finger, imprinted 1
Zscan18	1.56	0.64	2.5E-03	2.7E-04	232875	zinc finger and SCAN domain containing 18

upregulated (red)
downregulated (blue)

Supplementary Table 1d: Differentially expressed genes in TA of Spin1^{M5} compared to control mice at P21.

Gene symbol	FC (Spin1^{M5} vs. Ctrl)	logFC (Spin1^{M5} vs. Ctrl)	FDR	p-value	Entrez gene	Protein name
1500017E21Rik	2.18	1.13	2.6E-06	7.4E-08	668215	RIKEN cDNA 1500017E21 gene
1700009P17Rik	2.51	1.33	1.1E-04	5.4E-06	75472	RIKEN cDNA 1700009P17 gene
1700016C15Rik	5.46	2.45	1.1E-11	8.1E-14	69428	RIKEN cDNA 1700016C15 gene
1700020I14Rik	0.33	-1.61	2.1E-05	7.9E-07	66602	RIKEN cDNA 1700020I14 gene
1700056E22Rik	4.38	2.13	2.3E-08	3.4E-10	73363	RIKEN cDNA 1700056E22 gene
1700088E04Rik	1.88	0.91	4.4E-06	1.3E-07	27660	RIKEN cDNA 1700088E04 gene
1810041L15Rik	10.91	3.45	1.0E-50	2.0E-54	72301	RIKEN cDNA 1810041L15 gene
1810058I24Rik	2.41	1.27	4.0E-11	3.3E-13	67705	RIKEN cDNA 1810058I24 gene
2010015L04Rik	2.31	1.21	2.6E-05	1.0E-06	544678	RIKEN cDNA 2010015L04 gene
2010300C02Rik	3.07	1.62	3.3E-05	1.3E-06	72097	RIKEN cDNA 2010300C02 gene
2310002L09Rik	0.38	-1.40	6.6E-18	1.8E-20	71886	RIKEN cDNA 2310002L09 gene
2310007L24Rik	1.96	0.97	4.4E-05	1.9E-06	75573	RIKEN cDNA 2310007L24 gene
2310016D03Rik	0.24	-2.07	6.0E-07	1.3E-08	69566	RIKEN cDNA 2310016D03 gene
2310020H05Rik	3.95	1.98	9.3E-20	2.1E-22	69603	RIKEN cDNA 2310020H05 gene
2310022B05Rik	2.89	1.53	2.5E-08	3.6E-10	69551	RIKEN cDNA 2310022B05 gene
2310057M21Rik	2.14	1.10	2.1E-11	1.6E-13	68277	RIKEN cDNA 2310057M21 gene
2310065F04Rik	0.20	-2.30	2.4E-14	1.1E-16	74184	RIKEN cDNA 2310065F04 gene
2310067B10Rik	0.57	-0.80	1.8E-04	1.0E-05	71947	RIKEN cDNA 2310067B10 gene
2610002J02Rik	2.81	1.49	1.9E-05	6.9E-07	67513	RIKEN cDNA 2610002J02 gene
2610507B11Rik	0.59	-0.77	4.8E-06	1.4E-07	72503	RIKEN cDNA 2610507B11 gene
2900079G21Rik	6.30	2.66	9.9E-10	1.1E-11	620760	RIKEN cDNA 2900079G21 gene
3110057O12Rik	0.54	-0.90	2.7E-05	1.1E-06	269423	RIKEN cDNA 3110057O12 gene
3425401B19Rik	0.64	-0.64	4.0E-05	1.7E-06	100504518	RIKEN cDNA 3425401B19 gene
3632451O06Rik	7.90	2.98	9.4E-26	1.4E-28	67419	RIKEN cDNA 3632451O06 gene
4430402I18Rik	5.04	2.33	4.6E-08	7.4E-10	381218	RIKEN cDNA 4430402I18 gene
4833412C05Rik	102.58	6.68	8.5E-94	2.8E-98	73904	RIKEN cDNA 4833412C05 gene
4930413G21Rik	2.51	1.33	1.7E-05	6.1E-07	73951	RIKEN cDNA 4930413G21 gene
4930513N10Rik	2.88	1.53	9.7E-05	4.8E-06	319960	RIKEN cDNA 4930513N10 gene
5330439B14Rik	0.46	-1.11	1.6E-05	5.9E-07	321015	RIKEN cDNA 5330439B14 gene
5730508B09Rik	2.22	1.15	3.8E-06	1.1E-07	70617	RIKEN cDNA 5730508B09 gene
5930412G12Rik	3.10	1.63	6.6E-22	1.3E-24	319616	RIKEN cDNA 5930412G12 gene
6030419C18Rik	1.89	0.92	1.8E-07	3.5E-09	319477	RIKEN cDNA 6030419C18 gene
A230073K19Rik	0.59	-0.75	9.9E-05	4.9E-06	613263	RIKEN cDNA A230073K19 gene
A4galt	4.54	2.18	5.5E-11	4.7E-13	239559	alpha 1,4-galactosyltransferase
A630019I02Rik	0.18	-2.48	1.2E-05	4.0E-07	408254	uncharacterized LOC408254
A930003A15Rik	3.81	1.93	1.7E-12	1.0E-14	68162	RIKEN cDNA A930003A15 gene
Aacs	2.83	1.50	6.0E-10	6.2E-12	78894	acetoacetyl-CoA synthetase
Aatk	2.26	1.18	2.0E-08	2.8E-10	11302	apoptosis-associated tyrosine kinase
Abcc4	0.47	-1.08	1.7E-06	4.4E-08	239273	ATP-binding cassette, sub-family C (CFTR/MRP), member 4
Abcc9	0.56	-0.85	3.2E-08	4.7E-10	20928	ATP-binding cassette, sub-family C (CFTR/MRP), member 9
Abcd1	0.61	-0.71	1.1E-05	3.7E-07	11666	ATP-binding cassette, sub-family D (ALD), member 1
Abcg2	2.36	1.24	1.0E-10	9.2E-13	26357	ATP-binding cassette, sub-family G (WHITE), member 2
Abi2	1.78	0.84	3.6E-05	1.5E-06	329165	abl-interactor 2
Acad11	0.58	-0.80	4.9E-06	1.5E-07	102632	acyl-Coenzyme A dehydrogenase family, member 11
Acadvl	0.58	-0.78	4.0E-06	1.2E-07	11370	acyl-Coenzyme A dehydrogenase, very long chain
Acer2	3.47	1.80	1.6E-04	8.4E-06	230379	alkaline ceramidase 2
Ache	2.19	1.13	1.4E-07	2.6E-09	11423	acetylcholinesterase
Ackr1	4.95	2.31	2.9E-07	5.8E-09	13349	atypical chemokine receptor 1
Acot10	2.67	1.42	1.9E-16	6.8E-19	64833	acyl-CoA thioesterase 10
Acot9	2.45	1.30	3.2E-15	1.3E-17	56360	acyl-CoA thioesterase 9
Acp5	4.51	2.17	1.2E-07	2.3E-09	11433	acid phosphatase 5, tartrate resistant
Actn3	0.55	-0.86	2.4E-06	6.4E-08	11474	actinin alpha 3
Actr3b	3.28	1.71	2.4E-06	6.7E-08	242894	ARP3 actin-related protein 3B
Acvr1b	0.57	-0.81	1.3E-05	4.7E-07	11479	activin A receptor, type 1B
Acvr1c	7.96	2.99	4.7E-12	3.1E-14	269275	activin A receptor, type IC
Adam23	3.56	1.83	7.0E-07	1.6E-08	23792	a disintegrin and metallopeptidase domain 23
Adam33	3.37	1.75	1.0E-08	1.4E-10	110751	a disintegrin and metallopeptidase domain 33
Adamts20	2.30	1.20	6.7E-11	5.8E-13	223838	a disintegrin-like and metallopeptidase (reprolysin type) with thrombospondin type 1 motif, 20
Adap1	10.41	3.38	4.2E-18	1.1E-20	231821	ArfGAP with dual PH domains 1
Adcy1	2.35	1.23	1.2E-08	1.6E-10	432530	adenylate cyclase 1
Adcy9	0.52	-0.94	7.2E-07	1.7E-08	11515	adenylate cyclase 9
Adig	4.37	2.13	1.0E-05	3.5E-07	246747	adipogenin
Adipoq	6.99	2.81	3.6E-11	2.9E-13	11450	adiponectin, C1Q and collagen domain containing
Adk	0.46	-1.14	1.8E-06	4.8E-08	11534	adenosine kinase
Adsl	0.58	-0.78	3.2E-06	9.0E-08	11564	adenylosuccinate lyase
AF357359	5.34	2.42	4.4E-14	2.1E-16	100303647	snoRNA AF357359
Afmid	1.78	0.83	1.7E-04	9.5E-06	71562	arylformamidase
Agap3	1.68	0.75	4.5E-05	1.9E-06	213990	ArfGAP with GTPase domain, ankyrin repeat and PH domain 3
Agbl1	0.25	-2.03	1.4E-15	5.4E-18	244071	ATP/GTP binding protein-like 1
Agl	0.53	-0.92	4.6E-08	7.4E-10	77559	amylo-1,6-glucosidase, 4-alpha-glucanotransferase
Ago3	0.39	-1.35	3.3E-05	1.3E-06	214150	angiotensin II, type I receptor-associated protein
Agtrap	2.49	1.32	6.2E-17	2.0E-19	11610	angiotensin II receptor-associated protein
AI413582	2.66	1.41	9.3E-05	4.5E-06	106672	expressed sequence AI413582
Ak4	4.85	2.28	6.2E-10	6.4E-12	11639	adenylate kinase 4
Akap11	2.07	1.05	3.4E-05	1.4E-06	219181	A kinase (PRKA) anchor protein 11
Akap5	0.26	-1.96	7.1E-06	2.3E-07	238276	A kinase (PRKA) anchor protein 5
Akap9	0.60	-0.74	3.8E-05	1.6E-06	100986	A kinase (PRKA) anchor protein (yotiao) 9
Akirin2	1.62	0.70	1.7E-04	8.9E-06	433693	akirin 2
Alas1	0.63	-0.68	3.8E-05	1.6E-06	11655	aminolevulinic acid synthase 1
Aldh1a1	5.32	2.41	2.4E-36	1.6E-39	11668	aldehyde dehydrogenase family 1, subfamily A1
Aldh1b1	17.89	4.16	5.0E-46	1.5E-49	72535	aldehyde dehydrogenase 1 family, member B1
Aldh4a1	0.60	-0.75	8.6E-06	2.8E-07	212647	aldehyde dehydrogenase 4 family, member A1
Aldh6a1	0.53	-0.91	3.5E-06	1.0E-07	104776	aldehyde dehydrogenase family 6, subfamily A1
Aldoart1	0.59	-0.77	1.2E-04	6.3E-06	353204	aldolase 1 A retrogene 1
Alg10b	0.51	-0.97	6.7E-08	1.1E-09	380959	asparagine-linked glycosylation 10B (alpha-1,2-glucosyltransferase)
Alkbh8	0.51	-0.97	2.4E-06	6.5E-08	67667	alkB, alkylation repair homolog 8 (E. coli)
Alox5ap	2.13	1.09	5.7E-06	1.7E-07	11690	arachidonate 5-lipoxygenase activating protein
Alx4	11.93	3.58	7.4E-21	1.5E-23	11695	aristaless-like homeobox 4
Amhr2	3.16	1.66	6.2E-08	1.1E-09	110542	anti-Mullerian hormone type 2 receptor
Amigo3	0.28	-1.82	1.2E-15	4.6E-18	320844	adhesion molecule with Ig like domain 3
Amot	1.58	0.66	9.2E-06	3.1E-07	27494	angiomotin
Ankhd1	0.57	-0.81	2.1E-05	8.1E-07	108857	ankyrin repeat and KH domain containing 1
Ankrd1	14.19	3.83	9.6E-36	6.6E-39	107765	ankyrin repeat domain 1 (cardiac muscle)
Ankrd23	0.45	-1.14	1.4E-04	7.2E-06	78321	ankyrin repeat domain 23
Ankrd27	1.92	0.94	3.7E-07	7.6E-09	245886	ankyrin repeat domain 27 (VPS9 domain)
Ankrd6	2.16	1.11	1.6E-06	4.2E-08	140577	ankyrin repeat domain 6
Ano4	2.41	1.27	3.8E-05	1.6E-06	320091	anoctamin 4
Ano5	0.52	-0.95	2.6E-07	5.1E-09	233246	anoctamin 5
Anpep	2.01	1.01	3.1E-05	1.2E-06	16790	alanyl (membrane) aminopeptidase
Anxa1	2.38	1.25	1.5E-09	1.7E-11	16952	annexin A1
Anxa2	1.69	0.75	1.4E-04	7.1E-06	12306	annexin A2
Anxa4	2.68	1.42	4.4E-08	7.0E-10	11746	annexin A4
Anxa7	2.56	1.36	1.2E-07	2.2E-09	11750	annexin A7
Anxa8	55.46	5.79	2.6E-17	8.0E-20	11752	annexin A8
Aoc3	1.71	0.77	1.1E-04	5.6E-06	11754	amine oxidase, copper containing 3
Ap1s1	1.65	0.73	1.1E-04	5.7E-06	11769	adaptor protein complex AP-1, sigma 1
Apod	2.15	1.10	5.8E-06	1.8E-07	11815	apolipoprotein D
Arfgap3	2.29	1.19	4.4E-06	1.3E-07	66251	ADP-ribosylation factor GTPase activating protein 3
Arhgap24	1.77	0.82	6.2E-06	1.9E-07	231532	Rho GTPase activating protein 24
Arhgap33	2.79	1.48	1.4E-05	5.1E-07	233071	Rho GTPase activating protein 33
Arhgap36	71.47	6.16	2.1E-42	8.2E-46	75404	Rho GTPase activating protein 36
Arhgap44	1.96	0.97	8.2E-05	3.9E-06	216831	Rho GTPase activating protein 44
Arhgap5	0.52	-0.95	3.4E-05	1.4E-06	11855	Rho GTPase activating protein 5
Arhgdig	6.54	2.71	3.8E-25	6.0E-28	14570	Rho GDP dissociation inhibitor (GDI) gamma
Arhgef7	0.61	-0.71	1.0E-05	3.4E-07	54126	Rho guanine nucleotide exchange factor (GEF7)
Armcx4	0.56	-0.83	9.7E-08	1.7E-09	100503043	armadillo repeat containing, X-linked 4
Arsg	3.21	1.68	5.6E-08	9.4E-10	74008	arylsulfatase G
Arx	7.44	2.89	5.6E-15	2.3E-17	11878	aristaless related homeobox
Asb11	0.48	-1.05	3.3E-06	9.6E-08	68854	ankyrin repeat and SOCS box-containing 11
Asb13	0.58	-0.78	3.8E-05	1.6E-06	142688	ankyrin repeat and SOCS box-containing 13

Asb14	0.59	-0.79	6.2E-05	2.8E-06	142687	ankyrin repeat and SOCS box-containing 14
Asb15	0.21	-2.27	2.2E-13	1.2E-15	78910	ankyrin repeat and SOCS box-containing 15
Asb16	0.57	-0.81	1.8E-05	6.8E-07	217217	ankyrin repeat and SOCS box-containing 16
Asic1	0.38	-1.40	2.5E-06	6.8E-08	11419	acid sensing (proton gated) ion channel 1
Asns	3.67	1.88	1.3E-05	4.7E-07	27053	asparagine synthetase
Astn2	4.46	2.16	1.5E-06	3.9E-08	56079	astrotactin 2
Atf3	2.56	1.36	1.3E-04	6.7E-06	11910	activating transcription factor 3
Atp13a5	2.57	1.36	2.9E-07	6.0E-09	268878	ATPase type 13A5
Atp1b4	17.13	4.10	2.5E-34	1.8E-37	67821	ATPase, (Na+)/K+ transporting, beta 4 polypeptide
Atp2a1	0.55	-0.86	3.7E-06	1.1E-07	11937	ATPase, Ca++ transporting, cardiac muscle, fast twitch 1
Atp2a2	2.54	1.35	1.1E-06	2.7E-08	11938	ATPase, Ca++ transporting, cardiac muscle, slow twitch 2
Atp5a1	0.62	-0.68	4.8E-05	2.1E-06	11946	ATP synthase, H+ transporting, mitochondrial F1 complex, alpha subunit 1
Atp6v0e	1.82	0.86	2.3E-07	4.6E-09	11974	ATPase, H+ transporting, lysosomal V0 subunit E
Atp9a	1.84	0.88	1.1E-04	5.8E-06	11981	ATPase, class II, type 9A
Atrnl1	1.77	0.83	9.7E-05	4.8E-06	226255	attractin like 1
AU021092	2.47	1.30	4.2E-05	1.8E-06	239691	expressed sequence AU021092
Avpi1	2.03	1.02	3.7E-07	7.7E-09	69534	arginine vasopressin-induced 1
Axl	1.93	0.95	1.1E-06	2.7E-08	26362	AXL receptor tyrosine kinase
B3galt2	3.04	1.60	2.3E-14	1.1E-16	26878	UDP-Gal:betaGlcNAc beta 1,3-galactosyltransferase, polypeptide 2
B3gnt2	1.76	0.81	2.6E-05	1.0E-06	53625	UDP-GlcNAc:betaGal beta-1,3-N-acetylglucosaminyltransferase 2
B4galnt1	2.36	1.24	1.6E-04	8.3E-06	14421	beta-1,4-N-acetyl-galactosaminyl transferase 1
B4galt6	3.40	1.77	6.2E-08	1.0E-09	56386	UDP-Gal:betaGlcNAc beta 1,4-galactosyltransferase, polypeptide 6
B930041F14Rik	3.53	1.82	3.1E-07	6.4E-09	230991	RIKEN cDNA B930041F14 gene
Baiap2l1	4.07	2.03	2.0E-09	2.3E-11	66898	BAI1-associated protein 2-like 1
Barx2	0.36	-1.46	3.3E-06	9.6E-08	12023	BarH-like homeobox 2
Bbs5	2.14	1.10	1.2E-04	6.2E-06	72569	Bardet-Biedl syndrome 5
Bbx	0.57	-0.80	7.2E-05	3.3E-06	70508	bobby sox homolog
BC049352	6.78	2.76	4.7E-09	5.9E-11	408059	cDNA sequence BC049352
Bcam	1.57	0.65	1.1E-04	5.6E-06	57278	basal cell adhesion molecule
Bcas1	2.55	1.35	6.1E-06	1.9E-07	76960	breast carcinoma amplified sequence 1
Bdnf	4.22	2.08	2.5E-11	2.0E-13	12064	brain derived neurotrophic factor
Bex1	3.74	1.90	2.3E-09	2.7E-11	19716	brain expressed gene 1
Bin1	1.58	0.66	9.3E-05	4.5E-06	30948	bridging integrator 1
Birc6	0.63	-0.67	1.2E-04	6.1E-06	12211	baculoviral IAP repeat-containing 6
Bloc1s6	2.09	1.07	8.6E-09	1.1E-10	18457	biogenesis of organelles complex-1, subunit 6, pallidin
Brk1	1.65	0.72	9.6E-06	3.2E-07	101314	BRICK1, SCAR/WAVE actin-nucleating complex subunit
C1galt1	0.49	-1.03	1.3E-04	6.9E-06	94192	core 1 synthase, glycoprotein-N-acetylgalactosamine 3-beta-galactosyltransferase, 1
C3	2.09	1.06	1.8E-05	6.5E-07	12266	complement component 3
C530008M17Rik	2.29	1.19	3.5E-07	7.3E-09	320827	RIKEN cDNA C530008M17 gene
Cacna1g	2.03	1.02	1.8E-04	9.8E-06	12291	calcium channel, voltage-dependent, T type, alpha 1G subunit
Cacna1s	0.58	-0.79	4.7E-08	7.7E-10	12292	calcium channel, voltage-dependent, L type, alpha 1S subunit
Cacna2d3	5.95	2.57	5.5E-10	5.7E-12	12294	calcium channel, voltage-dependent, alpha2/delta subunit 3
Cadm3	2.24	1.17	1.5E-05	5.2E-07	94332	cell adhesion molecule 3
Calcr	0.06	-4.09	8.5E-11	7.5E-13	12311	calcitonin receptor
Camk2a	0.59	-0.75	5.0E-05	2.2E-06	12322	calcium/calmodulin-dependent protein kinase II alpha
Camk2d	1.64	0.71	4.6E-05	2.0E-06	108058	calcium/calmodulin-dependent protein kinase II, delta
Camk2g	0.59	-0.75	3.1E-05	1.3E-06	12325	calcium/calmodulin-dependent protein kinase II gamma
Camk2n2	2.79	1.48	7.8E-05	3.6E-06	73047	calcium/calmodulin-dependent protein kinase II inhibitor 2
Capg	1.70	0.77	9.0E-05	4.3E-06	12332	capping protein (actin filament), gelsolin-like
Capn3	0.43	-1.22	2.2E-10	2.1E-12	12335	calpain 3
Car3	0.54	-0.90	1.1E-05	3.6E-07	12350	carbonic anhydrase 3
Casq2	2.64	1.40	1.7E-10	1.6E-12	12373	calsequestrin 2
Casz1	0.53	-0.92	8.4E-06	2.7E-07	69743	castor zinc finger 1
Cbfb	0.56	-0.84	1.3E-04	6.6E-06	12400	core binding factor beta
Cbl1	0.51	-0.97	8.8E-08	1.5E-09	70266	cysteine conjugate-beta lyase 1
Ccdc110	19.79	4.31	4.7E-09	5.9E-11	212392	coiled-coil domain containing 110
Ccdc122	3.24	1.70	7.3E-06	2.3E-07	108811	coiled-coil domain containing 122
Ccdc127	0.62	-0.69	6.4E-05	2.9E-06	67433	coiled-coil domain containing 127
Ccdc136	3.61	1.85	3.0E-08	4.4E-10	232664	coiled-coil domain containing 136
Ccdc186	0.48	-1.07	2.4E-05	9.3E-07	213993	coiled-coil domain containing 186
Ccl6	2.38	1.25	1.7E-04	9.3E-06	20305	chemokine (C-C motif) ligand 6
Ccndbp1	2.30	1.20	8.0E-09	1.0E-10	17151	cyclin D-type binding-protein 1
Cd151	1.62	0.70	1.5E-04	8.1E-06	12476	CD151 antigen
Cd24a	3.33	1.74	1.2E-16	4.0E-19	12484	CD24a antigen
Cd276	2.19	1.13	2.0E-08	2.7E-10	102657	CD276 antigen
Cd9	1.80	0.85	3.2E-06	9.0E-08	12527	CD9 antigen
Cdc42ep3	0.66	-0.59	1.2E-04	6.0E-06	260409	CDC42 effector protein (Rho GTPase binding) 3
Cdkn1a	5.33	2.41	1.8E-08	2.5E-10	12575	cyclin-dependent kinase inhibitor 1A (P21)
Cdkn1b	0.59	-0.75	1.9E-06	4.9E-08	12576	cyclin-dependent kinase inhibitor 1B
Cdo1	7.50	2.91	4.2E-13	2.4E-15	12583	cysteine dioxygenase 1, cytosolic
Cebpa	3.36	1.75	1.2E-08	1.6E-10	12606	CCAAT/enhancer binding protein (C/EBP), alpha
Cecr2	0.55	-0.86	2.9E-05	1.1E-06	330409	cat eye syndrome chromosome region, candidate 2
Cercam	2.04	1.03	1.4E-04	7.5E-06	99151	cerebral endothelial cell adhesion molecule
Ces1d	5.34	2.42	3.6E-08	5.6E-10	104158	carboxylesterase 1D
Cfd	6.58	2.72	9.5E-18	2.7E-20	11537	complement factor D (adipsin)
Cgref1	5.60	2.48	6.0E-07	1.3E-08	68567	cell growth regulator with EF hand domain 1
Chd2	0.58	-0.80	5.3E-05	2.3E-06	244059	chromodomain helicase DNA binding protein 2
Chd9	0.61	-0.72	1.5E-04	8.2E-06	109151	chromodomain helicase DNA binding protein 9
Chdh	5.45	2.45	2.4E-10	2.3E-12	218865	choline dehydrogenase
Chm	0.50	-1.00	1.2E-06	3.0E-08	12662	choroidermia
Chrna1	3.95	1.98	1.9E-07	3.6E-09	11435	cholinergic receptor, nicotinic, alpha polypeptide 1 (muscle)
Chrnd	8.33	3.06	7.0E-11	6.0E-13	11447	cholinergic receptor, nicotinic, delta polypeptide
Chrne	2.07	1.05	1.2E-12	7.5E-15	11448	cholinergic receptor, nicotinic, epsilon polypeptide
Chrng	51.81	5.70	6.5E-12	4.5E-14	11449	cholinergic receptor, nicotinic, gamma polypeptide
Cidea	5.62	2.49	1.2E-20	2.5E-23	12683	cell death-inducing DNA fragmentation factor, alpha subunit-like effector A
Cidec	8.09	3.02	1.8E-11	1.3E-13	14311	cell death-inducing DFFA-like effector c
Cilp	7.38	2.88	3.4E-44	1.2E-47	214425	cartilage intermediate layer protein, nucleotide pyrophosphohydrolase
Cilp2	2.98	1.57	2.4E-07	4.8E-09	68709	cartilage intermediate layer protein 2
Ckap4	1.80	0.85	1.6E-04	8.6E-06	216197	cytoskeleton-associated protein 4
Ckb	1.96	0.97	9.0E-05	4.3E-06	12709	creatine kinase, brain
Clcn1	0.44	-1.19	2.1E-09	2.4E-11	12723	chloride channel 1
Cldn12	0.56	-0.83	1.6E-06	4.0E-08	64945	claudin 12
Cldn19	2.93	1.55	2.2E-09	2.6E-11	242653	claudin 19
Cluh	0.62	-0.70	1.5E-05	5.2E-07	74148	clustered mitochondria (cluA/CLU1) homolog
Cmss1	0.40	-1.33	8.8E-09	1.2E-10	66497	cms small ribosomal subunit 1
Cmya5	0.42	-1.25	2.8E-12	1.8E-14	76469	cardiomyopathy associated 5
Cnksr1	1.99	1.00	1.9E-07	3.7E-09	194231	connector enhancer of kinase suppressor of Ras 1
Cnnm4	0.52	-0.95	1.9E-06	5.0E-08	94220	cyclin M4
Cnot1	0.62	-0.69	1.9E-05	7.2E-07	234594	CCR4-NOT transcription complex, subunit 1
Cnp	1.99	1.00	1.2E-05	4.1E-07	12799	2',3'-cyclic nucleotide 3' phosphodiesterase
Cntnap4	87.33	6.45	7.2E-30	8.7E-33	170571	contactin associated protein-like 4
Col19a1	3.73	1.90	5.9E-07	1.3E-08	12823	collagen, type XIX, alpha 1
Col22a1	1.93	0.95	2.5E-06	6.9E-08	69700	collagen, type XXII, alpha 1
Col23a1	3.83	1.94	1.1E-09	1.2E-11	237759	collagen, type XXIII, alpha 1
Col6a6	0.31	-1.70	7.2E-05	3.3E-06	245026	collagen, type VI, alpha 6
Col7a1	2.64	1.40	1.8E-06	4.7E-08	12836	collagen, type VII, alpha 1
Col8a2	3.66	1.87	3.8E-06	1.1E-07	329941	collagen, type VIII, alpha 2
Colq	3.39	1.76	1.3E-08	1.7E-10	382864	collagen-like tail subunit (single strand of homotrimer) of asymmetric acetylcholinesterase
Coprs	2.14	1.10	9.4E-08	1.7E-09	66423	coordinator of PRMT5, differentiation stimulator
Cox19	1.54	0.62	9.6E-05	4.7E-06	68033	COX19 cytochrome c oxidase assembly homolog
Cox8a	1.80	0.85	3.8E-05	1.6E-06	12868	cytochrome c oxidase, subunit VIIIa
Cpeb2	2.60	1.38	4.2E-15	1.7E-17	231207	cytoplasmic polyadenylation element binding protein 2
Cpne2	8.23	3.04	7.3E-24	1.3E-26	234577	copine II
Cpox	0.58	-0.78	5.5E-06	1.7E-07	12892	coproporphyrinogen oxidase
Creb1	0.63	-0.66	1.4E-04	7.2E-06	12912	cAMP responsive element binding protein 1
Crip1	1.84	0.88	2.4E-06	6.5E-08	12925	cysteine-rich protein 1 (intestinal)
Crlf2	3.02	1.59	1.0E-07	1.8E-09	57914	cytokine receptor-like factor 2
Cryab	1.97	0.98	6.1E-06	1.9E-07	12955	crystallin, alpha B
Crym	2.18	1.13	2.8E-07	5.6E-09	12971	crystallin, mu
Cs	0.66	-0.60	9.5E-05	4.6E-06	12974	citrate synthase
Csdc2	3.52	1.81	2.7E-09	3.3E-11	105859	cold shock domain containing C2, RNA binding
Csl	0.54	-0.90	1.2E-07	2.2E-09	71832	citrate synthase like
Csrp3	4.02	2.01	5.6E-05	2.5E-06	13009	cysteine and glycine-rich protein 3

Cst6	2.78	1.48	9.9E-13	5.9E-15	73720	cystatin E/M
Ctdspl	1.95	0.96	6.1E-06	1.9E-07	69274	CTD (carboxy-terminal domain, RNA polymerase II, polypeptide A) small phosphatase-like
Ctgf	2.78	1.47	9.1E-07	2.2E-08	14219	connective tissue growth factor
Ctnna3	2.46	1.30	1.0E-07	1.9E-09	216033	catenin (cadherin associated protein), alpha 3
Ctss	2.81	1.49	9.9E-05	4.9E-06	13040	cathepsin S
Ctxn3	0.33	-1.62	6.3E-12	4.3E-14	629147	cortexin 3
Cul7	2.08	1.05	1.4E-05	4.8E-07	66515	cullin 7
Cux1	0.63	-0.68	1.9E-05	7.1E-07	13047	cut-like homeobox 1
Cxcl14	0.33	-1.59	1.5E-05	5.6E-07	57266	chemokine (C-X-C motif) ligand 14
Cxcr4	0.43	-1.23	1.5E-04	8.0E-06	12767	chemokine (C-X-C motif) receptor 4
Cyb5r3	2.42	1.27	3.6E-06	1.0E-07	109754	cytochrome b5 reductase 3
Cyfip2	3.88	1.96	7.3E-14	3.8E-16	76884	cytoplasmic FMR1 interacting protein 2
Cyp2e1	9.35	3.22	7.0E-08	1.2E-09	13106	cytochrome P450, family 2, subfamily e, polypeptide 1
D830015G02Rik	15.16	3.92	7.4E-08	1.3E-09	791403	RIKEN cDNA D830015G02 gene
Dact3	2.27	1.18	1.5E-04	7.9E-06	629378	dapper homolog 3, antagonist of beta-catenin
Dapk1	2.10	1.07	2.2E-05	8.3E-07	69635	death associated protein kinase 1
Dapk2	1.74	0.80	7.4E-05	3.4E-06	13143	death-associated protein kinase 2
Dbn1	1.93	0.95	3.9E-05	1.6E-06	56320	drebrin 1
Dcaf11	0.60	-0.73	1.2E-05	4.3E-07	28199	DDB1 and CUL4 associated factor 11
Dcaf12l1	6.41	2.68	8.0E-18	2.2E-20	245404	DDB1 and CUL4 associated factor 12-like 1
Dclk1	4.96	2.31	1.2E-04	5.9E-06	13175	doublecortin-like kinase 1
Dcun1d2	0.62	-0.69	1.7E-04	8.9E-06	102323	DCN1, defective in cullin neddylation 1, domain containing 2
Ddah2	1.69	0.75	1.0E-04	5.2E-06	51793	dimethylarginine dimethylaminohydrolase 2
Ddhd1	1.86	0.89	6.2E-05	2.8E-06	114874	DDHD domain containing 1
Ddit4l	0.41	-1.29	6.9E-08	1.2E-09	73284	DNA-damage-inducible transcript 4-like
Dennd4b	0.63	-0.67	1.0E-04	5.1E-06	229541	DENN/MADD domain containing 4B
Dennd4c	0.35	-1.52	6.6E-07	1.5E-08	329877	DENN/MADD domain containing 4C
Des	1.99	1.00	7.2E-09	9.2E-11	13346	desmin
Dfna5	4.64	2.21	2.7E-07	5.4E-09	54722	deafness, autosomal dominant 5
Dhcr24	2.00	1.00	1.3E-04	6.6E-06	74754	24-dehydrocholesterol reductase
Dhrs3	1.80	0.85	5.4E-05	2.4E-06	20148	dehydrogenase/reductase (SDR family) member 3
Dhrs7	2.22	1.15	2.3E-08	3.3E-10	66375	dehydrogenase/reductase (SDR family) member 7
Dixdc1	0.53	-0.92	1.1E-04	5.7E-06	330938	DIX domain containing 1
Dlg5	1.74	0.80	1.5E-04	7.7E-06	71228	discs, large homolog 5
Dlk1	8.79	3.14	6.4E-41	2.9E-44	13386	delta-like 1 homolog
Dnajb5	0.55	-0.85	6.7E-06	2.1E-07	56323	DnaJ (Hsp40) homolog, subfamily B, member 5
Dnase1l1	0.60	-0.73	1.2E-04	6.1E-06	69537	deoxyribonuclease 1-like 1
Dnm1	1.96	0.97	5.7E-06	1.7E-07	13429	dynamin 1
Dnph1	0.43	-1.23	6.6E-06	2.1E-07	381101	2'-deoxynucleoside 5'-phosphate N-hydrolase 1
Dok1	2.11	1.08	3.2E-05	1.3E-06	13448	docking protein 1
Dpf3	0.54	-0.90	2.6E-05	1.0E-06	70127	D4, zinc and double PHD fingers, family 3
Dpp8	0.61	-0.70	7.1E-06	2.2E-07	74388	dipeptidylpeptidase 8
Dram1	2.77	1.47	7.7E-06	2.5E-07	71712	DNA-damage regulated autophagy modulator 1
Drp2	2.14	1.10	6.3E-05	2.8E-06	13497	dystrophin related protein 2
Dst	0.56	-0.85	5.4E-05	2.3E-06	13518	dystonin
Dtx4	0.58	-0.78	1.1E-05	3.9E-07	207521	deltex 4 homolog
Dusp13	0.59	-0.76	1.1E-05	3.6E-07	27389	dual specificity phosphatase 13
Dusp15	4.17	2.06	6.2E-08	1.0E-09	252864	dual specificity phosphatase-like 15
E030003E18Rik	4.40	2.14	4.3E-31	4.4E-34	320092	RIKEN cDNA E030003E18 gene
Ecd	0.65	-0.61	1.2E-04	5.9E-06	70601	ecdysoneless homolog
Ecm1	2.19	1.13	5.3E-06	1.6E-07	13601	extracellular matrix protein 1
Eda2r	5.38	2.43	1.0E-14	4.6E-17	245527	ectodysplasin A2 receptor
Egf	1.65	0.72	9.0E-05	4.3E-06	13645	epidermal growth factor
Egflam	0.48	-1.05	2.7E-08	3.9E-10	268780	EGF-like, fibronectin type III and laminin G domains
Egln3	5.79	2.53	4.0E-11	3.3E-13	112407	EGL nine homolog 3
Egr1	5.42	2.44	1.0E-04	5.2E-06	13653	early growth response 1
Egr2	4.19	2.07	3.1E-06	8.8E-08	13654	early growth response 2
Ehd3	4.43	2.15	3.8E-26	5.7E-29	57440	EH-domain containing 3
Ehd4	2.41	1.27	2.3E-07	4.5E-09	98878	EH-domain containing 4
Eid1	1.99	1.00	1.3E-08	1.8E-10	58521	EP300 interacting inhibitor of differentiation 1
Eln	1.78	0.83	2.4E-06	6.5E-08	13717	elastin
Elovl1	2.02	1.01	1.5E-05	5.5E-07	54325	elongation of very long chain fatty acids (FEN1/Elo2, SUR4/Elo3, yeast)-like 1
Emb	5.84	2.55	1.3E-14	6.2E-17	13723	embigin
Emp2	1.68	0.75	8.5E-05	4.0E-06	13731	epithelial membrane protein 2
Enah	1.88	0.91	8.7E-07	2.1E-08	13800	enabled homolog
Engase	1.96	0.97	8.0E-05	3.7E-06	217364	endo-beta-N-acetylglucosaminidase
Enho	3.12	1.64	1.9E-11	1.4E-13	69638	energy homeostasis associated
Enkur	4.67	2.22	3.5E-08	5.4E-10	71233	enkurin, TRPC channel interacting protein
Epb4.1	0.61	-0.71	1.2E-04	6.4E-06	269587	erythrocyte protein band 4.1
Epb4.1l4a	2.28	1.19	5.2E-05	2.2E-06	13824	erythrocyte protein band 4.1-like 4a
Epg5	0.56	-0.85	5.8E-05	2.6E-06	100502841	ectopic P-granules autophagy protein 5 homolog
Ephb3	3.45	1.79	1.1E-04	5.5E-06	13845	Eph receptor B3
Ephb6	3.45	1.79	6.5E-12	4.5E-14	13848	Eph receptor B6
Epm2aip1	0.52	-0.94	4.9E-06	1.5E-07	77781	EPM2A (laforin) interacting protein 1
Erbb2	2.10	1.07	1.2E-05	4.2E-07	13866	v-erb-b2 erythroblastic leukemia viral oncogene homolog 2, neuro/glioblastoma derived oncogene homolog
Esr1	0.28	-1.85	2.5E-07	4.9E-09	13982	estrogen receptor 1 (alpha)
Etv5	2.36	1.24	4.8E-08	7.9E-10	104156	ets variant gene 5
Fa2h	4.14	2.05	1.3E-09	1.4E-11	338521	fatty acid 2-hydroxylase
Fam110a	2.98	1.58	3.0E-11	2.4E-13	73847	family with sequence similarity 110, member A
Fam124a	3.21	1.68	5.3E-09	6.8E-11	629059	family with sequence similarity 124, member A
Fam171a2	3.44	1.78	1.1E-04	5.7E-06	217219	family with sequence similarity 171, member A2
Fam171b	14.32	3.84	8.1E-33	7.2E-36	241520	family with sequence similarity 171, member B
Fam178b	3.24	1.70	3.3E-05	1.3E-06	381337	family with sequence similarity 178, member B
Fam210a	0.54	-0.89	3.2E-05	1.3E-06	108654	family with sequence similarity 210, member A
Fam214b	1.93	0.95	2.0E-08	2.8E-10	230088	family with sequence similarity 214, member B
Fam46a	2.12	1.08	1.4E-07	2.7E-09	212943	family with sequence similarity 46, member A
Fam65b	3.45	1.79	9.0E-07	2.1E-08	193385	family with sequence similarity 65, member B
Fam78a	0.40	-1.33	4.8E-09	6.0E-11	241303	family with sequence similarity 78, member A
Fam81a	9.60	3.26	7.3E-19	1.7E-21	76886	family with sequence similarity 81, member A
Fam83h	7.15	2.84	2.3E-09	2.7E-11	105732	family with sequence similarity 83, member H
Fan1	0.49	-1.04	8.2E-06	2.7E-07	330554	FANCD2/FANCI-associated nuclease 1
Fat1	2.17	1.12	9.2E-06	3.1E-07	14107	FAT tumor suppressor homolog 1
Fbxl4	1.87	0.91	9.8E-09	1.3E-10	269514	F-box and leucine-rich repeat protein 4
Fbxo17	2.65	1.41	5.7E-10	5.9E-12	50760	F-box protein 17
Fbxo3	0.61	-0.71	1.3E-05	4.4E-07	57443	F-box protein 3
Fbxo36	4.21	2.07	1.4E-06	3.6E-08	66153	F-box protein 36
Fbxo40	0.53	-0.93	6.0E-05	2.7E-06	207215	F-box protein 40
Fbxo44	1.76	0.82	1.1E-04	5.3E-06	230903	F-box protein 44
Fech	0.63	-0.66	8.4E-05	4.0E-06	14151	ferrochelatase
Fert2	1.78	0.83	6.0E-05	2.7E-06	14158	fer (fms/fps related) protein kinase, testis specific 2
Fez1	3.42	1.77	3.9E-07	8.1E-09	235180	fasciculation and elongation protein zeta 1 (zygin I)
Fgf5	5.70	2.51	1.6E-07	3.0E-09	14176	fibroblast growth factor 5
Fgf6	2.29	1.20	5.6E-06	1.7E-07	14177	fibroblast growth factor 6
Fgf7	3.10	1.63	3.1E-05	1.2E-06	14178	fibroblast growth factor 7
Fgf9	3.22	1.69	3.9E-06	1.1E-07	14180	fibroblast growth factor 9
Fgfr3	2.63	1.40	1.1E-04	5.3E-06	14184	fibroblast growth factor receptor 3
Fgfrl1	3.06	1.61	1.5E-20	3.3E-23	116701	fibroblast growth factor receptor-like 1
Figf	2.26	1.18	5.6E-05	2.4E-06	14205	c-fos induced growth factor
Fkbp5	2.41	1.27	1.6E-05	5.7E-07	14229	FK506 binding protein 5
Fndc5	0.43	-1.20	7.8E-06	2.5E-07	384061	fibronectin type III domain containing 5
Foxd3	5.29	2.40	2.2E-08	3.2E-10	15221	forkhead box D3
Foxo4	0.61	-0.71	2.9E-05	1.2E-06	54601	forkhead box O4
Fras1	3.00	1.58	2.9E-07	5.9E-09	231470	Fraser syndrome 1 homolog
Frem2	3.12	1.64	6.7E-09	8.5E-11	242022	Fras1 related extracellular matrix protein 2
Fry	0.62	-0.68	1.1E-05	3.9E-07	320365	furry homolog
Fst	3.61	1.85	5.3E-10	5.5E-12	14313	follistatin
Fuca2	1.69	0.76	1.4E-05	5.1E-07	66848	fucosidase, alpha-L- 2, plasma
Fut8	2.09	1.07	4.6E-08	7.4E-10	53618	fucosyltransferase 8
Fv1	0.34	-1.58	6.6E-06	2.1E-07	14349	Friend virus susceptibility 1
Fyco1	0.46	-1.13	1.2E-07	2.2E-09	17281	FYVE and coiled-coil domain containing 1
Gabrb2	10.63	3.41	2.9E-16	1.0E-18	14401	gamma-aminobutyric acid (GABA) A receptor, subunit beta 2
Gabrb3	26.90	4.75	2.5E-17	7.3E-20	14402	gamma-aminobutyric acid (GABA) A receptor, subunit beta 3
Galk2	2.10	1.07	7.5E-05	3.5E-06	69976	galactokinase 2

Galm	2.80	1.49	9.6E-06	3.2E-07	319625	galactose mutarotase
Galns	3.38	1.76	2.4E-05	9.4E-07	50917	galactosamine (N-acetyl)-6-sulfate sulfatase
Ganc	0.58	-0.79	5.1E-05	2.2E-06	76051	glucosidase, alpha; neutral C
Gas2l1	2.24	1.16	2.0E-05	7.3E-07	78926	growth arrest-specific 2 like 1
Gatm	5.52	2.46	3.3E-07	6.8E-09	67092	glycine amidinotransferase (L-arginine:glycine amidinotransferase)
Gck	3.25	1.70	8.3E-05	3.9E-06	103988	glucokinase
Gdf11	2.55	1.35	5.1E-08	8.5E-10	14561	growth differentiation factor 11
Gdf5	7.72	2.95	2.4E-14	1.1E-16	14563	growth differentiation factor 5
Gdnf	2.52	1.34	2.2E-05	8.3E-07	14573	glial cell line derived neurotrophic factor
Gfm1	0.66	-0.60	1.3E-04	6.6E-06	28030	G elongation factor, mitochondrial 1
Gga2	2.16	1.11	8.3E-06	2.7E-07	74105	golgi associated, gamma adaptin ear containing, ARF binding protein 2
Ghr	0.61	-0.72	3.2E-06	9.3E-08	14600	growth hormone receptor
Gjc3	1.99	0.99	1.8E-04	9.8E-06	118446	gap junction protein, gamma 3
Gldn	5.09	2.35	9.5E-18	2.7E-20	235379	gliomedin
Gls	0.54	-0.89	8.9E-06	2.9E-07	14660	glutaminase
Gltp	1.99	0.99	1.4E-05	4.9E-07	56356	glycolipid transfer protein
Gm11149	4.59	2.20	8.2E-05	3.9E-06	100036537	predicted gene 11149
Gm13889	6.59	2.72	8.0E-21	1.6E-23	620695	predicted gene 13889
Gm16793	13.58	3.76	3.1E-17	9.6E-20	100504714	predicted gene, 16793
Gm4814	0.60	-0.74	2.3E-06	6.2E-08	100502942	predicted gene 4814
Gm4980	0.59	-0.76	1.3E-06	3.3E-08	100503386	predicted gene 4980
Gm5105	0.40	-1.33	6.9E-08	1.2E-09	329763	predicted gene 5105
Gm973	2.67	1.41	1.0E-05	3.5E-07	381260	predicted gene 973
Gnai1	2.28	1.19	4.1E-05	1.7E-06	14677	guanine nucleotide binding protein (G protein), alpha inhibiting 1
Gnl1	1.62	0.69	1.6E-04	8.7E-06	14670	guanine nucleotide binding protein-like 1
Got1	0.57	-0.82	5.3E-06	1.6E-07	14718	glutamate oxaloacetate transaminase 1, soluble
Gp1bb	3.26	1.71	1.2E-05	4.3E-07	14724	glycoprotein Ib, beta polypeptide
Gpc4	2.23	1.15	3.2E-06	9.1E-08	14735	glypican 4
Gpcpd1	0.46	-1.12	9.0E-07	2.1E-08	74182	glycerophosphocholine phosphodiesterase GDE1 homolog
Gpd2	0.46	-1.12	1.9E-06	5.0E-08	14571	glycerol phosphate dehydrogenase 2, mitochondrial
Gpt2	0.43	-1.20	1.9E-06	5.1E-08	108682	glutamic pyruvate transaminase (alanine aminotransferase) 2
Gpx1	2.45	1.29	3.3E-15	1.3E-17	14775	glutathione peroxidase 1
Gpx8	2.27	1.18	4.2E-09	5.2E-11	69590	glutathione peroxidase 8 (putative)
Grb10	1.88	0.91	8.2E-05	3.9E-06	14783	growth factor receptor bound protein 10
Grin3b	2.62	1.39	2.1E-05	7.8E-07	170483	glutamate receptor, ionotropic, NMDA3B
Grrp1	2.37	1.24	2.8E-05	1.1E-06	72690	glycine/arginine rich protein 1
Gsn	1.80	0.84	6.3E-06	2.0E-07	227753	gelsolin
Guk1	2.36	1.24	1.1E-11	7.9E-14	14923	guanylate kinase 1
H19	2.29	1.20	7.1E-05	3.3E-06	14955	H19 fetal liver mRNA
H2afj	1.75	0.81	1.1E-04	5.4E-06	232440	H2A histone family, member J
H6pd	0.62	-0.70	4.1E-06	1.2E-07	100198	hexose-6-phosphate dehydrogenase (glucose 1-dehydrogenase)
Hadha	0.64	-0.64	1.0E-05	3.5E-07	97212	hydroxyacyl-Coenzyme A dehydrogenase/3-ketoacyl-Coenzyme A thiolase/enoyl-Coenzyme A hydratase (trifunctional protein), alpha subunit
Haghl	2.12	1.08	1.9E-09	2.2E-11	68977	hydroxyacylglutathione hydrolase-like
Hccs	0.59	-0.77	2.1E-05	7.7E-07	15159	holocytochrome c synthetase
Herc1	0.54	-0.89	1.3E-06	3.3E-08	235439	hect (homologous to the E6-AP (UBE3A) carboxyl terminus) domain and RCC1 (CHC1)-like domain (RLD) 1
Hexa	1.82	0.87	1.0E-06	2.5E-08	15211	hexosaminidase A
Hif3a	3.48	1.80	3.1E-08	4.6E-10	53417	hypoxia inducible factor 3, alpha subunit
Hist1h1c	1.71	0.78	2.0E-05	7.5E-07	50708	histone cluster 1, H1c
Hist1h1d	4.18	2.06	3.0E-09	3.7E-11	14957	histone cluster 1, H1d
Hist1h1e	2.51	1.33	1.0E-05	3.5E-07	50709	histone cluster 1, H1e
Hist1h4i	1.97	0.98	1.8E-06	4.7E-08	319158	histone cluster 1, H4i
Hk1	2.63	1.40	7.5E-10	7.9E-12	15275	hexokinase 1
Hmga1	1.98	0.99	7.4E-05	3.4E-06	15361	high mobility group AT-hook 1
Hmga1-rs1	2.02	1.01	7.0E-05	3.2E-06	111241	high mobility group AT-hook I, related sequence 1
Hmgcs2	3.65	1.87	6.6E-07	1.5E-08	15360	3-hydroxy-3-methylglutaryl-Coenzyme A synthase 2
Hoga1	2.22	1.15	4.6E-05	2.0E-06	67432	4-hydroxy-2-oxoglutarate aldolase 1
Homer1	0.53	-0.91	5.7E-06	1.7E-07	26556	homer homolog 1
Hook3	0.47	-1.10	2.1E-10	1.9E-12	320191	hook homolog 3
Hoxd8	2.30	1.20	9.9E-05	4.8E-06	15437	homeobox D8
Hoxd9	3.28	1.71	1.7E-05	6.2E-07	15438	homeobox D9
Hp	9.12	3.19	3.8E-16	1.4E-18	15439	haptoglobin
Hrasls	0.32	-1.63	1.7E-06	4.4E-08	27281	HRAS-like suppressor
Hrc	0.61	-0.72	6.7E-06	2.1E-07	15464	histidine rich calcium binding protein
Hs6st2	2.03	1.02	4.8E-06	1.4E-07	50786	heparan sulfate 6-O-sulfotransferase 2
Hsd11b1	0.51	-0.99	5.7E-05	2.5E-06	15483	hydroxysteroid 11-beta dehydrogenase 1
Hsd17b14	4.43	2.15	2.7E-07	5.3E-09	66065	hydroxysteroid (17-beta) dehydrogenase 14
Hsd17b4	0.60	-0.74	4.5E-06	1.3E-07	15488	hydroxysteroid (17-beta) dehydrogenase 4
Hsdl2	0.63	-0.66	3.1E-05	1.2E-06	72479	hydroxysteroid dehydrogenase like 2
Hspa1a	4.12	2.04	1.7E-13	9.2E-16	193740	heat shock protein 1A
Hspa1b	3.87	1.95	1.7E-06	4.6E-08	15511	heat shock protein 1B
Hspa1l	1.94	0.96	1.2E-08	1.7E-10	15482	heat shock protein 1-like
Hspb1	3.84	1.94	4.4E-15	1.8E-17	15507	heat shock protein 1
Hspb11	2.08	1.06	1.8E-05	6.4E-07	72938	heat shock protein family B (small), member 11
Hspb8	1.95	0.97	4.0E-06	1.2E-07	80888	heat shock protein 8
Htatip2	2.39	1.26	2.1E-06	5.8E-08	53415	HIV-1 tat interactive protein 2, homolog
Id1	1.81	0.85	1.3E-05	4.5E-07	15901	inhibitor of DNA binding 1
Id2	2.22	1.15	3.0E-05	1.2E-06	15902	inhibitor of DNA binding 2
Idh3a	0.56	-0.84	6.6E-06	2.1E-07	67834	isocitrate dehydrogenase 3 (NAD+) alpha
Idh3g	0.58	-0.78	3.7E-05	1.5E-06	15929	isocitrate dehydrogenase 3 (NAD+), gamma
Ifitm10	3.10	1.63	5.9E-05	2.6E-06	320802	interferon induced transmembrane protein 10
Ifnar2	2.17	1.12	4.9E-09	6.2E-11	15976	interferon (alpha and beta) receptor 2
Igdcc4	1.74	0.80	6.7E-06	2.1E-07	56741	immunoglobulin superfamily, DCC subclass, member 4
Igf2bp2	7.34	2.88	5.0E-25	8.0E-28	319765	insulin-like growth factor 2 mRNA binding protein 2
Igf2os	6.73	2.75	2.6E-06	7.3E-08	111975	insulin-like growth factor 2, opposite strand
Igfbp2	14.85	3.89	2.8E-07	5.7E-09	16008	insulin-like growth factor binding protein 2
Igsf1	18.20	4.19	1.4E-27	1.9E-30	209268	immunoglobulin superfamily, member 1
Il17re	17.25	4.11	2.7E-34	2.0E-37	57890	interleukin 17 receptor E
Il18bp	2.72	1.44	2.6E-06	7.2E-08	16068	interleukin 18 binding protein
Il20rb	2.84	1.51	1.8E-05	6.8E-07	213208	interleukin 20 receptor beta
Inf2	1.66	0.73	9.7E-05	4.8E-06	70435	inverted formin, FH2 and WH2 domain containing
Inha	0.50	-1.00	4.3E-05	1.8E-06	16322	inhibin alpha
Ino80	0.59	-0.75	1.7E-04	8.9E-06	68142	INO80 homolog
Ino80dos	0.59	-0.75	9.1E-06	3.0E-07	100380944	INO80 complex subunit D, opposite strand
Irf2bp2	1.84	0.88	6.7E-05	3.1E-06	270110	interferon regulatory factor 2 binding protein 2
Irx3	5.71	2.51	2.1E-10	1.9E-12	16373	Iroquois related homeobox 3
Islr2	2.65	1.41	1.8E-04	9.7E-06	320563	immunoglobulin superfamily containing leucine-rich repeat 2
Itga5	1.91	0.94	3.0E-06	8.4E-08	16402	integrin alpha 5 (fibronectin receptor alpha)
Itga9	5.47	2.45	6.1E-32	6.0E-35	104099	integrin alpha 9
Itgb1bp2	1.71	0.77	3.5E-08	5.5E-10	26549	integrin beta 1 binding protein 2
Itgb5	0.63	-0.67	3.0E-05	1.2E-06	16419	integrin beta 5
Ivd	0.54	-0.90	4.3E-05	1.8E-06	56357	isovaleryl coenzyme A dehydrogenase
Junb	2.78	1.47	3.6E-05	1.5E-06	16477	Jun-B oncogene
Kank1	1.90	0.93	1.2E-05	4.0E-07	107351	KN motif and ankyrin repeat domains 1
Kat6a	0.55	-0.86	2.4E-05	9.4E-07	244349	lysine (K) acetyltransferase 6A
Katnal2	76.87	6.26	2.1E-47	4.8E-51	71206	katanin p60 subunit A-like 2
Kbtbd12	0.42	-1.24	5.0E-05	2.1E-06	74589	kelch repeat and BTB (POZ) domain containing 12
Kbtbd13	0.41	-1.28	3.6E-12	2.3E-14	74492	kelch repeat and BTB (POZ) domain containing 13
Kcna1	2.94	1.56	4.6E-12	3.0E-14	16485	potassium voltage-gated channel, shaker-related subfamily, member 1
Kcna6	3.25	1.70	2.0E-06	5.2E-08	16494	potassium voltage-gated channel, shaker-related, subfamily, member 6
Kcne1l	2.33	1.22	6.0E-14	3.0E-16	66240	potassium voltage-gated channel, Isk-related family, member 1-like, pseudogene
Kcnf1	2.72	1.45	2.9E-05	1.1E-06	382571	potassium voltage-gated channel, subfamily F, member 1
Kcnh2	2.77	1.47	6.7E-14	3.4E-16	16511	potassium voltage-gated channel, subfamily H (eag-related), member 2
Kcnn3	2.58	1.37	9.1E-11	8.0E-13	140493	potassium intermediate/small conductance calcium-activated channel, subfamily N, member 3
Kdm5a	0.57	-0.81	6.6E-07	1.5E-08	214899	lysine (K)-specific demethylase 5A
Kdm7a	0.59	-0.75	1.1E-05	3.7E-07	338523	lysine (K)-specific demethylase 7a
Khdrbs3	0.42	-1.24	8.4E-05	4.0E-06	13992	KH domain containing, RNA binding, signal transduction associated 3
Kif19a	3.62	1.86	9.0E-07	2.1E-08	286942	kinesin family member 19A
Kif1a	2.99	1.58	1.3E-04	7.0E-06	16560	kinesin family member 1A
Kif1b	0.57	-0.82	2.5E-06	6.8E-08	16561	kinesin family member 1B
Klf12	0.44	-1.17	1.9E-05	7.2E-07	16597	Kruppel-like factor 12
Klf2	1.84	0.88	1.7E-04	8.9E-06	16598	Kruppel-like factor 2 (lung)
Klf5	3.66	1.87	1.3E-06	3.2E-08	12224	Kruppel-like factor 5
Klhdc7a	9.87	3.30	3.1E-10	2.9E-12	242721	kelch domain containing 7A

Klhdc8b	2.37	1.24	8.5E-09	1.1E-10	78267	kelch domain containing 8B
Klhl13	2.54	1.34	1.0E-06	2.4E-08	67455	kelch-like 13
Klhl24	0.60	-0.73	7.9E-05	3.7E-06	75785	kelch-like 24
Klhl30	1.64	0.71	4.4E-05	1.9E-06	70788	kelch-like 30
Klhl34	0.44	-1.20	2.0E-06	5.3E-08	245683	kelch-like 34
Krt18	5.96	2.58	3.7E-12	2.5E-14	16668	keratin 18
Lamc2	3.11	1.64	1.1E-06	2.6E-08	16782	laminin, gamma 2
Large	1.81	0.86	4.1E-06	1.2E-07	16795	like-glycosyltransferase
Larp4	0.57	-0.80	3.0E-05	1.2E-06	207214	La ribonucleoprotein domain family, member 4
Lbx2	3.61	1.85	3.0E-07	6.0E-09	16815	ladybird homeobox homolog 2
Lcor	0.29	-1.77	1.2E-05	4.3E-07	212391	ligand dependent nuclear receptor corepressor
Ldlrad3	3.11	1.64	5.8E-09	7.3E-11	241576	low density lipoprotein receptor class A domain containing 3
Lep	8.31	3.06	2.3E-09	2.8E-11	16846	leptin
Leprel1	3.70	1.89	3.6E-13	2.1E-15	210530	leprecan-like 1
Lgals1	2.68	1.42	1.4E-08	1.9E-10	16852	lectin, galactose binding, soluble 1
Lgals12	9.93	3.31	1.5E-12	9.4E-15	56072	lectin, galactose binding, soluble 12
Lgals3	17.05	4.09	1.1E-11	8.0E-14	16854	lectin, galactose binding, soluble 3
Lifr	0.46	-1.12	2.7E-07	5.3E-09	16880	leukemia inhibitory factor receptor
Limd2	1.81	0.86	3.1E-05	1.2E-06	67803	LIM domain containing 2
Lin9	0.38	-1.38	7.5E-05	3.5E-06	72568	lin-9 homolog
Lingo3	2.29	1.19	1.0E-07	1.8E-09	237403	leucine rich repeat and Ig domain containing 3
Lman1	0.57	-0.82	2.9E-06	8.3E-08	70361	lectin, mannose-binding, 1
Lmcd1	2.32	1.21	3.6E-08	5.6E-10	30937	LIM and cysteine-rich domains 1
Lmo2	1.90	0.93	4.8E-05	2.1E-06	16909	LIM domain only 2
Lnpep	0.38	-1.41	3.2E-05	1.3E-06	240028	leucyl/cystinyl aminopeptidase
Lox	1.98	0.98	9.0E-05	4.3E-06	16948	lysyl oxidase
Lpar1	2.05	1.04	8.2E-06	2.7E-07	14745	lysophosphatidic acid receptor 1
Lrp11	1.90	0.93	2.0E-05	7.5E-07	237253	low density lipoprotein receptor-related protein 11
Lrrc1	3.24	1.69	1.3E-04	6.6E-06	214345	leucine rich repeat containing 1
Lrrc38	0.31	-1.68	4.6E-07	9.8E-09	242735	leucine rich repeat containing 38
Lrrc3b	6.38	2.67	1.1E-23	2.0E-26	218763	leucine rich repeat containing 3B
Lrrcc1	1.90	0.92	6.5E-05	2.9E-06	71710	leucine rich repeat and coiled-coil domain containing 1
Lrrn1	2.97	1.57	6.4E-08	1.1E-09	16979	leucine rich repeat protein 1, neuronal
Lsp1	1.61	0.68	1.0E-05	3.5E-07	16985	lymphocyte specific 1
Lypd6	0.26	-1.95	3.5E-07	7.2E-09	320343	LY6/PLAUR domain containing 6
Lyplal1	2.16	1.11	6.9E-05	3.2E-06	226791	lysophospholipase-like 1
Lysmd2	2.70	1.43	2.0E-11	1.6E-13	70082	LysM, putative peptidoglycan-binding, domain containing 2
Lysmd3	0.52	-0.93	7.5E-05	3.5E-06	80289	LysM, putative peptidoglycan-binding, domain containing 3
Lyz1	2.94	1.56	2.1E-08	3.0E-10	17110	lysozyme 1
Maged2	2.36	1.24	1.2E-05	4.0E-07	80884	melanoma antigen, family D, 2
Mal	4.67	2.22	1.6E-11	1.2E-13	17153	myelin and lymphocyte protein, T cell differentiation protein
Mamdc2	3.58	1.84	5.5E-06	1.7E-07	71738	MAM domain containing 2
Man2a2	0.62	-0.69	1.1E-04	5.3E-06	140481	mannosidase 2, alpha 2
Map1a	3.13	1.64	7.3E-14	3.8E-16	17754	microtubule-associated protein 1A
Map1b	3.16	1.66	1.2E-20	2.5E-23	17755	microtubule-associated protein 1B
Map2k1	1.75	0.81	9.4E-05	4.5E-06	26395	mitogen-activated protein kinase kinase 1
Map3k7cl	3.11	1.64	2.6E-17	7.8E-20	224419	MAP3K7 C-terminal like
Mapk11	2.51	1.33	1.5E-05	5.2E-07	19094	mitogen-activated protein kinase 11
March6	0.58	-0.78	8.6E-07	2.0E-08	223455	membrane-associated ring finger (C3HC4) 6
Mark1	2.04	1.03	4.0E-06	1.2E-07	226778	MAP/microtubule affinity-regulating kinase 1
Marveld1	1.79	0.84	3.7E-05	1.5E-06	277010	MARVEL (membrane-associating) domain containing 1
Mavs	0.66	-0.61	9.9E-05	4.9E-06	228607	mitochondrial antiviral signaling protein
Mbnl1	0.52	-0.95	1.3E-04	6.5E-06	56758	muscleblind-like 1
Mbp	4.72	2.24	4.2E-11	3.5E-13	17196	myelin basic protein
Mccc1	0.44	-1.19	4.5E-06	1.3E-07	72039	methylcrotonoyl-Coenzyme A carboxylase 1 (alpha)
Mdm4	0.58	-0.78	1.1E-04	5.5E-06	17248	transformed mouse 3T3 cell double minute 4
Me2	3.92	1.97	1.2E-07	2.2E-09	107029	malic enzyme 2, NAD(+)-dependent, mitochondrial
Me3	0.56	-0.84	1.1E-06	2.8E-08	109264	malic enzyme 3, NADP(+)-dependent, mitochondrial
Meg3	3.50	1.81	1.1E-14	5.1E-17	17263	maternally expressed 3
Met	0.40	-1.33	1.8E-10	1.6E-12	17295	met proto-oncogene
Metrn	2.56	1.35	1.2E-14	5.5E-17	70083	meteorin, glial cell differentiation regulator
Mettl21c	2.39	1.26	1.5E-12	9.2E-15	433294	methyltransferase like 21C
Mfap3l	1.65	0.72	6.5E-05	3.0E-06	71306	microfibrillar-associated protein 3-like
Mfap5	1.92	0.94	7.9E-05	3.7E-06	50530	microfibrillar associated protein 5
Mfhas1	4.74	2.25	4.4E-34	3.4E-37	52065	malignant fibrous histiocytoma amplified sequence 1
Mfn1	0.62	-0.69	6.9E-05	3.1E-06	67414	mitofusin 1
Mfsd7b	0.44	-1.17	3.0E-06	8.5E-08	226844	major facilitator superfamily domain containing 7B
Mga	0.58	-0.78	2.1E-05	8.1E-07	29808	MAX gene associated
Mgmt	2.55	1.35	8.2E-05	3.9E-06	17314	O-6-methylguanine-DNA methyltransferase
Mgp	1.85	0.89	2.5E-09	3.0E-11	17313	matrix Gla protein
Mgst1	3.13	1.65	3.8E-08	5.9E-10	56615	microsomal glutathione S-transferase 1
Mib1	0.52	-0.96	1.4E-04	7.3E-06	225164	mindbomb homolog 1
Mical2	0.33	-1.59	2.6E-05	1.0E-06	320878	microtubule associated monoxygenase, calponin and LIM domain containing 2
Micall1	1.77	0.82	6.1E-06	1.9E-07	27008	microtubule associated monoxygenase, calponin and LIM domain containing -like 1
Micu2	1.74	0.80	1.8E-05	6.7E-07	68514	mitochondrial calcium uptake 2
Minpp1	1.76	0.82	9.5E-05	4.6E-06	17330	multiple inositol polyphosphate histidine phosphatase 1
Mir1906-1	2.90	1.54	1.2E-05	4.2E-07	100316809	microRNA 1906-1
Mir1906-2	2.90	1.54	1.3E-05	4.5E-07	100499530	microRNA 1906-2
Mir410	9.16	3.20	3.3E-12	2.1E-14	723863	microRNA 410
Mir675	2.14	1.10	1.1E-04	5.4E-06	735280	microRNA 675
Mirg	7.56	2.92	3.3E-22	6.2E-25	100040724	miRNA containing gene
Mllt11	1.86	0.90	5.1E-09	6.4E-11	56772	myeloid/lymphoid or mixed-lineage leukemia (trithorax homolog); translocated to, 11
Mob1b	0.50	-1.01	1.9E-05	7.0E-07	68473	MOB kinase activator 1B
Mpp1	1.82	0.87	1.6E-07	3.0E-09	17524	membrane protein, palmitoylated
Mpp3	0.35	-1.52	3.3E-09	3.9E-11	13384	membrane protein, palmitoylated 3 (MAGUK p55 subfamily member 3)
Mpz	4.52	2.18	2.3E-12	1.5E-14	17528	myelin protein zero
Mreg	0.54	-0.90	2.3E-06	6.3E-08	381269	melanoregulin
Msln	24.78	4.63	6.8E-10	7.1E-12	56047	mesothelin
Mt1	7.22	2.85	6.2E-11	5.3E-13	17748	metallothionein 1
Mt2	29.49	4.88	2.7E-17	8.4E-20	17750	metallothionein 2
Mt3	9.33	3.22	4.5E-10	4.6E-12	17751	metallothionein 3
Mthfd2	2.21	1.14	1.2E-04	6.1E-06	17768	methylenetetrahydrofolate dehydrogenase (NAD+ dependent), methenyltetrahydrofolate cyclohydrolase
Mum1l1	0.49	-1.04	5.4E-05	2.4E-06	245631	melanoma associated antigen (mutated) 1-like 1
Mustn1	3.33	1.74	6.0E-07	1.3E-08	66175	musculoskeletal, embryonic nuclear protein 1
Mut	0.56	-0.84	6.6E-06	2.1E-07	17850	methylmalonyl-Coenzyme A mutase
Mvp	1.86	0.90	1.9E-09	2.1E-11	78388	major vault protein
Myh1	1.59	0.67	3.4E-05	1.4E-06	17879	myosin, heavy polypeptide 1, skeletal muscle, adult
Myh3	27.47	4.78	1.8E-18	4.5E-21	17883	myosin, heavy polypeptide 3, skeletal muscle, embryonic
Myh7	9.97	3.32	6.3E-06	2.0E-07	140781	myosin, heavy polypeptide 7, cardiac muscle, beta
Myh8	34.29	5.10	3.7E-13	2.1E-15	17885	myosin, heavy polypeptide 8, skeletal muscle, perinatal
Myl12a	1.86	0.89	3.2E-05	1.3E-06	67268	myosin, light chain 12A, regulatory, non-sarcomeric
Myl2	4.74	2.25	1.2E-04	6.1E-06	17906	myosin, light polypeptide 2, regulatory, cardiac, slow
Myl4	23.04	4.53	2.1E-19	4.8E-22	17896	myosin, light polypeptide 4
Myl6	2.02	1.02	5.2E-12	3.5E-14	17904	myosin, light polypeptide 6, alkali, smooth muscle and non-muscle
Myl6b	4.57	2.19	1.6E-07	2.9E-09	216459	myosin, light polypeptide 6B
Myo5c	0.23	-2.12	8.2E-06	2.7E-07	208943	myosin VC
Myoc	3.27	1.71	4.4E-10	4.4E-12	17926	myocilin
Myog	3.09	1.63	3.5E-05	1.4E-06	17928	myogenin
Myom3	4.95	2.31	2.0E-18	5.1E-21	242702	myomesin family, member 3
Myoz2	4.90	2.29	3.5E-16	1.3E-18	59006	myozenin 2
Myoz3	0.54	-0.90	2.0E-07	3.8E-09	170947	myozenin 3
Nacad	3.39	1.76	1.6E-09	1.9E-11	192950	NAC alpha domain containing
Nat8l	5.95	2.57	7.9E-07	1.8E-08	269642	N-acetyltransferase 8-like
Nbeal1	0.47	-1.08	2.6E-05	1.0E-06	269198	neurobeachin like 1
Ncam1	4.18	2.06	9.5E-06	3.2E-07	17967	neural cell adhesion molecule 1
Nckap1l	3.83	1.94	1.3E-11	9.4E-14	105855	NCK associated protein 1 like
Ncmap	3.41	1.77	7.0E-08	1.2E-09	230822	noncompact myelin associated protein
Ncoa1	0.56	-0.83	4.8E-08	7.8E-10	17977	nuclear receptor coactivator 1
Ncoa2	0.66	-0.60	1.8E-04	1.0E-05	17978	nuclear receptor coactivator 2
Ncs1	2.26	1.17	8.8E-06	2.9E-07	14299	neuronal calcium sensor 1
Ndn	3.70	1.89	4.7E-10	4.8E-12	17984	necdin
Ndrg1	1.90	0.93	4.1E-06	1.2E-07	17988	N-myc downstream regulated gene 1
Ndrg4	2.85	1.51	1.1E-11	8.3E-14	234593	N-myc downstream regulated gene 4

Ndufs1	0.60	-0.75	1.4E-05	5.0E-07	227197	NADH dehydrogenase (ubiquinone) Fe-S protein 1
Neb	0.39	-1.35	4.1E-09	5.0E-11	17996	nebulin
Nek7	0.57	-0.81	2.3E-07	4.4E-09	59125	NIMA (never in mitosis gene a)-related expressed kinase 7
Nes	3.05	1.61	1.6E-14	7.5E-17	18008	nestin
Net1	2.72	1.45	3.9E-08	6.2E-10	56349	neuroepithelial cell transforming gene 1
Neurl3	2.70	1.43	2.2E-10	2.1E-12	214854	neuralized homolog 3 homolog
Nfatc2	2.02	1.02	5.3E-05	2.3E-06	18019	nuclear factor of activated T cells, cytoplasmic, calcineurin dependent 2
Nfix	0.63	-0.68	6.1E-05	2.7E-06	18032	nuclear factor I/X
Nfyb	0.63	-0.66	1.5E-04	7.8E-06	18045	nuclear transcription factor-Y beta
Ninj1	2.18	1.12	3.1E-11	2.5E-13	18081	ninjurin 1
Nkd1	2.35	1.24	9.0E-05	4.3E-06	93960	naked cuticle 1 homolog
Nkx6-2	2.13	1.09	5.7E-05	2.5E-06	14912	NK6 homeobox 2
Nlrc3	10.86	3.44	7.4E-18	2.0E-20	268857	NLR family, CARD domain containing 3
Nlrp10	3.12	1.64	5.4E-08	9.0E-10	244202	NLR family, pyrin domain containing 10
Nme4	4.49	2.17	5.1E-09	6.4E-11	56520	NME/NM23 nucleoside diphosphate kinase 4
Nnat	9.18	3.20	2.7E-13	1.5E-15	18111	neuronatin
Nog	4.65	2.22	4.0E-07	8.4E-09	18121	noggin
Nol3	1.57	0.65	1.7E-05	6.1E-07	78688	nucleolar protein 3 (apoptosis repressor with CARD domain)
Nov	2.23	1.16	2.1E-05	8.1E-07	18133	nephroblastoma overexpressed gene
Nova1	2.01	1.01	1.2E-04	6.3E-06	664883	neuro-oncological ventral antigen 1
Npnt	5.44	2.44	1.0E-12	6.2E-15	114249	nephronectin
Npr3	8.20	3.04	1.2E-54	1.7E-58	18162	natriuretic peptide receptor 3
Nrcam	16.39	4.03	2.1E-12	1.3E-14	319504	neuron-glia-CAM-related cell adhesion molecule
Nrep	0.40	-1.31	3.4E-08	5.3E-10	27528	neuronal regeneration related protein
Nt5c1a	0.33	-1.59	4.6E-06	1.4E-07	230718	5'-nucleotidase, cytosolic IA
Nt5c2	2.43	1.28	7.5E-07	1.7E-08	76952	5'-nucleotidase, cytosolic II
Ntrk2	2.36	1.24	1.6E-04	8.9E-06	18212	neurotrophic tyrosine kinase, receptor, type 2
Nudt16	3.25	1.70	5.0E-17	1.6E-19	75686	nudix (nucleoside diphosphate linked moiety X)-type motif 16
Nudt6	0.50	-1.01	6.9E-05	3.2E-06	229228	nudix (nucleoside diphosphate linked moiety X)-type motif 6
Nup153	0.61	-0.70	1.6E-04	8.9E-06	218210	nucleoporin 153
Nup210	0.56	-0.84	2.2E-05	8.2E-07	54563	nucleoporin 210
Nup93	1.86	0.90	4.6E-05	2.0E-06	71805	nucleoporin 93
Nupr1	2.27	1.19	7.7E-08	1.3E-09	56312	nuclear protein 1
Nwd1	2.82	1.50	2.2E-05	8.2E-07	319555	NACHT and WD repeat domain containing 1
Nxn	1.74	0.80	1.2E-05	4.0E-07	18230	nucleoredoxin
Obscn	0.48	-1.07	5.3E-08	8.8E-10	380698	obscurin, cytoskeletal calmodulin and titin-interacting RhoGEF
Odc1	2.92	1.54	2.3E-12	1.5E-14	18263	ornithine decarboxylase, structural 1
Ogdh	0.55	-0.86	1.0E-07	1.8E-09	18293	oxoglutarate dehydrogenase (lipoamide)
Ogdhl	2.09	1.06	4.5E-05	1.9E-06	239017	oxoglutarate dehydrogenase-like
Orai2	4.65	2.22	1.3E-15	5.0E-18	269717	ORAI calcium release-activated calcium modulator 2
Ormdl3	1.56	0.65	4.3E-05	1.8E-06	66612	ORM1-like 3
Osbpl3	5.15	2.36	6.1E-15	2.6E-17	71720	oxysterol binding protein-like 3
Osbpl6	0.49	-1.01	8.4E-05	4.0E-06	99031	oxysterol binding protein-like 6
Oscp1	3.58	1.84	2.3E-05	8.7E-07	230751	organic solute carrier partner 1
Osgin2	2.07	1.05	1.4E-06	3.6E-08	209212	oxidative stress induced growth inhibitor family member 2
Otulin	1.85	0.89	1.7E-05	6.1E-07	209212	OTU deubiquitinase with linear linkage specificity
Oxct1	0.47	-1.08	2.4E-05	9.5E-07	67041	3-oxoacid CoA transferase 1
P2ry1	0.42	-1.23	9.1E-09	1.2E-10	18441	purinergic receptor P2Y, G-protein coupled 1
P4ha1	2.03	1.02	1.3E-05	4.7E-07	18451	procollagen-proline, 2-oxoglutarate 4-dioxygenase (proline 4-hydroxylase), alpha 1 polypeptide
Pag1	0.49	-1.04	4.6E-05	2.0E-06	94212	phosphoprotein associated with glycosphingolipid microdomains 1
Palm2	3.94	1.98	1.3E-06	3.1E-08	242481	paralemmin 2
Pamr1	2.40	1.26	6.6E-07	1.5E-08	210622	peptidase domain containing associated with muscle regeneration 1
Paqr7	2.32	1.22	1.4E-08	1.9E-10	71904	progestin and adipoQ receptor family member VII
Paqr8	4.05	2.02	2.5E-06	6.8E-08	74229	progestin and adipoQ receptor family member VIII
Pax7	0.22	-2.16	5.3E-07	1.1E-08	18509	paired box gene 7
Pcbd1	9.97	3.32	6.8E-20	1.5E-22	13180	pterin 4 alpha carbinolamine dehydratase/dimerization cofactor of hepatocyte nuclear factor 1 alpha (TCF1) 1
Pcdhb20	0.35	-1.53	3.6E-06	1.0E-07	93891	protocadherin beta 20
Pck1	26.45	4.73	1.1E-16	3.5E-19	18534	phosphoenolpyruvate carboxykinase 1, cytosolic
Pcmtd1	0.63	-0.67	8.3E-05	4.0E-06	319263	protein-L-isoaspartate (D-aspartate) O-methyltransferase domain containing 1
Pcmtd2	0.57	-0.81	1.5E-05	5.4E-07	245867	protein-L-isoaspartate (D-aspartate) O-methyltransferase domain containing 2
Pcnxl4	2.59	1.38	3.7E-09	4.4E-11	67708	pecanex-like 4
Pcsk5	2.42	1.27	1.9E-05	7.2E-07	18552	proprotein convertase subtilisin/kexin type 5
Pcx	0.58	-0.79	1.7E-05	6.1E-07	18563	pyruvate carboxylase
Pde4a	0.44	-1.20	8.2E-07	1.9E-08	18577	phosphodiesterase 4A, cAMP specific
Pde4b	0.45	-1.16	1.1E-08	1.4E-10	18578	phosphodiesterase 4B, cAMP specific
Pde4dip	0.41	-1.29	4.1E-08	6.6E-10	83679	phosphodiesterase 4D interacting protein (myomegalin)
Pdk1	1.78	0.83	1.2E-07	2.1E-09	228026	pyruvate dehydrogenase kinase, isoenzyme 1
Pdk3	5.22	2.38	3.3E-27	4.6E-30	236900	pyruvate dehydrogenase kinase, isoenzyme 3
Pdlim3	1.55	0.63	1.1E-04	5.6E-06	53318	PDZ and LIM domain 3
Pdpr	0.43	-1.20	5.7E-05	2.5E-06	319518	pyruvate dehydrogenase phosphatase regulatory subunit
Pds5b	0.62	-0.68	8.8E-05	4.2E-06	100710	PDS5, regulator of cohesion maintenance, homolog B
Pdzd3	3.80	1.92	3.6E-05	1.5E-06	170761	PDZ domain containing 3
Pdzrn3	0.53	-0.91	8.2E-07	1.9E-08	55983	PDZ domain containing RING finger 3
Pdzrn4	3.96	1.98	3.3E-05	1.3E-06	239618	PDZ domain containing RING finger 4
Peg10	18.78	4.23	3.4E-13	1.9E-15	170676	paternally expressed 10
Penk	2.35	1.23	1.1E-06	2.6E-08	18619	preproenkephalin
Perp	2.85	1.51	5.1E-08	8.4E-10	64058	PERP, TP53 apoptosis effector
Pfkl	2.26	1.17	1.8E-04	9.9E-06	18641	phosphofructokinase, liver, B-type
Pfkm	0.58	-0.78	1.5E-04	7.8E-06	18642	phosphofructokinase, muscle
Pgm2l1	0.44	-1.20	9.2E-05	4.4E-06	70974	phosphoglucomutase 2-like 1
Phka1	0.40	-1.32	2.2E-05	8.3E-07	18679	phosphorylase kinase alpha 1
Phkb	0.55	-0.86	3.1E-05	1.2E-06	102093	phosphorylase kinase beta
Phkg1	0.42	-1.25	5.7E-06	1.7E-07	18682	phosphorylase kinase gamma 1
Pik3r5	4.24	2.08	8.6E-08	1.5E-09	320207	phosphoinositide-3-kinase, regulatory subunit 5, p101
Pink1	0.57	-0.81	9.7E-07	2.3E-08	68943	PTEN induced putative kinase 1
Pitpnc1	0.45	-1.14	2.6E-08	3.9E-10	71795	phosphatidylinositol transfer protein, cytoplasmic 1
Pknox2	0.59	-0.75	5.3E-06	1.6E-07	208076	Pbx/knotted 1 homeobox 2
Pla2g7	1.85	0.89	1.2E-08	1.6E-10	27226	phospholipase A2, group VII (platelet-activating factor acetylhydrolase, plasma)
Plb1	2.36	1.24	4.1E-05	1.7E-06	665270	phospholipase B1
Plce1	2.01	1.01	1.8E-05	6.5E-07	74055	phospholipase C, epsilon 1
Plekha4	2.74	1.45	3.5E-06	1.0E-07	69217	pleckstrin homology domain containing, family A (phosphoinositide binding specific) member 4
Plekha6	1.75	0.81	3.1E-06	9.0E-08	240753	pleckstrin homology domain containing, family A member 6
Plekha7	3.41	1.77	4.8E-05	2.1E-06	233765	pleckstrin homology domain containing, family A member 7
Plekhh3	0.55	-0.86	8.3E-05	3.9E-06	217198	pleckstrin homology domain containing, family H (with MyTH4 domain) member 3
Plekho1	2.89	1.53	1.4E-12	8.8E-15	67220	pleckstrin homology domain containing, family O member 1
Plin1	9.45	3.24	3.6E-13	2.1E-15	103968	perilipin 1
Plin5	0.46	-1.12	8.8E-07	2.1E-08	66968	perilipin 5
Pllp	2.72	1.44	1.1E-04	5.8E-06	67801	plasma membrane proteolipid
Plp1	2.78	1.47	3.1E-07	6.3E-09	18823	proteolipid protein (myelin) 1
Plp2	2.40	1.26	2.3E-05	8.7E-07	18824	proteolipid protein 2
Plvap	2.28	1.19	4.0E-07	8.5E-09	84094	plasmalemma vesicle associated protein
Plxna4	2.21	1.14	8.6E-08	1.5E-09	243743	plexin A4
Plxnb2	2.43	1.28	1.4E-05	5.0E-07	140570	plexin B2
Pmm1	1.59	0.66	1.7E-04	9.4E-06	29858	phosphomannomutase 1
Pmp22	3.77	1.91	4.4E-10	4.4E-12	18858	peripheral myelin protein 22
Pon2	2.01	1.01	3.8E-10	3.8E-12	330260	paraoxonase 2
Popdc3	2.02	1.02	1.9E-05	7.2E-07	78977	popeye domain containing 3
Postn	2.26	1.18	6.6E-07	1.5E-08	50706	periostin, osteoblast specific factor
Pou3f1	2.39	1.26	9.5E-05	4.6E-06	18991	POU domain, class 3, transcription factor 1
Ppapdc3	0.51	-0.98	7.9E-07	1.8E-08	227721	phosphatidic acid phosphatase type 2 domain containing 3
Ppara	0.50	-0.99	1.7E-05	6.3E-07	19013	peroxisome proliferator activated receptor alpha
Ppfibp1	2.05	1.03	7.3E-07	1.7E-08	67533	PTPRF interacting protein, binding protein 1 (liprin beta 1)
Ppm1l	0.50	-1.00	9.0E-06	3.0E-07	242083	protein phosphatase 1 (formerly 2C)-like
Ppp1r3a	0.44	-1.18	3.3E-08	5.0E-10	140491	protein phosphatase 1, regulatory (inhibitor) subunit 3A
Pqlc1	1.84	0.88	1.2E-05	4.0E-07	66943	PQ loop repeat containing 1
Prdm8	3.81	1.93	1.4E-07	2.6E-09	77630	PR domain containing 8
Prg4	2.28	1.19	9.4E-05	4.5E-06	96875	proteoglycan 4 (megakaryocyte stimulating factor, articular superficial zone protein)
Prima1	0.14	-2.84	5.8E-12	4.0E-14	170952	proline rich membrane anchor 1
Prkaa2	0.51	-0.97	1.2E-07	2.1E-09	108079	protein kinase, AMP-activated, alpha 2 catalytic subunit
Prkag3	0.50	-1.01	3.3E-05	1.3E-06	241113	protein kinase, AMP-activated, gamma 3 non-catatlytic subunit
Prkar1a	2.67	1.42	1.1E-08	1.4E-10	19084	protein kinase, cAMP dependent regulatory, type I, alpha
Prkar2b	5.69	2.51	6.6E-11	5.6E-13	19088	protein kinase, cAMP dependent regulatory, type II beta
Prkcd	2.15	1.10	3.2E-07	6.6E-09	18753	protein kinase C, delta

Prkcq	0.57	-0.80	1.5E-05	5.2E-07	18761	protein kinase C, theta
Prkcz	4.29	2.10	1.1E-06	2.8E-08	18762	protein kinase C, zeta
Prmt2	2.42	1.27	1.6E-05	5.9E-07	15468	protein arginine N-methyltransferase 2
Prodh	2.29	1.19	7.5E-05	3.5E-06	19125	proline dehydrogenase
Proser2	3.55	1.83	9.6E-12	6.8E-14	227545	proline and serine-rich protein
Prps1l3	0.60	-0.74	7.1E-06	2.3E-07	328099	phosphoribosyl pyrophosphate synthetase 1-like 3
Prr32	84.71	6.40	7.6E-72	5.0E-76	68800	proline rich 32
Prrx2	4.69	2.23	1.0E-10	9.2E-13	20204	paired related homeobox 2
Prss12	2.81	1.49	1.5E-05	5.2E-07	19142	protease, serine, 12 neurotrypsin (motopsin)
Prune2	6.69	2.74	1.7E-30	1.8E-33	353211	prune homolog 2
Prx	3.07	1.62	6.2E-09	8.0E-11	19153	periaxin
Psmd8	1.94	0.96	2.3E-08	3.3E-10	57296	proteasome (prosome, macropain) 26S subunit, non-ATPase, 8
Ptar1	0.33	-1.60	1.3E-09	1.5E-11	72351	protein prenyltransferase alpha subunit repeat containing 1
Ptdss1	1.69	0.76	1.9E-06	5.0E-08	19210	phosphatidylserine synthase 1
Ptpdc1	1.82	0.86	1.8E-05	6.6E-07	218232	protein tyrosine phosphatase domain containing 1
Ptpn3	0.46	-1.13	1.8E-05	6.7E-07	545622	protein tyrosine phosphatase, non-receptor type 3
Ptpn4	0.39	-1.37	4.7E-10	4.7E-12	19258	protein tyrosine phosphatase, non-receptor type 4
Ptpru	4.07	2.02	1.4E-04	7.2E-06	19273	protein tyrosine phosphatase, receptor type, U
Pygl	2.36	1.24	5.0E-05	2.2E-06	110095	liver glycogen phosphorylase
Pygm	0.55	-0.87	5.9E-06	1.8E-07	19309	muscle glycogen phosphorylase
Qsox1	2.36	1.25	3.4E-11	2.7E-13	104009	quiescin Q6 sulfhydryl oxidase 1
Rab13	3.22	1.69	2.9E-18	7.5E-21	68328	RAB13, member RAS oncogene family
Rab15	6.30	2.66	8.2E-34	6.7E-37	104886	RAB15, member RAS oncogene family
Rab27b	4.57	2.19	1.7E-08	2.4E-10	80718	RAB27b, member RAS oncogene family
Ralgapa2	1.80	0.85	1.3E-06	3.3E-08	241694	Ral GTPase activating protein, alpha subunit 2 (catalytic)
Ramp1	0.50	-1.00	1.2E-04	6.4E-06	51801	receptor (calcitonin) activity modifying protein 1
Rapgef4	2.83	1.50	1.5E-05	5.2E-07	56508	Rap guanine nucleotide exchange factor (GEF) 4
Rasd1	4.98	2.32	4.9E-07	1.0E-08	19416	RAS, dexamethasone-induced 1
Rasgrp3	0.60	-0.75	3.6E-05	1.5E-06	240168	RAS, guanyl releasing protein 3
Rassf7	54.82	5.78	2.3E-17	6.9E-20	66985	Ras association (RalGDS/AF-6) domain family (N-terminal) member 7
Rbp4	5.74	2.52	1.9E-09	2.2E-11	19662	retinol binding protein 4, plasma
Reep1	0.63	-0.68	6.3E-05	2.8E-06	52250	receptor accessory protein 1
Reep6	3.20	1.68	2.0E-06	5.3E-08	70335	receptor accessory protein 6
Reln	2.09	1.06	3.5E-05	1.4E-06	19699	reelin
Retn	4.52	2.18	4.2E-06	1.2E-07	57264	resistin
Retnla	1.67	0.74	7.2E-05	3.3E-06	57262	resistin like alpha
Rev3l	0.59	-0.77	1.7E-05	6.2E-07	19714	REV3-like, catalytic subunit of DNA polymerase zeta RAD54 like
Rgcc	5.14	2.36	7.3E-25	1.2E-27	66214	regulator of cell cycle
Rgs11	8.00	3.00	1.3E-14	6.0E-17	50782	regulator of G-protein signaling 11
Rgs12	3.29	1.72	2.4E-05	9.3E-07	71729	regulator of G-protein signaling 12
Rgs5	0.47	-1.08	2.3E-06	6.3E-08	19737	regulator of G-protein signaling 5
Rhod	3.74	1.90	3.8E-05	1.6E-06	11854	ras homolog gene family, member D
Rhpn2	4.58	2.20	1.9E-16	6.5E-19	52428	rhophilin, Rho GTPase binding protein 2
Rian	5.20	2.38	3.0E-39	1.9E-42	75745	RNA imprinted and accumulated in nucleus
Rin1	5.60	2.48	2.6E-08	3.9E-10	225870	Ras and Rab interactor 1
Rmi2	0.20	-2.32	8.4E-06	2.7E-07	223970	RMI2, RecQ mediated genome instability 2, homolog
Rnf217	2.08	1.05	6.1E-06	1.9E-07	268291	ring finger protein 217
Rora	0.38	-1.40	4.0E-05	1.7E-06	19883	RAR-related orphan receptor alpha
Rph3al	0.48	-1.04	9.6E-05	4.7E-06	380714	rabphilin 3A-like (without C2 domains)
Rpl3l	0.52	-0.95	5.7E-05	2.5E-06	66211	ribosomal protein L3-like
Rps2	1.64	0.71	1.3E-04	6.5E-06	16898	ribosomal protein S2
Rps20	1.77	0.82	1.1E-04	5.4E-06	67427	ribosomal protein S20
Rps6ka2	0.56	-0.84	6.3E-06	2.0E-07	20112	ribosomal protein S6 kinase, polypeptide 2
Rps6ka5	0.50	-0.99	6.2E-07	1.4E-08	73086	ribosomal protein S6 kinase, polypeptide 5
Rrad	5.44	2.44	2.3E-07	4.6E-09	56437	Ras-related associated with diabetes
Rras	3.32	1.73	5.9E-13	3.5E-15	20130	Harvey rat sarcoma oncogene, subgroup R
Rras2	2.31	1.21	4.2E-07	8.9E-09	66922	related RAS viral (r-ras) oncogene homolog 2
Rrm1	0.56	-0.83	7.8E-05	3.7E-06	20133	ribonucleotide reductase M1
Rspo3	3.44	1.78	2.2E-07	4.3E-09	72780	R-spondin 3 homolog
Rtl1	5.74	2.52	1.4E-08	1.9E-10	353326	retrotransposon-like 1
Rtn2	0.57	-0.80	3.8E-05	1.6E-06	20167	reticulon 2 (Z-band associated protein)
Runx1	7.30	2.87	4.0E-10	3.9E-12	12394	runt related transcription factor 1
Ryr1	0.40	-1.33	1.4E-15	5.5E-18	20190	ryanodine receptor 1, skeletal muscle
Ryr3	6.82	2.77	9.0E-12	6.3E-14	20192	ryanodine receptor 3
S100a13	2.00	1.00	8.2E-05	3.8E-06	20196	S100 calcium binding protein A13
S100a4	14.78	3.89	1.8E-32	1.7E-35	20198	S100 calcium binding protein A4
S100a6	2.65	1.41	8.0E-10	8.4E-12	20200	S100 calcium binding protein A6 (calcyclin)
S100b	3.01	1.59	7.3E-07	1.7E-08	20203	S100 protein, beta polypeptide, neural
Sacs	0.59	-0.77	2.1E-05	8.1E-07	50720	sacsin
Sap30	1.90	0.93	3.2E-05	1.3E-06	60406	sin3 associated polypeptide
Sat1	2.37	1.25	8.1E-18	2.2E-20	20229	spermidine/spermine N1-acetyl transferase 1
Scamp5	1.92	0.94	6.8E-06	2.1E-07	56807	secretory carrier membrane protein 5
Scand1	2.17	1.12	2.4E-05	9.4E-07	19018	SCAN domain-containing 1
Scarf2	1.89	0.92	9.0E-06	3.0E-07	224024	scavenger receptor class F, member 2
Scd1	2.75	1.46	1.2E-13	6.3E-16	20249	stearoyl-Coenzyme A desaturase 1
Scd2	2.23	1.16	1.2E-07	2.1E-09	20250	stearoyl-Coenzyme A desaturase 2
Scg3	3.74	1.90	1.6E-06	4.0E-08	20255	secretogranin III
Scn2a1	23.75	4.57	6.5E-46	2.1E-49	110876	sodium channel, voltage-gated, type II, alpha 1
Scn4a	0.51	-0.96	1.5E-09	1.7E-11	110880	sodium channel, voltage-gated, type IV, alpha
Scn4b	0.55	-0.86	7.2E-05	3.3E-06	399548	sodium channel, type IV, beta
Scn5a	8.13	3.02	1.5E-05	5.1E-07	20271	sodium channel, voltage-gated, type V, alpha
Scube2	3.96	1.99	4.6E-08	7.5E-10	56788	signal peptide, CUB domain, EGF-like 2
Scx	3.05	1.61	8.4E-10	8.9E-12	20289	scleraxis
Sdc4	1.80	0.85	6.3E-06	1.9E-07	20971	syndecan 4
Sdha	0.56	-0.84	4.0E-07	8.3E-09	66945	succinate dehydrogenase complex, subunit A, flavoprotein (Fp)
Sec14l5	0.34	-1.56	4.5E-07	9.5E-09	665119	SEC14-like 5
Sec31b	0.38	-1.40	8.7E-05	4.2E-06	240667	Sec31 homolog B
Sema3b	2.42	1.28	2.1E-07	4.0E-09	20347	sema domain, immunoglobulin domain (Ig), short basic domain, secreted, (semaphorin) 3B
Sema6c	0.52	-0.93	3.7E-08	5.9E-10	20360	sema domain, transmembrane domain (TM), and cytoplasmic domain, (semaphorin) 6C
Sept3	4.18	2.06	3.3E-09	4.0E-11	24050	septin 3
Sept5	2.68	1.42	1.0E-05	3.4E-07	18951	septin 5
Serf1	2.86	1.52	2.1E-06	5.7E-08	20365	small EDRK-rich factor 1
Serpina3c	6.64	2.73	7.3E-09	9.4E-11	16625	serine (or cysteine) peptidase inhibitor, clade A, member 3C
Serpina3n	11.35	3.50	1.1E-04	5.7E-06	20716	serine (or cysteine) peptidase inhibitor, clade A, member 3N
Serpinb1a	5.66	2.50	2.2E-24	3.6E-27	66222	serine (or cysteine) peptidase inhibitor, clade B, member 1a
Serpinb1c	3.52	1.81	1.3E-05	4.6E-07	380839	serine (or cysteine) peptidase inhibitor, clade B, member 1c
Serpinb6a	4.13	2.05	3.4E-30	3.7E-33	20719	serine (or cysteine) peptidase inhibitor, clade B, member 6a
Serpine1	2.61	1.39	2.9E-05	1.2E-06	18787	serine (or cysteine) peptidase inhibitor, clade E, member 1
Serpine2	0.57	-0.81	1.4E-06	3.5E-08	20720	serine (or cysteine) peptidase inhibitor, clade E, member 2
Sfn	3.43	1.78	6.4E-07	1.4E-08	55948	stratifin
Sgcb	0.61	-0.71	2.4E-06	6.6E-08	24051	sarcoglycan, beta (dystrophin-associated glycoprotein)
Sgpp1	0.64	-0.64	1.5E-04	7.7E-06	81535	sphingosine-1-phosphate phosphatase 1
Sh3bp2	2.82	1.50	7.0E-07	1.6E-08	24055	SH3-domain binding protein 2
Shbg	3.65	1.87	6.8E-06	2.2E-07	20415	sex hormone binding globulin
Shc4	3.34	1.74	5.2E-07	1.1E-08	271849	SHC (Src homology 2 domain containing) family, member 4
Shisa3	3.07	1.62	3.3E-08	5.0E-10	330096	shisa homolog 3
Shmt1	0.53	-0.93	1.4E-04	7.1E-06	20425	serine hydroxymethyltransferase 1 (soluble)
Shprh	0.41	-1.28	1.1E-07	1.9E-09	268281	SNF2 histone linker PHD RING helicase
Sik1	1.97	0.98	1.4E-04	7.3E-06	17691	salt inducible kinase 1
Sim1	0.30	-1.72	9.3E-07	2.2E-08	20464	single-minded homolog 1
Sirpa	1.95	0.96	5.4E-05	2.3E-06	19261	signal-regulatory protein alpha
Six4	0.56	-0.83	1.5E-05	5.3E-07	20474	sine oculis-related homeobox 4
Skap2	1.82	0.86	3.3E-05	1.3E-06	54353	src family associated phosphoprotein 2
Ski	1.52	0.61	1.1E-04	5.6E-06	20481	ski sarcoma viral oncogene homolog
Slc16a10	0.49	-1.03	1.8E-06	4.8E-08	72472	solute carrier family 16 (monocarboxylic acid transporters), member 10
Slc1a1	2.34	1.22	2.0E-08	2.8E-10	20510	solute carrier family 1 (neuronal/epithelial high affinity glutamate transporter, system Xag), member 1
Slc1a5	2.57	1.36	2.8E-10	2.7E-12	20514	solute carrier family 1 (neutral amino acid transporter), member 5
Slc22a23	0.58	-0.78	1.1E-04	5.3E-06	73102	solute carrier family 22, member 23
Slc24a2	4.62	2.21	6.9E-05	3.1E-06	76376	solute carrier family 24 (sodium/potassium/calcium exchanger), member 2
Slc25a12	0.64	-0.64	1.6E-04	8.6E-06	78830	solute carrier family 25 (mitochondrial carrier, Aralar), member 12
Slc25a24	4.51	2.17	2.5E-07	4.8E-09	229731	solute carrier family 25 (mitochondrial carrier, phosphate carrier), member 24
Slc2a12	0.45	-1.16	1.4E-07	2.7E-09	353169	solute carrier family 2 (facilitated glucose transporter), member 12
Slc35f5	1.91	0.93	2.2E-05	8.3E-07	74150	solute carrier family 35, member F5

Slc36a2	7.38	2.88	1.3E-11	9.9E-14	246049	solute carrier family 36 (proton/amino acid symporter), member 2
Slc37a2	2.85	1.51	1.7E-05	6.3E-07	56857	solute carrier family 37 (glycerol-3-phosphate transporter), member 2
Slc37a4	0.57	-0.80	7.1E-05	3.3E-06	14385	solute carrier family 37 (glucose-6-phosphate transporter), member 4
Slc40a1	0.30	-1.74	3.2E-11	2.6E-13	53945	solute carrier family 40 (iron-regulated transporter), member 1
Slc43a1	0.48	-1.06	7.5E-05	3.5E-06	72401	solute carrier family 43, member 1
Slc4a3	2.76	1.47	4.7E-06	1.4E-07	20536	solute carrier family 4 (anion exchanger), member 3
Slc6a17	4.86	2.28	3.2E-06	9.1E-08	229706	solute carrier family 6 (neurotransmitter transporter), member 17
Slc7a10	3.10	1.63	3.4E-06	9.8E-08	53896	solute carrier family 7 (cationic amino acid transporter, y+ system), member 10
Sln	51.97	5.70	3.9E-46	1.0E-49	66402	sarcolipin
Smad3	0.53	-0.92	8.0E-06	2.6E-07	17127	SMAD family member 3
Smcr8	0.49	-1.02	1.8E-04	9.8E-06	237782	Smith-Magenis syndrome chromosome region, candidate 8 homolog
Smim3	3.66	1.87	1.6E-11	1.2E-13	106878	small integral membrane protein 3
Smoc1	5.61	2.49	1.3E-06	3.1E-08	64075	SPARC related modular calcium binding 1
Smpx	1.99	0.99	9.7E-10	1.0E-11	66106	small muscle protein, X-linked
Sms	1.87	0.90	4.9E-08	8.0E-10	20603	spermine synthase
Sncg	3.51	1.81	6.7E-07	1.5E-08	20618	synuclein, gamma
Snhg5	2.51	1.33	9.8E-08	1.7E-09	72655	small nucleolar RNA host gene 5
Snx10	2.07	1.05	6.7E-08	1.1E-09	71982	sorting nexin 10
Sobp	0.54	-0.90	7.1E-07	1.6E-08	109205	sine oculis-binding protein homolog
Sod2	0.59	-0.75	2.9E-05	1.1E-06	20656	superoxide dismutase 2, mitochondrial
Sod3	2.95	1.56	3.9E-18	1.0E-20	20657	superoxide dismutase 3, extracellular
Son	0.53	-0.93	1.4E-06	3.5E-08	20658	Son DNA binding protein
Sorbs2	3.03	1.60	4.7E-27	6.9E-30	234214	sorbin and SH3 domain containing 2
Sorbs3	1.60	0.68	1.1E-04	5.6E-06	20410	sorbin and SH3 domain containing 3
Sord	3.08	1.62	1.1E-27	1.5E-30	20322	sorbitol dehydrogenase
Sox11	8.46	3.08	2.9E-05	1.2E-06	20666	SRY-box containing gene 11
Sox6	0.61	-0.72	1.0E-04	4.9E-06	20679	SRY-box containing gene 6
Sp4	0.44	-1.18	2.9E-06	8.1E-08	20688	trans-acting transcription factor 4
Spin1	see Supplementary Table 1e				20729	spindlin 1
Spint2	2.66	1.41	2.5E-05	9.6E-07	20733	serine protease inhibitor, Kunitz type 2
Spock2	2.27	1.19	9.9E-05	4.9E-06	94214	sparc/osteonectin, cwcv and kazal-like domains proteoglycan 2
Spp1	17.18	4.10	1.7E-15	6.8E-18	20750	secreted phosphoprotein 1
Spred3	4.27	2.09	4.1E-15	1.7E-17	101809	sprouty-related, EVH1 domain containing 3
Spsb2	2.50	1.32	1.0E-05	3.5E-07	14794	splA/ryanodine receptor domain and SOCS box containing 2
Spsb4	2.25	1.17	5.9E-05	2.6E-06	211949	splA/ryanodine receptor domain and SOCS box containing 4
Src	2.33	1.22	1.4E-07	2.6E-09	20779	Rous sarcoma oncogene
Srgap3	4.56	2.19	7.3E-15	3.2E-17	259302	SLIT-ROBO Rho GTPase activating protein 3
Srxn1	1.72	0.78	1.7E-06	4.5E-08	76650	sulfiredoxin 1 homolog
St3gal2	0.57	-0.81	3.8E-08	6.0E-10	20444	ST3 beta-galactoside alpha-2,3-sialyltransferase 2
Stac3	0.52	-0.93	2.6E-07	5.2E-09	237611	SH3 and cysteine rich domain 3
Stard10	0.45	-1.16	3.5E-08	5.4E-10	56018	START domain containing 10
Stard7	0.62	-0.68	9.5E-06	3.2E-07	99138	START domain containing 7
Stat5b	0.55	-0.85	1.8E-04	9.6E-06	20851	signal transducer and activator of transcription 5B
Stau2	2.05	1.03	4.6E-09	5.7E-11	29819	staufen (RNA binding protein) homolog 2
Stbd1	2.38	1.25	5.1E-12	3.4E-14	52331	starch binding domain 1
Stmn4	2.71	1.44	1.2E-08	1.7E-10	56471	stathmin-like 4
Stom	2.08	1.05	1.1E-08	1.5E-10	13830	stomatin
Strada	1.60	0.68	7.7E-05	3.6E-06	72149	STE20-related kinase adaptor alpha
Strbp	0.53	-0.91	1.2E-04	6.1E-06	20744	spermatid perinuclear RNA binding protein
Sulf2	2.78	1.47	9.2E-11	8.1E-13	72043	sulfatase 2
Sumf2	0.47	-1.07	8.1E-05	3.8E-06	67902	sulfatase modifying factor 2
Sun2	0.59	-0.76	1.3E-06	3.4E-08	223697	Sad1 and UNC84 domain containing 2
Supt3	2.26	1.18	6.4E-08	1.1E-09	109115	suppressor of Ty 3
Synm	0.51	-0.98	3.9E-06	1.2E-07	233335	synemin, intermediate filament protein
Syt11	2.02	1.02	4.5E-05	1.9E-06	229521	synaptotagmin XI
Tbc1d1	0.58	-0.79	7.8E-05	3.6E-06	57915	TBC1 domain family, member 1
Tbc1d10b	1.68	0.75	5.5E-05	2.4E-06	68449	TBC1 domain family, member 10b
Tbkbp1	1.84	0.88	2.7E-06	7.5E-08	73174	TBK1 binding protein 1
Tcea3	0.49	-1.02	5.7E-07	1.2E-08	21401	transcription elongation factor A (SII), 3
Tceal5	2.39	1.26	3.3E-15	1.3E-17	331532	transcription elongation factor A (SII)-like 5
Tceal7	8.98	3.17	9.7E-57	9.5E-61	100040972	transcription elongation factor A (SII)-like 7
Tdrd9	19.95	4.32	1.0E-20	2.0E-23	74691	tudor domain containing 9
Tdrp	1.94	0.95	1.1E-04	5.8E-06	72148	testis development related protein
Tec	2.55	1.35	5.2E-07	1.1E-08	21682	tec protein tyrosine kinase
Tecr	0.61	-0.71	1.8E-06	4.6E-08	106529	trans-2,3-enoyl-CoA reductase
Tenm3	0.46	-1.12	2.0E-07	3.8E-09	23965	teneurin transmembrane protein 3
Tet1	0.46	-1.13	7.5E-06	2.4E-07	52463	tet methylcytosine dioxygenase 1
Tgfbi	1.94	0.95	1.1E-05	3.7E-07	21810	transforming growth factor, beta induced
Tgm2	2.41	1.27	2.1E-18	5.4E-21	21817	transglutaminase 2, C polypeptide
Thada	0.57	-0.80	1.2E-04	6.4E-06	240174	thyroid adenoma associated
Thbs1	2.16	1.11	1.7E-05	6.3E-07	21825	thrombospondin 1
Thbs4	2.75	1.46	5.6E-07	1.2E-08	21828	thrombospondin 4
Thrb	0.35	-1.53	9.2E-15	4.0E-17	21834	thyroid hormone receptor beta
Thsd7b	5.07	2.34	7.5E-12	5.2E-14	210417	thrombospondin, type I, domain containing 7B
Tiam2	3.18	1.67	2.5E-08	3.7E-10	24001	T cell lymphoma invasion and metastasis 2
Tigd4	2.27	1.19	6.4E-06	2.0E-07	403175	tigger transposable element derived 4
Timp1	9.65	3.27	6.2E-07	1.4E-08	21857	tissue inhibitor of metalloproteinase 1
Tlcd2	0.48	-1.07	3.2E-05	1.3E-06	380712	TLC domain containing 2
Tle3	1.83	0.87	5.9E-06	1.8E-07	21887	transducin-like enhancer of split 3, homolog of Drosophila E(spl)
Tln2	0.57	-0.81	2.4E-05	9.3E-07	70549	talin 2
Tlr4	2.52	1.33	2.3E-07	4.4E-09	21898	toll-like receptor 4
Tmbim1	1.80	0.84	6.3E-06	2.0E-07	69660	transmembrane BAX inhibitor motif containing 1
Tmem100	0.53	-0.92	7.7E-06	2.5E-07	67888	transmembrane protein 100
Tmem109	0.59	-0.77	5.8E-06	1.8E-07	68539	transmembrane protein 109
Tmem116	3.77	1.91	2.2E-07	4.3E-09	77462	transmembrane protein 116
Tmem132a	2.99	1.58	2.4E-08	3.5E-10	98170	transmembrane protein 132A
Tmem143	0.56	-0.84	5.8E-05	2.6E-06	70209	transmembrane protein 143
Tmem158	3.71	1.89	7.6E-06	2.5E-07	72309	transmembrane protein 158
Tmem159	1.77	0.82	5.1E-05	2.2E-06	233806	transmembrane protein 159
Tmem176b	2.07	1.05	4.2E-09	5.1E-11	65963	transmembrane protein 176B
Tmem189	2.09	1.07	1.5E-07	2.9E-09	407243	transmembrane protein 189
Tmem205	1.90	0.93	3.4E-05	1.4E-06	235043	transmembrane protein 205
Tmem233	0.60	-0.74	2.3E-05	8.9E-07	545798	transmembrane protein 233
Tmem38b	1.88	0.91	4.4E-06	1.3E-07	52076	transmembrane protein 38B
Tmem53	3.55	1.83	8.5E-11	7.4E-13	68777	transmembrane protein 53
Tmem62	3.03	1.60	3.8E-14	1.8E-16	96957	transmembrane protein 62
Tmem8c	7.32	2.87	1.1E-06	2.7E-08	66139	transmembrane protein 8C
Tmem9	1.72	0.78	8.2E-05	3.9E-06	66241	transmembrane protein 9
Tmod4	0.49	-1.02	4.6E-08	7.5E-10	50874	tropomodulin 4
Tnc	2.31	1.21	5.4E-05	2.4E-06	21923	tenascin C
Tnfrsf12a	2.41	1.27	3.9E-07	8.2E-09	27279	tumor necrosis factor receptor superfamily, member 12a
Tnfrsf23	3.15	1.66	5.2E-10	5.3E-12	79201	tumor necrosis factor receptor superfamily, member 23
Tnip2	1.97	0.98	3.2E-05	1.3E-06	231130	TNFAIP3 interacting protein 2
Tnnc1	14.54	3.86	2.9E-11	2.3E-13	21924	troponin C, cardiac/slow skeletal
Tnni1	14.97	3.90	3.0E-12	1.9E-14	21952	troponin I, skeletal, slow 1
Tnnt1	5.44	2.44	1.7E-07	3.2E-09	21955	troponin T1, skeletal, slow
Tnnt2	12.15	3.60	1.0E-09	1.1E-11	21956	troponin T2, cardiac
Tnrc18	0.59	-0.77	1.2E-04	6.2E-06	231861	trinucleotide repeat containing 18
Tnrc6b	0.62	-0.68	1.3E-04	6.8E-06	213988	trinucleotide repeat containing 6b
Tpd52l1	1.83	0.87	3.9E-08	6.3E-10	21987	tumor protein D52-like 1
Tppp3	2.10	1.07	1.0E-08	1.4E-10	67971	tubulin polymerization-promoting protein family member 3
Tprn	2.21	1.14	7.3E-05	3.4E-06	97031	taperin
Tpt1	1.87	0.90	7.0E-07	1.6E-08	22070	tumor protein, translationally-controlled 1
Traf3	2.18	1.12	3.3E-08	5.1E-10	22031	TNF receptor-associated factor 3
Tram1l1	8.35	3.06	1.4E-10	1.3E-12	229801	translocation associated membrane protein 1-like 1
Trap1	0.64	-0.65	1.0E-04	5.2E-06	68015	TNF receptor-associated protein 1
Trim47	2.04	1.03	4.7E-08	7.6E-10	217333	tripartite motif-containing 47
Trim55	0.60	-0.75	1.6E-05	5.9E-07	381485	tripartite motif-containing 55
Trip11	0.43	-1.23	3.9E-06	1.1E-07	109181	thyroid hormone receptor interactor 11
Trp63	4.38	2.13	3.3E-23	5.9E-26	22061	transformation related protein 63
Tsc22d1	2.26	1.18	2.9E-06	8.2E-08	21807	TSC22 domain family, member 1
Tsc22d4	1.61	0.69	3.4E-05	1.4E-06	78829	TSC22 domain family, member 4
Tspan17	2.03	1.02	6.6E-07	1.5E-08	74257	tetraspanin 17

Tspan5	2.71	1.44	2.5E-08	3.7E-10	56224	tetraspanin 5
Tspan9	4.92	2.30	3.6E-28	4.6E-31	109246	tetraspanin 9
Tspo	2.05	1.04	2.7E-08	4.0E-10	12257	translocator protein
Ttc37	0.55	-0.85	3.5E-06	1.0E-07	218343	tetratricopeptide repeat domain 37
Ttc39a	5.08	2.34	2.5E-10	2.4E-12	230603	tetratricopeptide repeat domain 39A
Ttc39c	2.29	1.19	8.2E-06	2.7E-07	72747	tetratricopeptide repeat domain 39C
Ttc9	5.91	2.56	6.7E-15	2.9E-17	69480	tetratricopeptide repeat domain 9
Ttll12	1.80	0.85	2.1E-05	7.9E-07	223723	tubulin tyrosine ligase-like family, member 12
Ttn	0.54	-0.89	3.0E-07	6.1E-09	22138	titin
Tuba4a	0.58	-0.80	6.1E-06	1.9E-07	22145	tubulin, alpha 4A
Tubb6	3.16	1.66	2.7E-07	5.4E-09	67951	tubulin, beta 6 class V
Uap1l1	2.25	1.17	4.5E-07	9.7E-09	227620	UDP-N-acteylglucosamine pyrophosphorylase 1-like 1
Ube3a	0.52	-0.96	2.8E-07	5.7E-09	22215	ubiquitin protein ligase E3A
Ubxn2a	0.55	-0.86	6.6E-07	1.5E-08	217379	UBX domain protein 2A
Uchl1	22.23	4.47	2.1E-32	2.0E-35	22223	ubiquitin carboxy-terminal hydrolase L1
Uck2	2.28	1.19	4.2E-05	1.8E-06	80914	uridine-cytidine kinase 2
Ufsp1	3.23	1.69	1.1E-09	1.2E-11	70240	UFM1-specific peptidase 1
Ugt8a	4.38	2.13	4.4E-06	1.3E-07	22239	UDP galactosyltransferase 8A
Uhrf1bp1l	1.93	0.95	1.3E-07	2.4E-09	75089	UHRF1 (ICBP90) binding protein 1-like
Unc45b	0.60	-0.74	8.6E-05	4.1E-06	217012	unc-45 homolog B
Ung	0.43	-1.23	1.7E-07	3.3E-09	22256	uracil DNA glycosylase
Uqcrc2	0.61	-0.72	6.0E-05	2.7E-06	67003	ubiquinol cytochrome c reductase core protein 2
Usp20	1.71	0.77	2.2E-06	5.8E-08	74270	ubiquitin specific peptidase 20
Usp45	0.56	-0.84	6.1E-05	2.7E-06	77593	ubiquitin specific petidase 45
Usp9x	0.59	-0.76	1.3E-04	7.0E-06	22284	ubiquitin specific peptidase 9, X chromosome
Utp11l	1.61	0.69	1.3E-04	6.5E-06	67205	UTP11-like, U3 small nucleolar ribonucleoprotein
Vash2	3.62	1.85	2.1E-10	1.9E-12	226841	vasohibin 2
Vim	1.84	0.88	2.7E-05	1.1E-06	22352	vimentin
Vit	3.71	1.89	3.9E-07	8.1E-09	74199	vitrin
Vps13c	0.46	-1.12	5.0E-08	8.2E-10	320528	vacuolar protein sorting 13C
Vtn	0.54	-0.89	1.0E-04	4.9E-06	22370	vitronectin
Wbscr17	2.87	1.52	6.2E-08	1.1E-09	212996	Williams-Beuren syndrome chromosome region 17 homolog
Wbscr27	2.19	1.13	4.6E-05	2.0E-06	79565	Williams Beuren syndrome chromosome region 27
Wdyhv1	1.97	0.98	1.0E-04	5.2E-06	76773	WDYHV motif containing 1
Whrn	5.33	2.41	3.0E-05	1.2E-06	73750	whirlin
Wipf3	0.55	-0.89	2.5E-08	3.6E-10	330319	WAS/WASL interacting protein family, member 3
Wls	2.28	1.19	3.0E-10	2.8E-12	68151	wntless homolog
Wnt5b	2.85	1.51	9.7E-06	3.2E-07	22419	wingless-related MMTV integration site 5B
Wsb2	1.84	0.88	1.8E-06	4.8E-08	59043	WD repeat and SOCS box-containing 2
Xpo4	0.52	-0.95	5.2E-05	2.2E-06	57258	exportin 4
Xylt1	0.33	-1.61	1.3E-06	3.2E-08	233781	xylosyltransferase 1
Ybx2	0.31	-1.69	3.2E-11	2.5E-13	53422	Y box protein 2
Zdbf2	2.83	1.50	1.5E-20	3.3E-23	73884	zinc finger, DBF-type containing 2
Zfhx4	0.48	-1.05	1.6E-06	4.0E-08	80892	zinc finger homeodomain 4
Zfp281	0.54	-0.89	2.6E-06	7.2E-08	226442	zinc finger protein 281
Zfp286	2.34	1.23	2.4E-05	9.1E-07	192651	zinc finger protein 286
Zfp365	8.30	3.05	3.6E-10	3.6E-12	216049	zinc finger protein 365
Zfp385a	2.40	1.26	9.5E-15	4.2E-17	29813	zinc finger protein 385A
Zfp46	0.63	-0.67	4.1E-05	1.7E-06	22704	zinc finger protein 46
Zfp691	0.55	-0.86	8.2E-05	3.9E-06	195522	zinc finger protein 691
Zfp697	3.39	1.76	3.6E-08	5.7E-10	242109	zinc finger protein 697
Zfp703	1.95	0.96	2.1E-06	5.6E-08	353310	zinc finger protein 703
Zfp710	0.62	-0.70	6.5E-05	2.9E-06	209225	zinc finger protein 710
Zfp827	0.43	-1.21	1.7E-07	3.2E-09	622675	zinc finger protein 827
Zfp866	0.57	-0.80	6.9E-05	3.2E-06	330788	zinc finger protein 866
Zfp871	0.60	-0.74	5.3E-05	2.3E-06	208292	zinc finger protein 871
Zfp873	2.33	1.22	7.3E-05	3.4E-06	408062	zinc finger protein 873
Zfr2	2.86	1.51	2.3E-06	6.3E-08	103406	zinc finger RNA binding protein 2
Zmat3	1.87	0.90	6.7E-05	3.0E-06	22401	zinc finger matrin type 3
Zmym2	0.61	-0.72	2.3E-06	6.4E-08	76007	zinc finger, MYM-type 2

upregulated (red)
downregulated (blue)

Supplementary Table 1e: Normalized reads at exons 1 to 6 of the *Spin1* gene in SkM of Spin1^{M5} and control mice at P21, E16.5, and E15.5.

P21

Exon	FC (Spin1^{M5} vs. Ctrl)	p-value (t-test)	average Spin1^{M5}	average Ctrl
1	1.11	6.8E-01	12.7 +/- 2.6	11.5 +/- 5.1
2	1.15	4.5E-01	53.8 +/- 16.6	46.8 +/- 5.1
3	0.94	7.4E-01	16.6 +/- 3.3	17.6 +/- 5.2
4	0.37	1.0E-02	21.8 +/- 3.4	59.5 +/- 20.0
5	1.05	6.9E-01	86.3 +/- 11.9	82.4 +/- 14.5
6	1.14	1.5E-01	917.4 +/- 116.6	803.0 +/- 75.1

E16.5

Exon	FC (Spin1^{M5} vs. Ctrl)	p-value (t-test)	average Spin1^{M5}	average Ctrl
1	1.35	9.1E-02	35.3 +/- 5.8	26.1 +/- 5.7
2	1.25	2.4E-02	120.9 +/- 11.0	96.4 +/- 9.3
3	1.11	2.4E-01	36.0 +/- 4.7	32.5 +/- 2.3
4	0.46	1.2E-04	72.4 +/- 13.1	158.7 +/- 8.3
5	1.05	5.6E-01	165.2 +/- 15.6	157.3 +/- 17.2
6	1.14	2.9E-02	2178.7 +/- 152.6	1918.2 +/- 75.3

E15.5

Exon	FC (Spin1^{M5} vs. Ctrl)	p-value (t-test)	average Spin1^{M5}	average Ctrl
1	1.10	4.3E-01	31.2 +/- 5.2	28.3 +/- 3.9
2	1.10	1.2E-01	125.7 +/- 2.5	114.7 +/- 9.7
3	1.07	2.9E-01	41.8 +/- 4.4	39.0 +/- 1.7
4	0.76	8.5E-03	125.0 +/- 4.1	165.2 +/- 15.8
5	1.07	1.2E-01	199.1 +/- 6.3	185.2 +/- 11.5
6	1.09	1.0E-01	2361.9 +/- 121.0	2162.2 +/- 139.2

The targeted exon 4 is highlighted in green.
