# Supplementary material for: The histone code reader Spin1 controls skeletal muscle development
Source: Cell Death Dis. 2017 Nov 23;8(11):e3173–. doi: 10.1038/cddis.2017.468 (PMC5775400; doi:10.1038/cddis.2017.468)
Supplement: Supplementary Figure and Table Legends [file cddis2017468x3.docx]

**Supplementary Figure and Table Legends**

**Supplementary Figure 1** Loss of Spin1 in SkM results in postnatal lethality. (**a**) Schematic representation of generation of conditional (p) and deleted (d) *Spin1* alleles. Cre recombinase recognition sites (loxP), heterotypic recognition sites for Flp recombinase (FRT and F3), and neomycin (NeoR) and puromycin (PuroR) resistance cassettes are indicated. Genotyping primers used for detection of wild-type (+), floxed, and deleted alleles are represented by red arrows. (**b**) Ubiquitous loss of *Spin1* upon crossing of *Spin1*^p/p^ mice with the *Rosa26-Cre* (R26) deleter strain. The table depicts the number of offspring obtained from matings of *Spin1*^+/d^ mice. (**c**) Quantification of Pax7-positive (red) and Spin1/Pax7-positive (yellow) nuclei of myogenic precursors in hind limb sections of Spin1^M5^ and control fetuses at E15. (**d**) Quantification of Spin1-negative [DAPI (blue)] and Spin1-positive nuclei (green) in myofibers of Spin1^M5^ and control fetuses at E15. (**c**, **d**) Error bars represent +SD, ***p < 0.001, n > 3. (**e**, **f**) Immunofluorescence (IF) staining of hind limb sections of Spin1^M5^ or control mice with antibodies directed against Spin1 (green), Tcf4 (red), Pax7 (red), or GNZ reporter (white) as indicated. Nuclei were visualized with DAPI (blue). (**e**) Arrowheads mark nuclei of fibroblasts expressing Spin1 and Tcf4. (**f**) Arrowheads mark Pax7-positive nuclei expressing Spin1 (but not Cre-dependent GNZ reporter) in SkM of control mice (top row). Arrowheads and arrows mark Pax7-positive or -negative nuclei, respectively, expressing GNZ reporter (but not Spin1) in SkM of Spin1^M5^ mice (bottom row). The figure extends data presented in Figure 1b (bottom row). (**g**) Reduced *Spin1* transcript levels in SkM of newborn Spin1^M5^ compared to control mice (n = 3 in triplicates) observed by quantitative RT-PCR analysis. *Spin1* expression was normalized to that of *Tbp* and *Hprt*. Error bars represent +SD, ***p < 0.001. (**h**) Ablation of *Spin1* in myogenic precursors upon crossing of *Spin1*^p/p^ mice with the *Myf5-Cre* deleter strain. The table depicts the number of offspring obtained from matings, in which one parent had the genotype *Spin1*^+/p +/Cre^ and the other one *Spin1*^p/p^.

**Supplementary Figure 2** SkM of Spin1^M5^ mice is characterized by necrosis and structural defects in non-necrotic fibers. Electron microscopy (EM) images of SkM samples of Spin1^M5^ and control fetuses at E16.5. Dashed lines demarcate degenerating, necrotic fibers (I-II, bottom), arrowheads mark normal mitochondria (III, top), arrows point at defective mitochondria (III, bottom), 'M' marks normal M-lines (III, top), triangles point at aberrant M-lines (III, bottom), and asterisks indicate abnormal glycogen accumulation (IV, bottom).

**Supplementary Figure 3** Transcriptome and histological analyses provide evidence for aberrant fetal myogenesis in Spin1^M5^ mice. **(a**) Detection of Ankrd1 or Ankrd2 (green) by immunofluorescence (IF) staining in transversal hind limb sections of Spin1^M5^ and control fetuses at E15. Muscle fibers and nuclei were visualized with MHC antibody (red) and DAPI (blue), respectively. (**b**) Immunofluorescence staining of Ankrd2 (green) in control fetuses at E15. IgG served as background control. (**c**, **d**) Phenotype (**c**) and pathway (**d**) analysis for the set of 2042 genes differentially expressed in control fetuses at E15.5 and E16.5. The number of genes in each category is indicated.

**Supplementary Figure 4** Deregulated basic helix-loop-helix transcription factor networks account for SkM defects in Spin1^M5^ fetuses. (**a**) Genome-wide distribution of 17106 Spin1 or 30763 H3K4me3 peaks observed in primary myoblasts by ChIP-seq. (**b**) ChIP-seq tracks for selected E16.5 DEGs. Spin1 (green) and H3K4me3 (blue) tracks were obtained by ChIP-seq analysis of primary myoblasts, Myod1 (red) tracks were obtained by analysis of previously deposited ChIP-seq data for C2C12 myoblasts (GEO dataset GSE36024). Identified peaks and promoters (TSS +/- 1 kb) are indicated by bars. E16.5 DEGs without Spin1, H3K4me3, or Myod1 promoter occupancy served as control (Ctrl).

**Supplementary Figure 5** Surviving Spin1^M5^ mice exhibit major defects in soleus, tibialis anterior, and diaphragm. (**a**) Mass of hind limb muscles [gastrocnemius (GC), plantaris (PL), tibialis anterior (TA), extensor digitorum longus (EDL), and quadriceps (QC)] of Spin1^M5^ and control mice at 30 weeks of age [n = 4 animals (muscles of left and right hind limb) in each category]. Error bars represent +SD, ***p < 0.001. (**b**) Numbers of fibers in TA (n = 3 Ctrl and n = 5 Spin1^M5^ females) and EDL (n = 4 Ctrl and n = 5 Spin1^M5^ females) at 30 weeks of age. Error bars represent +SD, ***p < 0.001 (**c**) Hematoxylin & eosin (H&E) staining of the diaphragm (DP) of Spin1^M5^ and control mice at 15 weeks of age. (**d**) Gomori (Gom) staining of gastrocnemius, soleus, TA, and EDL of Spin1^M5^ and control mice at 30 weeks of age. Blue staining indicates fibrosis. (**e**) Fiber types in soleus, plantaris, and TA of Spin1^M5^ and control mice at 15 weeks of age observed by immunofluorescence staining (IF) (top and middle rows). Tissue sections were stained with selective antibody directed against MHC-I (purple), MHC-IIb (cyan), MHC-IIa (red), and MHC-IIx (green) and images recorded by confocal microscopy. For comparison, NADH staining was included (bottom row). Fibers with abnormal NADH staining are marked with arrows. Corresponding fibers in each column of images are squared.

**Supplementary Table 1** Differential gene expression in Spin1^M5^ and control mice. (**a**, **b**, **d**) Genes differentially expressed in SkM of Spin1^M5^ and control fetuses at E15.5 (**a**) and E16.5 (**b**) or in TA of Spin1^M5^ and control mice at P21 (**d**). (**c**) Genes differentially expressed in SkM of control fetuses at E16.5 and E15.5. (**e**) Average normalized reads at exons 1 to 6 of the *Spin1* gene in SkM of Spin1^M5^ and control mice at P21, E16.5, and E15.5. *Spin1* depletion is characterized by a significant reduction of reads at the targeted exon 4 (highlighted in green). (FC, fold change; FDR, false discovery rate).
